# Coffee Consumption and Risk of Incident Gastrointestinal Disease: A Large Prospective Cohort Study

**DOI:** 10.1002/fsn3.71271

**Published:** 2025-11-27

**Authors:** Shenxuan Zhou, Fulan Hu, Zhiping Long, Hao Huang, Dapeng Li, Minjie Chu, Lei Zhang

**Affiliations:** ^1^ Department of Epidemiology, School of Public Health Nantong University Nantong Jiangsu China; ^2^ Department of Biostatistics and Epidemiology, School of Public Health, Shenzhen University Medical School Shenzhen Guangdong China; ^3^ Jinhua Center for Disease Control and Prevention Jinhua Zhejiang China; ^4^ Department of Preventive Medicine, Zhuhai Campus of Zunyi Medical University Zhuhai Guangdong China; ^5^ Department of Epidemiology and Biostatistics, School of Public Health of Wenzhou Medical University Wenzhou Zhejiang China

**Keywords:** gastrointestinal disease, risk factors, sweetened coffee, UK Biobank

## Abstract

Coffee's health effects are well studied, but its relationship with gastrointestinal diseases (GIDs) remains unclear, particularly with the frequent use of sweeteners. A prospective cohort of 147,263 participants without pre‐existing GIDs was analyzed. Coffee intake, assessed via a 24‐h dietary recall, was categorized as unsweetened, naturally sweetened, or artificially sweetened. Incident GIDs were identified through medical records. Cox proportional hazards models estimated hazard ratios (HRs) and 95% confidence intervals (CIs). Genetic predisposition was assessed using polygenic risk scores (PRS), with subgroup and sensitivity analyses conducted for robustness. Over a 12.6‐year median follow‐up, 29,118 incident GIDs occurred. Unsweetened coffee showed a dose‐dependent, U‐shaped association with lower GID risk (P‐non‐linear < 0.001), with the lowest risk at 2–4 cups/day (HR: 0.84, 95% CI: 0.81–0.88). Naturally sweetened coffee had limited benefits, while artificial sweeteners showed inconsistent associations. Protective effects were observed for GERD, gastritis/duodenitis, and biliary diseases. Unsweetened coffee mitigated genetic GID risk, with significant additive interactions for diverticulosis and cirrhosis. Findings were robust across subgroup and sensitivity analyses. Unsweetened coffee consumption is associated with a lower GID risk, supporting its inclusion in dietary recommendations. Further studies are needed to clarify underlying mechanisms and validate findings in diverse populations.

## Graphical abstract

In a large prospective cohort, unsweetened coffee consumption demonstrated a U‐shaped association with a reduced risk of multiple gastrointestinal diseases and attenuated genetic susceptibility, whereas adding sweeteners, particularly artificial ones, diminished these protective effects.

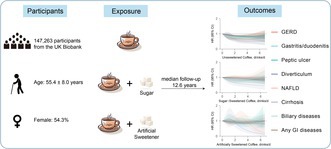

## 1 Introduction

Gastrointestinal (GI) diseases represent a significant public health challenge worldwide, contributing to substantial morbidity, healthcare costs, and reduced quality of life (Bai et al. 2024; Sperber et al. 2021; Wang et al. 2023). Despite advancements in medical interventions and preventive measures, the burden of GI diseases continues to rise, driven by complex interactions between genetic predispositions, environmental factors, and lifestyle behaviors (Avramidou et al. 2018; Machicado et al. 2021). Among modifiable lifestyle factors, dietary patterns and specific food and beverage consumption have emerged as critical areas of interest in understanding GI health (Corsello et al. 2020). Coffee, given its global ubiquity and direct interaction with the GI tract as the primary site of bioactive compound exposure, presents a compelling yet understudied research focus (Nehlig 2022; Saygili et al. 2024).

Coffee contains a complex array of compounds, including caffeine, polyphenols, and diterpenes, which influence various physiological processes (Koníčková et al. 2024; Lopes and Cunha 2024). Evidence suggests that moderate coffee consumption may exert protective effects on oral and gut microbiota, enhance motility, and stimulate gastric, biliary, and pancreatic secretions, facilitating digestion (Saygili et al. 2024). However, excessive intake (> 5 cups/day) has been associated with adverse outcomes such as gastroesophageal reflux, periodontal disease, and potential exacerbation of Crohn's disease (Nehlig 2022; Saygili et al. 2024). Critically, these dose‐dependent effects likely reflect physiological thresholds governing the transition from beneficial to detrimental impacts, underscoring the need to investigate how preparation methods and additives—notably sweeteners—modulate coffee's GI effects (Thomas and Hodges 2020).

In many populations, coffee is commonly consumed with added sugar or artificial sweeteners, which may have distinct health implications (Gyntelberg et al. 2009). Excessive sugar intake is a well‐established risk factor for metabolic disorders and chronic inflammation, both of which may contribute to GI disease development (Arnone et al. 2022; Belkova et al. 2017; Evans 2017). In contrast, artificial sweeteners remain controversial, with studies suggesting both potential metabolic benefits and adverse effects, such as dysbiosis and changes in gut function (Kossiva et al. 2024; Spencer et al. 2016). These divergent effects underscore the importance of distinguishing between unsweetened coffee and sweetened coffee when evaluating their associations with GI health.

Leveraging data from the UK Biobank, a large‐scale prospective cohort study (Sudlow et al. 2015), this research aims to address these gaps by examining the associations between unsweetened and sweetened coffee consumption and the risk of incident GI diseases. Furthermore, the study investigates how coffee consumption influences specific GI disease subtypes and explores potential population‐level differences in its effects. This study represents one of the first large‐scale investigations to differentiate the effects of unsweetened and sweetened coffee consumption on GI disease risk. The findings are expected to advance the understanding of coffee's impact on GI health, informing dietary guidelines and public health recommendations.

## 2 Materials and Methods

### 2.1 Study Design and Participants

This study utilized a prospective cohort design based on data from the UK Biobank, a large‐scale population‐based cohort that recruited over 500,000 participants aged 37–73 years between 2006 and 2010 across the United Kingdom. Participants completed baseline assessments, including questionnaires, physical measurements, and biological sample collection. Furthermore, a subset of participants was invited to complete a 24‐h dietary recall questionnaire. In this analysis, individuals who dropped out of the UK Biobank, had a history of cancer or GI disease at baseline, did not complete a 24‐h dietary recall, had unreliable dietary information, or had abnormal coffee consumption data were excluded (Figure S1). Detailed inclusion and exclusion criteria are shown in Figure S2, and the final analytical sample included 147,263 participants. The UK Biobank study was approved by the North West Multi‐Centre Research Ethics Committee, and all participants provided written informed consent.

### 2.2 Exposure Assessment

Coffee consumption data were gathered utilizing the Oxford WebQ (Ho et al. 2020). a validated web‐based 24‐h dietary recall questionnaire, completed by participants up to five times between 2009 and 2012 to account for seasonal variations in dietary habits. The questionnaire captured detailed information on the types and quantities of beverages consumed, specifically including coffee, along with any additions of sugar or artificial sweeteners (Figure S3). Based on their coffee consumption patterns, participants were stratified into four groups: Non‐consumers (no coffee reported), Unsweetened coffee consumers (coffee consumed without sugar or sweeteners in all recalls), Sugar‐sweetened coffee consumers (sugar added only), and Artificially sweetened coffee consumers (artificial sweeteners added only). Notably, 5655 overlapping consumers, as detailed in Table S1, were excluded from the analysis. Average daily coffee intake (cups/day) was determined by averaging consumption across all available recalls, and extreme outliers were subsequently excluded to ensure data accuracy.

### 2.3 Outcome Assessment

The primary outcomes of this study were the first occurrences of 15 specific GI diseases: Barrett's esophagus (BE), gastroesophageal reflux disease (GERD), gastritis and duodenitis, celiac disease, peptic ulcer, Crohn's disease, ulcerative colitis (UC), irritable bowel syndrome (IBS), diverticular disease, pancreatitis, non‐alcoholic fatty liver disease (NAFLD), cirrhosis, biliary diseases, appendicitis, and GI cancers. These conditions were identified through linkage with hospital inpatient records, and death registries in the UK Biobank, using diagnostic codes from the International Classification of Diseases, 10th Revision (ICD‐10). Detailed definitions and corresponding ICD‐10 codes are provided in Table S2. Follow‐up time was calculated from the date of recruitment to the earliest of the following: diagnosis of a GI disease, death, or the end of the follow‐up period (May 31, 2022). For participants with multiple diagnoses, the first occurrence of any of the specified GI conditions was recorded as the primary outcome. Person‐years of follow‐up were computed for each participant to account for varying follow‐up durations.

### 2.4 Assessment of Covariates

Covariates were selected based on their potential to confound the relationship between coffee consumption and GI disease risk, as identified in our previous research (Zhang et al. 2024). These included sociodemographic factors (age, sex, ethnicity, and socioeconomic status measured by the Townsend Deprivation Index, current employment status, and education level), lifestyle factors (smoking status, pack‐years of smoking, physical activity, healthy sleep pattern, and diet‐related factors, including total energy intake, total sugar intake, tea intake and adherence to dietary quality indices such as the Alternative Healthy Eating Index [AHEI]), and clinical factors basal metabolic rate [BMI], vitamin and mineral supplements, mineral and other dietary supplements, Nonsteroidal anti‐inflammatory drugs (NSAIDs) use, proton pump inhibitor (PPI) use, family history of CVD or cancer, pre‐existing long‐term chronic conditions, immune biomarkers and an aggregated inflammation score [INFLA‐score] (Shi et al. 2022). Detailed information on the assessment and definitions of these covariates is provided in the Supplemental Methods and Tables S3–S4.

### 2.5 Genetic Susceptibility Assessment

Detailed information on genotyping, imputation, and quality control in the UK Biobank has been previously reported (Bycroft et al. 2018). For this study, polygenic risk scores (PRS) were constructed to quantify genetic predisposition to five specific GI diseases: GERD, peptic ulcer, diverticular disease, NAFLD, and cirrhosis. Single‐nucleotide polymorphisms (SNPs) associated with these diseases were identified from published genome‐wide association studies (GWAS) (An et al. 2019; Emdin et al. 2021; Maguire et al. 2018; Vujkovic et al. 2022; Wu et al. 2021). The number of SNPs selected for each condition is provided in Table S5, along with corresponding effect sizes.

The PRS for each disease was calculated using a weighted method:

$$\mathrm{PRS}=\beta 1^{*}\text{SNP1}+\beta 2^{*}\text{SNP2}+\ldots +{\mathrm{\beta n}}^{*}\text{SNPn}$$

where β represents the effect size (log odds ratio) of each SNP derived from GWAS, and SNP denotes the number of risk alleles (0, 1, or 2) carried by the individual for that variant. The calculated PRS for each disease was normalized and categorized into “low genetic risk” and “high genetic risk” groups based on the median value within the study population. The inclusion of PRS in this analysis allowed for the evaluation of whether genetic predisposition modified the association between coffee consumption and gastrointestinal disease outcomes.

### 2.6 Statistical Analysis

Missing covariate data were addressed using multiple imputations by chained equations, incorporating all variables into the imputation model (Table S6). Baseline characteristics were summarized as means with standard deviations (SDs) for continuous variables and as counts with percentages for categorical variables. Cox proportional hazards regression models were applied to estimate hazard ratios (HRs) and 95% confidence intervals (CIs) for the association between coffee consumption and GI disease risk. Dose–response relationships were explored using restricted cubic spline regression. Subgroup and sensitivity analyses were conducted to ensure the robustness and consistency of findings across different population groups and analytical scenarios. Detailed statistical methods and analyses are provided in the Supplemental Methods.

## 3 Result

### 3.1 Participants and Characteristics

Baseline characteristics of the study population, stratified by coffee consumption patterns, are summarized in Table 1. The analysis included 147,263 participants, among whom 24.1% reported no coffee consumption, 56.0% consumed unsweetened coffee, 14.0% consumed coffee with natural sugar, and 5.8% consumed coffee with artificial sweeteners. The cohort was predominantly of White European ancestry (95.5%), with a mean age of 55.4 years (SD: 8.0). Instant coffee was the most consumed type, followed by filtered coffee. Participants who added natural sugar to their coffee used an average of 1.1 teaspoons (SD: 0.6) per cup, while those who used artificial sweeteners added an average of 1.4 teaspoons (SD: 0.6). Significant variations in demographic, lifestyle, and health‐related characteristics were observed across the coffee consumption groups. Participants consuming coffee with natural sugar were more likely to be male (61.2%), have lower educational attainment (63.9% without a college or university degree), and report smoking (14.0%). Additionally, they were more likely to exhibit poorer sleep quality (41.7% without a healthy sleep pattern), higher energy intake, and greater socioeconomic deprivation as reflected by higher Townsend deprivation index scores. In contrast, those consuming coffee with artificial sweeteners demonstrated a higher prevalence of obesity (31.7%), increased NSAID use (45.4%), increased PPI use (5.6%), and a greater burden of long‐term illnesses (18.7%) compared to other groups.

**TABLE 1:** Basic characteristics of participants.

Characteristic	Total	Non‐consumers	Coffee consumers		
			Unsweetened	Sugar‐sweetened	Artificially sweetened
Participants, *n* (%)	147,263 (100)	35,528 (24.1)	82,477 (56.0)	20,679 (14.0)	8579 (5.8)
Mean age (SD), years	55.4 (8.0)	53.9 (8.0)	55.8 (7.8)	55.6 (8.3)	57.0 (7.8)
Male, *n* (%)	67,243 (45.7)	15,240 (42.9)	35,389 (42.9)	12,660 (61.2)	3954 (46.1)
BMI (kg/m^2^), *n* (%)					
< 25	57,692 (39.2)	13,795 (38.9)	33,449 (40.6)	8453 (40.9)	1995 (23.3)
≥ 25 & < 30	60,850 (41.4)	14,234 (40.1)	33,795 (41.0)	8961 (43.4)	3860 (45.1)
≥ 30	28,537 (19.4)	7438 (21.0)	15,156 (18.4)	3231 (15.7)	2712 (31.7)
Ethnicity, *n* (%)					
White	140,339 (95.5)	32,341 (91.3)	80,439 (97.7)	19,317 (93.7)	8242 (96.3)
Other	6568 (4.5)	3089 (8.7)	1857 (2.3)	1306 (6.3)	316 (3.7)
Mean Townsend deprivation index (SD)	−1.6 (2.9)	−1.3 (3.0)	−1.8 (2.7)	−1.4 (3.0)	−1.6 (2.9)
Current employment status, *n* (%)					
Work	94,469 (64.2)	24,183 (68.2)	52,626 (63.9)	12,898 (62.5)	4762 (55.6)
Retired	42,126 (28.6)	8091 (22.8)	24,692 (30.0)	6170 (29.9)	3173 (37.0)
Other	10,457 (7.1)	3182 (9.0)	5069 (6.2)	1571 (7.6)	635 (7.4)
Education					
Degree	65,327 (44.5)	13,709 (38.7)	41,480 (50.4)	7433 (36.1)	3954 (46.1)
No degree	81,523 (55.5)	21,684 (61.3)	40,831 (49.6)	13,163 (63.9)	4625 (53.9)
Smoking status, *n* (%)					
Never	86,640 (58.9)	22,422 (63.2)	49,613 (60.2)	10,786 (52.2)	3819 (44.6)
Former	49,465 (33.6)	10,728 (30.2)	27,910 (33.9)	6989 (33.8)	3838 (44.8)
Current	11,028 (7.5)	2347 (6.6)	4884 (5.9)	2886 (14.0)	911 (10.6)
Mean pack‐years of smoking for current or former smokers (SD)	19.8 (16.4)	20.5 (17.1)	17.9 (151.)	22.2 (16.8)	24.4 (19.0)
Physical activity level, *n* (%)					
Low	22,719 (17.8)	5832 (19.2)	12,147 (16.9)	3237 (18.3)	1503 (20.5)
Moderate	53,933 (42.4)	12,477 (41.1)	31,175 (43.4)	7251 (40.9)	3030 (41.4)
High	50,673 (39.8)	12,079 (39.7)	28,577 (39.7)	7232 (40.8)	2785 (38.1)
Healthy sleep pattern, *n* (%)^a^	77,745 (62.2)	18,498 (61.6)	44,972 (64.1)	10,143 (58.3)	4132 (56.1)
Vitamin use, *n* (%)	46,614 (31.7)	11,555 (32.6)	25,883 (31.4)	6132 (29.7)	3044 (35.5)
Minerals and other dietary supplements use, *n* (%)	62,987 (42.8)	14,585 (41.1)	36,219 (43.9)	8193 (39.7)	3990 (46.5)
NSAIDS use, *n* (%)	51,578 (35.2)	12,719 (36.1)	27,562 (33.6)	7431 (36.2)	3866 (45.4)
PPI, *n* (%)	4800 (3.3)	1232 (3.5)	2385 (2.9)	700 (3.4)	483 (5.6)
Mean INFLA (SD), years	−0.8 (6.0)	−0.5 (6.1)	−1.2 (5.9)	−0.4 (6.0)	0.1 (6.0)
Family history of CVD, *n* (%)	82,185 (56.3)	19,382 (55.0)	46,659 (57.0)	11,039 (53.9)	5105 (60.1)
Family history of cancer, *n* (%)	50,299 (34.4)	11,714 (33.2)	28,615 (35.0)	7020 (34.3)	2950 (34.7)
Number of long‐term conditions, *n* (%)					
None	52,792 (35.8)	12,521 (35.2)	30,491 (37.0)	7564 (36.6)	2216 (25.8)
One	50,358 (34.2)	11,963 (33.7)	28,507 (34.6)	7129 (34.5)	2759 (32.2)
Two	27,948 (19.0)	6737 (19.0)	15,279 (18.5)	3928 (19.0)	2004 (23.4)
Three and more	16,165 (11.0)	4307 (12.1)	8200 (9.9)	2058 (10.0)	1600 (18.7)
Mean energy intake (SD), kcal/day	2084.6 (581.1)	2032.4 (616.4)	2075.2 (552.0)	2225.3 (605.8)	2051.3 (590.2)
Mean total sugar intake (SD), g/day	118.3 (47.1)	117.4 (50.4)	115.0 (43.9)	135.2 (49.87)	113.6 (47.1)
Mean tea intake (SD), drinks/day	2.9 (2.0)	3.9 (2.1)	2.7 (1.9)	2.5 (1.9)	2.2 (1.9)
Mean coffee intake on average in the past year (SD), drinks/day	1.9 (1.9)	0.3 (0.9)	2.5 (1.9)	2.2 (1.9)	2.7 (2.1)
Hot drink temperature, *n* (%)					
Very hot	24,387 (16.6)	5990 (16.9)	13,718 (16.6)	3234 (15.6)	1445 (16.8)
Hot	98,634 (67.0)	22,225 (62.6)	56,317 (68.3)	14,309 (69.2)	5783 (67.4)
Warm	22,640 (15.4)	5863 (16.5)	12,336 (15.0)	3101 (15.0)	1340 (15.6)
Other	1591 (1.1)	1444 (4.1)	105 (0.1)	31 (0.1)	11 (0.1)
Mean coffee intake (SD), drinks/day^c^					
Total coffee	2.0 (1.6)	0.0 (0.0)	2.6 (1.4)	2.3 (1.3)	2.7 (1.5)
Instant coffee	1.2 (1.4)	0.0 (0.0)	1.5 (1.4)	1.6 (1.4)	2.0 (1.5)
Filtered coffee	0.5 (0.9)	0.0 (0.0)	0.7 (1.0)	0.4 (0.8)	0.4 (0.8)
Cappuccino coffee	0.1 (0.4)	0.0 (0.0)	0.1 (0.4)	0.1 (0.4)	0.1 (0.4)
Latte coffee	0.1 (0.4)	0.0 (0.0)	0.1 (0.4)	0.1 (0.4)	0.1 (0.4)
Espresso coffee	0.0 (0.3)	0.0 (0.0)	0.1 (0.3)	0.1 (0.3)	0.0 (0.3)
Other coffee	0.0 (0.2)	0.0 (0.0)	0.0 (0.2)	0.0 (0.2)	0.0 (0.2)
Decaffeinated coffee, *n* (%)					
Yes	16,847 (11.4)	0 (0.0)	12,560 (15.2)	2610 (12.6)	1677 (19.5)
No	121,170 (82.3)	35,528 (100.0%)	62,300 (75.5)	17,133 (82.9)	6209 (72.4)
Varied	9246 (6.3)	0 (0.0)	7617 (9.2)	936 (4.5)	693 (8.1)
Type of sweetener added to coffee, teaspoons/day					
Sugar	0.2 (0.4)	—	0.0 (0.0)	1.1 (0.6)	—
Artificial sweetener	0.1 (0.4)	—	0.0 (0.0)	—	1.4 (0.6)
Mean Alternative healthy eating index (AHEI, SD)^b^	38.3 (13.0)	37.4 (13.3)	39.6 (12.8)	34.8 (12.5)	37.2 (12.4)
Mean completed 24‐h dietary recalls (SD), *n*					
1	60,102 (40.8)	18,221 (51.3)	29,118 (35.3)	8960 (43.3)	3803 (44.3)
2	33,790 (22.9)	7502 (21.1)	19,557 (23.7)	4708 (22.8)	2023 (23.6)
3	29,087 (19.8)	5592 (15.7)	18,097 (21.9)	3862 (18.7)	1536 (17.9)
4	20,540 (13.9)	3606 (10.1)	13,213 (16.0)	2648 (12.8)	1073 (12.5)
5	3744 (2.5)	607 (1.7)	2492 (3.0)	501 (2.4)	144 (1.7)

Abbreviations: AHEI, Alternative Healthy Eating Index; BMI, body mass index; CVD, cardiovascular disease; IQR, interquartile range; NSAIDs, nonsteroidal anti‐inflammatory drugs; PPI, proton pump inhibitors; SD, standard deviation.

^a^ Healthy sleep pattern: healthy sleep score ≥ 4; healthy sleep score: Range 0–5 points; positively connected with the degree of adherence to a healthy sleep pattern.

^b^ AHEI: The modified Alternative Healthy Eating Index is a combination of nine dietary indicators, including vegetables, fruit, whole grains, sugar‐sweetened beverages and fruit juice, nuts and legumes, red or processed meat, Long‐chain (*n* − 3) fats (EPA + DHA), PUFA, % of energy, and alcohol (Table). The scores were summed up, producing an overall score ranging from 0–90.

^c^ One drink was standardized to approximately 250 mL, with the questionnaire providing guidance on typical drink sizes (e.g., mug or cup) to avoid confusion.

### 3.2 Associations Between Coffee Consumption Patterns and Risk of Gastrointestinal Disease

**FIGURE 1:**
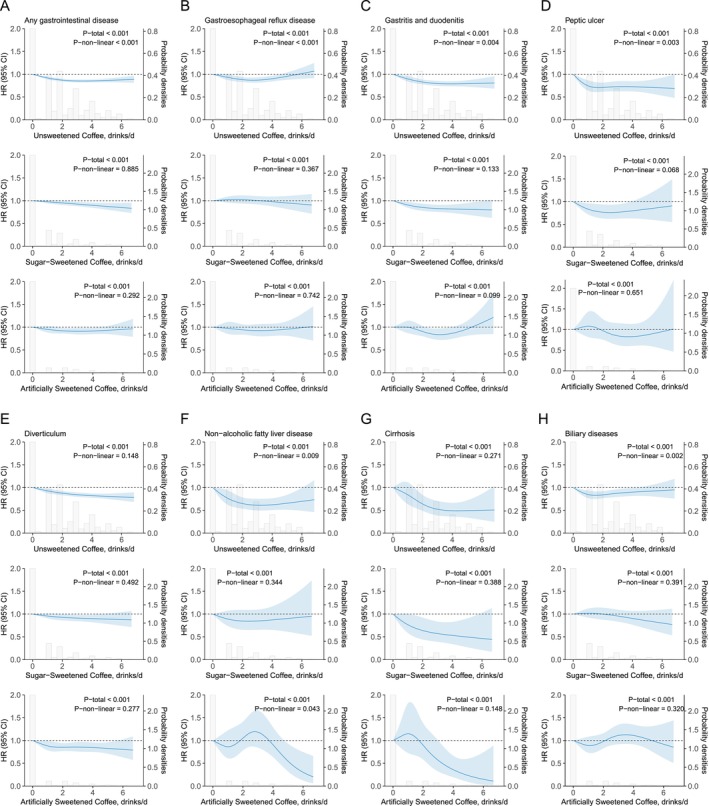
Dose–response associations of coffee consumption with incident. Any gastrointestinal disease (A), gastroesophageal reflux disease (B), gastritis and duodenitis (C), peptic ulcer (D), diverticulum (E), non‐alcoholic fatty liver disease (F), cirrhosis (G), and biliary diseases (H). Multivariable Cox regression model with restricted cubic splines adjusted for age (continuous), sex (male or female), body mass index (< 25, ≥ 25 & < 30, and ≥ 30 km/m^2^), ethnicity (white or other), Townsend deprivation index (continuous), current employment status (work, retired or other), education level (degree or no degree), smoking status (current, former, or never), pack‐years of smoking (continuous), physical activity level (low, moderate, or high), healthy sleep pattern (yes or no), hot drink temperature (very hot, hot, warm, or other), vitamin use (yes or no), mineral and other dietary supplements use (yes or no), NSAIDs use (yes or no), PPI use (yes or no), INFLA‐score (continuous), family history of CVD disease (yes or no), family history of cancer (yes or no), number of long‐term conditions (none, one, two, three and more), and intake of total energy, total sugar, tea, and AHEI score. AHEI, Alternative Healthy Eating Index; CVD, cardiovascular disease; INFLA‐score, Immune biomarkers and an aggregated inflammation‐score; NSAIDs, Nonsteroidal anti‐inflammatory drugs; PPI, Proton pump inhibitor.

**FIGURE 2:**
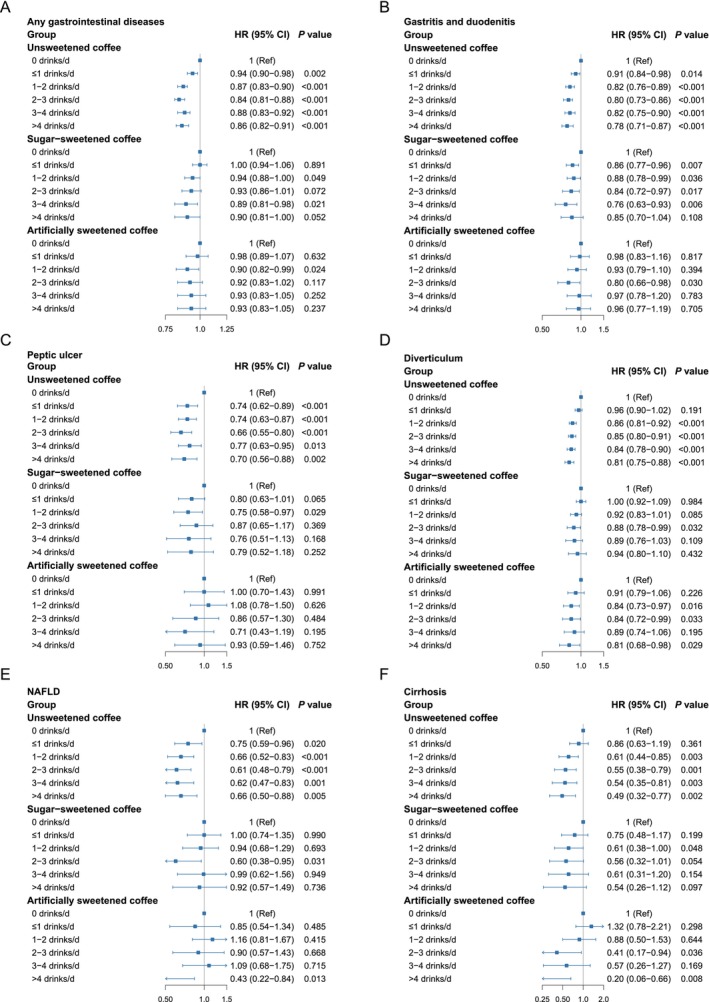
Association of coffee intake and risk of any gastrointestinal disease (A), gastritis and duodenitis (B), peptic ulcer (C), diverticulum (D), NAFLD (E), and cirrhosis (F). Multivariable Cox regression model with restricted cubic splines adjusted for age (continuous), sex (male or female), body mass index (< 25, ≥ 25 & < 30, and ≥ 30 km/m^2^), ethnicity (white or other), Townsend deprivation index (continuous), current employment status (work, retired or other), education level (degree or no degree), smoking status (current, former, or never), pack‐years of smoking (continuous), physical activity level (low, moderate, or high), healthy sleep pattern (yes or no), hot drink temperature (very hot, hot, warm, or other), vitamin use (yes or no), mineral and other dietary supplements use (yes or no), NSAIDs use (yes or no), PPI use (yes or no), INFLA‐score (continuous), family history of CVD disease (yes or no), family history of cancer (yes or no), number of long‐term conditions (none, one, two, three and more), and intake of total energy, total sugar, tea, and AHEI score. AHEI, Alternative Healthy Eating Index; CI, confidence interval; CVD, cardiovascular disease; HR, hazard ratio; NAFLD, Non‐alcoholic fatty liver disease; NSAIDs, Nonsteroidal anti‐inflammatory drugs; INFLA‐score, Immune biomarkers and an aggregated inflammation‐score; PPI, Proton pump inhibitor.

During a median follow‐up of 12.6 years (IQR: 11.2–13.5 years; total person‐years: 1,720,966), a total of 29,118 incident GI disease cases were identified. The association between coffee consumption and GI disease varied depending on the presence and type of sweetener added. Penalized spline analyses revealed a significant U‐shaped relationship between unsweetened coffee consumption and GI disease risk (P for non‐linearity < 0.001; Figure 1A). This pattern suggested the lowest risk among moderate consumers. In contrast, the association was not statistically significant for coffee with natural or artificial sweeteners. In fully adjusted Cox proportional hazards models, unsweetened coffee consumption was consistently associated with a lower risk of GI disease across various intake levels. Compared with non‐coffee drinkers, the HRs ranged from 0.94 (95% CI: 0.90–0.98) for those consuming 1 cup/day to 0.84 (95% CI: 0.81–0.88) for individuals consuming 2–3 cups/day. For coffee with natural sugar, a modest protective association was observed, though attenuated compared to unsweetened coffee. The lowest risk was noted among those consuming 3–4 cups/day, with an HR of 0.89 (95% CI: 0.81–0.98). In contrast, the relationship between artificially sweetened coffee and GI disease risk was inconsistent. HRs frequently included 1.0, indicating a lack of clear statistical significance (Figure 2A, Table S7).

### 3.3 Associations Between Coffee Intake and Subtypes of Gastrointestinal Diseases

This study analyzed associations between coffee consumption and 15 subtypes of GI diseases. Among the 29,118 recorded cases, the most frequent diagnosis was diverticular disease (12,522 cases, 43.0%), followed by GERD (8792 cases, 30.2%), gastritis and duodenitis (8258 cases, 28.3%), and biliary diseases (4073 cases, 14.0%) (Figure S4). In fully adjusted Cox proportional hazards models, unsweetened coffee consumption was significantly associated with lower risks of seven GI disease subtypes: GERD, gastritis and duodenitis, peptic ulcer, diverticular disease, NAFLD, cirrhosis, and biliary diseases (Figure 2B–F, Table S7, Figures S5, S6). Restricted cubic spline models revealed significant U‐shaped dose–response relationships for five of these subtypes (excluding diverticular disease and cirrhosis), consistent with the overall risk pattern for GI diseases (Figure 1). For coffee with natural sugar, a reduced risk was observed only for gastritis and duodenitis, with a linear dose–response relationship (P for non‐linearity = 0.133; Figure 1C). In contrast, coffee with artificial sweeteners was associated with a lower risk of diverticular disease, also following a linear pattern (P for non‐linearity = 0.277; Figure 1E).

### 3.4 Combined Effect and Interactions of Coffee Consumption and Genetic Risk on Gastrointestinal Diseases Risk

**FIGURE 3:**
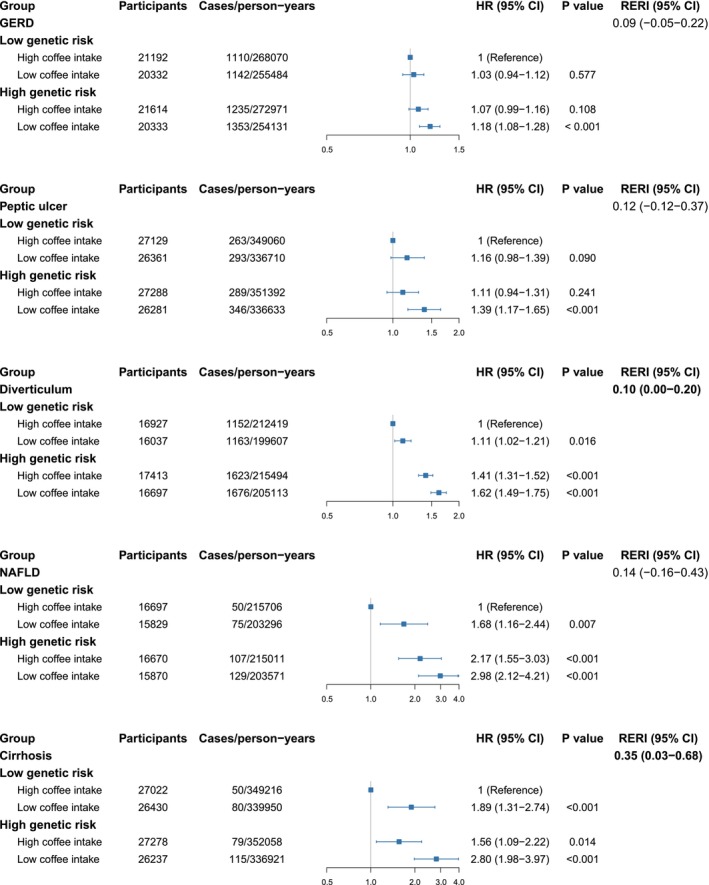
Joint associations of unsweetened coffee consumption and PRS on GERD, peptic ulcer, diverticulum, NAFLD, and cirrhosis risk. Multivariable model, estimates are hazard ratios (95% CIs) from multivariable Cox proportional hazard models adjusted for age (continuous), gender, body mass index (< 25, ≥ 25 & < 30, and ≥ 30 kg/m^2^), ethnicity (white or other), Townsend deprivation index (continuous), Current employment status (work, retired, or other), education level (degree or no degree), smoking status (current, former, or never), pack‐years of smoking (continuous), physical activity level (low, moderate, or high), healthy sleep pattern (yes or no), hot drink temperature (very hot, hot, warm, or other), vitamin use (yes or no), mineral and other dietary supplements use (yes or no), NSAIDs use (yes or no), PPI use (yes or no), INFLA‐score (continuous), family history of CVD disease (yes or no), family history of cancer (yes or no), number of long‐term conditions (none, one, two, three and more), and intake of total energy, total sugar, total tea, AHEI score, genotyping batch, and the first 10 genetic principal components. AHEI, Alternative Healthy Eating Index; CI, confidence interval; CVD, cardiovascular disease; GERD, Gastroesophageal reflux disease; HR, hazard ratio; INFLA‐score, Immune biomarkers and an aggregated inflammation‐score; NAFLD, Non‐alcoholic fatty liver disease; NSAIDs, Nonsteroidal anti‐inflammatory drugs; PPI, Proton pump inhibitor; PRS, polygenic risk score.

The study assessed PRS for five GI diseases: GERD, peptic ulcer, diverticular disease, NAFLD, and cirrhosis. Higher PRS values were consistently associated with increased risks for these diseases across all coffee consumption groups (Table S8). Even after adjusting for PRS, unsweetened coffee consumption was independently associated with reduced risks for all five diseases, supporting its potential protective role (Table S9). In contrast, coffee with natural or artificial sweeteners showed no significant associations with these diseases, in line with previous findings. Further analysis of the combined effects of genetic risk and coffee consumption revealed notable additive interactions for two conditions: diverticular disease and cirrhosis. Specifically, significant relative excess risks due to interaction (RERI) were observed for diverticular disease (RERI = 0.10, 95% CI: 0.00–0.20) and cirrhosis (RERI = 0.35, 95% CI: 0.03–0.68) (Figure 3). Participants with low unsweetened coffee consumption and high genetic risk exhibited substantially elevated risks for these conditions compared to those with high coffee intake and low genetic risk. The HRs for this high‐risk group were 1.62 (95% CI: 1.49–1.75, *p* < 0.001) for diverticular disease and 2.80 (95% CI: 1.98–3.97, *p* < 0.001) for cirrhosis. In contrast, multiplicative interaction analyses did not identify statistically significant interactions between genetic risk and unsweetened coffee intake for any of the five GI disease subtypes (Figures S7–S11).

### 3.5 Subgroup and Sensitivity Analyses

The results of our subgroup analyses, as shown in Figures S12–S22, revealed no significant effect modification for most outcomes. Across the majority of subgroups, the associations between coffee consumption and the risk of GI diseases remained consistent. However, notable interactions were observed for specific factors: sex (P for interaction = 0.032 for unsweetened coffee and overall GI disease), BMI (P for interaction = 0.038 for unsweetened coffee and GI disease), smoking status (P for interaction = 0.035 for coffee with natural sugar and GI disease), and the number of long‐term conditions (P for interaction = 0.012 for unsweetened coffee and GI disease). For other variables, including age, dietary quality, and socioeconomic status, no significant interactions were detected, suggesting that coffee consumption had broadly similar effects across these groups. To assess the robustness of our findings, several sensitivity analyses were conducted, with results detailed in Tables S10–S19. Overall, the results remained consistent with the primary analyses. Applying Fine and Gray's competing risk model or excluding participants who developed GI diseases within the first 2 years of follow‐up slightly attenuated the associations. In contrast, excluding participants with atypical dietary patterns strengthened the inverse associations between unsweetened coffee intake and GI disease risk.

## 4 Discussion

In this large prospective cohort study, we found that unsweetened coffee consumption was consistently associated with a reduced risk of GI diseases, following a U‐shaped dose–response relationship. The strongest protective effects were observed at moderate intake levels, particularly for subtypes such as GERD, NAFLD, and biliary diseases. In contrast, coffee with natural sweeteners demonstrated a weaker protective association, limited to gastritis and duodenitis, while coffee with artificial sweeteners showed no significant relationship with GI diseases risk. Furthermore, genetic predisposition to certain GI diseases modified the protective effects of unsweetened coffee, with significant additive interactions observed for diverticulosis and cirrhosis. These findings remained robust across subgroup and sensitivity analyses, emphasizing the potential role of coffee consumption, particularly unsweetened coffee, in reducing the burden of GI diseases.

### 4.1 Comparison With Previous Studies

Our study provides important insights into the distinct effects of unsweetened and sweetened coffee consumption on GI diseases, findings that partially align with and extend previous research. A key contribution of our work is the differentiation between unsweetened and sweetened coffee, which has often been overlooked in earlier studies that typically considered all coffee consumption as a single category (Poole et al. 2017). This approach has the potential to introduce confounding due to added sugar, which could obscure the true effects of coffee itself on GI health (Grosso et al. 2017). Previous studies, such as those from the US NIH‐AARP Diet and Health study, have shown that adding sugar or sweeteners to coffee can negatively impact overall health outcomes, including an increased risk of depression (Guo et al. 2014). Similarly, a Korean study found that sugar‐sweetened coffee was associated with a higher likelihood of developing metabolic syndrome (Kim, Cho, et al. 2014). However, despite the established negative impact of sugar on health, direct evidence linking sweetened coffee consumption to GI diseases risk remains limited, and our study is one of the few to examine this relationship directly.

In our study, the association between coffee consumption and the risk of GI diseases was influenced by the type of sweetener added. Unsweetened coffee consumption showed a significant U‐shaped dose–response relationship with GI disease risk, with moderate consumption (2–3 cups/day) being associated with the lowest risk. This finding aligns with previous research indicating that moderate coffee intake may have protective effects against certain GI conditions (Nehlig 2022). In contrast, coffee with natural or artificial sweeteners did not show statistically significant associations, suggesting that the type of sweetener added to coffee may influence its effects on GI health. The variability in the associations between coffee with different types of sweeteners and GI diseases underscores the complexity of coffee's effects, which is further highlighted by the heterogeneous outcomes observed in the existing literature. Our findings indicated that moderate consumption of unsweetened coffee (1–3 cups per day) was associated with a reduced risk of GERD, while other dosage levels had no significant effect. This contrasts with previous studies reporting mixed results: while some suggested low coffee consumption has minimal influence, others highlighted potential risks at higher intake levels (Y. Chen et al. 2022; Kim, Oh, et al. 2014). Notably, research on BE, a condition closely linked to GERD, also yielded conflicting findings, with some studies identifying no association and others reporting increased risks among former or long‐term coffee drinkers (Sajja et al. 2016). Regarding IBS and IBD, our findings showed no association between unsweetened coffee consumption and the risk of developing IBS or IBD. This contrasts with several studies suggesting that coffee drinkers may have a reduced likelihood of developing these conditions (Koochakpoor et al. 2021; Lee et al. 2023). However, other studies have indicated that caffeine may exacerbate GI symptoms, such as diarrhea, in IBS/IBD patients (Clevers et al. 2024). For peptic ulcers, our study found that unsweetened coffee consumption was linked to a lower risk, whereas other research, including a large meta‐analysis, found no significant association between coffee and peptic ulcers (Kurata and Nogawa 1997; Shimamoto et al. 2013). Similar inconsistencies exist in studies on biliary diseases, with some reporting a protective effect of coffee, especially in women, while others show no effect (Ruhl and Everhart 2000; Walcher et al. 2010). In the case of GI cancers, our findings revealed no significant association between unsweetened coffee consumption and cancer risk, consistent with the IARC's conclusion that coffee is “not classifiable as to its carcinogenicity to humans” (Humans 2018). However, earlier research has reported mixed results, with some studies suggesting potential protective effects for specific cancers, such as pancreatic cancer (Ran et al. 2016), and others finding no significant associations (Zhou et al. 2019).

The inconsistency in findings may be attributed to various factors, including individual differences in metabolism, the presence of underlying GI disorders, and the way in which coffee is consumed (e.g., with or without food) (Thomas and Hodges 2020). Moreover, most studies to date have been cross‐sectional, which limits the ability to draw causal conclusions about the long‐term effects of coffee on GI diseases. Importantly, our study emphasizes the need for further research to disentangle the effects of different coffee types and preparation methods on GI health. Prospective studies that account for potential confounders, such as lifestyle factors, pre‐existing conditions, and the presence of sweeteners, will be essential for providing more definitive insights. Additionally, exploring the mechanisms underlying the protective effects of coffee—particularly in relation to its bioactive compounds—could offer valuable information to inform future dietary recommendations.

### 4.2 Mechanistic Insights

The protective effects of coffee consumption, particularly unsweetened coffee, on GI health are likely mediated through a variety of interconnected mechanisms. Caffeine, a primary bioactive compound in coffee, has been shown to stimulate gastric acid secretion and bile production, both of which promote digestion and contribute to overall gut health (Kidd et al. 2009; Nehlig 2022). Additionally, coffee enhances colonic motility (Latthe et al. 2025), a critical factor in the development and progression of GI diseases such as diverticular disease (Rezapour et al. 2018). By improving digestive system function, coffee may help reduce the risk of conditions like GERD, gastritis, duodenitis, and peptic ulcers.

Another key mechanism involves the metabolic effects of caffeine. Caffeine promotes fat oxidation, increases metabolic rate, and enhances energy expenditure (Greenberg et al. 2006). These effects are particularly relevant in the context of obesity, a well‐established risk factor for numerous GI diseases (Emerenziani et al. 2019). By promoting weight loss and reducing fat accumulation, caffeine may help alleviate the inflammatory burden associated with conditions such as NAFLD, gastritis, and GERD (Lange et al. 2021; Yesil and Yilmaz 2013). Moreover, bioactive compounds like polyphenols and chlorogenic acid found in coffee also exhibit anti‐inflammatory properties and may provide additional protective benefits (Loftfield et al. 2015).

Coffee consumption has also been shown to influence gut microbiota, promoting beneficial bacterial populations such as Bifidobacteria, Bacteroides, and Prevotella (Mansour et al. 2020; Mills et al. 2015). These bacteria play an essential role in maintaining gut health by modulating immune responses, improving intestinal barrier function, and reducing gut inflammation (Chen et al. 2024; Pan et al. 2022; Takiishi et al. 2017; Wang et al. 2024). Interestingly, coffee containing natural or artificial sweeteners appears to show weaker or inconsistent associations with GI health, possibly due to the interference of sweeteners with coffee's bioactive compounds or their own metabolic effects. Consumption of added sweeteners may also lead to weight gain or an increase in total energy intake. Although calorie‐free, artificial sweeteners have been shown to alter gut microbiota and induce inflammatory responses, which may counteract the health benefits of coffee (Bueno‐Hernández et al. 2019). Furthermore, individuals with a higher BMI, particularly those with obesity, often exhibit altered gut microbiota composition and increased gut inflammation (Emerenziani et al. 2019), potentially diminishing the beneficial effects of coffee on GI health. This is consistent with the findings of our subgroup analysis, which revealed stronger protective effects of coffee in individuals with a lower BMI, suggesting that coffee may be more effective in those with less visceral fat.

Gender differences in caffeine metabolism may also influence coffee's protective effects. Females generally metabolize caffeine more slowly than males, resulting in prolonged exposure to caffeine's metabolic effects (Nehlig 2018). This could explain why the observed protective effects of coffee on GI health were more pronounced in females than in males. Additionally, genetic factors contribute significantly to how coffee affects GI disease risk. Our study found that individuals with a high genetic risk for conditions such as diverticular disease and cirrhosis appeared to benefit more from coffee consumption, particularly unsweetened coffee. The additive interaction between genetic risk and coffee consumption for conditions like diverticular disease and cirrhosis underscores the complexity of the relationship between genetics and diet.

These mechanisms underscore the multifactorial nature of coffee's impact on GI health, with factors such as metabolic rate, gut microbiota, obesity, gender, and genetic susceptibility all interacting to modulate the benefits of coffee consumption. While these insights provide a more nuanced understanding of coffee's role in GI disease prevention, further research is needed to better elucidate the molecular pathways involved, examine the variations between different types of coffee and preparation methods, and explore how individual genetic and metabolic factors influence coffee's effects. Ultimately, such research will enable the development of personalized recommendations for coffee consumption based on individual characteristics, optimizing its protective effects on GI health.

## 5 Strengths and Limitations

This study benefits from several key strengths that enhance the robustness of our findings. First, we utilized a large and diverse sample from the UK Biobank, which provides a high level of statistical power and allows for the generalization of results to the broader population. The prospective design of the study minimizes recall bias and supports a stronger causal inference compared to cross‐sectional studies. Additionally, the use of detailed dietary data, including information on both unsweetened and sweetened coffee consumption, enables a nuanced analysis of the differential effects of coffee on GI diseases. This distinction is critical, as previous research often did not separate these types of coffee consumption, potentially masking important differences in health outcomes. Moreover, the inclusion of genetic risk scores for GI diseases is a notable strength, as it allows us to investigate the interaction between genetic predisposition and coffee consumption.

Despite these strengths, several limitations warrant consideration. First, while we distinguished between coffee types, our study design could not directly investigate the potential molecular interactions between sweeteners and coffee's inherent bioactive constituents (e.g., polyphenols or caffeine). Experimental evidence suggests that sweeteners may alter the stability, absorption, or biological activity of these compounds (Agulló et al. 2021; Liu et al. 2022), meaning that additives may not merely contribute caloric load but could actively modulate the physiological effects of coffee. Although this represents a fascinating avenue for future mechanistic research, it remains a limitation of our observational approach. Second, our reliance on self‐reported dietary data, although comprehensive, is prone to measurement error and recall bias. Third, the number of participants consuming artificially sweetened coffee was considerably lower than those consuming unsweetened or sugar‐sweetened coffee, potentially limiting the statistical power for this group. Future studies with more detailed information on sweetener types and quantities would provide a clearer understanding of their role in modulating coffee's effects. Fourth, the observational nature of our study precludes definitive causal conclusions, as unmeasured confounders or reverse causality could still influence the observed associations. Furthermore, although our study included a broad spectrum of GI diseases, certain conditions—such as Crohn's disease, Celiac disease, and UC—had relatively few cases, which may limit the statistical power to detect associations for these specific conditions. Finally, while our analysis accounted for various subgroups, potential effect modifiers, such as long‐term dietary patterns or specific coffee brewing methods, were not fully explored.

## 6 Conclusions

In conclusion, this study systematically evaluated the associations between different types of coffee consumption and the risk of GI diseases. The results demonstrate that unsweetened coffee consumption is significantly associated with a lower risk of overall GI diseases and several specific subtypes, while coffee with natural sugar shows limited protective effects, and coffee with artificial sweeteners exhibits no significant associations. These findings provide important evidence for the potential benefits of unsweetened coffee as part of a healthy dietary pattern and highlight the need for future research to explore the underlying mechanisms and validate these results in diverse populations.

## Author Contributions

L.Z. and M.C. contributed to the study conception and design. F.H. contributed to data collection, assembly, and analysis. S.Z., H.H., D.L., and Z.L. contributed to the interpretation of the data. S.Z. contributed to the manuscript drafting. M.C., L.Z., and F.H. contributed to the manuscript revision. All authors read and approved the final manuscript.

## Funding

This work was supported by the Science and Technology Development Foundation of Shenzhen (JCYJ20190808111817192, JCYJ20210324093807020).

## Ethics Statement

The UK Biobank Cohort Study was approved by the Northwest Multi‐Centre Research Ethics Committee (21/NW/0157). All the participants provided written informed consent before participating in the study.

## Consent

Written informed consent was obtained from all study participants.

## Conflicts of Interest

The authors declare no conflicts of interest.

## Supporting information


**Data S1:** fsn371271‐sup‐0001‐TableS1‐S19‐FigureS1‐S2.docx.

## Data Availability

The data that support the findings of this study are available on request from the UK Biobank (www.ukbiobank.ac.uk/).
