# Supplementary material for: Coffee Consumption and Risk of Incident Gastrointestinal Disease: A Large Prospective Cohort Study
Source: Food Sci Nutr. 2025 Nov 27;13(12):e71271. doi: 10.1002/fsn3.71271 (PMC12658384; doi:10.1002/fsn3.71271)
Supplement: Supplementary file 1 — Data S1: fsn371271‐sup‐0001‐TableS1‐S19‐FigureS1‐S2.docx. [file FSN3-13-e71271-s001.docx]

**Supplementary information**

Coffee consumption and risk of incident gastrointestinal disease: A large prospective cohort study

Contents

[**Supplementary Methods 7**](#_Toc206342239)

[**Supplementary Table 1. Classification of coffee consumers by sweeteners. 11**](#_Toc206342240)

[**Supplementary Table 2. Definitions of disease in this study. 12**](#_Toc206342241)

[**Supplementary Table 3. Information about all variables involved in this study. 13**](#_Toc206342242)

[**Supplementary Table 4. Definition and list of long-term conditions in UK Biobank. 15**](#_Toc206342243)

[**Supplementary Table 5. Detail information of SNP for genetic risk score. 18**](#_Toc206342244)

[**Supplementary Table 6. Detailed information on missing covariates. 22**](#_Toc206342245)

[**Supplementary Table 7. Associations of coffee consumption with risk of Gastrointestinal disease incident. 23**](#_Toc206342246)

[**Supplementary Table 8. Association of polygenic risk score (PRS) with the risk of GERD, peptic ulcer, diverticulum, NAFLD, and cirrhosis. 33**](#_Toc206342247)

[**Supplementary Table 9. Association of coffee consumption with the incidence of GERD, peptic ulcer, diverticulum, NAFLD, and cirrhosis. 35**](#_Toc206342248)

[**Supplementary Table 10. Associations of coffee consumption with incident GI diseases after excluding participants with missing covariate values. 38**](#_Toc206342249)

[**Supplementary Table 11. Associations of coffee consumption with incident GI diseases using Fine and Gray's competing risk model 41**](#_Toc206342250)

[**Supplementary Table 12. Associations of coffee consumption with incident GI diseases after excluding participants who had an outcome event during the first two years of follow-up 44**](#_Toc206342251)

[**Supplementary Table 13. Associations of coffee consumption with incident GI diseases after excluding participants who had an outcome event during the first five years of follow-up 47**](#_Toc206342252)

[**Supplementary Table 14. Associations of coffee consumption with incident GI diseases after excluding participants who reported that their diet was not typical on any recall day 50**](#_Toc206342253)

[**Supplementary Table 15. Associations of coffee consumption with incident GI diseases after excluding participants who reported coffee consumption at baseline but were classified as non-consumers in their 24-hour dietary recalls. 53**](#_Toc206342254)

[**Supplementary Table 16. Associations of coffee consumption with incident GI diseases, based on their first completed 24-hour dietary recall. 56**](#_Toc206342255)

[**Supplementary Table 17. Associations of coffee consumption with incident GI diseases after excluding high-risk participants who simultaneously suffered from at least three chronic diseases. 59**](#_Toc206342256)

[**Supplementary Table 18. Associations of coffee consumption with incident GI diseases after additional adjustments to the intake of sugar-sweetened beverages, artificially sweetened beverages, natural juices, milk and water. 62**](#_Toc206342257)

[**Supplementary Table 19. Associations of coffee consumption with incident GI diseases after removing sugar added to coffee from total sugar and total energy. 65**](#_Toc206342258)

[**Supplementary Figure 1. Histogram of total coffee intake. The dashed blue line is the 99th percentile of coffee intake. 68**](#_Toc206342259)

[**Supplementary Figure 2. Flowchart of study design and participant enrollment. 69**](#_Toc206342260)

[**Supplementary Figure 3. Flow chart of coffee consumer classification. 70**](#_Toc206342261)

[**Supplementary Figure 4. An overview of case distribution among gastrointestinal disease subtypes. 71**](#_Toc206342262)

[**Supplementary Figure 5. Dose–response associations of coffee consumption with incident barrett’s oesophagus (A), celiac disease (B), crohn’s disease (C), ulcerative colitis (D), irritable bowel syndrome (E), pancreatitis (F), appendicitis (G), and gastrointestinal cancer (H). 72**](#_Toc206342263)

[**Supplementary Figure 6. Association of coffee intake and risk of GERD and biliary diseases. 74**](#_Toc206342264)

[**Supplementary Figure 7. Associations of unsweetened coffee consumption with incident GERD according to the polygenic risk score. 75**](#_Toc206342265)

[**Supplementary Figure 8. Associations of unsweetened coffee consumption with incident peptic ulcer according to the polygenic risk score. 76**](#_Toc206342266)

[**Supplementary Figure 9. Associations of unsweetened coffee consumption with incident diverticulum according to the polygenic risk score. 77**](#_Toc206342267)

[**Supplementary Figure 10. Associations of unsweetened coffee consumption with incident NAFLD according to the polygenic risk score. 78**](#_Toc206342268)

[**Supplementary Figure 11. Associations of unsweetened coffee consumption with incident cirrhosis according to the polygenic risk score. 79**](#_Toc206342269)

[**Supplementary Figure 12. Age-stratified analysis of the association between coffee consumption and incident GI disease in the multivariable model. 80**](#_Toc206342270)

[**Supplementary Figure 13. Sex-stratified analysis of the association between coffee consumption and incident GI disease in the multivariable model. 81**](#_Toc206342271)

[**Supplementary Figure 14. BMI-stratified analysis of the association between coffee consumption and incident GI disease in the multivariable model. 82**](#_Toc206342272)

[**Supplementary Figure 15. Employment-stratified analysis of the association between coffee consumption and incident GI disease in the multivariable model. 83**](#_Toc206342273)

[**Supplementary Figure 16. TDI-stratified analysis of the association between coffee consumption and incident GI disease in the multivariable model. 84**](#_Toc206342274)

[**Supplementary Figure 17. Smoking status-stratified analysis of the association between coffee consumption and incident GI disease in the multivariable model. 85**](#_Toc206342275)

[**Supplementary Figure 18. Physical activity level-stratified analysis of the association between coffee consumption and incident GI disease in the multivariable model. 86**](#_Toc206342276)

[**Supplementary Figure 19. Healthy sleep pattern-stratified analysis of the association between coffee consumption and incident GI disease in the multivariable model. 87**](#_Toc206342277)

[**Supplementary Figure 20. Hot drink temperature-stratified analysis of the association between coffee consumption and incident GI disease in the multivariable model. 88**](#_Toc206342278)

[**Supplementary Figure 21. AHEI score-stratified analysis of the association between coffee consumption and incident GI disease in the multivariable model. 89**](#_Toc206342279)

[**Supplementary Figure 22. Number of long-term conditions-stratified analysis of the association between coffee consumption and incident GI disease in the multivariable model. 90**](#_Toc206342280)

# Supplementary Methods

**Assessment of covariates**

Baseline data were collected through self-administered touchscreen questionnaires, physical measurements, and linked health records at recruitment. The covariates considered for inclusion in this study included age, sex, body mass index (BMI), basal metabolic rate, ethnicity, Townsend deprivation index (TDI), current employment status, education level, smoking habits, pack-years of smoking , physical activity, healthy sleep pattern, vitamin intake (A, B, C, D, E, folic acid, or multivitamins), mineral and other dietary supplement use (such as fish oil, glucosamine, calcium, zinc, iron, and selenium), use of nonsteroidal anti-inflammatory drugs (NSAIDs; aspirin, ibuprofen, and paracetamol), use of proton pump inhibitors (PPI; omeprazole, esomeprazole, pantoprazole, lansoprazole, aripiprazole, and rabeprazole sodium), Immune biomarkers and an aggregated inflammation-score (INFLA-score), family history of cardiovascular disease (CVD), family history of cancer, number of chronic conditions, and dietary intake of total energy, sugar, total tea, and alternative healthy eating index. BMI was determined by dividing an individual's weight in kilograms by the square of their height in meters, and participants were subsequently categorized into three groups: under 25, 25.0 to 29.9, and 30.0 or above kg/m^2. The TDI was utilized as a measure of socioeconomic status, with lower values signifying higher affluence. Participants' current employment status was categorized into three main groups: employed, retired, and others (including looking after home and/or family, unable to work because of sickness or disability, unemployed, doing unpaid or voluntary work, and full or part-time student). The education level is classified as degree and no degree (including A levels/AS levels or equivalent, O levels/GCSEs or equivalent, CSEs or equivalent, NVQ or HND or HNC or equivalent, and other professional qualifications). Pack-years, a metric for quantifying lifetime smoking exposure, was calculated by dividing the average number of cigarettes smoked per day by 20 and then multiplying the result by the total number of years of smoking. Physical activity levels were stratified into low, moderate, and high categories based on the responses obtained from the International Physical Activity Questionnaire (IPAQ), a standardized tool for assessing physical activity levels across various populations (Bull et al., 2020). Sleep patterns were assessed based on five specific sleep characteristics, with each trait being rated as either 1 for 'healthy' or 0 for 'unhealthy' (Fan et al., 2020). The total score, ranging from 0 to 5, was employed to categorize sleep patterns, with a score of 4 or above being indicative of a healthy sleep pattern.

Total sugar and energy intake were derived from UK Biobank dietary data using McCance and Widdowson's Composition of Foods. The AHEI score was based on nine components, including fruits, vegetables, whole grains, sugary drinks and fruit juice, nuts and legumes, red/processed meats, long-chain (n-3) fats (EPA + DHA), polyunsaturated fats (PUFA), and alcohol intake, with scores ranging from 0 (nonadherence) to 90 (perfect adherence) (Chiuve et al., 2012; Shang et al., 2023). The number of long-term chronic diseases, which are critical for assessing quality of life and longevity, was included as an adjustment factor. Here we included 34 major chronic conditions identified in a large study in Scotland (Table S4) (Barnett et al., 2012). Disease history was collected at baseline and defined based on self-reporting, physician diagnosis, or treatment with corresponding medications. The INFLA-score is a comprehensive metric reflecting low-grade inflammation, constituted by four validated biomarkers indicative of systemic inflammation: C-reactive protein (CRP), total white blood cell (WBC) count, platelet count, and the neutrophil-to-lymphocyte ratio (NLR). The scoring algorithm proceeds as follows: each of the four biomarkers—CRP, WBC, platelet count, and NLR—is assigned a value based on its distribution across the population. Specifically, individuals falling within the upper four deciles (7th to 10th percentiles) receive scores ranging from +1 to +4, while those in the lower four deciles (1st to 4th percentiles) are assigned scores from -4 to -1. The INFLA-score is calculated by aggregating these individual biomarker scores, yielding a cumulative measure of inflammation. A higher cumulative score signifies a more pronounced state of low-grade inflammation.

Participants who selected “do not know” or “prefer not to answer” were categorized as having missing values. Details of covariate definitions, Field IDs, and scoring criteria are presented in **Table S2-S4**. Additional information on these assessments is available on the UK Biobank website (www.ukbiobank.ac.uk).

**Statistical Analysis**

To address missing data, we employed multiple imputation by chained equations to generate five complete datasets. Subsequently, we pooled the results using Rubin's rules for a comprehensive analysis. Details regarding the extent and handling of missing covariates are provided in Table S6. Baseline characteristics of the study population were summarized as means with standard deviations (SD) for continuous variables and counts with percentages for categorical variables. The associations between coffee consumption and the risk of gastrointestinal diseases were analyzed using Cox proportional hazards regression models, providing hazard ratios (HRs) and 95% confidence intervals (CIs). Nonlinear dose-response relationships were explored using restricted cubic spline regression with knots positioned at the 5th, 35th, 65th, and 90th percentiles of coffee consumption. Coffee intake was categorized into predefined groups based on daily consumption levels, divided into six categories: 0 drinks/day (non-consumers), >0 to 1 drink/day, >1 to 2 drinks/day, >2 to 3 drinks/day, >3 to 4 drinks/day, and >4 drinks/day.

The proportional hazards assumption was evaluated using Kaplan–Meier estimates, and all tests met the predetermined criteria. The basic model adjusted for age (continuous) ,sex (male or female) and ethnicity (White or other), and the multivariable model further adjusted for BMI (≥30, 25–30, <25 kg/m^2^), basal metabolic rate (continuous), TDI (continuous), current employment status (work, retired or other), education level (degree or no degree), smoking status (never, former, or current), and pack-years of smoking (continuous), physical activity level (low, moderate, or high), healthy sleep pattern (yes or no), vitamin use (yes or no), mineral and other dietary supplement use (yes or no), NSAID use (yes or no), PPI (yes or no), INFLA-score (continuous), family history of CVD (yes or no), family history of cancer (yes or no), number of long-term conditions (none, one, two, three and more), and intake of total energy (including sugar added to coffee), total sugar (including sugar added to coffee), tea, and AHEI score.

Subgroup analyses were conducted to examine potential effect modifications by various factors, including age, sex, BMI, smoking status, physical activity, dietary quality (AHEI), TDI, employment, healthy sleep pattern, hot drink temperature and number of long-term conditions. Interaction terms were included in the regression models to assess multiplicative interactions, and additive interactions were evaluated using the relative excess risk due to interaction (RERI). Statistical significance of additive interactions was determined by the exclusion of zero in the 95% confidence intervals of RERI(Li & Chambless, 2007).

Sensitivity analyses were undertaken to assess the robustness of the findings. These included: 1) Excluding participants with missing covariate data; 2) Excluding participants who developed outcomes within the first two or five years of follow-up; 3) Applying Fine and Gray's competing risk model to account for competing mortality risks; 4) Excluding any participants who reported atypical dietary intake in any 24-hour dietary recall; 5) Excluding participants who reported coffee consumption at baseline but are classified as non-consumers in the 24-hour dietary recall; 6) Using data from the participants' first 24-hour dietary recall to evaluate coffee intake; 7) Excluding participants who simultaneously suffer from at least three chronic diseases; 8) Adjust the intake of sugar-sweetened beverages, artificially sweetened beverages, natural juices, milk and water additionally; 9) removing the sugar added to the coffee from the total sugar, and removing the energy produced from the total energy.

All statistical analyses were conducted using R software (version 4.2.3). A two-sided P-value of <0.05 was considered indicative of statistical significance.

**References**

Barnett, K., Mercer, S. W., Norbury, M., Watt, G., Wyke, S., & Guthrie, B. (2012). Epidemiology of multimorbidity and implications for health care, research, and medical education: a cross-sectional study. *Lancet, 380*(9836), 37-43. doi:10.1016/S0140-6736(12)60240-2

Bull, F. C., Al-Ansari, S. S., Biddle, S., Borodulin, K., Buman, M. P., Cardon, G., . . . Willumsen, J. F. (2020). World Health Organization 2020 guidelines on physical activity and sedentary behaviour. *Br J Sports Med, 54*(24), 1451-1462. doi:10.1136/bjsports-2020-102955

Chiuve, S. E., Fung, T. T., Rimm, E. B., Hu, F. B., McCullough, M. L., Wang, M., . . . Willett, W. C. (2012). Alternative dietary indices both strongly predict risk of chronic disease. *J Nutr, 142*(6), 1009-1018. doi:10.3945/jn.111.157222

Fan, M., Sun, D., Zhou, T., Heianza, Y., Lv, J., Li, L., & Qi, L. (2020). Sleep patterns, genetic susceptibility, and incident cardiovascular disease: a prospective study of 385 292 UK biobank participants. *Eur Heart J, 41*(11), 1182-1189. doi:10.1093/eurheartj/ehz849

Li, R., & Chambless, L. (2007). Test for additive interaction in proportional hazards models. *Ann Epidemiol, 17*(3), 227-236. doi:10.1016/j.annepidem.2006.10.009

Shang, X., Liu, J., Zhu, Z., Zhang, X., Huang, Y., Liu, S., . . . He, M. (2023). Healthy dietary patterns and the risk of individual chronic diseases in community-dwelling adults. *Nat Commun, 14*(1), 6704. doi:10.1038/s41467-023-42523-9

Zhang, L., Ma, C., Huang, H., Li, D., Zhang, D., Wu, T., . . . Hu, F. (2024). Association of unsweetened and sweetened cereal consumption with all-cause and cause-specific mortality: a large prospective population-based cohort study. *Food Funct, 15*(19), 10151-10162. doi:10.1039/d4fo03761h

# Supplementary Table 1. Classification of coffee consumers by sweeteners.

| **Categories of coffee consumers** | **n** | **%** |
| --- | --- | --- |
| Non-consumers | 36542 | 23.0% |
| Sole consumers |  |  |
| Unsweetened | 85757 | 54.0% |
| Sugar-sweetened | 21695 | 13.7% |
| Artificially sweetened | 9095 | 5.7% |
| Overlapped consumers of sweeteners |  |  |
| Unsweetened & Sugar-sweetened | 2445 | 1.5% |
| Unsweetened & Artificially sweetened | 652 | 0.4% |
| Sugar-sweetened & Artificially sweetened | 2447 | 1.5% |
| Unsweetened & Sugar-sweetened & Artificially sweetened | 111 | 0.0% |
| Total | 158744 | 100% |

Note: As shown in the workflow, a total of 502,411 individuals were recruited at baseline. We excluded participants who withdrew from UK Biobank (n = 42), participants who reported prevalent cancer at baseline (n = 41,791), participants who reported prevalent gastrointestinal diseases at baseline (n = 83,144), and participants who did not complete any of the 24-hour diet recalls (n = 215,878). Furthermore, we excluded participants who were lost during follow-up (n = 450) and participants whose diets were not credible (n = 2,362), leaving 158,744 participants with reliable coffee intake. We then further classified consumers based on whether additional sugar or artificial sweeteners were added to the coffee. In detail, the type and number of cross-consuming sweeteners are shown here.

# Supplementary Table 2. Definitions of disease in this study.

| **Prevalent diseases** | **Diagnosed by doctor (codes)** | **Self-reported (codes)** | **ICD10 (codes) *** |
| --- | --- | --- | --- |
| **Cancer** | 2453 (1) | 20001 (1001-1088, except 1060-1062, 1072, 99999) | C00-C97 (except C44) |
| **Gastrointestinal** **disease** |  |  |  |
| **Esophagus** | - |  |  |
| Barrett’s oesophagus | 1 | 20002 (1139) | K22.7 |
| GERD | 2 | 20002 (1138) | K21 |
| **Stomach and bowel** | - |  |  |
| Gastritis and duodenitis | 3 | 20002 (1143) | K29 |
| Peptic ulcer | 5 | 20002 (1400, 1142, 1457) | K25-K27 |
| Celiac disease | 4 | 20002 (1456) | K90.0 |
| Crohn’s disease (CD) | 6 | 20002 (1461, 1462) | K50 |
| Ulcerative colitis (UC) | 7 | 20002 (1461, 1463) | K51 |
| Irritable bowel syndrome (IBS) | 8 | 20002 (1154) | K58 |
| Diverticulum | 9 | 20002 (1458) | K57 |
| **Pancreas** | - |  |  |
| Pancreatitis (acute and chronic) | 10 | 20002 (1164, 1165) | K85, K86.0, K86.1 |
| **Liver** | - |  |  |
| Non-alcoholic fatty liver disease (NAFLD) | 11 | 20002 (1155, 1157) | K76.0 |
| Cirrhosis | 12 | 20002 (1141, 1158, 1604) | K74.0, K74.1, K74.2, K74.6, K70.2, K70.3, K70.4, K76.6, I85.0, I85.9 |
| **Gallbladder and biliary** | - |  |  |
| Biliary diseases (including cholangitis, cholecystitis, and cholelithiasis) | 13 | 20002 (1159, 1160, 1161, 1162, 1163, 1475) | K80, K81, K83.0 |
| **Appendix** | - |  |  |
| Appendicitis | 14 | 20002 (1502) | K35-K37 |
| **Gastrointestinal cancer** | - |  |  |
| Cancer (including oesophageal, gastric, small intestinal, colorectal, pancreatic cancer, gallbladder and biliary, liver cancers) | 15 | 20001 (1017, 1018, 1019, 1020, 1022, 1023, 1024, 1025, 1026) | C15-C20, C22-C25 |

Note: * Data was extracted from Field ID 41270/41280.

# Supplementary Table 3. Information about all variables involved in this study.

| **Variables** | **Field ID** |
| --- | --- |
| **Exposure** |  |
| Unsweetened coffee intake | Field ID 100240/100250/100270/100290/  100300/100310/100330/100370/100380 |
| Sugar-sweetened coffee intake |  |
| Artificially sweetened coffee intake |  |
| **Inclusion and exclusion criteria** |  |
| Date lost to follow-up | Field ID 191 |
| Cancer at baseline | Details are shown in Supplemental Table 2 |
| Barrett’s oesophagus at baseline | Details are shown in Supplemental Table 2 |
| GERD at baseline | Details are shown in Supplemental Table 2 |
| Gastritis and duodenitis at baseline | Details are shown in Supplemental Table 2 |
| Celiac disease at baseline | Details are shown in Supplemental Table 2 |
| Peptic ulcer at baseline | Details are shown in Supplemental Table 2 |
| Crohn’s disease (CD) at baseline | Details are shown in Supplemental Table 2 |
| Ulcerative colitis (UC) at baseline | Details are shown in Supplemental Table 2 |
| Irritable bowel syndrome (IBS) at baseline | Details are shown in Supplemental Table 2 |
| Diverticulum at baseline | Details are shown in Supplemental Table 2 |
| Pancreatitis at baseline | Details are shown in Supplemental Table 2 |
| Non-alcoholic fatty liver disease (NAFLD) at baseline | Details are shown in Supplemental Table 2 |
| Cirrhosis at baseline | Details are shown in Supplemental Table 2 |
| Biliary diseases at baseline | Details are shown in Supplemental Table 2 |
| Appendicitis at baseline | Details are shown in Supplemental Table 2 |
| 24-hour dietary recall | Field ID 105010/105030/20077 |
| Daily dietary data not credible | Field ID 100026 |
| **Covariates** |  |
| Age | Field ID 21003 |
| SEX | Field ID 21003 |
| BMI | Field ID 21001 |
| Basal metabolic rate | Field ID 23105 |
| Ethnicity | Field ID 21000 |
| Townsend deprivation index | Field ID 189 |
| Current employment status | Field ID 6142 |
| Education level | Field ID 6138 |
| Smoking status | Field ID 20116 |
| Pack years of smoking | Field ID 20161 |
| Physical activity | Field ID 22032 |
| Healthy sleep pattern | Details can be found in previous study (Zhang et al., 2024) |
| Energy | Field ID 100002 |
| Total sugar | Field ID 100008 |
| Total tea | Field ID 100400/100410/  100420/100430/100440 |
| AHEI score | Details can be found in previous study (Zhang et al., 2024) |
| Vitamin and mineral supplements | Field ID 6155 |
| Mineral and other dietary supplements | Field ID 6179 |
| NSAIDs use | Field ID 6154 |
| Proton pump inhibitors (PPI) use | Field ID 6154/20003 |
| Family history of CVD | Field ID 20107/20110/20111 |
| Family history of cancer | Field ID 20107/20110/20111 |
| Number of long-term chronic conditions | Details are shown in **Supplemental Table 4** |
| INFLA-score | Field ID 30710/30000/30080/30140/30120 |
| **Outcomes** |  |
| Barrett’s oesophagus |  |
| GERD |  |
| Gastritis and duodenitis |  |
| Celiac disease |  |
| Peptic ulcer |  |
| CD |  |
| UC | Details are shown in **Supplemental Table 2** |
| IBS |  |
| Diverticulum |  |
| Pancreatitis |  |
| NAFLD |  |
| Cirrhosis |  |
| Biliary diseases |  |
| Appendicitis |  |
| Gastrointestinal cancer |  |
| Any gastrointestinal diseases |  |

Note: GERD, Gastroesophageal reflux disease; CD, Crohn’s disease; UC, Ulcerative colitis UC; NAFLD, Non-alcoholic fatty liver disease; BMI, Body mass index; AHEI, Alternative Healthy Eating Index; CVD, cardiovascular disease; NSAIDs, Nonsteroidal anti-inflammatory drugs; PPI, Proton pump inhibitors; INFLA-score, Immune biomarkers and an aggregated inflammation-score.

# Supplementary Table 4. Definition and list of long-term conditions in UK Biobank.

|  | **Morbidity grouping** | **Conditions included** | **UKB Data field** |
| --- | --- | --- | --- |
| **1** | Hypertension | Hypertension | 6150 (4), 6153 (2), 6177 (2),  20002 (1065) |
|  |  | Essential hypertension | 20002 (1072) |
| **2** | Diabetes | Diabetes | 2443 (1), 6153 (3), 6177 (3),  20002 (1220) |
|  |  | Type 1 diabetes | 20002 (1222) |
|  |  | Type 2 diabetes | 20002 (1223) |
|  |  | Diabetic nephropathy | 20002 (1607) |
|  |  | Diabetic neuropathy/ulcers | 20002 (1468) |
|  |  | Diabetic eye disease | 6148 (1), 20002 (1276) |
| **3** | Coronary heart disease | Heart attack/MI | 6150 (1), 20002 (1075) |
|  |  | Angina | 6150 (2), 20002 (1074) |
| **4** | Stroke/TIA | Stroke | 6150 (3), 20002 (1081) |
|  |  | TIA | 20002 (1082) |
|  |  | Subarachnoid haemorrhage | 20002 (1086) |
|  |  | Brain haemorrhage | 20002 (1491) |
|  |  | Ischaemic stroke | 20002 (1583) |
| **5** | Atrial fibrillation | Atrial fibrillation | 20002 (1471) |
| **6** | Heart failure | Heart failure/pulmonary oedema | 20002 (1076) |
|  |  | Cardiomyopathy | 20002 (1079) |
|  |  | Hypertrophic cardiomyopathy | 20002 (1588) |
| **7** | Peripheral vascular disease | Peripheral vascular disease | 20002 (1067) |
|  |  | Leg claudication/intermittent claudication | 20002 (1087) |
| **8** | COPD | COPD/Chronic obstructive pulmonary disease | 20002 (1112) |
|  |  | Emphysema/Chronic bronchitis | 6152 (6), 20002 (1113) |
|  |  | Emphysema | 20002 (1472) |
| **9** | Asthma | Asthma | 6152 (8), 20002 (1111) |
| **10** | Bronchiectasis | Bronchiectasis | 20002 (1114) |
| **11** | Depression | Depression | 20002 (1286) |
|  |  | Postnatal depression | 20002 (1531) |
| **12** | Anxiety | Anxiety/panic attacks | 20002 (1287) |
|  |  | Nervous breakdown | 20002 (1288) |
|  |  | Post-traumatic stress disorder | 20002 (1469) |
|  |  | Obsessive compulsive disorder | 20002 (1615) |
|  |  | Stress | 20002 (1614) |
|  |  | Insomnia | 1200 (3), 20002 (1616) |
|  |  | Psychological/psychiatric problem | 20002 (1243) |
| **13** | Schizophrenia/Bipolar affective disorder | Schizophrenia | 20002 (1289) |
|  |  | Mania | 20002 (1291) |
|  |  | Bipolar disorder | 20002 (1291) |
|  |  | Manic depression | 20002 (1291) |
| **14** | Connective tissue diseases | Myositis/myopathy | 20002 (1322) |
|  |  | Systemic lupus erythematosus/SLE | 20002 (1381) |
|  |  | Connective tissue disorder | 20002 (1373) |
|  |  | Sjogren’s syndrome.sicca syndrome | 20002 (1382) |
|  |  | Dermatopolymyositis | 20002 (1383) |
|  |  | Scloeroderma/systemic sclerosis | 20002 (1384) |
|  |  | Rheumatoid arthritis | 20002 (1464) |
|  |  | Psoriatic arthropathy | 20002 (1477) |
|  |  | Dermatomyositis | 20002 (1480) |
|  |  | Polymyositis | 20002 (1481) |
|  |  | Polymyalgia rheumatica | 20002 (1377) |
| **15** | Painful conditions | Back pain | 20002 (1534) |
|  |  | Joint pain | 20002 (1537) |
|  |  | Headaches (not migraine) | 20002 (1436) |
|  |  | Sciatica | 20002 (1476) |
|  |  | Plantar fasciitis | 20002 (1540) |
|  |  | Carpal tunnel syndrome | 20002 (1541) |
|  |  | Fibromyalgia | 20002 (1542) |
|  |  | Arthritis | 20002 (1538) |
|  |  | Shingles | 20002 (1573) |
|  |  | Disc problem | 20002 (1532) |
|  |  | Prolapsed disc/slipped disc | 20002 (1312) |
|  |  | Spine arthritis/spondylitis | 20002 (1311) |
|  |  | Ankylosing spondylitis | 20002 (1313) |
|  |  | Back problem | 20002 (1294) |
|  |  | Osteoarthritis | 20002 (1465) |
|  |  | Gout | 20002 (1466) |
|  |  | Cervical spondylosis | 20002 (1478) |
|  |  | Trigeminal neuralgia | 20002 (1523) |
|  |  | Disc degeneration | 20002 (1533) |
|  |  | Trapped nerve/compressed nerve | 20002 (1257) |
| **16** | Osteoporosis | Osteoporosis | 20002 (1309) |
| **17** | Thyroid disorders | Thyroid problem (not cancer) | 20002 (1224) |
|  |  | Hyperthroidism/thyrotoxicosis | 20002 (1225) |
|  |  | Hypothyroidism/myxoedema | 20002 (1226) |
|  |  | Grave’s disease | 20002 (1522) |
|  |  | Thyroid goitre | 20002 (1610) |
|  |  | Thyroititis | 20002 (1428) |
| **18** | Alcohol problems | Alcohol dependency | 20002 (1408) |
|  |  | Alcoholic liver disease/alcoholic cirrhosis | 20002 (1604) |
| **19** | Chronic kidney disease | Polycystic kidney | 20002 (1427) |
|  |  | Diabetic nephropathy | 20002 (1607) |
|  |  | Renal/kidney failure | 20002 (1192) |
|  |  | Renal failure requiring dialysis | 20002 (1193) |
|  |  | Renal failure not requiring dialysis | 20002 (1194) |
|  |  | Kidney nephropathy | 20002 (1519) |
|  |  | Immunoglobulin A (IgA) nephropathy | 20002 (1520) |
| **20** | Prostate disorders | Prostate problem (not cancer) | 20002 (1207) |
|  |  | Enlarged prostate | 20002 (1396) |
|  |  | Benign prostatic hypertrophy | 20002 (1516) |
| **21** | Glaucoma | Glaucoma | 6148 (2), 20002 (1277) |
| **22** | Epilepsy | Epilepsy | 20002 (1264) |
| **23** | Dementia | Dementia/Alzheimer/cognitive impairment | 20002 (1263) |
| **24** | Psoriasis or eczema | Eczema/dermatitis | 20002 (1452) |
|  |  | Psoriasis | 20002 (1453) |
| **25** | Migraine | Migraine | 20002 (1265) |
| **26** | Chronic sinusitis | Chronic sinusitis | 20002 (1416) |
| **27** | Anorexia or bulimia | Anorexia, bulimia/other eating disorder | 20002 (1470) |
| **28** | Parkinson’s disease | Parkinson’s disease | 20002 (1262) |
| **29** | Multiple sclerosis | Multiple sclerosis | 20002 (1261) |
| **30** | Chronic fatigue syndrome | Chronic fatigue syndrome | 20002 (1482) |
| **31** | Endometriosis | Endometriosis | 20002 (1402) |
| **32** | Meniere disease | Meniere disease | 20002 (1421) |
| **33** | Pernicious anaemia | Pernicious anaemia | 20002 (1331) |
| **34** | Polycystic ovaries | Polycystic ovaries | 20002 (1350) |

# Supplementary Table 5. Detail information of SNP for genetic risk score.

| **number** | **Outcomes** | **SNP** | **CHR** | **EA** | **beta** |
| --- | --- | --- | --- | --- | --- |
| 1 | NAFLD | rs1497406 | 1 | A/G | -0.042 |
| 2 |  | rs79598313 | 1 | T/C | 0.180 |
| 3 |  | rs74816838 | 1 | T/C | 0.087 |
| 4 |  | rs1337101 | 1 | T/G | -0.051 |
| 5 |  | rs2642438 | 1 | A/G | -0.079 |
| 6 |  | rs848559 | 2 | T/A | -0.061 |
| 7 |  | rs10195619 | 2 | T/C | -0.049 |
| 8 |  | rs13409360 | 2 | A/G | -0.060 |
| 9 |  | rs6717858 | 2 | C/T | -0.051 |
| 10 |  | rs73024760 | 2 | T/C | 0.107 |
| 11 |  | rs10201587 | 2 | G/A | -0.045 |
| 12 |  | rs2138157 | 2 | A/C | -0.064 |
| 13 |  | rs7604422 | 2 | C/A | -0.053 |
| 14 |  | rs4684847 | 3 | T/C | -0.072 |
| 15 |  | rs9867368 | 3 | A/G | -0.078 |
| 16 |  | rs9833411 | 3 | A/T | -0.048 |
| 17 |  | rs1594320 | 3 | G/C | 0.053 |
| 18 |  | rs12486792 | 3 | G/C | 0.060 |
| 19 |  | rs574044675 | 3 | C/A | -0.251 |
| 20 |  | rs12500824 | 4 | A/G | 0.046 |
| 21 |  | rs71633358 | 4 | C/T | -0.090 |
| 22 |  | rs4148824 | 7 | G/A | -0.057 |
| 23 |  | rs4841133 | 8 | A/G | 0.130 |
| 24 |  | rs4484649 | 8 | C/A | 0.045 |
| 25 |  | rs4734654 | 8 | G/A | -0.052 |
| 26 |  | rs2954038 | 8 | C/A | 0.139 |
| 27 |  | rs147998249 | 8 | C/G | -2.019 |
| 28 |  | rs7041363 | 9 | G/C | -0.135 |
| 29 |  | rs10883451 | 10 | C/T | -0.161 |
| 30 |  | rs17780834 | 10 | T/A | 0.086 |
| 31 |  | rs2792751 | 10 | T/C | 0.072 |
| 32 |  | rs11601507 | 11 | A/C | 0.088 |
| 33 |  | rs174535 | 11 | C/T | -0.061 |
| 34 |  | rs7117339 | 11 | T/C | -0.130 |
| 35 |  | rs4919741 | 12 | A/G | -0.057 |
| 36 |  | rs1169292 | 12 | T/C | 0.054 |
| 37 |  | rs148015593 | 12 | G/T | -0.042 |
| 38 |  | rs11621792 | 14 | T/C | 0.042 |
| 39 |  | rs28929474 | 14 | T/C | 0.481 |
| 40 |  | rs168144 | 15 | C/T | -0.052 |
| 41 |  | rs55868793 | 15 | G/T | 0.059 |
| 42 |  | rs72754571 | 15 | A/C | -0.083 |
| 43 |  | rs112128680 | 16 | A/G | 0.061 |
| 44 |  | rs4782568 | 16 | G/C | -0.064 |
| 45 |  | rs1801689 | 17 | C/A | 0.176 |
| 46 |  | rs4940689 | 18 | A/G | 0.054 |
| 47 |  | rs3810367 | 19 | G/T | 0.045 |
| 48 |  | rs58542926 | 19 | T/C | 0.222 |
| 49 |  | rs7599 | 19 | A/G | 0.049 |
| 50 |  | rs429358 | 19 | C/T | -0.094 |
| 51 |  | rs2377957 | 20 | A/G | -0.053 |
| 52 |  | rs2207132 | 20 | A/G | 0.189 |
| 53 |  | rs1547014 | 22 | T/C | -0.064 |
| 54 |  | rs132665 | 22 | G/A | -0.069 |
| 55 |  | rs738409 | 22 | G/C | 0.269 |
| 1 | Cirrhosis | rs28929474 | 14 | T | 0.708 |
| 2 |  | rs738409 | 22 | G | 0.489 |
| 3 |  | rs58542926 | 19 | T | 0.365 |
| 4 |  | rs1799992 | 11 | C | 0.207 |
| 5 |  | rs1883711 | 20 | C | 0.191 |
| 6 |  | rs888655 | 5 | G | -0.073 |
| 7 |  | rs9398804 | 6 | A | -0.073 |
| 8 |  | rs2642438 | 1 | A | -0.094 |
| 9 |  | rs12904 | 1 | A | -0.105 |
| 10 |  | rs7029757 | 9 | A | -0.163 |
| 11 |  | rs429358 | 19 | C | -0.163 |
| 12 |  | rs6834314 | 4 | G | -0.163 |
| 1 | GERD | rs1937450 | 1 | T/G | -0.03 |
| 2 |  | rs7552188 | 1 | T/C | 0.039 |
| 3 |  | rs11901649 | 2 | G/A | 0.03 |
| 4 |  | rs4362541 | 2 | A/T | 0.049 |
| 5 |  | rs7609078 | 2 | G/A | 0.039 |
| 6 |  | rs74652506 | 3 | C/T | -0.041 |
| 7 |  | rs7613875 | 3 | C/A | -0.03 |
| 8 |  | rs4676893 | 3 | A/T | 0.039 |
| 9 |  | rs809955 | 4 | G/A | 0.039 |
| 10 |  | rs10940767 | 5 | T/A | -0.041 |
| 11 |  | rs72771256 | 5 | G/A | 0.039 |
| 12 |  | rs7763910 | 6 | A/G | -0.041 |
| 13 |  | rs9266237 | 6 | G/C | -0.041 |
| 14 |  | rs4721096 | 7 | T/C | -0.041 |
| 15 |  | rs10242223 | 7 | A/G | 0.039 |
| 16 |  | rs10228350 | 7 | A/T | -0.03 |
| 17 |  | rs12706746 | 7 | G/A | -0.03 |
| 18 |  | rs12792379 | 11 | G/A | -0.041 |
| 19 |  | rs11171710 | 12 | G/A | -0.03 |
| 20 |  | rs597808 | 12 | A/G | 0.03 |
| 21 |  | rs34796998 | 17 | C/G | -0.041 |
| 22 |  | rs1363119 | 19 | A/G | 0.039 |
| 23 |  | rs12974777 | 19 | C/T | 0.039 |
| 24 |  | rs1297211 | 21 | C/G | 0.03 |
| 25 |  | rs7282609 | 21 | A/G | -0.03 |
| 1 | Diverticulum | rs6734367 | 2 | G | -0.010 |
| 2 |  | rs4333882 | 1 | G | 0.007 |
| 3 |  | rs7609897 | 3 | T | -0.006 |
| 4 |  | rs7086249 | 10 | C | -0.004 |
| 5 |  | rs1802575 | 2 | C | 0.006 |
| 6 |  | rs11667256 | 19 | T | -0.004 |
| 7 |  | rs962369 | 11 | C | -0.005 |
| 8 |  | rs6949391 | 7 | T | 0.004 |
| 9 |  | rs61823192 | 1 | T | -0.012 |
| 10 |  | rs9520344 | 13 | A | -0.004 |
|  |  | rs11619840 |  | A | -0.004 |
| 11 |  | rs7098322 | 10 | T | -0.006 |
| 12 |  | rs10472291 | 5 | A | 0.004 |
| 13 |  | rs582094 | 9 | T | -0.004 |
| 14 |  | rs75434097 | 21 | A | 0.005 |
| 15 |  | rs2280028 | 16 | A | -0.005 |
| 16 |  | rs9856118 | 3 | G | -0.005 |
| 17 |  | rs71472433 | 15 | C | 0.005 |
| 18 |  | rs2131755 | 16 | G | 0.003 |
| 19 |  | rs4839715 | 6 | A | -0.004 |
| 20 |  | rs148376933 | 2 | T* | 1.1564 |
| 21 |  | rs1381335 | 8 | T | -0.004 |
| 22 |  | rs61814883 | 1 | A | -0.004 |
| 23 |  | rs8074740 | 17 | A | 0.004 |
| 24 |  | rs3113037 | 7 | T | 0.004 |
| 25 |  | rs12293535 | 11 | A | 0.004 |
| 26 |  | rs875107 | 11 | A | -0.003 |
|  |  | rs72945112 |  | T | -0.003 |
| 27 |  | rs3823878 | 7 | A | 0.007 |
| 28 |  | rs10471645 | 5 | C | -0.004 |
| 29 |  | rs1888693 | 10 | A | -0.003 |
| 30 |  | rs4871180 | 8 | T | 0.004 |
| 31 |  | rs2049865 | 8 | A | 0.003 |
| 32 |  | rs1544387 | 4 | T | 0.003 |
| 33 |  | rs11934833 | 4 | G | -0.003 |
| 34 |  | rs2784255 | 1 | C | -0.003 |
| 35 |  | rs10120333 | 9 | G | 0.003 |
| 36 |  | rs12942267 | 17 | T | 0.003 |
| 37 |  | rs62126581 | 2 | A | -0.004 |
| 38 |  | rs115490395 | 1 | A | 0.016 |
| 39 |  | rs2470653 | 3 | A | -0.004 |
| 40 |  | rs10173528 | 2 | T | -0.003 |
| 41 |  | rs2056544 | 15 | A | 0.003 |
|  |  | rs10519134 |  | A | 0.004 |
| 42 |  | rs138699 | 22 | A | 0.003 |
| 1 | Peptic ulcer | rs147048677 | 1 | C | -0.15082 |
| 2 |  | rs2976388 | 8 | G | 0.086178 |
| 3 |  | rs687621 | 9 | A | 0.076961 |
| 4 |  | rs78459074 | 11 | A | 0.113329 |
| 5 |  | rs10500661 | 11 | T | -0.10536 |
| 6 |  | rs9581957 | 13 | C | -0.07257 |
| 7 |  | rs34074411 | 17 | C | -0.07257 |
| 8 |  | rs681343 | 19 | C | -0.08338 |

SNP, single nucleotide polymorphism; CHR, Chromosome; EA, effect allele. GERD, Gastroesophageal reflux disease; NAFLD, Non-alcoholic fatty liver disease.

# Supplementary Table 6. Detailed information on missing covariates.

| **Covariates** | **N*** | **Missing rate (%)** |
| --- | --- | --- |
| Townsend deprivation index | 155 | 0.1 |
| Ethnicity | 356 | 0.2 |
| BMI | 184 | 0.1 |
| Basal metabolic rate | 1724 | 1.2 |
| Education | 413 | 0.3 |
| Current employment status | 211 | 0.1 |
| Hot drink temperature | 11 | 0.0 |
| Family history of cancer | 1201 | 0.8 |
| Family history of CVD | 1201 | 0.8 |
| NSAIDs user | 934 | 0.6 |
| Physical activity | 19938 | 13.5 |
| Smoking status | 130 | 0.1 |
| Pack-years of smoking for current or former smokers | 20752 | 14.1 |
| Vitamin use | 216 | 0.1 |
| Minerals and other dietary supplements use | 78 | 0.1 |
| Healthy sleep pattern | 22311 | 15.2 |
| PPI | 2 | 0.0 |
| INFLA | 11421 | 7.8 |

Note: *N represents the number of missing responses.

Abbreviations: BMI, body mass index (calculated as weight in kilograms divided by height in meters squared); CVD, cardiovascular disease; NSAIDs, Nonsteroidal anti-inflammatory drugs (Aspirin, Ibuprofen, and Paracetamol); PPI, Proton pump inhibitors; INFLA, immune biomarkers and an aggregated inflammation.

# Supplementary Table 7. Associations of coffee consumption with risk of Gastrointestinal disease incident.

| **Category of coffee/diseases** | **Coffee intake (cups/d)** | **Number of participants** | **Case** | **Person-year** | **Basic model** | | **Multivariable model** | |
| --- | --- | --- | --- | --- | --- | --- | --- | --- |
|  |  |  |  |  | **HR (95% CI)** | **P value** | **HR (95% CI)** | **P value** |
| **Barrett’s oesophagus** |  |  |  |  |  |  |  |  |
| **Unsweetened coffee** | 0 | 35528 | 309 | 453611.3 | 1 (Reference) | 1 | 1 (Reference) | 1 |
|  | ≤ 1 | 17627 | 133 | 226657.7 | 0.79 (0.64-0.97) | 0.023 | 0.92 (0.75-1.14) | 0.448 |
|  | 1-2 | 22684 | 160 | 292328.2 | 0.72 (0.60-0.88) | 0.001 | 0.89 (0.73-1.09) | 0.248 |
|  | 2-3 | 18561 | 131 | 239258.7 | 0.72 (0.58-0.88) | 0.001 | 0.90 (0.73-1.12) | 0.358 |
|  | 3-4 | 12583 | 94 | 162308.2 | 0.77 (0.61-0.97) | 0.024 | 0.98 (0.76-1.25) | 0.847 |
|  | >4 | 11022 | 87 | 141762.1 | 0.83 (0.65-1.05) | 0.115 | 1.03 (0.79-1.33) | 0.851 |
| **Sugar-sweetened coffee** | 0 | 35528 | 309 | 453611.3 | 1 (Reference) | 1 | 1 (Reference) | 1 |
|  | ≤ 1 | 6512 | 86 | 82862.24 | 1.25 (0.98-1.59) | 0.07 | 1.37 (1.07-1.76) | 0.012 |
|  | 1-2 | 5963 | 45 | 76214.76 | 0.68 (0.49-0.93) | 0.015 | 0.78 (0.57-1.09) | 0.142 |
|  | 2-3 | 3935 | 35 | 50242.13 | 0.78 (0.55-1.11) | 0.175 | 0.95 (0.66-1.37) | 0.790 |
|  | 3-4 | 2256 | 15 | 28649.84 | 0.58 (0.34-0.97) | 0.039 | 0.69 (0.41-1.19) | 0.181 |
|  | >4 | 2013 | 30 | 25382.6 | 1.36 (0.93-1.98) | 0.11 | 1.61 (1.07-2.42) | 0.024 |
| **Artificially sweetened coffee** | 0 | 35528 | 309 | 453611.3 | 1 (Reference) | 1 | 1 (Reference) | 1 |
|  | ≤ 1 | 1944 | 28 | 24666.14 | 1.41 (0.96-2.08) | 0.082 | 1.37 (0.92-2.03) | 0.117 |
|  | 1-2 | 2205 | 16 | 28200.38 | 0.70 (0.42-1.16) | 0.168 | 0.70 (0.42-1.16) | 0.167 |
|  | 2-3 | 1820 | 11 | 23217.23 | 0.57 (0.31-1.05) | 0.07 | 0.63 (0.34-1.17) | 0.142 |
|  | 3-4 | 1296 | 13 | 16507.83 | 0.94 (0.54-1.63) | 0.814 | 1.03 (0.58-1.83) | 0.926 |
|  | >4 | 1314 | 15 | 16706.7 | 1.08 (0.64-1.81) | 0.778 | 1.19 (0.68-2.06) | 0.544 |
| **GERD** |  |  |  |  |  |  |  |  |
| **Unsweetened coffee** | 0 | 35528 | 2273 | 442959.3 | 1 (Reference) | 1 | 1 (Reference) | 1 |
|  | ≤ 1 | 17627 | 1030 | 221536 | 0.84 (0.78-0.90) | <0.001 | 0.95 (0.88-1.03) | 0.196 |
|  | 1-2 | 22684 | 1200 | 286720.1 | 0.75 (0.70-0.80) | <0.001 | 0.88 (0.82-0.94) | <0.001 |
|  | 2-3 | 18561 | 933 | 234970.7 | 0.71 (0.66-0.77) | <0.001 | 0.84 (0.77-0.91) | <0.001 |
|  | 3-4 | 12583 | 708 | 158973 | 0.81 (0.74-0.88) | <0.001 | 0.95 (0.87-1.04) | 0.262 |
|  | >4 | 11022 | 634 | 138710.4 | 0.85 (0.78-0.93) | <0.001 | 0.96 (0.87-1.06) | 0.425 |
| **Sugar-sweetened coffee** | 0 | 35528 | 2273 | 442959.3 | 1 (Reference) | 1 | 1 (Reference) | 1 |
|  | ≤ 1 | 6512 | 452 | 80729.9 | 1.01 (0.92-1.12) | 0.776 | 1.06 (0.96-1.18) | 0.272 |
|  | 1-2 | 5963 | 373 | 74358.09 | 0.91 (0.82-1.02) | 0.099 | 0.96 (0.85-1.07) | 0.448 |
|  | 2-3 | 3935 | 278 | 48933.9 | 1.04 (0.92-1.18) | 0.552 | 1.09 (0.95-1.24) | 0.208 |
|  | 3-4 | 2256 | 147 | 27866.26 | 0.97 (0.82-1.14) | 0.681 | 1.00 (0.84-1.19) | 0.966 |
|  | >4 | 2013 | 127 | 24917.4 | 0.98 (0.82-1.17) | 0.802 | 0.94 (0.77-1.13) | 0.488 |
| **Artificially sweetened coffee** | 0 | 35528 | 2273 | 442959.3 | 1 (Reference) | 1 | 1 (Reference) | 1 |
|  | ≤ 1 | 1944 | 147 | 23963.86 | 1.07 (0.90-1.26) | 0.445 | 0.97 (0.82-1.15) | 0.714 |
|  | 1-2 | 2205 | 165 | 27359.82 | 1.05 (0.89-1.23) | 0.56 | 0.93 (0.79-1.10) | 0.392 |
|  | 2-3 | 1820 | 135 | 22546.22 | 1.04 (0.87-1.23) | 0.687 | 0.99 (0.82-1.18) | 0.883 |
|  | 3-4 | 1296 | 83 | 16127.52 | 0.89 (0.72-1.11) | 0.311 | 0.84 (0.67-1.05) | 0.121 |
|  | >4 | 1314 | 107 | 16233.52 | 1.15 (0.94-1.39) | 0.168 | 1.01 (0.82-1.24) | 0.961 |
| **Gastritis and duodenitis** |  |  |  |  |  |  |  |  |
| **Unsweetened coffee** | 0 | 35528 | 2268 | 441280 | 1 (Reference) | 1 | 1 (Reference) | 1 |
|  | ≤ 1 | 17627 | 998 | 221182.9 | 0.82 (0.76-0.88) | <0.001 | 0.91 (0.84-0.98) | 0.014 |
|  | 1-2 | 22684 | 1147 | 286091.6 | 0.73 (0.68-0.78) | <0.001 | 0.82 (0.76-0.89) | <0.001 |
|  | 2-3 | 18561 | 897 | 234545.7 | 0.70 (0.64-0.75) | <0.001 | 0.80 (0.73-0.86) | <0.001 |
|  | 3-4 | 12583 | 624 | 159154.3 | 0.72 (0.66-0.79) | <0.001 | 0.82 (0.75-0.90) | <0.001 |
|  | >4 | 11022 | 530 | 139203.1 | 0.72 (0.65-0.79) | <0.001 | 0.78 (0.71-0.87) | <0.001 |
| **Sugar-sweetened coffee** | 0 | 35528 | 2268 | 441280 | 1 (Reference) | 1 | 1 (Reference) | 1 |
|  | ≤ 1 | 6512 | 382 | 80917.74 | 0.85 (0.76-0.95) | 0.003 | 0.86 (0.77-0.96) | 0.007 |
|  | 1-2 | 5963 | 353 | 74223.03 | 0.86 (0.77-0.97) | 0.011 | 0.88 (0.78-0.99) | 0.036 |
|  | 2-3 | 3935 | 221 | 49102.09 | 0.82 (0.72-0.95) | 0.006 | 0.84 (0.72-0.97) | 0.017 |
|  | 3-4 | 2256 | 117 | 27974.39 | 0.77 (0.64-0.92) | 0.005 | 0.76 (0.63-0.93) | 0.006 |
|  | >4 | 2013 | 120 | 24797.96 | 0.93 (0.77-1.12) | 0.425 | 0.85 (0.70-1.04) | 0.108 |
| **Artificially sweetened coffee** | 0 | 35528 | 2268 | 441280 | 1 (Reference) | 1 | 1 (Reference) | 1 |
|  | ≤ 1 | 1944 | 144 | 23877.41 | 1.05 (0.89-1.24) | 0.566 | 0.98 (0.83-1.16) | 0.817 |
|  | 1-2 | 2205 | 159 | 27374.08 | 1.02 (0.86-1.19) | 0.848 | 0.93 (0.79-1.10) | 0.394 |
|  | 2-3 | 1820 | 107 | 22647.91 | 0.83 (0.68-1.00) | 0.053 | 0.80 (0.66-0.98) | 0.030 |
|  | 3-4 | 1296 | 94 | 16052.78 | 1.02 (0.83-1.26) | 0.835 | 0.97 (0.78-1.20) | 0.783 |
|  | >4 | 1314 | 97 | 16192.85 | 1.06 (0.86-1.29) | 0.607 | 0.96 (0.77-1.19) | 0.705 |
| **Celiac disease** |  |  |  |  |  |  |  |  |
| **Unsweetened coffee** | 0 | 35528 | 122 | 454432.1 | 1 (Reference) | 1 | 1 (Reference) | 1 |
|  | ≤ 1 | 17627 | 56 | 227012.3 | 0.88 (0.64-1.21) | 0.420 | 0.92 (0.66-1.27) | 0.598 |
|  | 1-2 | 22684 | 64 | 292768.3 | 0.77 (0.57-1.05) | 0.099 | 0.84 (0.62-1.16) | 0.291 |
|  | 2-3 | 18561 | 43 | 239683.2 | 0.63 (0.45-0.90) | 0.011 | 0.71 (0.49-1.03) | 0.071 |
|  | 3-4 | 12583 | 33 | 162603.1 | 0.72 (0.49-1.06) | 0.096 | 0.83 (0.55-1.25) | 0.372 |
|  | >4 | 11022 | 23 | 142078.6 | 0.58 (0.37-0.90) | 0.016 | 0.69 (0.43-1.10) | 0.118 |
| **Sugar-sweetened coffee** | 0 | 35528 | 122 | 454432.1 | 1 (Reference) | 1 | 1 (Reference) | 1 |
|  | ≤ 1 | 6512 | 20 | 83160.54 | 0.91 (0.56-1.46) | 0.682 | 0.90 (0.55-1.45) | 0.653 |
|  | 1-2 | 5963 | 17 | 76379.17 | 0.84 (0.50-1.40) | 0.509 | 0.90 (0.53-1.53) | 0.702 |
|  | 2-3 | 3935 | 5 | 50409.04 | 0.38 (0.15-0.92) | 0.033 | 0.43 (0.17-1.07) | 0.070 |
|  | 3-4 | 2256 | 0 | 28732.48 | 0 (0-Inf) | 0.989 | 0 (0-Inf) | 0.989 |
|  | >4 | 2013 | 7 | 25503.73 | 1.08 (0.50-2.32) | 0.849 | 1.39 (0.62-3.13) | 0.421 |
| **Artificially sweetened coffee** | 0 | 35528 | 122 | 454432.1 | 1 (Reference) | 1 | 1 (Reference) | 1 |
|  | ≤ 1 | 1944 | 6 | 24772.08 | 0.86 (0.38-1.96) | 0.72 | 1.05 (0.46-2.41) | 0.908 |
|  | 1-2 | 2205 | 3 | 28274.12 | 0.38 (0.12-1.18) | 0.094 | 0.52 (0.16-1.66) | 0.269 |
|  | 2-3 | 1820 | 4 | 23252.65 | 0.60 (0.22-1.64) | 0.321 | 0.91 (0.33-2.52) | 0.857 |
|  | 3-4 | 1296 | 3 | 16577.42 | 0.64 (0.20-2.02) | 0.446 | 1.08 (0.34-3.50) | 0.893 |
|  | >4 | 1314 | 3 | 16779.83 | 0.63 (0.20-1.97) | 0.423 | 1.17 (0.36-3.81) | 0.796 |
| **Peptic ulcer** |  |  |  |  |  |  |  |  |
| **Unsweetened coffee** | 0 | 35528 | 490 | 452237.7 | 1 (Reference) | 1 | 1 (Reference) | 1 |
|  | ≤ 1 | 17627 | 177 | 226312 | 0.67 (0.57-0.80) | <0.001 | 0.74 (0.62-0.89) | <0.001 |
|  | 1-2 | 22684 | 230 | 291830 | 0.68 (0.58-0.79) | <0.001 | 0.74 (0.63-0.87) | <0.001 |
|  | 2-3 | 18561 | 171 | 238971.2 | 0.62 (0.52-0.73) | <0.001 | 0.66 (0.55-0.80) | <0.001 |
|  | 3-4 | 12583 | 138 | 162032 | 0.74 (0.61-0.90) | 0.002 | 0.77 (0.63-0.95) | 0.013 |
|  | >4 | 11022 | 116 | 141591.8 | 0.73 (0.60-0.90) | 0.003 | 0.70 (0.56-0.88) | 0.002 |
| **Sugar-sweetened coffee** | 0 | 35528 | 490 | 452237.7 | 1 (Reference) | 1 | 1 (Reference) | 1 |
|  | ≤ 1 | 6512 | 81 | 82846.14 | 0.79 (0.62-1.00) | 0.046 | 0.80 (0.63-1.01) | 0.065 |
|  | 1-2 | 5963 | 70 | 76017.3 | 0.74 (0.57-0.95) | 0.018 | 0.75 (0.58-0.97) | 0.029 |
|  | 2-3 | 3935 | 55 | 50137.56 | 0.88 (0.67-1.17) | 0.386 | 0.87 (0.65-1.17) | 0.369 |
|  | 3-4 | 2256 | 28 | 28575.75 | 0.78 (0.54-1.15) | 0.214 | 0.76 (0.51-1.13) | 0.168 |
|  | >4 | 2013 | 28 | 25444.2 | 0.93 (0.63-1.36) | 0.694 | 0.79 (0.52-1.18) | 0.252 |
| **Artificially sweetened coffee** | 0 | 35528 | 490 | 452237.7 | 1 (Reference) | 1 | 1 (Reference) | 1 |
|  | ≤ 1 | 1944 | 33 | 24656.03 | 1.07 (0.75-1.52) | 0.716 | 1.00 (0.70-1.43) | 0.991 |
|  | 1-2 | 2205 | 42 | 28071.56 | 1.20 (0.88-1.65) | 0.254 | 1.08 (0.78-1.50) | 0.626 |
|  | 2-3 | 1820 | 26 | 23150.85 | 0.89 (0.60-1.33) | 0.575 | 0.86 (0.57-1.30) | 0.484 |
|  | 3-4 | 1296 | 16 | 16502.68 | 0.77 (0.47-1.26) | 0.298 | 0.71 (0.43-1.19) | 0.195 |
|  | >4 | 1314 | 22 | 16669.95 | 1.06 (0.69-1.63) | 0.792 | 0.93 (0.59-1.46) | 0.752 |
| **Crohn’s disease** |  |  |  |  |  |  |  |  |
| **Unsweetened coffee** | 0 | 35528 | 58 | 454866.4 | 1 (Reference) | 1 | 1 (Reference) | 1 |
|  | ≤ 1 | 17627 | 20 | 227286.7 | 0.72 (0.43-1.20) | 0.203 | 0.79 (0.47-1.33) | 0.379 |
|  | 1-2 | 22684 | 30 | 292986.6 | 0.84 (0.54-1.31) | 0.435 | 0.96 (0.60-1.52) | 0.848 |
|  | 2-3 | 18561 | 20 | 239853.9 | 0.68 (0.41-1.13) | 0.139 | 0.79 (0.46-1.35) | 0.389 |
|  | 3-4 | 12583 | 11 | 162735.6 | 0.55 (0.29-1.05) | 0.068 | 0.64 (0.32-1.25) | 0.188 |
|  | >4 | 11022 | 24 | 142085.7 | 1.36 (0.84-2.20) | 0.209 | 1.55 (0.91-2.64) | 0.106 |
| **Sugar-sweetened coffee** | 0 | 35528 | 58 | 454866.4 | 1 (Reference) | 1 | 1 (Reference) | 1 |
|  | ≤ 1 | 6512 | 14 | 83198.13 | 1.24 (0.69-2.22) | 0.481 | 1.19 (0.65-2.17) | 0.568 |
|  | 1-2 | 5963 | 15 | 76380.91 | 1.43 (0.80-2.53) | 0.225 | 1.34 (0.73-2.46) | 0.338 |
|  | 2-3 | 3935 | 5 | 50411.06 | 0.71 (0.29-1.79) | 0.474 | 0.66 (0.25-1.70) | 0.387 |
|  | 3-4 | 2256 | 2 | 28715.62 | 0.50 (0.12-2.04) | 0.332 | 0.43 (0.10-1.83) | 0.255 |
|  | >4 | 2013 | 3 | 25541.42 | 0.84 (0.26-2.69) | 0.766 | 0.66 (0.19-2.25) | 0.503 |
| **Artificially sweetened coffee** | 0 | 35528 | 58 | 454866.4 | 1 (Reference) | 1 | 1 (Reference) | 1 |
|  | ≤ 1 | 1944 | 3 | 24790.63 | 0.99 (0.31-3.16) | 0.981 | 0.90 (0.28-2.93) | 0.863 |
|  | 1-2 | 2205 | 4 | 28257.89 | 1.16 (0.42-3.21) | 0.774 | 1.01 (0.35-2.89) | 0.982 |
|  | 2-3 | 1820 | 3 | 23250.49 | 1.05 (0.33-3.37) | 0.933 | 0.95 (0.29-3.17) | 0.938 |
|  | 3-4 | 1296 | 1 | 16585.06 | 0.48 (0.07-3.49) | 0.471 | 0.41 (0.06-3.11) | 0.391 |
|  | >4 | 1314 | 1 | 16793.15 | 0.48 (0.07-3.49) | 0.469 | 0.37 (0.05-2.78) | 0.331 |
| **Ulcerative colitis (UC)** |  |  |  |  |  |  |  |  |
| **Unsweetened coffee** | 0 | 35528 | 143 | 454397 | 1 (Reference) | 1 | 1 (Reference) | 1 |
|  | ≤ 1 | 17627 | 59 | 227017.7 | 0.81 (0.6-1.10) | 0.183 | 0.95 (0.69-1.30) | 0.741 |
|  | 1-2 | 22684 | 66 | 292762.2 | 0.70 (0.52-0.94) | 0.018 | 0.84 (0.62-1.14) | 0.261 |
|  | 2-3 | 18561 | 57 | 239601.1 | 0.73 (0.54-1.00) | 0.051 | 0.88 (0.63-1.22) | 0.449 |
|  | 3-4 | 12583 | 47 | 162532.6 | 0.89 (0.64-1.25) | 0.508 | 1.06 (0.74-1.51) | 0.761 |
|  | >4 | 11022 | 34 | 142029.3 | 0.74 (0.51-1.08) | 0.118 | 0.85 (0.56-1.27) | 0.417 |
| **Sugar-sweetened coffee** | 0 | 35528 | 143 | 454397 | 1 (Reference) | 1 | 1 (Reference) | 1 |
|  | ≤ 1 | 6512 | 20 | 83175.39 | 0.72 (0.45-1.15) | 0.172 | 0.73 (0.45-1.18) | 0.197 |
|  | 1-2 | 5963 | 20 | 76371.46 | 0.77 (0.48-1.23) | 0.271 | 0.77 (0.47-1.25) | 0.284 |
|  | 2-3 | 3935 | 18 | 50310.99 | 1.03 (0.63-1.70) | 0.895 | 1.02 (0.60-1.72) | 0.944 |
|  | 3-4 | 2256 | 4 | 28708.34 | 0.40 (0.15-1.08) | 0.069 | 0.37 (0.14-1.03) | 0.057 |
|  | >4 | 2013 | 7 | 25504.04 | 0.78 (0.36-1.66) | 0.513 | 0.65 (0.29-1.45) | 0.289 |
| **Artificially sweetened coffee** | 0 | 35528 | 143 | 454397 | 1 (Reference) | 1 | 1 (Reference) | 1 |
|  | ≤ 1 | 1944 | 8 | 24754.22 | 1.02 (0.50-2.08) | 0.96 | 0.98 (0.48-2.03) | 0.965 |
|  | 1-2 | 2205 | 14 | 28193.77 | 1.57 (0.90-2.72) | 0.111 | 1.53 (0.86-2.71) | 0.147 |
|  | 2-3 | 1820 | 9 | 23209.88 | 1.21 (0.62-2.38) | 0.58 | 1.20 (0.60-2.43) | 0.605 |
|  | 3-4 | 1296 | 5 | 16567.29 | 0.93 (0.38-2.27) | 0.871 | 0.89 (0.35-2.24) | 0.807 |
|  | >4 | 1314 | 6 | 16748.52 | 1.11 (0.49-2.51) | 0.808 | 1.02 (0.43-2.41) | 0.968 |
| **IBS** |  |  |  |  |  |  |  |  |
| **Unsweetened coffee** | 0 | 35528 | 411 | 453049.1 | 1 (Reference) | 1 | 1 (Reference) | 1 |
|  | ≤ 1 | 17627 | 201 | 226354.3 | 0.92 (0.78-1.09) | 0.338 | 1.00 (0.84-1.19) | 0.989 |
|  | 1-2 | 22684 | 217 | 292023.9 | 0.77 (0.65-0.91) | 0.002 | 0.86 (0.72-1.02) | 0.088 |
|  | 2-3 | 18561 | 173 | 239032.3 | 0.76 (0.64-0.91) | 0.003 | 0.86 (0.71-1.03) | 0.106 |
|  | 3-4 | 12583 | 124 | 162186 | 0.81 (0.66-0.99) | 0.042 | 0.90 (0.73-1.12) | 0.338 |
|  | >4 | 11022 | 101 | 141761.8 | 0.77 (0.62-0.96) | 0.018 | 0.82 (0.65-1.04) | 0.096 |
| **Sugar-sweetened coffee** | 0 | 35528 | 411 | 453049.1 | 1 (Reference) | 1 | 1 (Reference) | 1 |
|  | ≤ 1 | 6512 | 71 | 82936.07 | 1.00 (0.78-1.29) | 0.992 | 1.03 (0.79-1.33) | 0.831 |
|  | 1-2 | 5963 | 62 | 76137.21 | 0.99 (0.76-1.29) | 0.936 | 1.03 (0.78-1.36) | 0.851 |
|  | 2-3 | 3935 | 41 | 50247.98 | 1.01 (0.73-1.40) | 0.931 | 1.07 (0.77-1.5) | 0.682 |
|  | 3-4 | 2256 | 20 | 28631.72 | 0.89 (0.56-1.39) | 0.6 | 0.95 (0.60-1.51) | 0.819 |
|  | >4 | 2013 | 17 | 25463.61 | 0.88 (0.54-1.44) | 0.623 | 0.89 (0.53-1.48) | 0.649 |
| **Artificially sweetened coffee** | 0 | 35528 | 411 | 453049.1 | 1 (Reference) | 1 | 1 (Reference) | 1 |
|  | ≤ 1 | 1944 | 26 | 24687.32 | 1.08 (0.73-1.61) | 0.707 | 0.97 (0.65-1.45) | 0.893 |
|  | 1-2 | 2205 | 24 | 28171.99 | 0.87 (0.58-1.31) | 0.509 | 0.75 (0.49-1.15) | 0.186 |
|  | 2-3 | 1820 | 24 | 23138.43 | 1.07 (0.71-1.61) | 0.76 | 0.99 (0.65-1.52) | 0.968 |
|  | 3-4 | 1296 | 17 | 16510.3 | 1.08 (0.67-1.76) | 0.744 | 0.96 (0.58-1.59) | 0.885 |
|  | >4 | 1314 | 18 | 16705.34 | 1.12 (0.70-1.80) | 0.636 | 0.94 (0.57-1.55) | 0.808 |
| **Diverticulum** |  |  |  |  |  |  |  |  |
| **Unsweetened coffee** | 0 | 35528 | 3055 | 437555.7 | 1 (Reference) | 1 | 1 (Reference) | 1 |
|  | ≤ 1 | 17627 | 1541 | 218549.3 | 0.90 (0.85-0.96) | <0.001 | 0.96 (0.90-1.02) | 0.191 |
|  | 1-2 | 22684 | 1819 | 282840.6 | 0.81 (0.77-0.86) | <0.001 | 0.86 (0.81-0.92) | <0.001 |
|  | 2-3 | 18561 | 1484 | 231657.2 | 0.8 (0.76-0.86) | <0.001 | 0.85 (0.80-0.91) | <0.001 |
|  | 3-4 | 12583 | 997 | 157025.9 | 0.81 (0.76-0.87) | <0.001 | 0.84 (0.78-0.90) | <0.001 |
|  | >4 | 11022 | 860 | 137433.8 | 0.82 (0.76-0.88) | <0.001 | 0.81 (0.75-0.88) | <0.001 |
| **Sugar-sweetened coffee** | 0 | 35528 | 3055 | 437555.7 | 1 (Reference) | 1 | 1 (Reference) | 1 |
|  | ≤ 1 | 6512 | 624 | 79803.64 | 0.99 (0.91-1.08) | 0.804 | 1.00 (0.92-1.09) | 0.984 |
|  | 1-2 | 5963 | 540 | 73418.69 | 0.91 (0.83-1.00) | 0.054 | 0.92 (0.83-1.01) | 0.085 |
|  | 2-3 | 3935 | 342 | 48441.18 | 0.88 (0.78-0.98) | 0.021 | 0.88 (0.78-0.99) | 0.032 |
|  | 3-4 | 2256 | 205 | 27653.3 | 0.91 (0.79-1.05) | 0.212 | 0.89 (0.76-1.03) | 0.109 |
|  | >4 | 2013 | 189 | 24487.56 | 1.01 (0.87-1.17) | 0.92 | 0.94 (0.80-1.10) | 0.432 |
| **Artificially sweetened coffee** | 0 | 35528 | 3055 | 437555.7 | 1 (Reference) | 1 | 1 (Reference) | 1 |
|  | ≤ 1 | 1944 | 201 | 23724.81 | 1.02 (0.89-1.18) | 0.767 | 0.91 (0.79-1.06) | 0.226 |
|  | 1-2 | 2205 | 217 | 27011.08 | 0.96 (0.84-1.11) | 0.593 | 0.84 (0.73-0.97) | 0.016 |
|  | 2-3 | 1820 | 176 | 22232.19 | 0.93 (0.80-1.09) | 0.379 | 0.84 (0.72-0.99) | 0.033 |
|  | 3-4 | 1296 | 138 | 15747.31 | 1.03 (0.87-1.22) | 0.748 | 0.89 (0.74-1.06) | 0.195 |
|  | >4 | 1314 | 134 | 16102.42 | 0.98 (0.82-1.16) | 0.805 | 0.81 (0.68-0.98) | 0.029 |
| **Pancreatitis** |  |  |  |  |  |  |  |  |
| **Unsweetened coffee** | 0 | 35528 | 143 | 454454 | 1 (Reference) | 1 | 1 (Reference) | 1 |
|  | ≤ 1 | 17627 | 55 | 227091 | 0.71 (0.52-0.97) | 0.034 | 0.84 (0.61-1.15) | 0.279 |
|  | 1-2 | 22684 | 84 | 292729.3 | 0.84 (0.64-1.10) | 0.207 | 1.00 (0.75-1.33) | 0.988 |
|  | 2-3 | 18561 | 58 | 239651.4 | 0.71 (0.52-0.96) | 0.027 | 0.84 (0.61-1.17) | 0.299 |
|  | 3-4 | 12583 | 41 | 162620.7 | 0.75 (0.53-1.06) | 0.101 | 0.86 (0.60-1.25) | 0.436 |
|  | >4 | 11022 | 44 | 141968.3 | 0.94 (0.67-1.32) | 0.709 | 1.02 (0.70-1.48) | 0.922 |
| **Sugar-sweetened coffee** | 0 | 35528 | 143 | 454454 | 1 (Reference) | 1 | 1 (Reference) | 1 |
|  | ≤ 1 | 6512 | 36 | 83143.34 | 1.25 (0.87-1.81) | 0.234 | 1.31 (0.9-1.92) | 0.156 |
|  | 1-2 | 5963 | 25 | 76343.69 | 0.94 (0.61-1.44) | 0.763 | 0.96 (0.61-1.49) | 0.841 |
|  | 2-3 | 3935 | 13 | 50377.96 | 0.74 (0.42-1.30) | 0.293 | 0.73 (0.40-1.33) | 0.304 |
|  | 3-4 | 2256 | 8 | 28693.35 | 0.79 (0.39-1.62) | 0.52 | 0.73 (0.35-1.54) | 0.413 |
|  | >4 | 2013 | 7 | 25535.2 | 0.80 (0.37-1.72) | 0.567 | 0.66 (0.29-1.46) | 0.301 |
| **Artificially sweetened coffee** | 0 | 35528 | 143 | 454454 | 1 (Reference) | 1 | 1 (Reference) | 1 |
|  | ≤ 1 | 1944 | 9 | 24768.27 | 1.03 (0.52-2.02) | 0.941 | 0.87 (0.44-1.72) | 0.684 |
|  | 1-2 | 2205 | 11 | 28231.16 | 1.10 (0.60-2.05) | 0.751 | 0.89 (0.47-1.67) | 0.712 |
|  | 2-3 | 1820 | 9 | 23221.72 | 1.09 (0.55-2.15) | 0.801 | 0.91 (0.45-1.83) | 0.791 |
|  | 3-4 | 1296 | 6 | 16544.65 | 1.01 (0.45-2.30) | 0.975 | 0.75 (0.32-1.77) | 0.516 |
|  | >4 | 1314 | 9 | 16765.85 | 1.52 (0.77-2.98) | 0.229 | 1.02 (0.49-2.13) | 0.949 |
| **NAFLD** |  |  |  |  |  |  |  |  |
| **Unsweetened coffee** | 0 | 35528 | 296 | 454046.6 | 1 (Reference) | 1 | 1 (Reference) | 1 |
|  | ≤ 1 | 17627 | 92 | 226864.3 | 0.62 (0.49-0.78) | <0.001 | 0.75 (0.59-0.96) | 0.020 |
|  | 1-2 | 22684 | 110 | 292675.9 | 0.57 (0.46-0.71) | <0.001 | 0.66 (0.52-0.83) | <0.001 |
|  | 2-3 | 18561 | 89 | 239588.1 | 0.57 (0.45-0.72) | <0.001 | 0.61 (0.48-0.79) | <0.001 |
|  | 3-4 | 12583 | 67 | 162490.1 | 0.63 (0.48-0.82) | <0.001 | 0.62 (0.47-0.83) | 0.001 |
|  | >4 | 11022 | 71 | 141919.1 | 0.77 (0.59-1.00) | 0.046 | 0.66 (0.50-0.88) | 0.005 |
| **Sugar-sweetened coffee** | 0 | 35528 | 296 | 454046.6 | 1 (Reference) | 1 | 1 (Reference) | 1 |
|  | ≤ 1 | 6512 | 53 | 83052.95 | 0.92 (0.69-1.24) | 0.585 | 1.00 (0.74-1.35) | 0.990 |
|  | 1-2 | 5963 | 48 | 76246.82 | 0.91 (0.67-1.24) | 0.55 | 0.94 (0.68-1.29) | 0.693 |
|  | 2-3 | 3935 | 21 | 50353.48 | 0.60 (0.39-0.94) | 0.027 | 0.60 (0.38-0.95) | 0.031 |
|  | 3-4 | 2256 | 22 | 28650.27 | 1.11 (0.72-1.72) | 0.633 | 0.99 (0.62-1.56) | 0.949 |
|  | >4 | 2013 | 21 | 25457.98 | 1.22 (0.78-1.91) | 0.38 | 0.92 (0.57-1.49) | 0.736 |
| **Artificially sweetened coffee** | 0 | 35528 | 296 | 454046.6 | 1 (Reference) | 1 | 1 (Reference) | 1 |
|  | ≤ 1 | 1944 | 21 | 24742.25 | 1.25 (0.8-1.95) | 0.326 | 0.85 (0.54-1.34) | 0.485 |
|  | 1-2 | 2205 | 36 | 28097.96 | 1.89 (1.34-2.68) | <0.001 | 1.16 (0.81-1.67) | 0.415 |
|  | 2-3 | 1820 | 21 | 23194.5 | 1.34 (0.86-2.09) | 0.196 | 0.90 (0.57-1.43) | 0.668 |
|  | 3-4 | 1296 | 21 | 16495.09 | 1.88 (1.21-2.94) | 0.005 | 1.09 (0.68-1.75) | 0.715 |
|  | >4 | 1314 | 10 | 16767.69 | 0.88 (0.47-1.66) | 0.703 | 0.43 (0.22-0.84) | 0.013 |
| **Cirrhosis** |  |  |  |  |  |  |  |  |
| **Unsweetened coffee** | 0 | 35528 | 140 | 454818.2 | 1 (Reference) | 1 | 1 (Reference) | 1 |
|  | ≤ 1 | 17627 | 57 | 227172.7 | 0.77 (0.56-1.05) | 0.094 | 0.86 (0.63-1.19) | 0.361 |
|  | 1-2 | 22684 | 57 | 292933.2 | 0.59 (0.43-0.80) | <0.001 | 0.61 (0.44-0.85) | 0.003 |
|  | 2-3 | 18561 | 45 | 239761.1 | 0.56 (0.40-0.78) | <0.001 | 0.55 (0.38-0.79) | 0.001 |
|  | 3-4 | 12583 | 32 | 162723.4 | 0.59 (0.40-0.87) | 0.007 | 0.54 (0.35-0.81) | 0.003 |
|  | >4 | 11022 | 29 | 142147.6 | 0.62 (0.41-0.92) | 0.019 | 0.49 (0.32-0.77) | 0.002 |
| **Sugar-sweetened coffee** | 0 | 35528 | 140 | 454818.2 | 1 (Reference) | 1 | 1 (Reference) | 1 |
|  | ≤ 1 | 6512 | 24 | 83231.59 | 0.74 (0.48-1.15) | 0.178 | 0.75 (0.48-1.17) | 0.199 |
|  | 1-2 | 5963 | 21 | 76420.03 | 0.67 (0.42-1.07) | 0.091 | 0.61 (0.38-1.00) | 0.048 |
|  | 2-3 | 3935 | 14 | 50408.15 | 0.67 (0.38-1.16) | 0.152 | 0.56 (0.32-1.01) | 0.054 |
|  | 3-4 | 2256 | 10 | 28702.13 | 0.82 (0.43-1.56) | 0.550 | 0.61 (0.31-1.20) | 0.154 |
|  | >4 | 2013 | 9 | 25516.68 | 0.87 (0.44-1.72) | 0.691 | 0.54 (0.26-1.12) | 0.097 |
| **Artificially sweetened coffee** | 0 | 35528 | 140 | 454818.2 | 1 (Reference) | 1 | 1 (Reference) | 1 |
|  | ≤ 1 | 1944 | 17 | 24740.1 | 1.87 (1.13-3.10) | 0.015 | 1.32 (0.78-2.21) | 0.298 |
|  | 1-2 | 2205 | 15 | 28235.89 | 1.43 (0.84-2.45) | 0.188 | 0.88 (0.50-1.53) | 0.644 |
|  | 2-3 | 1820 | 6 | 23259.47 | 0.68 (0.30-1.54) | 0.352 | 0.41 (0.17-0.94) | 0.036 |
|  | 3-4 | 1296 | 7 | 16563.53 | 1.08 (0.50-2.31) | 0.843 | 0.57 (0.26-1.27) | 0.169 |
|  | >4 | 1314 | 3 | 16788.24 | 0.46 (0.15-1.45) | 0.187 | 0.20 (0.06-0.66) | 0.008 |
| **Biliary diseases** |  |  |  |  |  |  |  |  |
| **Unsweetened coffee** | 0 | 35528 | 1069 | 449041.9 | 1 (Reference) | 1 | 1 (Reference) | 1 |
|  | ≤ 1 | 17627 | 436 | 224804.1 | 0.76 (0.68-0.85) | <0.001 | 0.87 (0.78-0.97) | 0.016 |
|  | 1-2 | 22684 | 530 | 290025.3 | 0.71 (0.64-0.79) | <0.001 | 0.82 (0.74-0.92) | <0.001 |
|  | 2-3 | 18561 | 459 | 237133 | 0.76 (0.68-0.85) | <0.001 | 0.86 (0.77-0.97) | 0.013 |
|  | 3-4 | 12583 | 344 | 160744.7 | 0.85 (0.75-0.96) | 0.010 | 0.94 (0.82-1.07) | 0.313 |
|  | >4 | 11022 | 303 | 140411.9 | 0.87 (0.77-0.99) | 0.037 | 0.91 (0.80-1.05) | 0.212 |
| **Sugar-sweetened coffee** | 0 | 35528 | 1069 | 449041.9 | 1 (Reference) | 1 | 1 (Reference) | 1 |
|  | ≤ 1 | 6512 | 207 | 82115.53 | 1.04 (0.89-1.20) | 0.637 | 1.09 (0.94-1.27) | 0.266 |
|  | 1-2 | 5963 | 160 | 75586.58 | 0.89 (0.75-1.05) | 0.155 | 0.93 (0.78-1.10) | 0.393 |
|  | 2-3 | 3935 | 108 | 49800.64 | 0.92 (0.75-1.12) | 0.409 | 0.95 (0.77-1.17) | 0.644 |
|  | 3-4 | 2256 | 70 | 28355.41 | 1.06 (0.83-1.35) | 0.662 | 1.05 (0.81-1.35) | 0.730 |
|  | >4 | 2013 | 51 | 25280.97 | 0.90 (0.68-1.19) | 0.462 | 0.85 (0.63-1.14) | 0.275 |
| **Artificially sweetened coffee** | 0 | 35528 | 1069 | 449041.9 | 1 (Reference) | 1 | 1 (Reference) | 1 |
|  | ≤ 1 | 1944 | 77 | 24434.47 | 1.22 (0.97-1.54) | 0.087 | 1.04 (0.82-1.31) | 0.764 |
|  | 1-2 | 2205 | 69 | 27857.8 | 0.96 (0.75-1.23) | 0.763 | 0.79 (0.62-1.01) | 0.065 |
|  | 2-3 | 1820 | 78 | 22839.32 | 1.33 (1.06-1.68) | 0.015 | 1.17 (0.92-1.49) | 0.198 |
|  | 3-4 | 1296 | 58 | 16248.03 | 1.40 (1.08-1.83) | 0.012 | 1.17 (0.89-1.55) | 0.262 |
|  | >4 | 1314 | 54 | 16505.23 | 1.28 (0.97-1.69) | 0.075 | 1.02 (0.76-1.37) | 0.886 |
| **Appendicitis** |  |  |  |  |  |  |  |  |
| **Unsweetened coffee** | 0 | 35528 | 215 | 453723.8 | 1 (Reference) | 1 | 1 (Reference) | 1 |
|  | ≤ 1 | 17627 | 99 | 226624.8 | 0.95 (0.75-1.21) | 0.676 | 0.93 (0.73-1.19) | 0.555 |
|  | 1-2 | 22684 | 115 | 292411.6 | 0.86 (0.68-1.08) | 0.195 | 0.84 (0.66-1.06) | 0.136 |
|  | 2-3 | 18561 | 91 | 239310.8 | 0.83 (0.65-1.07) | 0.145 | 0.80 (0.62-1.04) | 0.097 |
|  | 3-4 | 12583 | 65 | 162381.5 | 0.87 (0.66-1.15) | 0.337 | 0.83 (0.62-1.12) | 0.219 |
|  | >4 | 11022 | 54 | 141864.2 | 0.82 (0.61-1.11) | 0.207 | 0.77 (0.56-1.06) | 0.111 |
| **Sugar-sweetened coffee** | 0 | 35528 | 215 | 453723.8 | 1 (Reference) | 1 | 1 (Reference) | 1 |
|  | ≤ 1 | 6512 | 42 | 83039.75 | 1.09 (0.78-1.52) | 0.605 | 1.07 (0.76-1.51) | 0.685 |
|  | 1-2 | 5963 | 33 | 76295.54 | 0.94 (0.65-1.35) | 0.721 | 0.90 (0.61-1.32) | 0.585 |
|  | 2-3 | 3935 | 19 | 50302.91 | 0.82 (0.51-1.31) | 0.402 | 0.78 (0.48-1.27) | 0.312 |
|  | 3-4 | 2256 | 6 | 28694.53 | 0.45 (0.20-1.02) | 0.056 | 0.43 (0.19-0.98) | 0.045 |
|  | >4 | 2013 | 20 | 25411.97 | 1.69 (1.06-2.68) | 0.026 | 1.51 (0.91-2.51) | 0.110 |
| **Artificially sweetened coffee** | 0 | 35528 | 215 | 453723.8 | 1 (Reference) | 1 | 1 (Reference) | 1 |
|  | ≤ 1 | 1944 | 12 | 24700.1 | 1.05 (0.59-1.88) | 0.873 | 0.98 (0.54-1.76) | 0.942 |
|  | 1-2 | 2205 | 12 | 28182.01 | 0.92 (0.51-1.65) | 0.780 | 0.83 (0.46-1.51) | 0.536 |
|  | 2-3 | 1820 | 8 | 23203.37 | 0.75 (0.37-1.52) | 0.425 | 0.68 (0.33-1.40) | 0.291 |
|  | 3-4 | 1296 | 5 | 16543.41 | 0.66 (0.27-1.61) | 0.366 | 0.58 (0.23-1.44) | 0.239 |
|  | >4 | 1314 | 5 | 16771.77 | 0.65 (0.27-1.58) | 0.345 | 0.54 (0.22-1.36) | 0.193 |
| **Gastrointestinal cancer** |  |  |  |  |  |  |  |  |
| **Unsweetened coffee** | 0 | 35528 | 606 | 453053.7 | 1 (Reference) | 1 | 1 (Reference) | 1 |
|  | ≤ 1 | 17627 | 330 | 226173.5 | 0.95 (0.83-1.09) | 0.479 | 0.98 (0.85-1.12) | 0.741 |
|  | 1-2 | 22684 | 439 | 291566.5 | 0.97 (0.85-1.09) | 0.599 | 0.97 (0.86-1.11) | 0.689 |
|  | 2-3 | 18561 | 366 | 238581.3 | 0.98 (0.86-1.11) | 0.744 | 0.97 (0.85-1.12) | 0.718 |
|  | 3-4 | 12583 | 248 | 161893.1 | 1.00 (0.86-1.16) | 0.997 | 0.97 (0.82-1.13) | 0.670 |
|  | >4 | 11022 | 224 | 141475.3 | 1.07 (0.91-1.24) | 0.419 | 0.99 (0.83-1.17) | 0.868 |
| **Sugar-sweetened coffee** | 0 | 35528 | 606 | 453053.7 | 1 (Reference) | 1 | 1 (Reference) | 1 |
|  | ≤ 1 | 6512 | 129 | 82856.37 | 0.93 (0.77-1.13) | 0.468 | 0.91 (0.74-1.10) | 0.318 |
|  | 1-2 | 5963 | 126 | 75938.69 | 0.96 (0.79-1.16) | 0.676 | 0.89 (0.73-1.09) | 0.279 |
|  | 2-3 | 3935 | 93 | 50108.52 | 1.07 (0.86-1.34) | 0.538 | 0.97 (0.77-1.23) | 0.820 |
|  | 3-4 | 2256 | 45 | 28578.19 | 0.90 (0.66-1.21) | 0.475 | 0.77 (0.56-1.06) | 0.112 |
|  | >4 | 2013 | 60 | 25364.27 | 1.45 (1.11-1.89) | 0.007 | 1.17 (0.88-1.57) | 0.283 |
| **Artificially sweetened coffee** | 0 | 35528 | 606 | 453053.7 | 1 (Reference) | 1 | 1 (Reference) | 1 |
|  | ≤ 1 | 1944 | 66 | 24561.62 | 1.58 (1.22-2.04) | <0.001 | 1.41 (1.09-1.83) | 0.010 |
|  | 1-2 | 2205 | 52 | 28101.98 | 1.08 (0.81-1.43) | 0.604 | 0.92 (0.69-1.24) | 0.601 |
|  | 2-3 | 1820 | 51 | 23078.2 | 1.26 (0.95-1.68) | 0.113 | 1.06 (0.78-1.43) | 0.722 |
|  | 3-4 | 1296 | 32 | 16483.21 | 1.09 (0.76-1.56) | 0.627 | 0.87 (0.60-1.26) | 0.465 |
|  | >4 | 1314 | 42 | 16675.3 | 1.44 (1.05-1.97) | 0.023 | 1.09 (0.78-1.53) | 0.613 |
| **Any** **gastrointestinal diseases** |  |  |  |  |  |  |  |  |
| **Unsweetened coffee** | 0 | 35528 | 7365 | 410564.7 | 1 (Reference) | 1 | 1 (Reference) | 1 |
|  | ≤ 1 | 17627 | 3465 | 206408.2 | 0.86 (0.82-0.89) | <0.001 | 0.94 (0.90-0.98) | 0.002 |
|  | 1-2 | 22684 | 4155 | 268498.8 | 0.79 (0.76-0.82) | <0.001 | 0.87 (0.83-0.90) | <0.001 |
|  | 2-3 | 18561 | 3335 | 220416.3 | 0.77 (0.74-0.80) | <0.001 | 0.84 (0.81-0.88) | <0.001 |
|  | 3-4 | 12583 | 2341 | 148954.6 | 0.81 (0.77-0.85) | <0.001 | 0.88 (0.83-0.92) | <0.001 |
|  | >4 | 11022 | 2054 | 130379.4 | 0.83 (0.79-0.87) | <0.001 | 0.86 (0.82-0.91) | <0.001 |
| **Sugar-sweetened coffee** | 0 | 35528 | 7365 | 410564.7 | 1 (Reference) |  | 1 (Reference) | 1 |
|  | ≤ 1 | 6512 | 1417 | 74739.54 | 0.97 (0.92-1.03) | 0.343 | 1.00 (0.94-1.06) | 0.891 |
|  | 1-2 | 5963 | 1240 | 68959.56 | 0.92 (0.87-0.98) | 0.007 | 0.94 (0.88-1.00) | 0.049 |
|  | 2-3 | 3935 | 814 | 45582.15 | 0.92 (0.85-0.99) | 0.019 | 0.93 (0.86-1.01) | 0.072 |
|  | 3-4 | 2256 | 457 | 26031.42 | 0.90 (0.82-0.99) | 0.026 | 0.89 (0.81-0.98) | 0.021 |
|  | >4 | 2013 | 413 | 23075.99 | 0.96 (0.87-1.06) | 0.433 | 0.90 (0.81-1.00) | 0.052 |
| **Artificially sweetened coffee** | 0 | 35528 | 7365 | 410564.7 | 1 (Reference) |  | 1 (Reference) | 1 |
|  | ≤ 1 | 1944 | 478 | 21877.63 | 1.08 (0.98-1.18) | 0.109 | 0.98 (0.89-1.07) | 0.632 |
|  | 1-2 | 2205 | 518 | 25128.51 | 1.01 (0.93-1.11) | 0.766 | 0.90 (0.82-0.99) | 0.024 |
|  | 2-3 | 1820 | 424 | 20753.79 | 1.00 (0.90-1.10) | 0.934 | 0.92 (0.83-1.02) | 0.117 |
|  | 3-4 | 1296 | 312 | 14675.76 | 1.04 (0.93-1.16) | 0.533 | 0.93 (0.83-1.05) | 0.252 |
|  | >4 | 1314 | 330 | 14919.57 | 1.08 (0.97-1.21) | 0.162 | 0.93 (0.83-1.05) | 0.237 |

Note: Basic model: adjusted for age (continuous), sex and ethnicity (white or other). Multivariable model: adjusted for variables in basic model plus body mass index ((<25, ≥25 & <30, and ≥30 kg/m^2^), basal metabolic rate (continuous), Townsend deprivation index (continuous), Current employment status (work, retired, or other), education level (degree or no degree), smoking status (current, former, or never), pack-years of smoking (continuous), physical activity level (low, moderate, or high), healthy sleep pattern (yes or no), vitamin use (yes or no), mineral and other dietary supplements use (yes or no), NSAIDs use (yes or no), PPI (yes or no), INFLA-score (continuous), family history of CVD disease (yes or no), family history of cancer (yes or no), number of long-term conditions (none, one, two, three and more), and intake of total energy, total sugar, tea, and AHEI score. HR, hazard ratio; CI, confidence interval; BMI, NSAIDs, Nonsteroidal anti-inflammatory drugs; AHEI, Alternative Healthy Eating Index, GI, gastrointestinal; GERD, Gastroesophageal reflux disease; IBS, Irritable bowel syndrome; NAFLD, Non-alcoholic fatty liver disease.

# Supplementary Table 8. Association of polygenic risk score (PRS) with the risk of GERD, peptic ulcer, diverticulum, NAFLD, and cirrhosis.

| **Genetic risk scores** | **Number of Participants** | **Case/person-years** | **HR (95% CI)** | **HR per SD (95% CI)** |
| --- | --- | --- | --- | --- |
| **GERD** |  |  |  |  |
| Unsweetened coffee |  |  |  |  |
| Low genetic risk | 41892 | 2267/528240 | 1 (Reference) | 1 (Reference) |
| High genetic risk | 41579 | 2573/522416 | 1.12 (1.05-1.18) | 1.06 (1.03-1.09) |
| Sugar-sweetened coffee |  |  |  |  |
| Low genetic risk | 19467 | 1203/243195 | 1 (Reference) | 1 (Reference) |
| High genetic risk | 19248 | 1350/239127 | 1.11 (1.02-1.20) | 1.05 (1.01-1.09) |
| Artificially sweetened coffee |  |  |  |  |
| Low genetic risk | 15425 | 943/192894 | 1 (Reference) | 1 (Reference) |
| High genetic risk | 15170 | 1086/188624 | 1.14 (1.04-1.24) | 1.09 (1.04-1.14) |
| **Peptic ulcer** |  |  |  |  |
| Unsweetened coffee |  |  |  |  |
| Low genetic risk | 54437 | 569/697895 | 1 (Reference) | 1 (Reference) |
| High genetic risk | 52622 | 622/675900 | 1.14 (1.02-1.28) | 1.1 (1.03-1.16) |
| Sugar-sweetened coffee |  |  |  |  |
| Low genetic risk | 25324 | 314/322059 | 1 (Reference) | 1 (Reference) |
| High genetic risk | 25275 | 365/322385 | 1.17 (1.01-1.36) | 1.11 (1.03-1.20) |
| Artificially sweetened coffee |  |  |  |  |
| Low genetic risk | 19882 | 259/252979 | 1 (Reference) | 1 (Reference) |
| High genetic risk | 19824 | 311/252903 | 1.22 (1.03-1.44) | 1.07 (0.99-1.17) |
| **Diverticulum** |  |  |  |  |
| Unsweetened coffee |  |  |  |  |
| Low genetic risk | 34561 | 2457/431847 | 1 (Reference) | 1 (Reference) |
| High genetic risk | 32513 | 3157/400785 | 1.42 (1.34-1.49) | 1.25 (1.22-1.29) |
| Sugar-sweetened coffee |  |  |  |  |
| Low genetic risk | 15973 | 1221/197949 | 1 (Reference) | 1 (Reference) |
| High genetic risk | 15260 | 1601/186262 | 1.45 (1.34-1.56) | 1.28 (1.23-1.33) |
| Artificially sweetened coffee |  |  |  |  |
| Low genetic risk | 12615 | 980/156177 | 1 (Reference) | 1 (Reference) |
| High genetic risk | 12036 | 1264/146879 | 1.44 (1.32-1.56) | 1.31 (1.24-1.37) |
| **NAFLD** |  |  |  |  |
| Unsweetened coffee |  |  |  |  |
| Low genetic risk | 32603 | 125/420000 | 1 (Reference) | 1 (Reference) |
| High genetic risk | 32463 | 236/417584 | 1.93 (1.56-2.41) | 1.39 (1.25-1.54) |
| Sugar-sweetened coffee |  |  |  |  |
| Low genetic risk | 15235 | 80/194920 | 1 (Reference) | 1 (Reference) |
| High genetic risk | 15219 | 149/194586 | 1.95 (1.48-2.56) | 1.31 (1.14-1.50) |
| Artificially sweetened coffee |  |  |  |  |
| Low genetic risk | 11972 | 74/153269 | 1 (Reference) | 1 (Reference) |
| High genetic risk | 11923 | 124/152427 | 1.68 (1.26-2.25) | 1.33 (1.16-1.54) |
| **Cirrhosis** |  |  |  |  |
| Unsweetened coffee |  |  |  |  |
| Low genetic risk | 53493 | 130/689702 | 1 (Reference) | 1 (Reference) |
| High genetic risk | 53474 | 194/688444 | 1.51 (1.21-1.89) | 1.30 (1.18-1.44) |
| Sugar-sweetened coffee |  |  |  |  |
| Low genetic risk | 25298 | 88/323999 | 1 (Reference) | 1 (Reference) |
| High genetic risk | 25292 | 112/323734 | 1.28 (0.97-1.69) | 1.24 (1.09-1.42) |
| Artificially sweetened coffee |  |  |  |  |
| Low genetic risk | 19851 | 68/254316 | 1 (Reference) | 1 (Reference) |
| High genetic risk | 19812 | 106/253587 | 1.62 (1.19-2.20) | 1.41 (1.23-1.62) |

Estimates are hazard ratios (95% CIs) from multivariable Cox proportional hazard models adjusted for age (continuous), gender, body mass index ((<25, ≥ 25 & < 30, and ≥ 30 kg/m^2^), ethnicity (white or other), Townsend deprivation index (continuous), Current employment status (work, retired, or other), education level (degree or no degree), smoking status (current, former, or never), pack-years of smoking (continuous), physical activity level (low, moderate, or high), healthy sleep pattern (yes or no), hot drink temperature (very hot, hot, warm, or other), vitamin use (yes or no), mineral and other dietary supplements use (yes or no), NSAIDs use (yes or no), PPI use (yes or no), INFLA-score (continuous), family history of CVD disease (yes or no), family history of cancer (yes or no), number of long-term conditions (none, one, two, three and more), and intake of total energy, total sugar, total tea, AHEI score, genotyping batch, and the first 10 genetic principal components. HR, hazard ratio; CI, confidence interval; NSAIDs, Nonsteroidal anti-inflammatory drugs; AHEI, Alternative Healthy Eating Index, GERD, Gastroesophageal reflux disease; NAFLD, Non-alcoholic fatty liver disease.

# Supplementary Table 9. Association of coffee consumption with the incidence of GERD, peptic ulcer, diverticulum, NAFLD, and cirrhosis.

| **Outcome** | **Nonconsumers** | **Coffee intake, drinks/d** | | | | |
| --- | --- | --- | --- | --- | --- | --- |
|  |  | **≤ 1** | **1-2** | **2-3** | **3-4** | **>4** |
| **GERD** |  |  |  |  |  |  |
| Unsweetened coffee |  |  |  |  |  |  |
| Number of participants | 24411 | 12471 | 16296 | 13313 | 9059 | 7921 |
| Cases/person-years | 1578/304722 | 724/156894 | 855/206322 | 694/168437 | 530/114570 | 459/99710 |
| Multivariable model* | 1 (Reference) | 0.95 (0.87-1.04) | 0.87 (0.80-0.95) | 0.87 (0.79-0.96) | 0.98 (0.88-1.09) | 0.97 (0.87-1.09) |
| Sugar-sweetened coffee |  |  |  |  |  |  |
| Number of participants | 24411 | 4392 | 4110 | 2736 | 1634 | 1432 |
| Cases/person-years | 1578/304722 | 307/54534 | 264/51256 | 204/33973 | 116/20122 | 84/17715 |
| Multivariable model* | 1 (Reference) | 1.05 (0.93-1.19) | 0.96 (0.83-1.10) | 1.13 (0.97-1.32) | 1.06 (0.87-1.30) | 0.85 (0.67-1.07) |
| Artificially sweetened coffee |  |  |  |  |  |  |
| Number of participants | 24411 | 1390 | 1583 | 1286 | 941 | 984 |
| Cases/person-years | 1578/304722 | 107/17179 | 123/19674 | 88/15995 | 56/11784 | 77/12164 |
| Multivariable model* | 1 (Reference) | 0.99 (0.81-1.21) | 0.95 (0.78-1.15) | 0.91 (0.73-1.14) | 0.74 (0.56-0.97) | 0.96 (0.75-1.23) |
| **Peptic ulcer** |  |  |  |  |  |  |
| Unsweetened coffee |  |  |  |  |  |  |
| Number of participants | 31933 | 15969 | 20702 | 16896 | 11468 | 10091 |
| Cases/person-years | 441/406906 | 159/205181 | 209/266495 | 153/217780 | 124/147780 | 105/129653 |
| Multivariable model* | 1 (Reference) | 0.73 (0.61-0.88) | 0.73 (0.61-0.86) | 0.64 (0.53-0.78) | 0.75 (0.61-0.93) | 0.68 (0.54-0.86) |
| Sugar-sweetened coffee |  |  |  |  |  |  |
| Number of participants | 31933 | 5845 | 5391 | 3557 | 2043 | 1830 |
| Cases/person-years | 441/406906 | 72/74397 | 63/68764 | 54/45292 | 23/25919 | 26/23165 |
| Multivariable model* | 1 (Reference) | 0.78 (0.60-1.00) | 0.72 (0.55-0.95) | 0.92 (0.68-1.24) | 0.66 (0.43-1.02) | 0.78 (0.51-1.19) |
| Artificially sweetened coffee |  |  |  |  |  |  |
| Number of participants | 31933 | 1729 | 1985 | 1655 | 1180 | 1224 |
| Cases/person-years | 441/406906 | 28/22025 | 39/25350 | 24/21064 | 16/15012 | 22/15524 |
| Multivariable model* | 1 (Reference) | 0.94 (0.64-1.39) | 1.12 (0.80-1.57) | 0.86 (0.56-1.32) | 0.75 (0.45-1.27) | 0.97 (0.62-1.54) |
| **Diverticulum** |  |  |  |  |  |  |
| Unsweetened coffee |  |  |  |  |  |  |
| Number of participants | 19715 | 10066 | 12978 | 10720 | 7265 | 6330 |
| Cases/person-years | 1725/242782 | 877/124976 | 1065/161639 | 873/133620 | 561/90813 | 513/78802 |
| Multivariable model* | 1 (Reference) | 0.93 (0.86-1.01) | 0.86 (0.79-0.93) | 0.85 (0.78-0.93) | 0.81 (0.73-0.90) | 0.83 (0.75-0.93) |
| Sugar-sweetened coffee |  |  |  |  |  |  |
| Number of participants | 19715 | 3572 | 3338 | 2213 | 1273 | 1122 |
| Cases/person-years | 1725/242782 | 346/43909 | 325/41009 | 211/27240 | 112/15651 | 103/13621 |
| Multivariable model* | 1 (Reference) | 0.98 (0.87-1.10) | 0.97 (0.86-1.10) | 0.97 (0.83-1.13) | 0.85 (0.69-1.03) | 0.94 (0.76-1.16) |
| Artificially sweetened coffee |  |  |  |  |  |  |
| Number of participants | 19715 | 1080 | 1235 | 1091 | 743 | 787 |
| Cases/person-years | 1725/242782 | 110/13240 | 124/15151 | 113/13245 | 90/8978 | 82/9659 |
| Multivariable model* | 1 (Reference) | 0.89 (0.73-1.08) | 0.87 (0.72-1.04) | 0.9 (0.74-1.10) | 0.97 (0.77-1.21) | 0.87 (0.69-1.10) |
| **NAFLD** |  |  |  |  |  |  |
| Unsweetened coffee |  |  |  |  |  |  |
| Number of participants | 19091 | 9694 | 12588 | 10455 | 7050 | 6188 |
| Cases/person-years | 141/244383 | 49/124756 | 49/162477 | 43/135001 | 31/91106 | 48/79861 |
| Multivariable model* | 1 (Reference) | 0.81 (0.58-1.13) | 0.59 (0.42-0.83) | 0.59 (0.41-0.85) | 0.55 (0.37-0.84) | 0.89 (0.61-1.28) |
| Sugar-sweetened coffee |  |  |  |  |  |  |
| Number of participants | 19091 | 3460 | 3307 | 2177 | 1292 | 1127 |
| Cases/person-years | 141/244383 | 27/44186 | 26/42435 | 14/27831 | 13/16340 | 8/14331 |
| Multivariable model* | 1 (Reference) | 1.06 (0.70-1.62) | 1.01 (0.65-1.57) | 0.79 (0.44-1.40) | 1.05 (0.57-1.92) | 0.69 (0.32-1.47) |
| Artificially sweetened coffee |  |  |  |  |  |  |
| Number of participants | 19091 | 1063 | 1251 | 1019 | 726 | 745 |
| Cases/person-years | 141/244383 | 13/13599 | 18/16015 | 10/13005 | 12/9215 | 4/9480 |
| Multivariable model* | 1 (Reference) | 1.09 (0.61-1.94) | 1.20 (0.71-2.01) | 0.91 (0.47-1.78) | 1.33 (0.71-2.50) | 0.37 (0.13-1.04) |
| **Cirrhosis** |  |  |  |  |  |  |
| Unsweetened coffee |  |  |  |  |  |  |
| Number of participants | 31929 | 15972 | 20604 | 16907 | 11488 | 10067 |
| Cases/person-years | 130/409076 | 53/206024 | 50/266182 | 40/218384 | 27/148585 | 24/129895 |
| Multivariable model* | 1 (Reference) | 0.83 (0.60-1.16) | 0.56 (0.40-0.79) | 0.5 (0.34-0.74) | 0.46 (0.29-0.71) | 0.41 (0.26-0.66) |
| Sugar-sweetened coffee |  |  |  |  |  |  |
| Number of participants | 31929 | 5828 | 5407 | 3586 | 2030 | 1810 |
| Cases/person-years | 130/409076 | 23/74576 | 20/69290 | 11/45974 | 9/25825 | 7/22991 |
| Multivariable model* | 1 (Reference) | 0.75 (0.47-1.18) | 0.62 (0.38-1.02) | 0.46 (0.24-0.88) | 0.59 (0.29-1.20) | 0.44 (0.2-1.00) |
| Artificially sweetened coffee |  |  |  |  |  |  |
| Number of participants | 31929 | 1758 | 1975 | 1625 | 1179 | 1197 |
| Cases/person-years | 130/409076 | 15/22398 | 14/25294 | 6/20797 | 6/15043 | 3/15295 |
| Multivariable model* | 1 (Reference) | 1.35 (0.78-2.35) | 0.94 (0.52-1.67) | 0.45 (0.19-1.05) | 0.54 (0.23-1.27) | 0.23 (0.07-0.74) |

* Multivariable model, estimates are hazard ratios (95% CIs) from multivariable Cox proportional hazard models adjusted for age (continuous), sex (male or female), body mass index (<25, ≥ 25 & < 30, and ≥ 30 km/m^2^), ethnicity (white or other), Townsend deprivation index (continuous), current employment status (work, retired or other), education level (degree or no degree), smoking status (current, former, or never), pack-years of smoking (continuous), physical activity level (low, moderate, or high), healthy sleep pattern (yes or no), hot drink temperature (very hot, hot, warm, or other), vitamin use (yes or no), mineral and other dietary supplements use (yes or no), NSAIDs use (yes or no), PPI use (yes or no), INFLA-score (continuous), family history of CVD disease (yes or no), family history of cancer (yes or no), number of long-term conditions (none, one, two, three and more), and intake of total energy, total sugar, tea, and AHEI score, and PRS of diseases, genotyping batch, and the first 10 genetic principal components. HR, hazard ratio; CI, confidence interval; NSAIDs, Nonsteroidal anti-inflammatory drugs; AHEI, Alternative Healthy Eating Index, GERD, Gastroesophageal reflux disease; NAFLD, Non-alcoholic fatty liver disease.

# Supplementary Table 10. Associations of coffee consumption with incident GI diseases after excluding participants with missing covariate values.

| **Outcome** | **Nonconsumers** | **Coffee intake, drinks/d** | | | | |
| --- | --- | --- | --- | --- | --- | --- |
|  |  | **≤ 1** | **1-2** | **2-3** | **3-4** | **> 4** |
| **Unsweetened coffee** | | | | | | |
| **Number of participants** | 19744 | 9690 | 12596 | 10404 | 7068 | 6142 |
| **Barrett’s oesophagus** |  |  |  |  |  |  |
| Cases/person-years | 161/253088 | 74/124895 | 88/162828 | 66/134397 | 44/91538 | 43/79174 |
| Multivariable model* | 1 (Reference) | 0.99 (0.74-1.31) | 0.92 (0.7-1.21) | 0.85 (0.63-1.15) | 0.85 (0.59-1.21) | 0.94 (0.65-1.36) |
| **GERD** | | | | | | |
| Cases/person-years | 1203/247499 | 552/122198 | 622/159981 | 525/131905 | 368/89827 | 328/77573 |
| Multivariable model* | 1 (Reference) | 0.98 (0.88-1.09) | **0.86 (0.78-0.95)** | **0.89 (0.79-0.99)** | 0.93 (0.82-1.06) | 0.94 (0.83-1.08) |
| **Gastritis and duodenitis** | | | | | | |
| Cases/person-years | 1231/246348 | 569/121794 | 638/159341 | 486/131793 | 331/89840 | 280/77767 |
| Multivariable model* | 1 (Reference) | 0.97 (0.87-1.07) | **0.84 (0.76-0.93)** | **0.78 (0.69-0.87)** | **0.78 (0.69-0.89)** | **0.75 (0.65-0.86)** |
| **Celiac disease** | | | | | | |
| Cases/person-years | 69/253480 | 32/125123 | 40/163019 | 24/134604 | 17/91671 | 10/79337 |
| Multivariable model* | 1 (Reference) | 0.93 (0.60-1.42) | 0.93 (0.62-1.40) | 0.7 (0.43-1.14) | 0.76 (0.43-1.33) | 0.53 (0.27-1.07) |
| **Peptic ulcer** |  |  |  |  |  |  |
| Cases/person-years | 254/252388 | 97/124729 | 118/162555 | 93/134240 | 71/91355 | 61/79081 |
| Multivariable model* | 1 (Reference) | 0.81 (0.64-1.03) | **0.75 (0.6-0.94)** | **0.7 (0.54-0.90)** | 0.77 (0.58-1.02) | **0.73 (0.54-0.99)** |
| **Crohn’s disease** |  |  |  |  |  |  |
| Cases/person-years | 32/253743 | 12/125241 | 16/163174 | 16/134662 | 6/91722 | 14/79340 |
| Multivariable model* | 1 (Reference) | 0.85 (0.43-1.67) | 0.88 (0.47-1.65) | 1.06 (0.56-2.01) | 0.58 (0.23-1.44) | 1.49 (0.74-3.00) |
| **Ulcerative colitis** |  |  |  |  |  |  |
| Cases/person-years | 83/253488 | 31/125107 | 34/163052 | 34/134552 | 27/91621 | 17/79309 |
| Multivariable model* | 1 (Reference) | 0.85 (0.56-1.31) | 0.72 (0.47-1.09) | 0.85 (0.55-1.31) | 0.96 (0.60-1.53) | 0.67 (0.38-1.17) |
| **IBS** |  |  |  |  |  |  |
| Cases/person-years | 209/252873 | 106/124774 | 114/162663 | 93/134248 | 66/91469 | 53/79145 |
| Multivariable model* | 1 (Reference) | 1.05 (0.83-1.34) | 0.9 (0.71-1.14) | 0.9 (0.69-1.16) | 0.96 (0.72-1.29) | 0.86 (0.62-1.19) |
| **Diverticulum** |  |  |  |  |  |  |
| Cases/person-years | 1670/244275 | 809/120727 | 976/157720 | 806/130251 | 566/88434 | 493/76769 |
| Multivariable model* | 1 (Reference) | 0.93 (0.85-1.01) | **0.85 (0.78-0.92)** | **0.84 (0.76-0.91)** | **0.86 (0.78-0.95)** | **0.84 (0.75-0.94)** |
| **Pancreatitis** |  |  |  |  |  |  |
| Cases/person-years | 86/253469 | 28/125128 | 44/163043 | 31/134590 | 19/91664 | 25/79260 |
| Multivariable model* | 1 (Reference) | 0.74 (0.48-1.15) | 0.91 (0.62-1.33) | 0.78 (0.50-1.20) | 0.7 (0.41-1.18) | 1.01 (0.62-1.66) |
| **NAFLD** |  |  |  |  |  |  |
| Cases/person-years | 152/253354 | 58/124971 | 55/163000 | 42/134577 | 35/91587 | 37/79265 |
| Multivariable model* | 1 (Reference) | 0.99 (0.72-1.35) | **0.69 (0.50-0.96)** | **0.62 (0.43-0.89)** | 0.72 (0.48-1.07) | 0.77 (0.52-1.15) |
| **Cirrhosis** |  |  |  |  |  |  |
| Cases/person-years | 75/253737 | 29/125227 | 32/163119 | 21/134666 | 17/91725 | 17/79365 |
| Multivariable model* | 1 (Reference) | 0.80 (0.52-1.25) | **0.6 (0.39-0.94)** | **0.45 (0.27-0.75)** | **0.48 (0.27-0.84)** | **0.50 (0.28-0.89)** |
| **Biliary diseases** |  |  |  |  |  |  |
| Cases/person-years | 560/250705 | 249/123833 | 292/161481 | 251/133221 | 202/90537 | 156/78439 |
| Multivariable model* | 1 (Reference) | 0.95 (0.82-1.11) | **0.85 (0.73-0.99)** | 0.87 (0.74-1.02) | 1.02 (0.86-1.21) | 0.87 (0.72-1.06) |
| **Appendicitis** |  |  |  |  |  |  |
| Cases/person-years | 114/253166 | 53/124882 | 72/162821 | 48/134382 | 36/91508 | 28/79217 |
| Multivariable model* | 1 (Reference) | 0.96 (0.69-1.34) | 1.01 (0.74-1.38) | 0.82 (0.57-1.17) | 0.90 (0.61-1.35) | 0.80 (0.52-1.25) |
| **Gastrointestinal cancer** |  |  |  |  |  |  |
| Cases/person-years | 317/252780 | 183/124629 | 243/162366 | 207/133924 | 131/91240 | 120/79053 |
| Multivariable model* | 1 (Reference) | 1.05 (0.87-1.27) | 1.04 (0.87-1.24) | 1.06 (0.88-1.28) | 0.98 (0.79-1.22) | 1.02 (0.81-1.29) |
| **Any gastrointestinal diseases** |  |  |  |  |  |  |
| Cases/person-years | 3994/229780 | 1868/114048 | 2248/149886 | 1841/123948 | 1300/84094 | 1104/73071 |
| Multivariable model* | 1 (Reference) | **0.94 (0.89-1.00)** | **0.86 (0.82-0.91)** | **0.85 (0.80-0.90)** | **0.89 (0.83-0.95)** | **0.84 (0.79-0.91)** |
| **Sugar-sweetened coffee** |  |  |  |  |  |  |
| **Number of participants** | 19744 | 3450 | 3164 | 2135 | 1270 | 1138 |
| **Barrett’s oesophagus** |  |  |  |  |  |  |
| Cases/person-years | 161/253088 | 43/43988 | 18/40505 | 21/27347 | 7/16140 | 17/14351 |
| Multivariable model* | 1 (Reference) | 1.32 (0.93-1.87) | 0.61 (0.37-1.01) | 1.06 (0.66-1.72) | 0.56 (0.26-1.22) | 1.6 (0.92-2.80) |
| **GERD** |  |  |  |  |  |  |
| Cases/person-years | 1203/247499 | 240/42826 | 185/39540 | 149/26668 | 80/15710 | 68/14122 |
| Multivariable model* | 1 (Reference) | 1.12 (0.97-1.29) | 0.95 (0.81-1.11) | 1.13 (0.94-1.35) | 1.01 (0.80-1.28) | 0.94 (0.73-1.22) |
| **Gastritis and duodenitis** |  |  |  |  |  |  |
| Cases/person-years | 1231/246348 | 201/42897 | 181/39443 | 110/26776 | 69/15728 | 73/13965 |
| Multivariable model* | 1 (Reference) | 0.88 (0.76-1.03) | 0.88 (0.75-1.03) | **0.78 (0.64-0.96)** | 0.82 (0.64-1.06) | 0.95 (0.74-1.22) |
| **Celiac disease** |  |  |  |  |  |  |
| Cases/person-years | 69/253480 | 12/44146 | 9/40556 | 3/27429 | 0/16179 | 4/14424 |
| Multivariable model* | 1 (Reference) | 1.02 (0.55-1.92) | 0.95 (0.46-1.95) | 0.49 (0.15-1.59) | 0 (0-Inf) | 1.53 (0.52-4.46) |
| **Peptic ulcer** |  |  |  |  |  |  |
| Cases/person-years | 254/252388 | 39/44008 | 29/40393 | 26/27294 | 14/16087 | 16/14407 |
| Multivariable model* | 1 (Reference) | 0.75 (0.53-1.06) | 0.59 (0.39-0.87) | 0.74 (0.48-1.14) | 0.65 (0.37-1.14) | 0.75 (0.43-1.29) |
| **Crohn’s disease** |  |  |  |  |  |  |
| Cases/person-years | 32/253743 | 7/44181 | 11/40536 | 3/27426 | 2/16162 | 2/14443 |
| Multivariable model* | 1 (Reference) | 1.11 (0.48-2.57) | 1.8 (0.85-3.78) | 0.7 (0.20-2.40) | 0.73 (0.17-3.25) | 0.79 (0.17-3.65) |
| **Ulcerative colitis** |  |  |  |  |  |  |
| Cases/person-years | 83/253488 | 9/44180 | 13/40550 | 14/27345 | 1/16174 | 6/14400 |
| Multivariable model* | 1 (Reference) | 0.59 (0.29-1.19) | 0.86 (0.46-1.59) | 1.32 (0.71-2.45) | 0.14 (0.02-1.05) | 0.86 (0.35-2.14) |
| **IBS** |  |  |  |  |  |  |
| Cases/person-years | 209/252873 | 20/44100 | 28/40452 | 16/27362 | 11/16112 | 11/14401 |
| Multivariable model* | 1 (Reference) | **0.58 (0.36-0.92)** | 0.96 (0.64-1.45) | 0.83 (0.49-1.41) | 1.00 (0.53-1.87) | 1.15 (0.60-2.19) |
| **Diverticulum** |  |  |  |  |  |  |
| Cases/person-years | 1670/244275 | 324/42455 | 280/39109 | 186/26355 | 107/15614 | 120/13756 |
| Multivariable model* | 1 (Reference) | 0.98 (0.86-1.10) | 0.88 (0.77-1.01) | 0.86 (0.73-1.01) | **0.78 (0.64-0.96)** | 1.00 (0.82-1.22) |
| **Pancreatitis** |  |  |  |  |  |  |
| Cases/person-years | 86/253469 | 20/44141 | 13/40538 | 4/27429 | 5/16156 | 4/14438 |
| Multivariable model* | 1 (Reference) | 1.31 (0.79-2.17) | 0.88 (0.48-1.63) | 0.39 (0.14-1.08) | 0.76 (0.30-1.95) | 0.63 (0.22-1.82) |
| **NAFLD** |  |  |  |  |  |  |
| Cases/person-years | 152/253354 | 33/44058 | 19/40533 | 9/27413 | 9/16147 | 12/14404 |
| Multivariable model* | 1 (Reference) | 1.34 (0.91-1.98) | 0.79 (0.48-1.30) | 0.54 (0.27-1.08) | 0.81 (0.40-1.64) | 1.13 (0.59-2.16) |
| **Cirrhosis** |  |  |  |  |  |  |
| Cases/person-years | 75/253737 | 11/44200 | 9/40593 | 9/27434 | 6/16164 | 6/14437 |
| Multivariable model* | 1 (Reference) | 0.65 (0.34-1.24) | **0.47 (0.23-0.97)** | 0.64 (0.30-1.34) | 0.62 (0.26-1.50) | 0.63 (0.25-1.58) |
| **Biliary diseases** |  |  |  |  |  |  |
| Cases/person-years | 560/250705 | 100/43636 | 77/40188 | 56/27102 | 43/15953 | 31/14286 |
| Multivariable model* | 1 (Reference) | 1.07 (0.86-1.33) | 0.91 (0.71-1.17) | 0.97 (0.72-1.29) | 1.23 (0.88-1.70) | 0.95 (0.65-1.41) |
| **Appendicitis** |  |  |  |  |  |  |
| Cases/person-years | 114/253166 | 21/44103 | 14/40539 | 10/27378 | 4/16162 | 12/14377 |
| Multivariable model* | 1 (Reference) | 1.06 (0.66-1.71) | 0.75 (0.42-1.33) | 0.79 (0.40-1.55) | 0.54 (0.19-1.50) | 1.67 (0.86-3.27) |
| **Gastrointestinal cancer** |  |  |  |  |  |  |
| Cases/person-years | 317/252780 | 77/43955 | 68/40323 | 49/27252 | 24/16083 | 31/14361 |
| Multivariable model* | 1 (Reference) | 1.06 (0.82-1.37) | 0.94 (0.71-1.24) | 0.97 (0.71-1.34) | 0.75 (0.49-1.16) | 1.05 (0.70-1.57) |
| **Any gastrointestinal diseases** |  |  |  |  |  |  |
| Cases/person-years | 3994/229780 | 743/39710 | 642/36738 | 437/24832 | 249/14694 | 238/12999 |
| Multivariable model* | 1 (Reference) | 1.01 (0.93-1.10) | 0.93 (0.85-1.02) | 0.93 (0.84-1.03) | 0.87 (0.76-0.99) | 0.93 (0.81-1.07) |
| **Artificially sweetened coffee** |  |  |  |  |  |  |
| **Number of participants** | 19744 | 1023 | 1154 | 982 | 724 | 725 |
| **Barrett’s oesophagus** |  |  |  |  |  |  |
| Cases/person-years | 161/253088 | 19/12987 | 7/14789 | 6/12607 | 9/9249 | 8/9229 |
| Multivariable model* | 1 (Reference) | 1.76 (1.08-2.87) | 0.57 (0.26-1.23) | 0.63 (0.27-1.46) | 1.32 (0.65-2.69) | 1.15 (0.54-2.47) |
| **GERD** |  |  |  |  |  |  |
| Cases/person-years | 1203/247499 | 88/12640 | 86/14369 | 66/12282 | 46/9044 | 52/9035 |
| Multivariable model* | 1 (Reference) | 1.17 (0.94-1.45) | 0.97 (0.77-1.21) | 0.92 (0.71-1.19) | 0.89 (0.66-1.21) | 0.93 (0.69-1.25) |
| **Gastritis and duodenitis** |  |  |  |  |  |  |
| Cases/person-years | 1231/246348 | 87/12538 | 86/14347 | 56/12345 | 54/8992 | 58/8939 |
| Multivariable model* | 1 (Reference) | 1.16 (0.93-1.45) | 0.96 (0.76-1.20) | 0.77 (0.58-1.01) | 1 (0.75-1.32) | 1.03 (0.78-1.37) |
| **Celiac disease** |  |  |  |  |  |  |
| Cases/person-years | 69/253480 | 6/13055 | 0/14838 | 1/12633 | 1/9300 | 1/9272 |
| Multivariable model* | 1 (Reference) | 2.09 (0.89-4.88) | 0 (0-Inf) | 0.44 (0.06-3.24) | 0.71 (0.10-5.22) | 0.8 (0.11-6.03) |
| **Peptic ulcer** |  |  |  |  |  |  |
| Cases/person-years | 254/252388 | 16/13007 | 20/14745 | 14/12573 | 8/9261 | 14/9192 |
| Multivariable model* | 1 (Reference) | 0.98 (0.59-1.64) | 1.01 (0.63-1.62) | 0.89 (0.51-1.55) | 0.66 (0.32-1.36) | 1.1 (0.61-1.96) |
| **Crohn’s disease** |  |  |  |  |  |  |
| Cases/person-years | 32/253743 | 2/13076 | 1/14831 | 2/12621 | 0/9304 | 1/9271 |
| Multivariable model* | 1 (Reference) | 1.33 (0.31-5.7) | 0.52 (0.07-3.98) | 1.27 (0.28-5.66) | 0 (0-Inf) | 0.66 (0.08-5.42) |
| **Ulcerative colitis** |  |  |  |  |  |  |
| Cases/person-years | 83/253488 | 3/13065 | 7/14805 | 5/12603 | 2/9290 | 1/9272 |
| Multivariable model* | 1 (Reference) | 0.66 (0.21-2.13) | 1.32 (0.59-2.95) | 1.12 (0.44-2.88) | 0.57 (0.13-2.43) | 0.28 (0.04-2.09) |
| **IBS** |  |  |  |  |  |  |
| Cases/person-years | 209/252873 | 15/13028 | 15/14770 | 14/12557 | 10/9265 | 8/9227 |
| Multivariable model* | 1 (Reference) | 1.14 (0.67-1.94) | 0.95 (0.55-1.62) | 1.12 (0.64-1.96) | 1.08 (0.56-2.09) | 0.83 (0.39-1.74) |
| **Diverticulum** |  |  |  |  |  |  |
| Cases/person-years | 1670/244275 | 112/12495 | 118/14155 | 89/12112 | 70/8856 | 82/8838 |
| Multivariable model* | 1 (Reference) | 0.99 (0.82-1.21) | 0.86 (0.71-1.05) | 0.77 (0.62-0.96) | 0.80 (0.63-1.03) | 0.89 (0.71-1.13) |
| **Pancreatitis** |  |  |  |  |  |  |
| Cases/person-years | 86/253469 | 4/13078 | 8/14800 | 6/12609 | 1/9292 | 5/9250 |
| Multivariable model* | 1 (Reference) | 0.64 (0.23-1.77) | 1.02 (0.48-2.17) | 0.96 (0.40-2.27) | 0.17 (0.02-1.24) | 0.82 (0.31-2.17) |
| **NAFLD** |  |  |  |  |  |  |
| Cases/person-years | 152/253354 | 9/13060 | 22/14730 | 11/12592 | 11/9249 | 5/9265 |
| Multivariable model* | 1 (Reference) | 0.80 (0.40-1.59) | 1.67 (1.04-2.68) | 1.06 (0.56-2.02) | 1.28 (0.67-2.47) | 0.52 (0.20-1.31) |
| **Cirrhosis** |  |  |  |  |  |  |
| Cases/person-years | 75/253737 | 10/13057 | 6/14820 | 2/12635 | 5/9293 | 2/9266 |
| Multivariable model* | 1 (Reference) | 1.66 (0.84-3.27) | 0.74 (0.31-1.75) | 0.27 (0.06-1.11) | 0.72 (0.27-1.90) | 0.25 (0.06-1.07) |
| **Biliary diseases** |  |  |  |  |  |  |
| Cases/person-years | 560/250705 | 42/12901 | 39/14585 | 41/12423 | 33/9118 | 27/9123 |
| Multivariable model* | 1 (Reference) | 1.14 (0.83-1.57) | 0.89 (0.64-1.24) | 1.19 (0.85-1.65) | 1.22 (0.84-1.77) | 1.00 (0.66-1.51) |
| **Appendicitis** |  |  |  |  |  |  |
| Cases/person-years | 114/253166 | 8/13019 | 3/14811 | 1/12627 | 2/9288 | 4/9252 |
| Multivariable model* | 1 (Reference) | 1.26 (0.61-2.61) | 0.4 (0.12-1.27) | 0.16 (0.02-1.12) | 0.42 (0.1-1.73) | 0.86 (0.30-2.47) |
| **Gastrointestinal cancer** |  |  |  |  |  |  |
| Cases/person-years | 317/252780 | 40/12950 | 25/14717 | 24/12558 | 16/9254 | 24/9201 |
| Multivariable model* | 1 (Reference) | 1.70 (1.21-2.38) | 0.86 (0.56-1.31) | 0.92 (0.59-1.42) | 0.78 (0.46-1.32) | 1.11 (0.70-1.74) |
| **Any gastrointestinal diseases** |  |  |  |  |  |  |
| Cases/person-years | 3994/229780 | 264/11495 | 269/13148 | 218/11356 | 173/8239 | 183/8255 |
| Multivariable model* | 1 (Reference) | 1.07 (0.94-1.21) | 0.90 (0.79-1.02) | **0.87 (0.76-1.00)** | 0.94 (0.81-1.11) | 0.94 (0.80-1.10) |

Note: *Estimates are hazard ratios (95% confidence intervals), obtained from fully adjusted Cox regression models. GI, gastrointestinal; GERD, Gastroesophageal reflux disease; IBS, Irritable bowel syndrome; NAFLD, Non-alcoholic fatty liver disease.

# Supplementary Table 11. Associations of coffee consumption with incident GI diseases using Fine and Gray's competing risk model

| **Outcome** | **Nonconsumers** | **Coffee intake, drinks/d** | | | | |
| --- | --- | --- | --- | --- | --- | --- |
|  |  | **≤ 1** | **1-2** | **2-3** | **3-4** | **> 4** |
| **Unsweetened coffee** |  |  |  |  |  |  |
| **Number of participants** | 35528 | 17627 | 22684 | 18561 | 12583 | 11022 |
| **Barrett’s oesophagus** |  |  |  |  |  |  |
| Cases/person-years | 309/453611 | 133/226658 | 160/292328 | 131/239259 | 94/162308 | 87/141762 |
| Multivariable model* | 1 (Reference) | 0.93 (0.75-1.14) | 0.89 (0.73-1.09) | 0.91 (0.73-1.12) | 0.98 (0.77-1.25) | 1.04 (0.80-1.34) |
| **GERD** |  |  |  |  |  |  |
| Cases/person-years | 2273/442959 | 1030/221536 | 1200/286720 | 933/234971 | 708/158973 | 634/138710 |
| Multivariable model* | 1 (Reference) | 0.96 (0.89-1.03) | **0.88 (0.82-0.95)** | **0.84 (0.78-0.92)** | 0.96 (0.87-1.05) | 0.97 (0.88-1.07) |
| **Gastritis and duodenitis** |  |  |  |  |  |  |
| Cases/person-years | 2268/441280 | 998/221183 | 1147/286092 | 897/234546 | 624/159154 | 530/139203 |
| Multivariable model* | 1 (Reference) | **0.91 (0.85-0.98)** | **0.83 (0.77-0.89)** | **0.8 (0.74-0.87)** | **0.82 (0.75-0.91)** | **0.79 (0.71-0.87)** |
| **Celiac disease** |  |  |  |  |  |  |
| Cases/person-years | 122/454432 | 56/227012 | 64/292768 | 43/239683 | 33/162603 | 23/142079 |
| Multivariable model* | 1 (Reference) | 0.92 (0.67-1.27) | 0.85 (0.62-1.15) | 0.72 (0.50-1.03) | 0.83 (0.56-1.24) | 0.69 (0.43-1.09) |
| **Peptic ulcer** |  |  |  |  |  |  |
| Cases/person-years | 490/452238 | 177/226312 | 230/291830 | 171/238971 | 138/162032 | 116/141592 |
| Multivariable model* | 1 (Reference) | **0.75 (0.63-0.89)** | **0.74 (0.63-0.87)** | **0.67 (0.55-0.80)** | **0.78 (0.63-0.95)** | **0.71 (0.57-0.88)** |
| **Crohn’s disease** |  |  |  |  |  |  |
| Cases/person-years | 58/454866 | 20/227287 | 30/292987 | 20/239854 | 11/162736 | 24/142086 |
| Multivariable model* | 1 (Reference) | 0.79 (0.47-1.33) | 0.95 (0.59-1.55) | 0.79 (0.46-1.36) | 0.64 (0.33-1.23) | 1.55 (0.92-2.63) |
| **Ulcerative colitis** |  |  |  |  |  |  |
| Cases/person-years | 143/454397 | 59/227018 | 66/292762 | 57/239601 | 47/162533 | 34/142029 |
| Multivariable model* | 1 (Reference) | 0.95 (0.70-1.29) | 0.84 (0.62-1.14) | 0.88 (0.64-1.22) | 1.06 (0.75-1.50) | 0.85 (0.57-1.28) |
| **IBS** |  |  |  |  |  |  |
| Cases/person-years | 411/453049 | 201/226354 | 217/292024 | 173/239032 | 124/162186 | 101/141762 |
| Multivariable model* | 1 (Reference) | 1.00 (0.84-1.19) | 0.86 (0.73-1.03) | 0.86 (0.71-1.04) | 0.91 (0.73-1.13) | 0.82 (0.65-1.04) |
| **Diverticulum** |  |  |  |  |  |  |
| Cases/person-years | 3055/437556 | 1541/218549 | 1819/282841 | 1484/231657 | 997/157026 | 860/137434 |
| Multivariable model* | 1 (Reference) | 0.96 (0.90-1.03) | **0.87 (0.82-0.92)** | **0.86 (0.80-0.92)** | **0.84 (0.78-0.91)** | **0.82 (0.75-0.89)** |
| **Pancreatitis** |  |  |  |  |  |  |
| Cases/person-years | 143/454454 | 55/227091 | 84/292729 | 58/239651 | 41/162621 | 44/141968 |
| Multivariable model* | 1 (Reference) | 0.84 (0.61-1.16) | 1.00 (0.75-1.34) | 0.85 (0.61-1.18) | 0.87 (0.60-1.27) | 1.03 (0.70-1.50) |
| **NAFLD** |  |  |  |  |  |  |
| Cases/person-years | 296/454047 | 92/226864 | 110/292676 | 89/239588 | 67/162490 | 71/141919 |
| Multivariable model* | 1 (Reference) | **0.75 (0.59-0.96)** | **0.66 (0.52-0.83)** | **0.62 (0.48-0.80)** | **0.63 (0.47-0.84)** | **0.67 (0.50-0.90)** |
| **Cirrhosis** |  |  |  |  |  |  |
| Cases/person-years | 140/454818 | 57/227173 | 57/292933 | 45/239761 | 32/162723 | 29/142148 |
| Multivariable model* | 1 (Reference) | 0.87 (0.63-1.20) | **0.62 (0.44-0.86)** | **0.55 (0.38-0.8)** | **0.54 (0.35-0.83)** | **0.50 (0.33-0.77)** |
| **Biliary diseases** |  |  |  |  |  |  |
| Cases/person-years | 1069/449042 | 436/224804 | 530/290025 | 459/237133 | 344/160745 | 303/140412 |
| Multivariable model* | 1 (Reference) | **0.87 (0.78-0.98)** | **0.82 (0.74-0.92)** | **0.87 (0.77-0.97)** | 0.94 (0.82-1.07) | 0.92 (0.80-1.06) |
| **Appendicitis** |  |  |  |  |  |  |
| Cases/person-years | 215/453724 | 99/226625 | 115/292412 | 91/239311 | 65/162381 | 54/141864 |
| Multivariable model* | 1 (Reference) | 0.93 (0.73-1.19) | 0.84 (0.66-1.06) | 0.80 (0.62-1.04) | 0.84 (0.62-1.13) | 0.77 (0.56-1.07) |
| **Gastrointestinal cancer** |  |  |  |  |  |  |
| Cases/person-years | 606/453054 | 330/226173 | 439/291567 | 366/238581 | 248/161893 | 224/141475 |
| Multivariable model* | 1 (Reference) | 0.98 (0.85-1.12) | 0.97 (0.85-1.11) | 0.98 (0.85-1.12) | 0.97 (0.83-1.14) | 0.99 (0.84-1.17) |
| **Any gastrointestinal diseases** |  |  |  |  |  |  |
| Cases/person-years | 7365/410565 | 3465/206408 | 4155/268499 | 3335/220416 | 2341/148955 | 2054/130379 |
| Multivariable model* | 1 (Reference) | **0.94 (0.90-0.98)** | **0.87 (0.83-0.90)** | **0.85 (0.81-0.89)** | **0.88 (0.84-0.93)** | **0.87 (0.82-0.92)** |
| **Sugar-sweetened coffee** |  |  |  |  |  |  |
| **Number of participants** | 35528 | 6512 | 5963 | 3935 | 2256 | 2013 |
| **Barrett’s oesophagus** |  |  |  |  |  |  |
| Cases/person-years | 309/453611 | 86/82862 | 45/76215 | 35/50242 | 15/28650 | 30/25383 |
| Multivariable model* | 1 (Reference) | 1.38 (1.08-1.76) | 0.79 (0.58-1.09) | 0.96 (0.66-1.38) | 0.70 (0.42-1.19) | 1.62 (1.09-2.41) |
| **GERD** |  |  |  |  |  |  |
| Cases/person-years | 2273/442959 | 452/80730 | 373/74358 | 278/48934 | 147/27866 | 127/24917 |
| Multivariable model* | 1 (Reference) | 1.06 (0.96-1.18) | 0.97 (0.86-1.08) | 1.09 (0.96-1.25) | 1.00 (0.84-1.19) | 0.94 (0.78-1.14) |
| **Gastritis and duodenitis** |  |  |  |  |  |  |
| Cases/person-years | 2268/441280 | 382/80918 | 353/74223 | 221/49102 | 117/27974 | 120/24798 |
| Multivariable model* | 1 (Reference) | **0.86 (0.77-0.96)** | **0.89 (0.79-1.00)** | **0.85 (0.73-0.98)** | **0.77 (0.63-0.93)** | 0.86 (0.71-1.04) |
| **Celiac disease** |  |  |  |  |  |  |
| Cases/person-years | 122/454432 | 20/83161 | 17/76379 | 5/50409 | 0/28732 | 7/25504 |
| Multivariable model* | 1 (Reference) | 0.90 (0.56-1.45) | 0.91 (0.54-1.53) | 0.43 (0.17-1.08) | 0 (0-0) | 1.41 (0.63-3.17) |
| **Peptic ulcer** |  |  |  |  |  |  |
| Cases/person-years | 490/452238 | 81/82846 | 70/76017 | 55/50138 | 28/28576 | 28/25444 |
| Multivariable model* | 1 (Reference) | 0.80 (0.63-1.02) | **0.75 (0.58-0.98)** | 0.88 (0.65-1.18) | 0.76 (0.51-1.14) | 0.79 (0.53-1.19) |
| **Crohn’s disease** |  |  |  |  |  |  |
| Cases/person-years | 58/454866 | 14/83198 | 15/76381 | 5/50411 | 2/28716 | 3/25541 |
| Multivariable model* | 1 (Reference) | 1.21 (0.68-2.16) | 1.35 (0.73-2.51) | 0.66 (0.25-1.73) | 0.43 (0.10-1.82) | 0.64 (0.19-2.17) |
| **Ulcerative colitis** |  |  |  |  |  |  |
| Cases/person-years | 143/454397 | 20/83175 | 20/76371 | 18/50311 | 4/28708 | 7/25504 |
| Multivariable model* | 1 (Reference) | 0.73 (0.45-1.19) | 0.77 (0.48-1.25) | 1.02 (0.60-1.75) | 0.38 (0.13-1.06) | 0.64 (0.28-1.48) |
| **IBS** |  |  |  |  |  |  |
| Cases/person-years | 411/453049 | 71/82936 | 62/76137 | 41/50248 | 20/28632 | 17/25464 |
| Multivariable model* | 1 (Reference) | 1.03 (0.80-1.35) | 1.03 (0.78-1.37) | 1.08 (0.77-1.52) | 0.96 (0.60-1.52) | 0.89 (0.54-1.49) |
| **Diverticulum** |  |  |  |  |  |  |
| Cases/person-years | 3055/437556 | 624/79804 | 540/73419 | 342/48441 | 205/27653 | 189/24488 |
| Multivariable model* | 1 (Reference) | 1.01 (0.92-1.10) | 0.93 (0.84-1.02) | **0.88 (0.79-1.00)** | 0.89 (0.77-1.04) | 0.95 (0.81-1.11) |
| **Pancreatitis** |  |  |  |  |  |  |
| Cases/person-years | 143/454454 | 36/83143 | 25/76344 | 13/50378 | 8/28693 | 7/25535 |
| Multivariable model* | 1 (Reference) | 1.32 (0.90-1.94) | 0.96 (0.62-1.50) | 0.74 (0.40-1.34) | 0.74 (0.34-1.59) | 0.65 (0.28-1.50) |
| **NAFLD** |  |  |  |  |  |  |
| Cases/person-years | 296/454047 | 53/83053 | 48/76247 | 21/50353 | 22/28650 | 21/25458 |
| Multivariable model* | 1 (Reference) | 1.01 (0.75-1.36) | 0.95 (0.69-1.31) | **0.61 (0.39-0.96)** | 1.01 (0.63-1.60) | 0.92 (0.57-1.48) |
| **Cirrhosis** |  |  |  |  |  |  |
| Cases/person-years | 140/454818 | 24/83232 | 21/76420 | 14/50408 | 10/28702 | 9/25517 |
| Multivariable model* | 1 (Reference) | 0.76 (0.48-1.19) | 0.62 (0.38-1.01) | 0.58 (0.32-1.03) | 0.62 (0.31-1.25) | 0.54 (0.25-1.15) |
| **Biliary diseases** |  |  |  |  |  |  |
| Cases/person-years | 1069/449042 | 207/82116 | 160/75587 | 108/49801 | 70/28355 | 51/25281 |
| Multivariable model* | 1 (Reference) | 1.10 (0.94-1.28) | 0.94 (0.79-1.11) | 0.96 (0.78-1.18) | 1.05 (0.81-1.35) | 0.85 (0.63-1.14) |
| **Appendicitis** |  |  |  |  |  |  |
| Cases/person-years | 215/453724 | 42/83040 | 33/76296 | 19/50303 | 6/28695 | 20/25412 |
| Multivariable model* | 1 (Reference) | 1.08 (0.77-1.51) | 0.90 (0.61-1.32) | 0.78 (0.47-1.29) | **0.43 (0.19-0.99)** | 1.51 (0.90-2.52) |
| **Gastrointestinal cancer** |  |  |  |  |  |  |
| Cases/person-years | 606/453054 | 129/82856 | 126/75939 | 93/50109 | 45/28578 | 60/25364 |
| Multivariable model* | 1 (Reference) | 0.91 (0.75-1.10) | 0.9 (0.73-1.10) | 0.98 (0.78-1.23) | 0.77 (0.56-1.07) | 1.18 (0.88-1.57) |
| **Any gastrointestinal diseases** |  |  |  |  |  |  |
| Cases/person-years | 7365/410565 | 1417/74740 | 1240/68960 | 814/45582 | 457/26031 | 413/23076 |
| Multivariable model* | 1 (Reference) | 1.00 (0.94-1.06) | 0.94 (0.89-1.01) | 0.94 (0.87-1.01) | **0.89 (0.81-0.99)** | 0.91 (0.82-1.01) |
| **Artificially sweetened coffee** |  |  |  |  |  |  |
| **Number of participants** | 35528 | 1944 | 2205 | 1820 | 1296 | 1314 |
| **Barrett’s oesophagus** |  |  |  |  |  |  |
| Cases/person-years | 309/453611 | 28/24666 | 16/28200 | 11/23217 | 13/16508 | 15/16707 |
| Multivariable model* | 1 (Reference) | 1.37 (0.92-2.04) | 0.70 (0.42-1.18) | 0.63 (0.34-1.16) | 1.04 (0.59-1.82) | 1.22 (0.70-2.13) |
| **GERD** |  |  |  |  |  |  |
| Cases/person-years | 2273/442959 | 147/23964 | 165/27360 | 135/22546 | 83/16128 | 107/16234 |
| Multivariable model* | 1 (Reference) | 0.97 (0.82-1.15) | 0.94 (0.79-1.10) | 0.99 (0.82-1.19) | 0.84 (0.67-1.06) | 1.01 (0.82-1.25) |
| **Gastritis and duodenitis** |  |  |  |  |  |  |
| Cases/person-years | 2268/441280 | 144/23877 | 159/27374 | 107/22648 | 94/16053 | 97/16193 |
| Multivariable model* | 1 (Reference) | 0.98 (0.82-1.16) | 0.94 (0.79-1.11) | **0.8 (0.66-0.98)** | 0.98 (0.79-1.21) | 0.97 (0.78-1.21) |
| **Celiac disease** |  |  |  |  |  |  |
| Cases/person-years | 122/454432 | 6/24772 | 3/28274 | 4/23253 | 3/16577 | 3/16780 |
| Multivariable model* | 1 (Reference) | 1.05 (0.45-2.45) | 0.52 (0.16-1.70) | 0.91 (0.33-2.49) | 1.08 (0.34-3.38) | 1.16 (0.36-3.74) |
| **Peptic ulcer** |  |  |  |  |  |  |
| Cases/person-years | 490/452238 | 33/24656 | 42/28072 | 26/23151 | 16/16503 | 22/16670 |
| Multivariable model* | 1 (Reference) | 1.00 (0.70-1.42) | 1.09 (0.78-1.52) | 0.86 (0.57-1.29) | 0.71 (0.43-1.19) | 0.94 (0.59-1.49) |
| **Crohn’s disease** |  |  |  |  |  |  |
| Cases/person-years | 58/454866 | 3/24791 | 4/28258 | 3/23250 | 1/16585 | 1/16793 |
| Multivariable model* | 1 (Reference) | **0.89 (0.27-2.87)** | 1.01 (0.37-2.75) | 0.93 (0.28-3.10) | 0.41 (0.05-3.22) | 0.36 (0.05-2.75) |
| **Ulcerative colitis** |  |  |  |  |  |  |
| Cases/person-years | 143/454397 | 8/24754 | 14/28194 | 9/23210 | 5/16567 | 6/16749 |
| Multivariable model* | 1 (Reference) | 0.97 (0.48-1.96) | 1.53 (0.86-2.73) | 1.19 (0.58-2.42) | 0.89 (0.35-2.26) | 1.02 (0.41-2.58) |
| **IBS** |  |  |  |  |  |  |
| Cases/person-years | 411/453049 | 26/24687 | 24/28172 | 24/23138 | 17/16510 | 18/16705 |
| Multivariable model* | 1 (Reference) | 0.97 (0.65-1.45) | 0.76 (0.49-1.17) | 0.99 (0.65-1.51) | 0.97 (0.59-1.58) | 0.95 (0.58-1.56) |
| **Diverticulum** |  |  |  |  |  |  |
| Cases/person-years | 3055/437556 | 201/23725 | 217/27011 | 176/22232 | 138/15747 | 134/16102 |
| Multivariable model* | 1 (Reference) | 0.91 (0.79-1.06) | 0.84 (0.73-0.97) | 0.84 (0.72-0.99) | 0.90 (0.75-1.07) | **0.83 (0.69-1.00)** |
| **Pancreatitis** |  |  |  |  |  |  |
| Cases/person-years | 143/454454 | 9/24768 | 11/28231 | 9/23222 | 6/16545 | 9/16766 |
| Multivariable model* | 1 (Reference) | 0.86 (0.44-1.70) | 0.89 (0.48-1.66) | 0.91 (0.45-1.82) | 0.76 (0.31-1.86) | 1.04 (0.49-2.22) |
| **NAFLD** |  |  |  |  |  |  |
| Cases/person-years | 296/454047 | 21/24742 | 36/28098 | 21/23195 | 21/16495 | 10/16768 |
| Multivariable model* | 1 (Reference) | 0.84 (0.53-1.33) | 1.16 (0.80-1.68) | 0.91 (0.57-1.46) | 1.11 (0.69-1.79) | 0.44 (0.23-0.87) |
| **Cirrhosis** |  |  |  |  |  |  |
| Cases/person-years | 140/454818 | 17/24740 | 15/28236 | 6/23259 | 7/16564 | 3/16788 |
| Multivariable model* | 1 (Reference) | 1.28 (0.77-2.15) | 0.88 (0.50-1.54) | 0.40 (0.17-0.95) | 0.58 (0.26-1.32) | 0.21 (0.06-0.70) |
| **Biliary diseases** |  |  |  |  |  |  |
| Cases/person-years | 1069/449042 | 77/24434 | 69/27858 | 78/22839 | 58/16248 | 54/16505 |
| Multivariable model* | 1 (Reference) | 1.04 (0.82-1.31) | 0.79 (0.62-1.02) | 1.17 (0.92-1.49) | 1.18 (0.89-1.56) | 1.03 (0.77-1.38) |
| **Appendicitis** |  |  |  |  |  |  |
| Cases/person-years | 215/453724 | 12/24700 | 12/28182 | 8/23203 | 5/16543 | 5/16772 |
| Multivariable model* | 1 (Reference) | 0.98 (0.54-1.77) | 0.83 (0.45-1.52) | 0.68 (0.33-1.41) | 0.58 (0.24-1.43) | 0.55 (0.21-1.39) |
| **Gastrointestinal cancer** |  |  |  |  |  |  |
| Cases/person-years | 606/453054 | 66/24562 | 52/28102 | 51/23078 | 32/16483 | 42/16675 |
| Multivariable model* | 1 (Reference) | 1.41 (1.09-1.83) | 0.93 (0.69-1.24) | 1.06 (0.78-1.42) | 0.88 (0.61-1.27) | 1.12 (0.80-1.56) |
| **Any gastrointestinal diseases** |  |  |  |  |  |  |
| Cases/person-years | 7365/410565 | 478/21878 | 518/25129 | 424/20754 | 312/14676 | 330/14920 |
| Multivariable model* | 1 (Reference) | 0.98 (0.89-1.07) | 0.90 (0.82-0.99) | 0.92 (0.83-1.02) | 0.94 (0.83-1.06) | 0.94 (0.84-1.06) |

Note: *Estimates are hazard ratios (95% confidence intervals), obtained from fully adjusted Cox regression models. GI, gastrointestinal; GERD, Gastroesophageal reflux disease; IBS, Irritable bowel syndrome; NAFLD, Non-alcoholic fatty liver disease.

# Supplementary Table 12. Associations of coffee consumption with incident GI diseases after excluding participants who had an outcome event during the first two years of follow-up

| **Outcome** | **Nonconsumers** | **Coffee intake, drinks/d** | | | | |
| --- | --- | --- | --- | --- | --- | --- |
|  |  | **≤ 1** | **1-2** | **2-3** | **3-4** | **> 4** |
| **Unsweetened coffee** |  |  |  |  |  |  |
| **Number of participants** | 34610 | 17179 | 22167 | 18189 | 12310 | 10817 |
| **Barrett’s oesophagus** |  |  |  |  |  |  |
| Cases/person-years | 285/442480 | 114/221108 | 144/286054 | 124/234722 | 79/159006 | 78/139250 |
| Multivariable model* | 1 (Reference) | 0.85 (0.68-1.06) | 0.85 (0.69-1.05) | 0.9 (0.72-1.12) | 0.86 (0.66-1.12) | 0.96 (0.73-1.26) |
| **GERD** |  |  |  |  |  |  |
| Cases/person-years | 1998/434122 | 882/217329 | 1065/281606 | 827/231375 | 629/156280 | 554/136891 |
| Multivariable model* | 1 (Reference) | **0.92 (0.85-1.00)** | **0.88 (0.81-0.95)** | **0.83 (0.76-0.91)** | 0.95 (0.86-1.04) | 0.94 (0.85-1.04) |
| **Gastritis and duodenitis** |  |  |  |  |  |  |
| Cases/person-years | 1929/433449 | 829/217185 | 973/281525 | 780/231134 | 535/156652 | 467/137237 |
| Multivariable model* | 1 (Reference) | **0.88 (0.81-0.96)** | **0.81 (0.75-0.88)** | **0.8 (0.73-0.88)** | **0.81 (0.74-0.90)** | **0.8 (0.72-0.89)** |
| **Celiac disease** |  |  |  |  |  |  |
| Cases/person-years | 98/443367 | 45/221471 | 55/286487 | 38/235129 | 27/159250 | 18/139546 |
| Multivariable model* | 1 (Reference) | 0.91 (0.64-1.31) | 0.89 (0.63-1.26) | 0.77 (0.52-1.15) | 0.83 (0.53-1.30) | 0.65 (0.38-1.11) |
| **Peptic ulcer** |  |  |  |  |  |  |
| Cases/person-years | 409/441716 | 145/220958 | 191/285828 | 147/234601 | 124/158787 | 107/139108 |
| Multivariable model* | 1 (Reference) | **0.72 (0.59-0.88)** | **0.73 (0.61-0.87)** | **0.67 (0.55-0.82)** | 0.81 (0.66-1.01) | 0.75 (0.60-0.95) |
| **Crohn’s disease** |  |  |  |  |  |  |
| Cases/person-years | 45/443698 | 17/221647 | 24/286672 | 19/235272 | 8/159363 | 23/139518 |
| Multivariable model* | 1 (Reference) | 0.88 (0.50-1.55) | 0.97 (0.58-1.64) | 0.93 (0.53-1.66) | 0.57 (0.26-1.24) | **1.77 (1.00-3.12)** |
| **Ulcerative colitis** |  |  |  |  |  |  |
| Cases/person-years | 116/443347 | 46/221493 | 53/286516 | 52/235061 | 37/159221 | 29/139497 |
| Multivariable model* | 1 (Reference) | 0.91 (0.64-1.29) | 0.83 (0.59-1.17) | 0.99 (0.70-1.41) | 1.03 (0.69-1.53) | 0.89 (0.57-1.39) |
| **IBS** |  |  |  |  |  |  |
| Cases/person-years | 367/442161 | 166/220968 | 189/285907 | 157/234590 | 106/158918 | 90/139280 |
| Multivariable model* | 1 (Reference) | 0.92 (0.76-1.11) | **0.83 (0.69-1.00)** | 0.86 (0.70-1.05) | 0.86 (0.68-1.08) | 0.81 (0.63-1.04) |
| **Diverticulum** |  |  |  |  |  |  |
| Cases/person-years | 2679/430124 | 1340/214874 | 1605/278665 | 1334/228637 | 875/154897 | 778/135749 |
| Multivariable model* | 1 (Reference) | 0.95 (0.89-1.02) | **0.86 (0.81-0.92)** | **0.87 (0.81-0.93)** | **0.83 (0.77-0.90)** | **0.83 (0.76-0.91)** |
| **Pancreatitis** |  |  |  |  |  |  |
| Cases/person-years | 125/443359 | 47/221509 | 73/286448 | 50/235135 | 37/159263 | 37/139463 |
| Multivariable model* | 1 (Reference) | 0.82 (0.58-1.16) | 0.98 (0.72-1.34) | 0.81 (0.57-1.16) | 0.87 (0.59-1.29) | 0.94 (0.63-1.41) |
| **NAFLD** |  |  |  |  |  |  |
| Cases/person-years | 262/442974 | 80/221327 | 96/286399 | 83/235032 | 56/159192 | 66/139385 |
| Multivariable model* | 1 (Reference) | **0.75 (0.58-0.97)** | **0.66 (0.52-0.84)** | **0.66 (0.51-0.86)** | **0.61 (0.44-0.82)** | **0.72 (0.53-0.97)** |
| **Cirrhosis** |  |  |  |  |  |  |
| Cases/person-years | 124/443652 | 47/221577 | 45/286670 | 37/235235 | 28/159353 | 28/139574 |
| Multivariable model* | 1 (Reference) | 0.78 (0.55-1.10) | **0.53 (0.37-0.76)** | **0.49 (0.33-0.72)** | **0.51 (0.33-0.79)** | **0.51 (0.32-0.80)** |
| **Biliary diseases** |  |  |  |  |  |  |
| Cases/person-years | 923/439228 | 373/219819 | 445/284485 | 382/233341 | 299/157787 | 264/138272 |
| Multivariable model* | 1 (Reference) | **0.86 (0.76-0.98)** | **0.80 (0.71-0.90)** | **0.83 (0.73-0.94)** | 0.94 (0.82-1.08) | 0.92 (0.79-1.07) |
| **Appendicitis** |  |  |  |  |  |  |
| Cases/person-years | 174/442917 | 73/221257 | 94/286265 | 78/234877 | 52/159137 | 48/139352 |
| Multivariable model* | 1 (Reference) | 0.81 (0.62-1.08) | 0.79 (0.61-1.03) | 0.79 (0.59-1.05) | 0.75 (0.54-1.05) | 0.76 (0.54-1.08) |
| **Gastrointestinal cancer** |  |  |  |  |  |  |
| Cases/person-years | 531/442252 | 303/220748 | 390/285547 | 326/234276 | 228/158613 | 214/138987 |
| Multivariable model* | 1 (Reference) | 1.03 (0.89-1.19) | 0.99 (0.86-1.14) | 0.99 (0.86-1.15) | 1.02 (0.86-1.20) | 1.08 (0.90-1.28) |
| **Any gastrointestinal diseases** |  |  |  |  |  |  |
| Cases/person-years | 6480/409133 | 3040/205608 | 3661/267646 | 2977/219841 | 2075/148548 | 1857/130067 |
| Multivariable model* | 1 (Reference) | **0.93 (0.89-0.97)** | **0.86 (0.83-0.90)** | **0.85 (0.81-0.89)** | **0.88 (0.83-0.92)** | **0.88 (0.83-0.93)** |
| **Sugar-sweetened coffee** |  |  |  |  |  |  |
| **Number of participants** | 34610 | 6345 | 5810 | 3845 | 2201 | 1948 |
| **Barrett’s oesophagus** |  |  |  |  |  |  |
| Cases/person-years | 285/442480 | 76/80865 | 42/74354 | 31/49206 | 12/27983 | 25/24638 |
| Multivariable model* | 1 (Reference) | 1.31 (1.01-1.71) | 0.78 (0.56-1.09) | 0.89 (0.61-1.32) | 0.59 (0.32-1.07) | 1.42 (0.91-2.22) |
| **GERD** |  |  |  |  |  |  |
| Cases/person-years | 1998/434122 | 391/79239 | 330/72881 | 249/48143 | 123/27429 | 110/24268 |
| Multivariable model* | 1 (Reference) | 1.04 (0.93-1.16) | 0.95 (0.84-1.08) | 1.10 (0.95-1.26) | 0.93 (0.77-1.13) | 0.92 (0.75-1.12) |
| **Gastritis and duodenitis** |  |  |  |  |  |  |
| Cases/person-years | 1929/433449 | 328/79365 | 297/72940 | 191/48324 | 96/27524 | 96/24274 |
| Multivariable model* | 1 (Reference) | **0.87 (0.77-0.98)** | **0.86 (0.76-0.98)** | **0.84 (0.72-0.98)** | **0.73 (0.59-0.90)** | **0.79 (0.64-0.99)** |
| **Celiac disease** |  |  |  |  |  |  |
| Cases/person-years | 98/443367 | 18/81101 | 17/74498 | 5/49346 | 0/28049 | 3/24773 |
| Multivariable model* | 1 (Reference) | 1.04 (0.62-1.75) | 1.18 (0.69-2.02) | 0.57 (0.23-1.43) | 0 (0-Inf) | 0.81 (0.25-2.67) |
| **Peptic ulcer** |  |  |  |  |  |  |
| Cases/person-years | 409/441716 | 68/80875 | 54/74290 | 49/49135 | 24/27934 | 24/24688 |
| Multivariable model* | 1 (Reference) | 0.80 (0.61-1.04) | **0.68 (0.50-0.91)** | 0.91 (0.67-1.25) | 0.77 (0.50-1.18) | 0.79 (0.51-1.24) |
| **Crohn’s disease** |  |  |  |  |  |  |
| Cases/person-years | 45/443698 | 11/81149 | 10/74549 | 5/49349 | 1/28044 | 3/24762 |
| Multivariable model* | 1 (Reference) | 1.19 (0.61-2.35) | 1.13 (0.55-2.34) | 0.82 (0.31-2.17) | 0.27 (0.04-2.01) | 0.77 (0.22-2.73) |
| **Ulcerative colitis** |  |  |  |  |  |  |
| Cases/person-years | 116/443347 | 19/81119 | 17/74524 | 14/49287 | 4/28025 | 3/24774 |
| Multivariable model* | 1 (Reference) | 0.85 (0.52-1.40) | 0.80 (0.47-1.36) | 0.97 (0.54-1.74) | 0.45 (0.16-1.26) | 0.33 (0.10-1.07) |
| **IBS** |  |  |  |  |  |  |
| Cases/person-years | 367/442161 | 65/80913 | 54/74334 | 40/49192 | 16/27989 | 14/24710 |
| Multivariable model* | 1 (Reference) | 1.06 (0.81-1.38) | 1.00 (0.74-1.34) | 1.17 (0.83-1.65) | 0.85 (0.51-1.42) | 0.82 (0.47-1.43) |
| **Diverticulum** |  |  |  |  |  |  |
| Cases/person-years | 2679/430124 | 550/78405 | 472/72213 | 309/47721 | 188/27128 | 162/23967 |
| Multivariable model* | 1 (Reference) | 1.02 (0.93-1.12) | 0.93 (0.84-1.03) | 0.92 (0.81-1.04) | 0.95 (0.81-1.11) | 0.95 (0.80-1.12) |
| **Pancreatitis** |  |  |  |  |  |  |
| Cases/person-years | 125/443359 | 33/81094 | 20/74500 | 13/49315 | 8/28010 | 6/24756 |
| Multivariable model* | 1 (Reference) | 1.41 (0.95-2.10) | 0.90 (0.55-1.47) | 0.87 (0.48-1.59) | 0.88 (0.42-1.85) | 0.67 (0.28-1.59) |
| **NAFLD** |  |  |  |  |  |  |
| Cases/person-years | 262/442974 | 46/81034 | 45/74382 | 18/49315 | 19/27989 | 19/24695 |
| Multivariable model* | 1 (Reference) | 0.99 (0.72-1.37) | 1.01 (0.73-1.41) | **0.60 (0.36-0.98)** | 1.01 (0.62-1.65) | 1.00 (0.60-1.65) |
| **Cirrhosis** |  |  |  |  |  |  |
| Cases/person-years | 124/443652 | 20/81178 | 20/74543 | 11/49360 | 9/28020 | 5/24761 |
| Multivariable model* | 1 (Reference) | 0.68 (0.42-1.10) | 0.63 (0.38-1.03) | **0.47 (0.25-0.91)** | 0.58 (0.28-1.20) | 0.32 (0.12-0.81) |
| **Biliary diseases** |  |  |  |  |  |  |
| Cases/person-years | 923/439228 | 179/80321 | 140/73860 | 89/48905 | 60/27771 | 44/24567 |
| Multivariable model* | 1 (Reference) | 1.1 (0.93-1.30) | 0.95 (0.79-1.15) | 0.93 (0.74-1.17) | 1.06 (0.81-1.39) | 0.88 (0.64-1.21) |
| **Appendicitis** |  |  |  |  |  |  |
| Cases/person-years | 174/442917 | 35/81051 | 30/74452 | 15/49277 | 5/28023 | 16/24679 |
| Multivariable model* | 1 (Reference) | 1.09 (0.75-1.58) | 0.98 (0.65-1.47) | 0.72 (0.42-1.25) | 0.42 (0.17-1.03) | 1.41 (0.80-2.48) |
| **Gastrointestinal cancer** |  |  |  |  |  |  |
| Cases/person-years | 531/442252 | 110/80918 | 111/74180 | 81/49123 | 42/27911 | 52/24643 |
| Multivariable model* | 1 (Reference) | 0.90 (0.73-1.12) | 0.93 (0.75-1.15) | 1.00 (0.78-1.28) | 0.86 (0.62-1.19) | 1.23 (0.9-1.68) |
| **Any gastrointestinal diseases** |  |  |  |  |  |  |
| Cases/person-years | 6480/409133 | 1255/74471 | 1095/68706 | 725/45472 | 404/25957 | 351/22965 |
| Multivariable model* | 1 (Reference) | 1.01 (0.95-1.07) | 0.94 (0.88-1.01) | 0.94 (0.87-1.02) | **0.89 (0.80-0.99)** | **0.87 (0.78-0.97)** |
| **Artificially sweetened coffee** |  |  |  |  |  |  |
| **Number of participants** | 34610 | 1876 | 2133 | 1766 | 1261 | 1283 |
| **Barrett’s oesophagus** |  |  |  |  |  |  |
| Cases/person-years | 285/442480 | 25/23854 | 15/27309 | 10/22570 | 12/16063 | 14/16323 |
| Multivariable model* | 1 (Reference) | 1.34 (0.88-2.03) | 0.71 (0.42-1.20) | 0.61 (0.32-1.17) | 1.00 (0.55-1.83) | 1.16 (0.65-2.05) |
| **GERD** |  |  |  |  |  |  |
| Cases/person-years | 1998/434122 | 125/23370 | 143/26665 | 119/22027 | 70/15815 | 98/15927 |
| Multivariable model* | 1 (Reference) | 0.94 (0.78-1.13) | 0.92 (0.77-1.09) | 0.98 (0.81-1.18) | 0.79 (0.62-1.01) | 1.03 (0.83-1.28) |
| **Gastritis and duodenitis** |  |  |  |  |  |  |
| Cases/person-years | 1929/433449 | 118/23342 | 130/26746 | 95/22110 | 84/15719 | 87/15899 |
| Multivariable model* | 1 (Reference) | 0.95 (0.79-1.15) | 0.9 (0.75-1.08) | 0.83 (0.67-1.03) | 1.01 (0.80-1.27) | 1.00 (0.79-1.25) |
| **Celiac disease** |  |  |  |  |  |  |
| Cases/person-years | 98/443367 | 6/23950 | 3/27374 | 3/22606 | 2/16133 | 3/16387 |
| Multivariable model* | 1 (Reference) | 1.35 (0.58-3.11) | 0.68 (0.21-2.19) | 0.90 (0.28-2.90) | 0.97 (0.23-4.03) | 1.60 (0.48-5.28) |
| **Peptic ulcer** |  |  |  |  |  |  |
| Cases/person-years | 409/441716 | 26/23882 | 32/27251 | 21/22524 | 15/16063 | 19/16308 |
| Multivariable model* | 1 (Reference) | 0.95 (0.64-1.43) | 1.01 (0.69-1.46) | 0.85 (0.54-1.33) | 0.81 (0.47-1.38) | 0.97 (0.60-1.59) |
| **Crohn’s disease** |  |  |  |  |  |  |
| Cases/person-years | 45/443698 | 2/23981 | 3/27366 | 2/22610 | 1/16138 | 1/16400 |
| Multivariable model* | 1 (Reference) | 0.77 (0.18-3.25) | 0.97 (0.29-3.23) | 0.82 (0.19-3.55) | 0.51 (0.07-3.87) | 0.44 (0.06-3.43) |
| **Ulcerative colitis** |  |  |  |  |  |  |
| Cases/person-years | 116/443347 | 6/23952 | 11/27324 | 8/22570 | 5/16120 | 4/16379 |
| Multivariable model* | 1 (Reference) | 0.92 (0.40-2.10) | 1.51 (0.80-2.88) | 1.38 (0.65-2.92) | 1.18 (0.46-3.00) | 0.91 (0.32-2.57) |
| **IBS** |  |  |  |  |  |  |
| Cases/person-years | 367/442161 | 23/23893 | 19/27312 | 20/22520 | 11/16100 | 15/16345 |
| Multivariable model* | 1 (Reference) | 0.97 (0.63-1.49) | 0.68 (0.42-1.09) | 0.93 (0.58-1.48) | 0.71 (0.38-1.31) | 0.89 (0.51-1.53) |
| **Diverticulum** |  |  |  |  |  |  |
| Cases/person-years | 2679/430124 | 179/23111 | 185/26434 | 152/21810 | 121/15480 | 121/15843 |
| Multivariable model* | 1 (Reference) | 0.94 (0.81-1.10) | **0.83 (0.71-0.96)** | **0.84 (0.71-0.99)** | 0.9 (0.74-1.08) | 0.85 (0.70-1.03) |
| **Pancreatitis** |  |  |  |  |  |  |
| Cases/person-years | 125/443359 | 7/23954 | 8/27353 | 6/22592 | 4/16121 | 9/16373 |
| Multivariable model* | 1 (Reference) | 0.82 (0.38-1.78) | 0.78 (0.37-1.63) | 0.73 (0.31-1.69) | 0.61 (0.22-1.70) | 1.23 (0.58-2.58) |
| **NAFLD** |  |  |  |  |  |  |
| Cases/person-years | 262/442974 | 20/23922 | 32/27232 | 21/22541 | 18/16073 | 10/16375 |
| Multivariable model* | 1 (Reference) | 0.97 (0.61-1.54) | 1.26 (0.86-1.85) | 1.08 (0.68-1.71) | 1.14 (0.69-1.89) | 0.53 (0.27-1.02) |
| **Cirrhosis** |  |  |  |  |  |  |
| Cases/person-years | 124/443652 | 13/23943 | 12/27341 | 5/22606 | 6/16123 | 3/16395 |
| Multivariable model* | 1 (Reference) | 1.16 (0.65-2.08) | 0.80 (0.43-1.48) | 0.39 (0.15-0.97) | 0.56 (0.24-1.33) | **0.23 (0.07-0.77)** |
| **Biliary diseases** |  |  |  |  |  |  |
| Cases/person-years | 923/439228 | 62/23721 | 54/27114 | 60/22364 | 49/15881 | 47/16178 |
| Multivariable model* | 1 (Reference) | 0.99 (0.77-1.29) | **0.74 (0.56-0.98)** | 1.06 (0.81-1.39) | 1.18 (0.87-1.59) | 1.05 (0.77-1.44) |
| **Appendicitis** |  |  |  |  |  |  |
| Cases/person-years | 174/442917 | 8/23923 | 9/27311 | 6/22571 | 4/16102 | 4/16392 |
| Multivariable model* | 1 (Reference) | 0.78 (0.38-1.60) | 0.73 (0.37-1.44) | 0.59 (0.25-1.35) | 0.53 (0.19-1.46) | 0.49 (0.17-1.36) |
| **Gastrointestinal cancer** |  |  |  |  |  |  |
| Cases/person-years | 531/442252 | 56/23799 | 44/27264 | 45/22461 | 29/16047 | 40/16294 |
| Multivariable model* | 1 (Reference) | 1.41 (1.06-1.87) | 0.93 (0.68-1.28) | 1.11 (0.81-1.53) | 0.95 (0.64-1.40) | 1.26 (0.88-1.78) |
| **Any gastrointestinal diseases** |  |  |  |  |  |  |
| Cases/person-years | 6480/409133 | 411/21789 | 447/25026 | 371/20684 | 279/14609 | 301/14866 |
| Multivariable model* | 1 (Reference) | 0.96 (0.87-1.06) | **0.89 (0.80-0.98)** | 0.92 (0.82-1.02) | 0.95 (0.84-1.08) | 0.97 (0.86-1.10) |

Note: *Estimates are hazard ratios (95% confidence intervals), obtained from fully adjusted Cox regression models. GI, gastrointestinal; GERD, Gastroesophageal reflux disease; IBS, Irritable bowel syndrome; NAFLD, Non-alcoholic fatty liver disease.

**Supplementary Table 13. Associations of coffee consumption with incident GI diseases after excluding participants who had an outcome event during the first five years of follow-up**

| **Outcome** | **Nonconsumers** | **Coffee intake, drinks/d** | | | | |
| --- | --- | --- | --- | --- | --- | --- |
|  |  | **≤ 1** | **1-2** | **2-3** | **3-4** | **> 4** |
| **Unsweetened coffee** |  |  |  |  |  |  |
| **Number of participants** | 32803 | 16401 | 21219 | 17431 | 11782 | 10358 |
| **Barrett’s oesophagus** |  |  |  |  |  |  |
| Cases/person-years | 204/420772 | 80/211721 | 103/274673 | 80/225589 | 54/152622 | 56/133729 |
| Multivariable model* | 1 (Reference) | 0.84 (0.64-1.09) | 0.86 (0.67-1.10) | 0.82 (0.62-1.08) | 0.83 (0.60-1.14) | 0.98 (0.71-1.35) |
| **GERD** |  |  |  |  |  |  |
| Cases/person-years | 1463/415925 | 647/209511 | 805/271907 | 625/223461 | 482/150871 | 406/132343 |
| Multivariable model* | 1 (Reference) | 0.91 (0.83-1.01) | **0.9 (0.82-0.98)** | **0.85 (0.77-0.94)** | 0.98 (0.88-1.10) | 0.93 (0.83-1.05) |
| **Gastritis and duodenitis** |  |  |  |  |  |  |
| Cases/person-years | 1329/415996 | 593/209493 | 688/272097 | 552/223591 | 395/151223 | 335/132616 |
| Multivariable model* | 1 (Reference) | 0.92 (0.83-1.01) | **0.83 (0.75-0.91)** | **0.82 (0.73-0.91)** | **0.87 (0.77-0.98)** | **0.82 (0.72-0.94)** |
| **Celiac disease** |  |  |  |  |  |  |
| Cases/person-years | 62/421383 | 33/211917 | 41/274909 | 23/225809 | 19/152744 | 13/133913 |
| Multivariable model* | 1 (Reference) | 1.02 (0.66-1.57) | 1.01 (0.67-1.52) | 0.71 (0.43-1.18) | 0.88 (0.51-1.52) | 0.72 (0.38-1.35) |
| **Peptic ulcer** |  |  |  |  |  |  |
| Cases/person-years | 264/420582 | 97/211678 | 137/274533 | 92/225558 | 83/152507 | 79/133644 |
| Multivariable model* | 1 (Reference) | **0.74 (0.58-0.93)** | **0.79 (0.64-0.98)** | **0.63 (0.49-0.82)** | 0.84 (0.64-1.09) | 0.86 (0.65-1.13) |
| **Crohn’s disease** |  |  |  |  |  |  |
| Cases/person-years | 33/421515 | 13/212019 | 17/275038 | 15/225857 | 4/152817 | 14/133908 |
| Multivariable model* | 1 (Reference) | 0.86 (0.45-1.65) | 0.85 (0.46-1.56) | 0.88 (0.46-1.69) | **0.33 (0.11-0.97)** | 1.23 (0.61-2.48) |
| **Ulcerative colitis** |  |  |  |  |  |  |
| Cases/person-years | 80/421333 | 35/211935 | 36/274955 | 36/225762 | 26/152735 | 20/133882 |
| Multivariable model* | 1 (Reference) | 0.97 (0.64-1.45) | 0.76 (0.50-1.14) | 0.9 (0.59-1.37) | 0.93 (0.58-1.49) | 0.77 (0.45-1.31) |
| **IBS** |  |  |  |  |  |  |
| Cases/person-years | 266/420637 | 129/211564 | 144/274540 | 117/225427 | 78/152565 | 77/133701 |
| Multivariable model* | 1 (Reference) | 0.98 (0.79-1.21) | 0.87 (0.70-1.08) | 0.89 (0.70-1.12) | 0.88 (0.67-1.15) | 0.98 (0.75-1.29) |
| **Diverticulum** |  |  |  |  |  |  |
| Cases/person-years | 1997/413482 | 1037/207730 | 1213/270168 | 1017/221785 | 669/150134 | 592/131567 |
| Multivariable model* | 1 (Reference) | 0.98 (0.91-1.06) | **0.87 (0.81-0.94)** | **0.88 (0.81-0.95)** | **0.84 (0.77-0.92)** | **0.84 (0.76-0.92)** |
| **Pancreatitis** |  |  |  |  |  |  |
| Cases/person-years | 82/421370 | 35/211927 | 47/274939 | 36/225787 | 27/152747 | 26/133874 |
| Multivariable model* | 1 (Reference) | 0.89 (0.60-1.34) | 0.91 (0.62-1.33) | 0.83 (0.55-1.27) | 0.9 (0.57-1.44) | 0.94 (0.58-1.53) |
| **NAFLD** |  |  |  |  |  |  |
| Cases/person-years | 204/420994 | 56/211879 | 77/274823 | 72/225658 | 38/152718 | 57/133763 |
| Multivariable model* | 1 (Reference) | **0.67 (0.50-0.91)** | **0.68 (0.52-0.90)** | **0.75 (0.56-1.00)** | **0.54 (0.37-0.78)** | 0.82 (0.59-1.14) |
| **Cirrhosis** |  |  |  |  |  |  |
| Cases/person-years | 98/421462 | 37/211969 | 37/275006 | 32/225835 | 21/152798 | 22/133915 |
| Multivariable model* | 1 (Reference) | 0.80 (0.54-1.18) | **0.58 (0.39-0.86)** | **0.56 (0.37-0.86)** | **0.52 (0.31-0.85)** | **0.55 (0.33-0.92)** |
| **Biliary diseases** |  |  |  |  |  |  |
| Cases/person-years | 634/419139 | 262/211048 | 297/273955 | 265/224847 | 208/151945 | 185/133233 |
| Multivariable model* | 1 (Reference) | 0.88 (0.76-1.02) | **0.76 (0.66-0.88)** | **0.82 (0.70-0.96)** | 0.94 (0.79-1.11) | 0.92 (0.77-1.11) |
| **Appendicitis** |  |  |  |  |  |  |
| Cases/person-years | 105/421248 | 43/211882 | 61/274858 | 50/225691 | 37/152697 | 28/133844 |
| Multivariable model* | 1 (Reference) | 0.77 (0.54-1.11) | 0.82 (0.59-1.14) | 0.8 (0.56-1.15) | 0.85 (0.57-1.28) | 0.71 (0.45-1.11) |
| **Gastrointestinal cancer** |  |  |  |  |  |  |
| Cases/person-years | 375/420746 | 217/211545 | 286/274389 | 247/225280 | 164/152431 | 165/133553 |
| Multivariable model* | 1 (Reference) | 1.03 (0.87-1.23) | 1.02 (0.87-1.20) | 1.06 (0.89-1.26) | 1.03 (0.85-1.25) | 1.17 (0.96-1.44) |
| **Any gastrointestinal diseases** | | | | | | |
| Cases/person-years | 4759/401766 | 2295/202472 | 2750/263817 | 2260/216678 | 1580/146329 | 1415/128241 |
| Multivariable model* | 1 (Reference) | **0.95 (0.91-1.00)** | **0.87 (0.83-0.92)** | **0.87 (0.82-0.92)** | **0.9 (0.85-0.95)** | **0.9 (0.85-0.96)** |
| **Sugar-sweetened coffee** |  |  |  |  |  |  |
| **Number of participants** | 32803 | 5983 | 5503 | 3642 | 2105 | 1841 |
| **Barrett’s oesophagus** |  |  |  |  |  |  |
| Cases/person-years | 204/420772 | 53/76563 | 27/70698 | 18/46799 | 7/26860 | 13/23428 |
| Multivariable model* | 1 (Reference) | 1.32 (0.96-1.80) | 0.72 (0.47-1.09) | 0.75 (0.46-1.25) | 0.49 (0.23-1.07) | 1.06 (0.58-1.93) |
| **GERD** |  |  |  |  |  |  |
| Cases/person-years | 1463/415925 | 277/75612 | 242/69806 | 187/46137 | 93/26513 | 78/23200 |
| Multivariable model* | 1 (Reference) | 1.03 (0.90-1.17) | 0.97 (0.84-1.12) | 1.15 (0.98-1.35) | 0.98 (0.78-1.22) | 0.91 (0.72-1.16) |
| **Gastritis and duodenitis** |  |  |  |  |  |  |
| Cases/person-years | 1329/415996 | 220/75809 | 199/69916 | 128/46361 | 69/26603 | 56/23256 |
| Multivariable model* | 1 (Reference) | **0.84 (0.73-0.98)** | **0.84 (0.72-0.98)** | **0.81 (0.67-0.98)** | **0.74 (0.58-0.95)** | **0.66 (0.50-0.88)** |
| **Celiac disease** |  |  |  |  |  |  |
| Cases/person-years | 62/421383 | 8/76741 | 9/70786 | 5/46847 | 0/26893 | 3/23481 |
| Multivariable model* | 1 (Reference) | 0.73 (0.35-1.55) | 0.98 (0.47-2.03) | 0.9 (0.35-2.32) | 0 (0-Inf) | 1.26 (0.37-4.30) |
| **Peptic ulcer** |  |  |  |  |  |  |
| Cases/person-years | 264/420582 | 43/76599 | 27/70706 | 31/46753 | 16/26838 | 20/23426 |
| Multivariable model* | 1 (Reference) | 0.79 (0.56-1.09) | **0.54 (0.36-0.81)** | 0.92 (0.62-1.37) | 0.82 (0.48-1.38) | 1.12 (0.68-1.83) |
| **Crohn’s disease** |  |  |  |  |  |  |
| Cases/person-years | 33/421515 | 6/76750 | 7/70792 | 3/46857 | 1/26888 | 2/23480 |
| Multivariable model* | 1 (Reference) | 0.90 (0.37-2.19) | 1.11 (0.47-2.64) | 0.67 (0.19-2.30) | 0.37 (0.05-2.83) | 0.73 (0.16-3.42) |
| **Ulcerative colitis** |  |  |  |  |  |  |
| Cases/person-years | 80/421333 | 13/76716 | 11/70777 | 10/46821 | 4/26869 | 3/23482 |
| Multivariable model* | 1 (Reference) | 0.84 (0.46-1.53) | 0.72 (0.38-1.40) | 0.95 (0.47-1.91) | 0.62 (0.22-1.76) | 0.45 (0.13-1.50) |
| **IBS** |  |  |  |  |  |  |
| Cases/person-years | 266/420637 | 42/76614 | 39/70673 | 31/46763 | 13/26844 | 10/23456 |
| Multivariable model* | 1 (Reference) | 0.93 (0.67-1.29) | 0.99 (0.70-1.40) | 1.25 (0.84-1.85) | 0.95 (0.54-1.70) | 0.85 (0.44-1.65) |
| **Diverticulum** |  |  |  |  |  |  |
| Cases/person-years | 1997/413482 | 405/75161 | 348/69441 | 219/45979 | 145/26316 | 117/23022 |
| Multivariable model* | 1 (Reference) | 1.02 (0.91-1.14) | 0.93 (0.82-1.04) | 0.88 (0.76-1.02) | 0.98 (0.82-1.17) | 0.93 (0.76-1.13) |
| **Pancreatitis** |  |  |  |  |  |  |
| Cases/person-years | 82/421370 | 23/76699 | 15/70758 | 9/46839 | 7/26862 | 5/23473 |
| Multivariable model* | 1 (Reference) | 1.44 (0.89-2.32) | 0.94 (0.52-1.67) | 0.81 (0.39-1.66) | 1.01 (0.45-2.27) | 0.72 (0.27-1.88) |
| **NAFLD** |  |  |  |  |  |  |
| Cases/person-years | 204/420994 | 32/76654 | 29/70731 | 17/46817 | 16/26847 | 15/23434 |
| Multivariable model* | 1 (Reference) | 0.91 (0.62-1.34) | 0.87 (0.58-1.30) | 0.75 (0.45-1.26) | 1.13 (0.66-1.93) | 1.06 (0.60-1.87) |
| **Cirrhosis** |  |  |  |  |  |  |
| Cases/person-years | 98/421462 | 13/76753 | 15/70799 | 7/46867 | 6/26881 | 3/23485 |
| Multivariable model* | 1 (Reference) | 0.58 (0.32-1.04) | 0.61 (0.34-1.08) | **0.39 (0.17-0.87)** | 0.50 (0.21-1.19) | **0.24 (0.07-0.80)** |
| **Biliary diseases** |  |  |  |  |  |  |
| Cases/person-years | 634/419139 | 115/76336 | 94/70470 | 59/46624 | 44/26728 | 33/23372 |
| Multivariable model* | 1 (Reference) | 1.03 (0.84-1.26) | 0.92 (0.73-1.15) | 0.88 (0.66-1.16) | 1.09 (0.79-1.50) | 0.93 (0.64-1.35) |
| **Appendicitis** |  |  |  |  |  |  |
| Cases/person-years | 105/421248 | 24/76669 | 22/70738 | 10/46819 | 4/26876 | 10/23447 |
| Multivariable model* | 1 (Reference) | 1.20 (0.76-1.89) | 1.11 (0.68-1.80) | 0.73 (0.37-1.44) | 0.51 (0.18-1.41) | 1.31 (0.64-2.68) |
| **Gastrointestinal cancer** |  |  |  |  |  |  |
| Cases/person-years | 375/420746 | 76/76580 | 83/70574 | 56/46724 | 34/26801 | 37/23418 |
| Multivariable model* | 1 (Reference) | 0.89 (0.69-1.15) | 0.99 (0.77-1.27) | 0.99 (0.73-1.33) | 0.98 (0.68-1.42) | 1.26 (0.87-1.82) |
| **Any gastrointestinal diseases** |  |  |  |  |  |  |
| Cases/person-years | 4759/401766 | 916/72940 | 801/67444 | 529/44667 | 314/25604 | 252/22497 |
| Multivariable model* | 1 (Reference) | 1.01 (0.94-1.08) | 0.94 (0.87-1.02) | 0.94 (0.86-1.03) | 0.95 (0.84-1.07) | **0.86 (0.75-0.98)** |
| **Artificially sweetened coffee** |  |  |  |  |  |  |
| **Number of participants** | 32803 | 1761 | 2012 | 1679 | 1178 | 1209 |
| **Barrett’s oesophagus** |  |  |  |  |  |  |
| Cases/person-years | 204/420772 | 19/22466 | 9/25874 | 7/21526 | 7/15077 | 10/15479 |
| Multivariable model* | 1 (Reference) | 1.46 (0.90-2.35) | 0.61 (0.31-1.21) | 0.62 (0.28-1.33) | 0.85 (0.39-1.85) | 1.20 (0.61-2.37) |
| **GERD** |  |  |  |  |  |  |
| Cases/person-years | 1463/415925 | 86/22223 | 101/25502 | 90/21183 | 49/14944 | 77/15228 |
| Multivariable model* | 1 (Reference) | 0.90 (0.72-1.13) | 0.9 (0.73-1.10) | 1.02 (0.82-1.28) | 0.79 (0.59-1.06) | 1.16 (0.90-1.48) |
| **Gastritis and duodenitis** |  |  |  |  |  |  |
| Cases/person-years | 1329/415996 | 78/22250 | 96/25518 | 66/21297 | 60/14875 | 62/15241 |
| Multivariable model* | 1 (Reference) | 0.92 (0.73-1.16) | 0.97 (0.78-1.20) | 0.84 (0.65-1.08) | 1.06 (0.81-1.39) | 1.03 (0.78-1.35) |
| **Celiac disease** |  |  |  |  |  |  |
| Cases/person-years | 62/421383 | 4/22534 | 3/25897 | 3/21547 | 2/15113 | 1/15528 |
| Multivariable model* | 1 (Reference) | 1.50 (0.54-4.17) | 1.10 (0.34-3.58) | 1.49 (0.45-4.90) | 1.66 (0.39-7.07) | 0.89 (0.12-6.71) |
| **Peptic ulcer** |  |  |  |  |  |  |
| Cases/person-years | 264/420582 | 18/22502 | 25/25824 | 16/21495 | 11/15085 | 11/15483 |
| Multivariable model* | 1 (Reference) | 1.09 (0.67-1.76) | 1.30 (0.85-1.99) | 1.06 (0.63-1.79) | 1.01 (0.54-1.89) | 0.97 (0.52-1.84) |
| **Crohn’s disease** |  |  |  |  |  |  |
| Cases/person-years | 33/421515 | 1/22555 | 2/25895 | 2/21551 | 1/15118 | 1/15526 |
| Multivariable model* | 1 (Reference) | 0.51 (0.07-3.79) | 0.84 (0.19-3.67) | 1.04 (0.23-4.57) | 0.65 (0.08-5.11) | 0.59 (0.07-4.72) |
| **Ulcerative colitis** |  |  |  |  |  |  |
| Cases/person-years | 80/421333 | 3/22547 | 6/25885 | 6/21528 | 4/15111 | 3/15516 |
| Multivariable model* | 1 (Reference) | 0.65 (0.20-2.08) | 1.18 (0.50-2.78) | 1.44 (0.60-3.43) | 1.35 (0.47-3.88) | 0.98 (0.29-3.28) |
| **IBS** |  |  |  |  |  |  |
| Cases/person-years | 266/420637 | 18/22501 | 15/25844 | 14/21504 | 10/15086 | 13/15483 |
| Multivariable model* | 1 (Reference) | 1.04 (0.64-1.70) | 0.73 (0.43-1.24) | 0.89 (0.51-1.55) | 0.92 (0.48-1.77) | 1.10 (0.61-1.99) |
| **Diverticulum** |  |  |  |  |  |  |
| Cases/person-years | 1997/413482 | 133/22039 | 135/25363 | 117/21038 | 83/14776 | 94/15169 |
| Multivariable model* | 1 (Reference) | 0.95 (0.80-1.14) | **0.80 (0.67-0.96)** | 0.86 (0.71-1.04) | 0.81 (0.65-1.02) | 0.87 (0.70-1.09) |
| **Pancreatitis** |  |  |  |  |  |  |
| Cases/person-years | 82/421370 | 6/22535 | 6/25887 | 5/21543 | 3/15112 | 7/15516 |
| Multivariable model* | 1 (Reference) | 0.98 (0.42-2.29) | 0.78 (0.33-1.84) | 0.77 (0.30-1.96) | 0.57 (0.17-1.89) | 1.2 (0.51-2.84) |
| **NAFLD** |  |  |  |  |  |  |
| Cases/person-years | 204/420994 | 15/22520 | 24/25807 | 16/21515 | 16/15068 | 5/15523 |
| Multivariable model* | 1 (Reference) | 0.97 (0.57-1.66) | 1.26 (0.81-1.96) | 1.08 (0.63-1.84) | 1.36 (0.79-2.34) | **0.35 (0.14-0.87)** |
| **Cirrhosis** |  |  |  |  |  |  |
| Cases/person-years | 98/421462 | 9/22534 | 10/25870 | 4/21547 | 4/15108 | 2/15521 |
| Multivariable model* | 1 (Reference) | 1.05 (0.52-2.11) | 0.87 (0.44-1.73) | 0.4 (0.14-1.12) | 0.49 (0.17-1.40) | **0.20 (0.05-0.84)** |
| **Biliary diseases** |  |  |  |  |  |  |
| Cases/person-years | 634/419139 | 46/22411 | 40/25737 | 45/21409 | 33/15001 | 33/15424 |
| Multivariable model* | 1 (Reference) | 1.08 (0.80-1.47) | 0.79 (0.57-1.09) | 1.13 (0.82-1.55) | 1.14 (0.79-1.65) | 1.05 (0.73-1.53) |
| **Appendicitis** |  |  |  |  |  |  |
| Cases/person-years | 105/421248 | 5/22525 | 5/25875 | 2/21544 | 0/15121 | 3/15520 |
| Multivariable model* | 1 (Reference) | 0.74 (0.30-1.84) | 0.57 (0.23-1.42) | 0.26 (0.06-1.09) | 0 (0-Inf) | 0.46 (0.14-1.51) |
| **Gastrointestinal cancer** |  |  |  |  |  |  |
| Cases/person-years | 375/420746 | 41/22438 | 27/25854 | 33/21463 | 21/15067 | 33/15443 |
| Multivariable model* | 1 (Reference) | 1.44 (1.03-2.00) | 0.78 (0.52-1.17) | 1.11 (0.77-1.62) | 0.94 (0.60-1.50) | 1.41 (0.95-2.08) |
| **Any gastrointestinal diseases** | | | | | | |
| Cases/person-years | 4759/401766 | 298/21346 | 331/24536 | 287/20348 | 199/14304 | 231/14573 |
| Multivariable model* | 1 (Reference) | 0.95 (0.84-1.07) | 0.89 (0.79-1.00) | 0.96 (0.85-1.09) | 0.92 (0.79-1.07) | 1.02 (0.88-1.17) |

Note: *Estimates are hazard ratios (95% confidence intervals), obtained from fully adjusted Cox regression models. GI, gastrointestinal; GERD, Gastroesophageal reflux disease; IBS, Irritable bowel syndrome; NAFLD, Non-alcoholic fatty liver disease.

# Supplementary Table 14. Associations of coffee consumption with incident GI diseases after excluding participants who reported that their diet was not typical on any recall day

| **Outcome** | **Nonconsumers** | **Coffee intake, drinks/d** | | | | |
| --- | --- | --- | --- | --- | --- | --- |
|  |  | **≤ 1** | **1-2** | **2-3** | **3-4** | **> 4** |
| **Unsweetened coffee** |  |  |  |  |  |  |
| **Number of participants** | 25627 | 12423 | 15342 | 11920 | 7895 | 6589 |
| **Barrett’s oesophagus** |  |  |  |  |  |  |
| Cases/person-years | 235/326713 | 102/159573 | 115/197490 | 91/153547 | 57/101762 | 54/84559 |
| Multivariable model* | 1 (Reference) | 0.97 (0.76-1.23) | 0.91 (0.72-1.15) | 0.94 (0.73-1.22) | 0.90 (0.66-1.22) | 1.01 (0.73-1.39) |
| **GERD** |  |  |  |  |  |  |
| Cases/person-years | 1675/318909 | 733/155838 | 821/193719 | 607/150823 | 453/99544 | 370/82729 |
| Multivariable model* | 1 (Reference) | 0.94 (0.86-1.03) | **0.87 (0.80-0.95)** | **0.84 (0.76-0.92)** | 0.95 (0.85-1.06) | 0.91 (0.81-1.03) |
| **Gastritis and duodenitis** |  |  |  |  |  |  |
| Cases/person-years | 1694/317506 | 709/155710 | 769/193380 | 590/150484 | 393/99700 | 317/83057 |
| Multivariable model* | 1 (Reference) | **0.88 (0.80-0.96)** | **0.78 (0.71-0.86)** | **0.78 (0.71-0.86)** | **0.78 (0.69-0.88)** | **0.73 (0.64-0.83)** |
| **Celiac disease** |  |  |  |  |  |  |
| Cases/person-years | 91/327384 | 35/159893 | 46/197828 | 27/153863 | 23/101923 | 12/84754 |
| Multivariable model* | 1 (Reference) | 0.78 (0.52-1.16) | 0.85 (0.58-1.23) | 0.65 (0.41-1.02) | 0.84 (0.52-1.37) | 0.54 (0.28-1.01) |
| **Peptic ulcer** |  |  |  |  |  |  |
| Cases/person-years | 385/325586 | 127/159341 | 141/197261 | 114/153409 | 87/101562 | 74/84469 |
| Multivariable model* | 1 (Reference) | **0.70 (0.57-0.86)** | **0.63 (0.51-0.77)** | **0.65 (0.52-0.81)** | **0.72 (0.56-0.93)** | **0.69 (0.53-0.91)** |
| **Crohn’s disease** |  |  |  |  |  |  |
| Cases/person-years | 45/327729 | 13/160066 | 20/198000 | 15/153965 | 8/102009 | 13/84770 |
| Multivariable model* | 1 (Reference) | 0.74 (0.39-1.39) | 0.97 (0.55-1.69) | 0.97 (0.52-1.80) | 0.77 (0.35-1.7) | 1.45 (0.73-2.89) |
| **Ulcerative colitis** |  |  |  |  |  |  |
| Cases/person-years | 107/327395 | 44/159825 | 49/197792 | 37/153816 | 28/101899 | 25/84705 |
| Multivariable model* | 1 (Reference) | 0.96 (0.67-1.38) | 0.86 (0.60-1.23) | 0.81 (0.54-1.21) | 0.89 (0.57-1.39) | 0.89 (0.55-1.43) |
| **IBS** |  |  |  |  |  |  |
| Cases/person-years | 293/326460 | 133/159440 | 143/197404 | 109/153480 | 62/101767 | 59/84564 |
| Multivariable model* | 1 (Reference) | 0.94 (0.76-1.15) | 0.83 (0.68-1.03) | 0.82 (0.65-1.04) | **0.70 (0.53-0.94)** | 0.77 (0.57-1.04) |
| **Diverticulum** |  |  |  |  |  |  |
| Cases/person-years | 2237/315126 | 1113/153748 | 1235/191081 | 975/148617 | 630/98476 | 517/81930 |
| Multivariable model* | 1 (Reference) | 0.97 (0.90-1.05) | **0.85 (0.79-0.92)** | **0.86 (0.79-0.93)** | **0.82 (0.75-0.91)** | **0.79 (0.71-0.88)** |
| **Pancreatitis** |  |  |  |  |  |  |
| Cases/person-years | 105/327426 | 43/159874 | 48/197902 | 44/153814 | 28/101938 | 33/84663 |
| Multivariable model* | 1 (Reference) | 0.91 (0.63-1.31) | 0.81 (0.57-1.16) | 0.96 (0.66-1.40) | 0.89 (0.57-1.40) | 1.18 (0.76-1.83) |
| **NAFLD** |  |  |  |  |  |  |
| Cases/person-years | 206/327200 | 62/159788 | 76/197791 | 60/153806 | 39/101893 | 46/84658 |
| Multivariable model* | 1 (Reference) | **0.74 (0.55-0.99)** | **0.69 (0.52-0.91)** | **0.65 (0.48-0.88)** | **0.58 (0.4-0.83)** | **0.69 (0.48-0.99)** |
| **Cirrhosis** |  |  |  |  |  |  |
| Cases/person-years | 108/327669 | 40/159964 | 36/197994 | 22/153950 | 19/102003 | 23/84773 |
| Multivariable model* | 1 (Reference) | 0.80 (0.55-1.16) | **0.53 (0.35-0.78)** | **0.38 (0.23-0.62)** | **0.45 (0.27-0.76)** | **0.57 (0.35-0.94)** |
| **Biliary diseases** |  |  |  |  |  |  |
| Cases/person-years | 798/323381 | 308/158320 | 362/196040 | 318/152083 | 224/100730 | 187/83772 |
| Multivariable model* | 1 (Reference) | **0.83 (0.72-0.95)** | **0.78 (0.69-0.89)** | **0.86 (0.75-0.99)** | 0.9 (0.77-1.06) | 0.86 (0.72-1.02) |
| **Appendicitis** |  |  |  |  |  |  |
| Cases/person-years | 154/326921 | 68/159584 | 84/197548 | 66/153582 | 43/101807 | 27/84656 |
| Multivariable model* | 1 (Reference) | 0.91 (0.68-1.21) | 0.9 (0.68-1.19) | 0.91 (0.67-1.24) | 0.89 (0.62-1.28) | 0.66 (0.42-1.02) |
| **Gastrointestinal cancer** |  |  |  |  |  |  |
| Cases/person-years | 453/326352 | 239/159236 | 315/196991 | 247/153096 | 167/101460 | 135/84393 |
| Multivariable model* | 1 (Reference) | 0.96 (0.82-1.13) | 0.98 (0.85-1.15) | 0.98 (0.83-1.15) | 0.98 (0.81-1.18) | 0.93 (0.75-1.15) |
| **Any gastrointestinal diseases** |  |  |  |  |  |  |
| Cases/person-years | 5418/295013 | 2458/145115 | 2827/181396 | 2199/141148 | 1483/93367 | 1233/77768 |
| Multivariable model* | 1 (Reference) | **0.93 (0.88-0.97)** | **0.85 (0.81-0.89)** | **0.85 (0.80-0.89)** | **0.86 (0.81-0.92)** | **0.83 (0.78-0.89)** |
| **Sugar-sweetened coffee** |  |  |  |  |  |  |
| **Number of participants** | 25627 | 4712 | 4158 | 2727 | 1565 | 1357 |
| **Barrett’s oesophagus** |  |  |  |  |  |  |
| Cases/person-years | 235/326713 | 64/59851 | 34/53132 | 21/34837 | 11/19819 | 22/17048 |
| Multivariable model* | 1 (Reference) | 1.32 (0.99-1.76) | 0.80 (0.55-1.17) | 0.78 (0.49-1.24) | 0.69 (0.37-1.30) | 1.65 (1.02-2.68) |
| **GERD** |  |  |  |  |  |  |
| Cases/person-years | 1675/318909 | 332/58246 | 270/51802 | 191/33943 | 107/19250 | 85/16743 |
| Multivariable model* | 1 (Reference) | 1.05 (0.93-1.19) | 0.97 (0.85-1.11) | 1.06 (0.90-1.24) | 1.03 (0.84-1.27) | 0.90 (0.71-1.13) |
| **Gastritis and duodenitis** |  |  |  |  |  |  |
| Cases/person-years | 1694/317506 | 287/58380 | 249/51770 | 142/34118 | 80/19359 | 84/16642 |
| Multivariable model* | 1 (Reference) | **0.85 (0.75-0.97)** | **0.85 (0.74-0.98)** | **0.74 (0.62-0.88)** | **0.72 (0.57-0.90)** | 0.83 (0.66-1.05) |
| **Celiac disease** |  |  |  |  |  |  |
| Cases/person-years | 91/327384 | 16/60069 | 14/53254 | 5/34933 | 0/19879 | 5/17139 |
| Multivariable model* | 1 (Reference) | 0.91 (0.53-1.57) | 0.96 (0.54-1.74) | 0.56 (0.22-1.40) | 0 (0-Inf) | 1.33 (0.51-3.48) |
| **Peptic ulcer** |  |  |  |  |  |  |
| Cases/person-years | 385/325586 | 66/59806 | 51/53012 | 41/34733 | 19/19778 | 20/17106 |
| Multivariable model* | 1 (Reference) | 0.84 (0.64-1.09) | **0.74 (0.54-1.00)** | 0.89 (0.63-1.26) | 0.71 (0.44-1.15) | 0.80 (0.50-1.30) |
| **Crohn’s disease** |  |  |  |  |  |  |
| Cases/person-years | 45/327729 | 12/60082 | 8/53262 | 3/34941 | 2/19862 | 3/17164 |
| Multivariable model* | 1 (Reference) | 1.34 (0.69-2.60) | 1.01 (0.46-2.23) | 0.55 (0.16-1.86) | 0.61 (0.14-2.63) | 0.94 (0.26-3.36) |
| **Ulcerative colitis** |  |  |  |  |  |  |
| Cases/person-years | 107/327395 | 14/60081 | 16/53245 | 14/34868 | 4/19855 | 3/17166 |
| Multivariable model* | 1 (Reference) | 0.65 (0.37-1.15) | 0.79 (0.45-1.36) | 1.00 (0.55-1.82) | 0.47 (0.17-1.31) | 0.35 (0.10-1.15) |
| **IBS** |  |  |  |  |  |  |
| Cases/person-years | 293/326460 | 41/59949 | 49/53060 | 24/34836 | 13/19815 | 9/17146 |
| Multivariable model* | 1 (Reference) | 0.84 (0.60-1.17) | 1.18 (0.86-1.62) | 0.92 (0.60-1.42) | 0.89 (0.50-1.58) | 0.69 (0.34-1.38) |
| **Diverticulum** |  |  |  |  |  |  |
| Cases/person-years | 2237/315126 | 450/57705 | 379/51176 | 240/33536 | 142/19136 | 125/16481 |
| Multivariable model* | 1 (Reference) | 0.98 (0.88-1.08) | 0.91 (0.81-1.02) | 0.88 (0.76-1.01) | 0.88 (0.74-1.05) | 0.91 (0.75-1.10) |
| **Pancreatitis** |  |  |  |  |  |  |
| Cases/person-years | 105/327426 | 31/60049 | 21/53223 | 8/34920 | 6/19852 | 5/17169 |
| Multivariable model* | 1 (Reference) | 1.54 (1.01-2.33) | 1.11 (0.68-1.82) | 0.61 (0.29-1.30) | 0.76 (0.32-1.79) | 0.64 (0.25-1.64) |
| **NAFLD** |  |  |  |  |  |  |
| Cases/person-years | 206/327200 | 44/59964 | 31/53191 | 14/34901 | 15/19822 | 16/17111 |
| Multivariable model* | 1 (Reference) | 1.20 (0.86-1.68) | 0.88 (0.59-1.32) | 0.58 (0.33-1.03) | 0.97 (0.56-1.69) | 1.02 (0.58-1.78) |
| **Cirrhosis** |  |  |  |  |  |  |
| Cases/person-years | 108/327669 | 16/60129 | 17/53285 | 10/34935 | 8/19850 | 9/17139 |
| Multivariable model* | 1 (Reference) | 0.62 (0.36-1.06) | 0.63 (0.37-1.09) | **0.50 (0.25-0.99)** | 0.60 (0.28-1.28) | 0.66 (0.31-1.39) |
| **Biliary diseases** |  |  |  |  |  |  |
| Cases/person-years | 798/323381 | 151/59304 | 117/52701 | 73/34522 | 45/19657 | 37/16984 |
| Multivariable model* | 1 (Reference) | 1.06 (0.88-1.26) | 0.91 (0.74-1.12) | 0.86 (0.66-1.10) | 0.89 (0.65-1.22) | 0.83 (0.58-1.18) |
| **Appendicitis** |  |  |  |  |  |  |
| Cases/person-years | 154/326921 | 28/60009 | 23/53196 | 12/34871 | 4/19857 | 16/17059 |
| Multivariable model* | 1 (Reference) | 1.03 (0.68-1.55) | 0.94 (0.60-1.49) | 0.75 (0.40-1.37) | 0.43 (0.16-1.20) | 1.83 (1.02-3.28) |
| **Gastrointestinal cancer** |  |  |  |  |  |  |
| Cases/person-years | 453/326352 | 92/59859 | 94/52932 | 64/34743 | 31/19777 | 35/17058 |
| Multivariable model* | 1 (Reference) | 0.84 (0.66-1.05) | 0.9 (0.71-1.14) | 0.89 (0.67-1.18) | 0.71 (0.49-1.05) | 0.94 (0.65-1.37) |
| **Any gastrointestinal diseases** |  |  |  |  |  |  |
| Cases/person-years | 5418/295013 | 1041/53905 | 891/47911 | 556/31626 | 315/18053 | 279/15464 |
| Multivariable model* | 1 (Reference) | 0.98 (0.92-1.05) | 0.94 (0.87-1.01) | **0.89 (0.81-0.98)** | **0.86 (0.76-0.97)** | **0.87 (0.76-0.99)** |
| **Artificially sweetened coffee** |  |  |  |  |  |  |
| **Number of participants** | 25627 | 1436 | 1640 | 1302 | 903 | 899 |
| **Barrett’s oesophagus** |  |  |  |  |  |  |
| Cases/person-years | 235/326713 | 27/18131 | 13/20944 | 10/16554 | 8/11464 | 11/11374 |
| Multivariable model* | 1 (Reference) | 1.72 (1.14-2.58) | 0.76 (0.43-1.34) | 0.80 (0.41-1.52) | 0.89 (0.43-1.84) | 1.24 (0.65-2.37) |
| **GERD** |  |  |  |  |  |  |
| Cases/person-years | 1675/318909 | 113/17613 | 115/20366 | 94/16108 | 56/11214 | 77/11033 |
| Multivariable model* | 1 (Reference) | 0.99 (0.81-1.20) | 0.88 (0.72-1.07) | 0.97 (0.78-1.21) | 0.8 (0.61-1.05) | 1.03 (0.81-1.32) |
| **Gastritis and duodenitis** |  |  |  |  |  |  |
| Cases/person-years | 1694/317506 | 114/17540 | 114/20349 | 78/16166 | 63/11181 | 66/11046 |
| Multivariable model* | 1 (Reference) | 0.99 (0.82-1.20) | 0.86 (0.70-1.04) | **0.77 (0.61-0.97)** | 0.85 (0.65-1.10) | 0.86 (0.66-1.11) |
| **Celiac disease** |  |  |  |  |  |  |
| Cases/person-years | 91/327384 | 4/18247 | 2/20997 | 2/16583 | 2/11510 | 3/11429 |
| Multivariable model* | 1 (Reference) | 0.87 (0.32-2.40) | 0.43 (0.11-1.79) | 0.58 (0.14-2.40) | 0.92 (0.22-3.84) | 1.5 (0.45-5.02) |
| **Peptic ulcer** |  |  |  |  |  |  |
| Cases/person-years | 385/325586 | 24/18150 | 29/20847 | 20/16505 | 9/11461 | 18/11355 |
| Multivariable model* | 1 (Reference) | 0.90 (0.59-1.37) | 0.94 (0.64-1.39) | 0.85 (0.53-1.36) | 0.52 (0.26-1.03) | 1.02 (0.61-1.69) |
| **Crohn’s disease** |  |  |  |  |  |  |
| Cases/person-years | 45/327729 | 3/18254 | 4/20978 | 3/16579 | 1/11514 | 1/11443 |
| Multivariable model* | 1 (Reference) | 1.24 (0.38-4.08) | 1.44 (0.49-4.17) | 1.44 (0.43-4.89) | 0.66 (0.09-5.07) | 0.58 (0.07-4.53) |
| **Ulcerative colitis** |  |  |  |  |  |  |
| Cases/person-years | 107/327395 | 6/18238 | 13/20917 | 6/16567 | 4/11500 | 3/11428 |
| Multivariable model* | 1 (Reference) | 0.92 (0.40-2.12) | 1.75 (0.95-3.21) | 1.02 (0.43-2.38) | 0.86 (0.30-2.45) | 0.62 (0.19-2.04) |
| **IBS** |  |  |  |  |  |  |
| Cases/person-years | 293/326460 | 22/18166 | 19/20915 | 21/16489 | 11/11460 | 12/11383 |
| Multivariable model* | 1 (Reference) | 1.11 (0.72-1.73) | 0.82 (0.51-1.31) | 1.22 (0.77-1.93) | 0.89 (0.47-1.65) | 0.88 (0.48-1.63) |
| **Diverticulum** |  |  |  |  |  |  |
| Cases/person-years | 2237/315126 | 170/17355 | 154/20129 | 122/15889 | 95/10917 | 92/10955 |
| Multivariable model* | 1 (Reference) | 1.04 (0.89-1.22) | **0.80 (0.68-0.95)** | **0.81 (0.67-0.98)** | 0.86 (0.69-1.06) | **0.79 (0.64-0.99)** |
| **Pancreatitis** |  |  |  |  |  |  |
| Cases/person-years | 105/327426 | 6/18239 | 10/20962 | 8/16555 | 4/11487 | 7/11419 |
| Multivariable model* | 1 (Reference) | 0.75 (0.32-1.73) | 1.05 (0.53-2.07) | 1.07 (0.50-2.28) | 0.65 (0.23-1.84) | 1.03 (0.44-2.39) |
| **NAFLD** |  |  |  |  |  |  |
| Cases/person-years | 206/327200 | 16/18225 | 27/20863 | 14/16559 | 16/11452 | 7/11426 |
| Multivariable model* | 1 (Reference) | 0.91 (0.54-1.53) | 1.25 (0.82-1.91) | 0.89 (0.51-1.57) | 1.24 (0.72-2.15) | **0.44 (0.20-0.97)** |
| **Cirrhosis** |  |  |  |  |  |  |
| Cases/person-years | 108/327669 | 14/18211 | 12/20961 | 5/16588 | 5/11498 | 3/11438 |
| Multivariable model* | 1 (Reference) | 1.37 (0.77-2.43) | 0.88 (0.47-1.64) | 0.45 (0.18-1.13) | 0.52 (0.20-1.33) | **0.27 (0.08-0.87)** |
| **Biliary diseases** |  |  |  |  |  |  |
| Cases/person-years | 798/323381 | 59/17994 | 45/20722 | 55/16300 | 42/11272 | 45/11205 |
| Multivariable model* | 1 (Reference) | 1.04 (0.79-1.36) | **0.67 (0.49-0.91)** | 1.11 (0.84-1.48) | 1.14 (0.82-1.58) | 1.16 (0.84-1.61) |
| **Appendicitis** |  |  |  |  |  |  |
| Cases/person-years | 154/326921 | 7/18217 | 10/20918 | 8/16532 | 3/11485 | 3/11427 |
| Multivariable model* | 1 (Reference) | 0.83 (0.39-1.79) | 1.02 (0.53-1.97) | 1.03 (0.49-2.16) | 0.54 (0.17-1.75) | 0.52 (0.16-1.70) |
| **Gastrointestinal cancer** |  |  |  |  |  |  |
| Cases/person-years | 453/326352 | 52/18084 | 37/20866 | 30/16486 | 24/11425 | 30/11368 |
| Multivariable model* | 1 (Reference) | 1.43 (1.06-1.91) | 0.85 (0.60-1.20) | 0.82 (0.56-1.21) | 0.88 (0.57-1.35) | 1.07 (0.72-1.61) |
| **Any gastrointestinal diseases** |  |  |  |  |  |  |
| Cases/person-years | 5418/295013 | 378/15960 | 371/18766 | 297/14860 | 215/10190 | 239/10097 |
| Multivariable model* | 1 (Reference) | 1.03 (0.93-1.15) | **0.85 (0.77-0.95)** | **0.89 (0.79-1.00)** | 0.89 (0.77-1.02) | 0.95 (0.83-1.09) |

Note: *Estimates are hazard ratios (95% confidence intervals), obtained from fully adjusted Cox regression models. GI, gastrointestinal; GERD, Gastroesophageal reflux disease; IBS, Irritable bowel syndrome; NAFLD, Non-alcoholic fatty liver disease.

# Supplementary Table 15. Associations of coffee consumption with incident GI diseases after excluding participants who reported coffee consumption at baseline but were classified as non-consumers in their 24-hour dietary recalls.

| **Outcome** | **Nonconsumers** | **Coffee intake, drinks/d** | | | | |
| --- | --- | --- | --- | --- | --- | --- |
|  |  | **≤ 1** | **1-2** | **2-3** | **3-4** | **> 4** |
| **Unsweetened coffee** |  |  |  |  |  |  |
| **Number of participants** | 23640 | 17627 | 22684 | 18561 | 12583 | 11022 |
| **Barrett’s oesophagus** |  |  |  |  |  |  |
| Cases/person-years | 205/301749 | 133/226658 | 160/292328 | 131/239259 | 94/162308 | 87/141762 |
| Multivariable model* | 1 (Reference) | 0.89 (0.71-1.12) | 0.86 (0.69-1.06) | 0.87 (0.68-1.10) | 0.94 (0.72-1.22) | 0.98 (0.75-1.30) |
| **GERD** |  |  |  |  |  |  |
| Cases/person-years | 1563/294380 | 1030/221536 | 1200/286720 | 933/234971 | 708/158973 | 634/138710 |
| Multivariable model* | 1 (Reference) | **0.91 (0.84-0.99)** | **0.84 (0.77-0.91)** | **0.80 (0.73-0.87)** | **0.91 (0.82-1.00)** | 0.92 (0.83-1.02) |
| **Gastritis and duodenitis** |  |  |  |  |  |  |
| Cases/person-years | 1541/293280 | 998/221183 | 1147/286092 | 897/234546 | 624/159154 | 530/139203 |
| Multivariable model* | 1 (Reference) | **0.88 (0.81-0.95)** | **0.8 (0.73-0.86)** | **0.77 (0.70-0.84)** | **0.79 (0.71-0.87)** | **0.75 (0.68-0.84)** |
| **Celiac disease** |  |  |  |  |  |  |
| Cases/person-years | 81/302293 | 56/227012 | 64/292768 | 43/239683 | 33/162603 | 23/142079 |
| Multivariable model* | 1 (Reference) | 0.92 (0.65-1.30) | 0.84 (0.59-1.18) | 0.70 (0.47-1.04) | 0.81 (0.53-1.25) | 0.66 (0.40-1.09) |
| **Peptic ulcer** |  |  |  |  |  |  |
| Cases/person-years | 342/300698 | 177/226312 | 230/291830 | 171/238971 | 138/162032 | 116/141592 |
| Multivariable model* | 1 (Reference) | **0.69 (0.57-0.84)** | **0.69 (0.58-0.83)** | **0.62 (0.51-0.75)** | **0.72 (0.58-0.89)** | **0.65 (0.52-0.82)** |
| **Crohn’s disease** |  |  |  |  |  |  |
| Cases/person-years | 32/302606 | 20/227287 | 30/292987 | 20/239854 | 11/162736 | 24/142086 |
| Multivariable model* | 1 (Reference) | 0.96 (0.54-1.70) | 1.16 (0.69-1.97) | 0.97 (0.53-1.76) | 0.78 (0.38-1.61) | 1.92 (1.06-3.49) |
| **Ulcerative colitis** |  |  |  |  |  |  |
| Cases/person-years | 98/302239 | 59/227018 | 66/292762 | 57/239601 | 47/162533 | 34/142029 |
| Multivariable model* | 1 (Reference) | 0.92 (0.66-1.29) | 0.82 (0.59-1.14) | 0.86 (0.60-1.23) | 1.04 (0.71-1.52) | 0.84 (0.54-1.28) |
| **IBS** |  |  |  |  |  |  |
| Cases/person-years | 289/301266 | 201/226354 | 217/292024 | 173/239032 | 124/162186 | 101/141762 |
| Multivariable model* | 1 (Reference) | 0.96 (0.80-1.16) | **0.83 (0.69-1.00)** | 0.82 (0.67-1.01) | 0.86 (0.69-1.08) | **0.78 (0.61-1.00)** |
| **Diverticulum** |  |  |  |  |  |  |
| Cases/person-years | 1968/291233 | 1541/218549 | 1819/282841 | 1484/231657 | 997/157026 | 860/137434 |
| Multivariable model* | 1 (Reference) | 0.96 (0.90-1.03) | **0.86 (0.81-0.92)** | **0.85 (0.79-0.92)** | **0.84 (0.77-0.91)** | **0.81 (0.74-0.89)** |
| **Pancreatitis** |  |  |  |  |  |  |
| Cases/person-years | 95/302286 | 55/227091 | 84/292729 | 58/239651 | 41/162621 | 44/141968 |
| Multivariable model* | 1 (Reference) | 0.83 (0.59-1.16) | 0.99 (0.72-1.35) | 0.84 (0.59-1.19) | 0.87 (0.59-1.29) | 1.05 (0.70-1.56) |
| **NAFLD** |  |  |  |  |  |  |
| Cases/person-years | 202/302004 | 92/226864 | 110/292676 | 89/239588 | 67/162490 | 71/141919 |
| Multivariable model* | 1 (Reference) | **0.73 (0.56-0.94)** | **0.64 (0.50-0.82)** | **0.59 (0.45-0.78)** | **0.60 (0.45-0.82)** | **0.64 (0.47-0.87)** |
| **Cirrhosis** |  |  |  |  |  |  |
| Cases/person-years | 89/302555 | 57/227173 | 57/292933 | 45/239761 | 32/162723 | 29/142148 |
| Multivariable model* | 1 (Reference) | 0.87 (0.62-1.23) | **0.63 (0.44-0.90)** | **0.57 (0.38-0.84)** | **0.56 (0.36-0.87)** | **0.52 (0.33-0.83)** |
| **Biliary diseases** |  |  |  |  |  |  |
| Cases/person-years | 723/298596 | 436/224804 | 530/290025 | 459/237133 | 344/160745 | 303/140412 |
| Multivariable model* | 1 (Reference) | **0.85 (0.75-0.96)** | **0.80 (0.71-0.90)** | **0.84 (0.74-0.95)** | 0.91 (0.79-1.05) | 0.89 (0.77-1.03) |
| **Appendicitis** |  |  |  |  |  |  |
| Cases/person-years | 149/301758 | 99/226625 | 115/292412 | 91/239311 | 65/162381 | 54/141864 |
| Multivariable model* | 1 (Reference) | 0.89 (0.69-1.16) | 0.81 (0.63-1.05) | 0.78 (0.59-1.04) | 0.82 (0.60-1.12) | 0.76 (0.54-1.07) |
| **Gastrointestinal cancer** |  |  |  |  |  |  |
| Cases/person-years | 384/301459 | 330/226173 | 439/291567 | 366/238581 | 248/161893 | 224/141475 |
| Multivariable model* | 1 (Reference) | 0.99 (0.85-1.15) | 0.99 (0.85-1.14) | 0.99 (0.85-1.16) | 0.98 (0.83-1.17) | 1.01 (0.84-1.21) |
| **Any gastrointestinal diseases** |  |  |  |  |  |  |
| Cases/person-years | 4882/272782 | 3465/206408 | 4155/268499 | 3335/220416 | 2341/148955 | 2054/130379 |
| Multivariable model* | 1 (Reference) | **0.92 (0.88-0.96)** | **0.85 (0.82-0.89)** | **0.83 (0.79-0.87)** | **0.86 (0.82-0.91)** | **0.85 (0.81-0.90)** |
| **Sugar-sweetened coffee** |  |  |  |  |  |  |
| **Number of participants** | 23640 | 6512 | 5963 | 3935 | 2256 | 2013 |
| **Barrett’s oesophagus** |  |  |  |  |  |  |
| Cases/person-years | 205/301749 | 86/82862 | 45/76215 | 35/50242 | 15/28650 | 30/25383 |
| Multivariable model* | 1 (Reference) | 1.29 (0.99-1.68) | 0.73 (0.52-1.02) | 0.88 (0.60-1.29) | 0.63 (0.37-1.09) | 1.47 (0.95-2.25) |
| **GERD** |  |  |  |  |  |  |
| Cases/person-years | 1563/294380 | 452/80730 | 373/74358 | 278/48934 | 147/27866 | 127/24917 |
| Multivariable model* | 1 (Reference) | 1.01 (0.91-1.13) | 0.91 (0.81-1.03) | 1.03 (0.90-1.18) | 0.95 (0.79-1.13) | 0.89 (0.73-1.08) |
| **Gastritis and duodenitis** |  |  |  |  |  |  |
| Cases/person-years | 1541/293280 | 382/80918 | 353/74223 | 221/49102 | 117/27974 | 120/24798 |
| Multivariable model* | 1 (Reference) | **0.83 (0.74-0.93)** | **0.85 (0.75-0.96)** | **0.80 (0.69-0.93)** | **0.73 (0.60-0.89)** | **0.81 (0.66-0.99)** |
| **Celiac disease** |  |  |  |  |  |  |
| Cases/person-years | 81/302293 | 20/83161 | 17/76379 | 5/50409 | 0/28732 | 7/25504 |
| Multivariable model* | 1 (Reference) | 0.90 (0.54-1.50) | 0.91 (0.52-1.57) | 0.43 (0.17-1.10) | 0 (0-Inf) | 1.37 (0.59-3.16) |
| **Peptic ulcer** |  |  |  |  |  |  |
| Cases/person-years | 342/300698 | 81/82846 | 70/76017 | 55/50138 | 28/28576 | 28/25444 |
| Multivariable model* | 1 (Reference) | **0.75 (0.58-0.96)** | **0.70 (0.53-0.92)** | 0.82 (0.60-1.11) | 0.71 (0.47-1.07) | 0.73 (0.48-1.11) |
| **Crohn’s disease** |  |  |  |  |  |  |
| Cases/person-years | 32/302606 | 14/83198 | 15/76381 | 5/50411 | 2/28716 | 3/25541 |
| Multivariable model* | 1 (Reference) | 1.43 (0.74-2.76) | 1.62 (0.83-3.17) | 0.81 (0.30-2.19) | 0.53 (0.12-2.36) | 0.82 (0.23-2.96) |
| **Ulcerative colitis** |  |  |  |  |  |  |
| Cases/person-years | 98/302239 | 20/83175 | 20/76371 | 18/50311 | 4/28708 | 7/25504 |
| Multivariable model* | 1 (Reference) | 0.72 (0.44-1.19) | 0.77 (0.46-1.28) | 1.03 (0.59-1.78) | 0.38 (0.14-1.06) | 0.66 (0.29-1.51) |
| **IBS** |  |  |  |  |  |  |
| Cases/person-years | 289/301266 | 71/82936 | 62/76137 | 41/50248 | 20/28632 | 17/25464 |
| Multivariable model* | 1 (Reference) | 0.99 (0.76-1.30) | 0.99 (0.74-1.32) | 1.03 (0.73-1.46) | 0.91 (0.56-1.46) | 0.84 (0.50-1.41) |
| **Diverticulum** |  |  |  |  |  |  |
| Cases/person-years | 1968/291233 | 624/79804 | 540/73419 | 342/48441 | 205/27653 | 189/24488 |
| Multivariable model* | 1 (Reference) | 1.00 (0.91-1.10) | 0.92 (0.83-1.02) | **0.88 (0.78-0.99)** | 0.89 (0.76-1.03) | 0.94 (0.80-1.10) |
| **Pancreatitis** |  |  |  |  |  |  |
| Cases/person-years | 95/302286 | 36/83143 | 25/76344 | 13/50378 | 8/28693 | 7/25535 |
| Multivariable model* | 1 (Reference) | 1.37 (0.91-2.04) | 1.03 (0.64-1.64) | 0.80 (0.43-1.48) | 0.82 (0.38-1.76) | 0.77 (0.34-1.75) |
| **NAFLD** |  |  |  |  |  |  |
| Cases/person-years | 202/302004 | 53/83053 | 48/76247 | 21/50353 | 22/28650 | 21/25458 |
| Multivariable model* | 1 (Reference) | 0.95 (0.69-1.30) | 0.88 (0.63-1.23) | **0.56 (0.35-0.91)** | 0.92 (0.57-1.48) | 0.86 (0.52-1.41) |
| **Cirrhosis** |  |  |  |  |  |  |
| Cases/person-years | 89/302555 | 24/83232 | 21/76420 | 14/50408 | 10/28702 | 9/25517 |
| Multivariable model* | 1 (Reference) | 0.74 (0.46-1.19) | 0.62 (0.37-1.03) | 0.58 (0.31-1.07) | 0.63 (0.31-1.28) | 0.55 (0.26-1.18) |
| **Biliary diseases** |  |  |  |  |  |  |
| Cases/person-years | 723/298596 | 207/82116 | 160/75587 | 108/49801 | 70/28355 | 51/25281 |
| Multivariable model* | 1 (Reference) | 1.05 (0.89-1.23) | 0.89 (0.74-1.06) | 0.91 (0.73-1.13) | 0.99 (0.76-1.28) | 0.8 (0.59-1.08) |
| **Appendicitis** |  |  |  |  |  |  |
| Cases/person-years | 149/301758 | 42/83040 | 33/76296 | 19/50303 | 6/28695 | 20/25412 |
| Multivariable model* | 1 (Reference) | 1.04 (0.73-1.48) | 0.88 (0.59-1.31) | 0.77 (0.47-1.28) | **0.43 (0.19-1.00)** | 1.55 (0.91-2.62) |
| **Gastrointestinal cancer** |  |  |  |  |  |  |
| Cases/person-years | 384/301459 | 129/82856 | 126/75939 | 93/50109 | 45/28578 | 60/25364 |
| Multivariable model* | 1 (Reference) | 0.90 (0.73-1.10) | 0.89 (0.72-1.10) | 0.97 (0.76-1.25) | 0.77 (0.55-1.07) | 1.18 (0.87-1.60) |
| **Any gastrointestinal diseases** |  |  |  |  |  |  |
| Cases/person-years | 4882/272782 | 1417/74740 | 1240/68960 | 814/45582 | 457/26031 | 413/23076 |
| Multivariable model* | 1 (Reference) | 0.98 (0.92-1.04) | **0.92 (0.86-0.99)** | **0.92 (0.85-0.99)** | **0.88 (0.79-0.97)** | **0.89 (0.79-0.99)** |
| **Artificially sweetened coffee** |  |  |  |  |  |  |
| **Number of participants** | 23640 | 1944 | 2205 | 1820 | 1296 | 1314 |
| **Barrett’s oesophagus** |  |  |  |  |  |  |
| Cases/person-years | 205/301749 | 28/24666 | 16/28200 | 11/23217 | 13/16508 | 15/16707 |
| Multivariable model* | 1 (Reference) | 1.33 (0.89-2.00) | 0.68 (0.4-1.15) | 0.61 (0.33-1.14) | 1.01 (0.56-1.82) | 1.16 (0.65-2.06) |
| **GERD** |  |  |  |  |  |  |
| Cases/person-years | 1563/294380 | 147/23964 | 165/27360 | 135/22546 | 83/16128 | 107/16234 |
| Multivariable model* | 1 (Reference) | 0.92 (0.77-1.09) | 0.88 (0.74-1.04) | 0.93 (0.77-1.12) | **0.79 (0.63-1.00)** | 0.95 (0.77-1.18) |
| **Gastritis and duodenitis** |  |  |  |  |  |  |
| Cases/person-years | 1541/293280 | 144/23877 | 159/27374 | 107/22648 | 94/16053 | 97/16193 |
| Multivariable model* | 1 (Reference) | 0.93 (0.78-1.11) | 0.89 (0.75-1.05) | **0.76 (0.62-0.93)** | 0.92 (0.74-1.14) | 0.91 (0.73-1.13) |
| **Celiac disease** |  |  |  |  |  |  |
| Cases/person-years | 81/302293 | 6/24772 | 3/28274 | 4/23253 | 3/16577 | 3/16780 |
| Multivariable model* | 1 (Reference) | 1.06 (0.45-2.46) | 0.53 (0.16-1.71) | 0.93 (0.33-2.62) | 1.11 (0.34-3.65) | 1.19 (0.36-3.98) |
| **Peptic ulcer** |  |  |  |  |  |  |
| Cases/person-years | 342/300698 | 33/24656 | 42/28072 | 26/23151 | 16/16503 | 22/16670 |
| Multivariable model* | 1 (Reference) | 0.94 (0.65-1.35) | 1.02 (0.73-1.44) | 0.81 (0.54-1.24) | 0.68 (0.40-1.14) | 0.88 (0.55-1.41) |
| **Crohn’s disease** |  |  |  |  |  |  |
| Cases/person-years | 32/302606 | 3/24791 | 4/28258 | 3/23250 | 1/16585 | 1/16793 |
| Multivariable model* | 1 (Reference) | 1.01 (0.30-3.38) | 1.15 (0.38-3.45) | 1.14 (0.32-3.98) | 0.49 (0.06-3.85) | 0.44 (0.06-3.50) |
| **Ulcerative colitis** |  |  |  |  |  |  |
| Cases/person-years | 98/302239 | 8/24754 | 14/28194 | 9/23210 | 5/16567 | 6/16749 |
| Multivariable model* | 1 (Reference) | 0.98 (0.47-2.05) | 1.56 (0.86-2.82) | 1.23 (0.60-2.53) | 0.91 (0.35-2.33) | 1.07 (0.44-2.59) |
| **IBS** |  |  |  |  |  |  |
| Cases/person-years | 289/301266 | 26/24687 | 24/28172 | 24/23138 | 17/16510 | 18/16705 |
| Multivariable model* | 1 (Reference) | 0.92 (0.61-1.38) | 0.71 (0.46-1.09) | 0.93 (0.60-1.44) | 0.89 (0.53-1.49) | 0.87 (0.52-1.45) |
| **Diverticulum** |  |  |  |  |  |  |
| Cases/person-years | 1968/291233 | 201/23725 | 217/27011 | 176/22232 | 138/15747 | 134/16102 |
| Multivariable model* | 1 (Reference) | 0.92 (0.79-1.06) | **0.85 (0.73-0.98)** | **0.84 (0.72-0.99)** | 0.89 (0.74-1.07) | **0.82 (0.68-0.99)** |
| **Pancreatitis** |  |  |  |  |  |  |
| Cases/person-years | 95/302286 | 9/24768 | 11/28231 | 9/23222 | 6/16545 | 9/16766 |
| Multivariable model* | 1 (Reference) | 0.91 (0.45-1.84) | 0.94 (0.49-1.81) | 1.00 (0.48-2.05) | 0.85 (0.35-2.04) | 1.16 (0.54-2.49) |
| **NAFLD** |  |  |  |  |  |  |
| Cases/person-years | 202/302004 | 21/24742 | 36/28098 | 21/23195 | 21/16495 | 10/16768 |
| Multivariable model* | 1 (Reference) | 0.84 (0.53-1.34) | 1.15 (0.79-1.68) | 0.89 (0.55-1.44) | 1.07 (0.66-1.75) | 0.44 (0.22-0.85) |
| **Cirrhosis** |  |  |  |  |  |  |
| Cases/person-years | 89/302555 | 17/24740 | 15/28236 | 6/23259 | 7/16564 | 3/16788 |
| Multivariable model* | 1 (Reference) | 1.37 (0.79-2.35) | 0.90 (0.50-1.63) | 0.43 (0.18-1.02) | 0.61 (0.27-1.39) | 0.22 (0.07-0.73) |
| **Biliary diseases** |  |  |  |  |  |  |
| Cases/person-years | 723/298596 | 77/24434 | 69/27858 | 78/22839 | 58/16248 | 54/16505 |
| Multivariable model* | 1 (Reference) | 0.99 (0.78-1.26) | **0.76 (0.58-0.98)** | 1.12 (0.87-1.43) | 1.11 (0.84-1.48) | 0.97 (0.71-1.31) |
| **Appendicitis** |  |  |  |  |  |  |
| Cases/person-years | 149/301758 | 12/24700 | 12/28182 | 8/23203 | 5/16543 | 5/16772 |
| Multivariable model* | 1 (Reference) | 1.01 (0.55-1.84) | 0.89 (0.48-1.63) | 0.74 (0.35-1.55) | 0.66 (0.26-1.65) | 0.63 (0.25-1.61) |
| **Gastrointestinal cancer** |  |  |  |  |  |  |
| Cases/person-years | 384/301459 | 66/24562 | 52/28102 | 51/23078 | 32/16483 | 42/16675 |
| Multivariable model* | 1 (Reference) | 1.39 (1.06-1.83) | 0.93 (0.69-1.26) | 1.06 (0.78-1.45) | 0.88 (0.60-1.29) | 1.11 (0.78-1.58) |
| **Any gastrointestinal diseases** |  |  |  |  |  |  |
| Cases/person-years | 4882/272782 | 478/21878 | 518/25129 | 424/20754 | 312/14676 | 330/14920 |
| Multivariable model* | 1 (Reference) | 0.96 (0.87-1.06) | **0.89 (0.81-0.98)** | 0.91 (0.82-1.01) | 0.93 (0.82-1.04) | 0.93 (0.82-1.04) |

Note: *Estimates are hazard ratios (95% confidence intervals), obtained from fully adjusted Cox regression models. GI, gastrointestinal; GERD, Gastroesophageal reflux disease; IBS, Irritable bowel syndrome; NAFLD, Non-alcoholic fatty liver disease.

# Supplementary Table 16. Associations of coffee consumption with incident GI diseases, based on their first completed 24-hour dietary recall.

| **Outcome** | **Nonconsumers** | **Coffee intake, drinks/d** | | | | |
| --- | --- | --- | --- | --- | --- | --- |
|  |  | **≤ 1** | **1-2** | **2-3** | **3-4** | **> 4** |
| **Unsweetened coffee** |  |  |  |  |  |  |
| **Number of participants** | 35528 | 26583 | 25155 | 15701 | 8715 | 6323 |
| **Barrett’s oesophagus** |  |  |  |  |  |  |
| Cases/person-years | 309/453611 | 183/342392 | 185/324266 | 121/202495 | 61/112270 | 55/80892 |
| Multivariable model* | 1 (Reference) | 0.87 (0.72-1.05) | 0.95 (0.78-1.15) | 0.97 (0.78-1.22) | 0.91 (0.68-1.22) | 1.09 (0.80-1.49) |
| **GERD** |  |  |  |  |  |  |
| Cases/person-years | 2273/442959 | 1473/335041 | 1292/318343 | 844/198582 | 510/109882 | 386/79062 |
| Multivariable model* | 1 (Reference) | **0.92 (0.86-0.99)** | **0.86 (0.80-0.93)** | **0.89 (0.82-0.97)** | 0.97 (0.88-1.08) | 0.98 (0.87-1.10) |
| **Gastritis and duodenitis** |  |  |  |  |  |  |
| Cases/person-years | 2268/441280 | 1420/334556 | 1237/317663 | 775/198594 | 438/110026 | 326/79340 |
| Multivariable model* | 1 (Reference) | **0.87 (0.81-0.93)** | **0.81 (0.75-0.87)** | **0.81 (0.74-0.88)** | **0.83 (0.74-0.92)** | **0.82 (0.72-0.92)** |
| **Celiac disease** |  |  |  |  |  |  |
| Cases/person-years | 122/454432 | 78/342868 | 74/324767 | 35/202932 | 19/112476 | 13/81102 |
| Multivariable model* | 1 (Reference) | 0.85 (0.63-1.14) | 0.89 (0.66-1.21) | 0.7 (0.47-1.04) | 0.71 (0.43-1.19) | 0.69 (0.38-1.26) |
| **Peptic ulcer** |  |  |  |  |  |  |
| Cases/person-years | 490/452238 | 251/341843 | 243/323795 | 152/202281 | 109/112031 | 77/80787 |
| Multivariable model* | 1 (Reference) | **0.72 (0.61-0.84)** | **0.71 (0.61-0.84)** | **0.68 (0.56-0.83)** | 0.87 (0.69-1.08) | 0.78 (0.60-1.01) |
| **Crohn’s disease** |  |  |  |  |  |  |
| Cases/person-years | 58/454866 | 31/343182 | 29/325046 | 22/203052 | 10/112541 | 13/81127 |
| Multivariable model* | 1 (Reference) | 0.82 (0.53-1.29) | 0.84 (0.52-1.34) | 1.02 (0.60-1.73) | 0.84 (0.41-1.71) | 1.43 (0.74-2.76) |
| **Ulcerative colitis** |  |  |  |  |  |  |
| Cases/person-years | 143/454397 | 86/342842 | 79/324745 | 50/202847 | 30/112428 | 18/81080 |
| Multivariable model* | 1 (Reference) | 0.93 (0.71-1.24) | 0.91 (0.67-1.21) | 0.89 (0.63-1.26) | 0.93 (0.61-1.42) | 0.73 (0.43-1.22) |
| **IBS** |  |  |  |  |  |  |
| Cases/person-years | 411/453049 | 290/341855 | 246/323936 | 144/202410 | 75/112235 | 61/80922 |
| Multivariable model* | 1 (Reference) | 0.97 (0.83-1.13) | 0.89 (0.75-1.05) | 0.83 (0.68-1.02) | 0.77 (0.6-1.00) | 0.83 (0.62-1.11) |
| **Diverticulum** |  |  |  |  |  |  |
| Cases/person-years | 3055/437556 | 2217/330741 | 2001/313915 | 1250/196089 | 722/108375 | 511/78387 |
| Multivariable model* | 1 (Reference) | **0.93 (0.88-0.98)** | **0.86 (0.81-0.91)** | **0.83 (0.77-0.89)** | **0.87 (0.79-0.94)** | **0.81 (0.73-0.89)** |
| **Pancreatitis** |  |  |  |  |  |  |
| Cases/person-years | 143/454454 | 95/342864 | 82/324786 | 52/202939 | 31/112390 | 22/81081 |
| Multivariable model* | 1 (Reference) | 0.98 (0.75-1.28) | 0.88 (0.66-1.17) | 0.85 (0.60-1.19) | 0.88 (0.58-1.34) | 0.80 (0.49-1.29) |
| **NAFLD** |  |  |  |  |  |  |
| Cases/person-years | 296/454047 | 123/342699 | 127/324648 | 74/202844 | 53/112400 | 52/80946 |
| Multivariable model* | 1 (Reference) | **0.68 (0.55-0.85)** | **0.69 (0.55-0.86)** | **0.57 (0.43-0.75)** | **0.65 (0.48-0.89)** | 0.76 (0.55-1.05) |
| **Cirrhosis** |  |  |  |  |  |  |
| Cases/person-years | 140/454818 | 81/343048 | 61/324984 | 36/203034 | 18/112544 | 24/81128 |
| Multivariable model* | 1 (Reference) | 0.84 (0.63-1.12) | **0.59 (0.43-0.81)** | **0.47 (0.32-0.70)** | **0.39 (0.23-0.66)** | **0.62 (0.38-0.99)** |
| **Biliary diseases** |  |  |  |  |  |  |
| Cases/person-years | 1069/449042 | 633/339591 | 601/321630 | 388/200875 | 256/110996 | 194/80027 |
| Multivariable model* | 1 (Reference) | **0.86 (0.77-0.95)** | **0.85 (0.76-0.94)** | **0.84 (0.74-0.95)** | 0.97 (0.84-1.12) | 0.97 (0.82-1.14) |
| **Appendicitis** |  |  |  |  |  |  |
| Cases/person-years | 215/453724 | 139/342333 | 126/324411 | 87/202565 | 48/112262 | 24/81021 |
| Multivariable model* | 1 (Reference) | 0.88 (0.71-1.10) | 0.83 (0.65-1.04) | 0.89 (0.68-1.16) | 0.86 (0.61-1.20) | **0.58 (0.37-0.90)** |
| **Gastrointestinal cancer** |  |  |  |  |  |  |
| Cases/person-years | 606/453054 | 486/341514 | 476/323519 | 325/202016 | 177/111936 | 143/80705 |
| Multivariable model* | 1 (Reference) | 0.97 (0.85-1.09) | 0.96 (0.84-1.09) | 1.00 (0.86-1.16) | 0.99 (0.83-1.19) | 1.07 (0.88-1.31) |
| **Any gastrointestinal diseases** |  |  |  |  |  |  |
| Cases/person-years | 7365/410565 | 5003/313099 | 4528/298614 | 2900/186239 | 1674/102625 | 1245/74080 |
| Multivariable model* | 1 (Reference) | **0.91 (0.88-0.94)** | **0.86 (0.82-0.89)** | **0.86 (0.82-0.90)** | **0.90 (0.85-0.95)** | **0.89 (0.83-0.95)** |
| **Sugar-sweetened coffee** |  |  |  |  |  |  |
| **Number of participants** | 35528 | 8398 | 6027 | 3271 | 1673 | 1310 |
| **Barrett’s oesophagus** |  |  |  |  |  |  |
| Cases/person-years | 309/453611 | 95/107092 | 46/77077 | 39/41618 | 11/21120 | 20/16444 |
| Multivariable model* | 1 (Reference) | 1.19 (0.94-1.51) | 0.81 (0.59-1.12) | 1.27 (0.89-1.82) | 0.70 (0.38-1.31) | 1.63 (1.00-2.67) |
| **GERD** |  |  |  |  |  |  |
| Cases/person-years | 2273/442959 | 569/104329 | 395/75186 | 221/40578 | 111/20544 | 81/16169 |
| Multivariable model* | 1 (Reference) | 1.04 (0.95-1.14) | 1.01 (0.90-1.13) | 1.02 (0.88-1.18) | 1.00 (0.82-1.21) | 0.88 (0.70-1.12) |
| **Gastritis and duodenitis** |  |  |  |  |  |  |
| Cases/person-years | 2268/441280 | 482/104604 | 350/75137 | 189/40604 | 97/20563 | 75/16107 |
| Multivariable model* | 1 (Reference) | **0.85 (0.77-0.94)** | **0.87 (0.77-0.98)** | **0.86 (0.73-1.00)** | 0.85 (0.68-1.05) | **0.78 (0.61-1.00)** |
| **Celiac disease** |  |  |  |  |  |  |
| Cases/person-years | 122/454432 | 22/107437 | 18/77230 | 4/41816 | 0/21177 | 5/16526 |
| Multivariable model* | 1 (Reference) | 0.77 (0.48-1.22) | 0.98 (0.58-1.64) | 0.44 (0.16-1.22) | 0 (0-Inf) | 1.64 (0.63-4.24) |
| **Peptic ulcer** |  |  |  |  |  |  |
| Cases/person-years | 490/452238 | 103/106993 | 75/76890 | 48/41571 | 20/21060 | 16/16508 |
| Multivariable model* | 1 (Reference) | **0.79 (0.64-0.99)** | 0.78 (0.61-1.01) | 0.90 (0.65-1.23) | 0.71 (0.45-1.13) | 0.65 (0.38-1.09) |
| **Crohn’s disease** |  |  |  |  |  |  |
| Cases/person-years | 58/454866 | 23/107430 | 9/77267 | 3/41829 | 1/21172 | 3/16549 |
| Multivariable model* | 1 (Reference) | 1.50 (0.91-2.48) | 0.78 (0.37-1.62) | 0.45 (0.13-1.48) | 0.28 (0.04-2.07) | 0.90 (0.26-3.15) |
| **Ulcerative colitis** |  |  |  |  |  |  |
| Cases/person-years | 143/454397 | 26/107425 | 22/77220 | 16/41714 | 2/21156 | 3/16555 |
| Multivariable model* | 1 (Reference) | 0.74 (0.48-1.13) | 0.82 (0.51-1.32) | 1.05 (0.60-1.82) | **0.24 (0.06-0.97)** | 0.38 (0.11-1.23) |
| **IBS** |  |  |  |  |  |  |
| Cases/person-years | 411/453049 | 88/107115 | 64/77028 | 35/41649 | 14/21112 | 10/16513 |
| Multivariable model* | 1 (Reference) | 1.00 (0.79-1.27) | 1.06 (0.80-1.39) | 1.13 (0.79-1.63) | 0.89 (0.51-1.54) | 0.80 (0.42-1.54) |
| **Diverticulum** |  |  |  |  |  |  |
| Cases/person-years | 3055/437556 | 810/103127 | 524/74334 | 279/40170 | 148/20347 | 139/15826 |
| Multivariable model* | 1 (Reference) | 1.01 (0.93-1.09) | **0.88 (0.80-0.97)** | **0.85 (0.74-0.97)** | 0.87 (0.73-1.03) | 1.04 (0.87-1.25) |
| **Pancreatitis** |  |  |  |  |  |  |
| Cases/person-years | 143/454454 | 45/107378 | 25/77212 | 6/41819 | 9/21131 | 4/16554 |
| Multivariable model* | 1 (Reference) | 1.27 (0.90-1.80) | 0.94 (0.60-1.48) | 0.38 (0.17-0.89) | 1.06 (0.52-2.16) | 0.53 (0.19-1.50) |
| **NAFLD** |  |  |  |  |  |  |
| Cases/person-years | 296/454047 | 64/107285 | 48/77116 | 17/41767 | 20/21110 | 16/16484 |
| Multivariable model* | 1 (Reference) | 0.96 (0.72-1.26) | 0.94 (0.68-1.30) | **0.54 (0.33-0.90)** | 1.12 (0.69-1.81) | 0.99 (0.57-1.70) |
| **Cirrhosis** |  |  |  |  |  |  |
| Cases/person-years | 140/454818 | 34/107495 | 18/77293 | 11/41800 | 10/21155 | 5/16537 |
| Multivariable model* | 1 (Reference) | 0.81 (0.55-1.20) | **0.52 (0.31-0.86)** | **0.47 (0.24-0.89)** | 0.76 (0.38-1.51) | 0.40 (0.16-1.02) |
| **Biliary diseases** |  |  |  |  |  |  |
| Cases/person-years | 1069/449042 | 258/106117 | 164/76419 | 84/41338 | 59/20884 | 31/16382 |
| Multivariable model* | 1 (Reference) | 1.07 (0.93-1.23) | 0.94 (0.79-1.12) | 0.86 (0.68-1.09) | 1.16 (0.88-1.52) | 0.77 (0.53-1.12) |
| **Appendicitis** |  |  |  |  |  |  |
| Cases/person-years | 215/453724 | 57/107240 | 24/77195 | 15/41734 | 7/21134 | 17/16441 |
| Multivariable model* | 1 (Reference) | 1.13 (0.83-1.53) | **0.65 (0.42-1.00)** | 0.74 (0.43-1.29) | 0.67 (0.31-1.47) | 1.98 (1.14-3.43) |
| **Gastrointestinal cancer** |  |  |  |  |  |  |
| Cases/person-years | 606/453054 | 173/106961 | 126/76804 | 72/41591 | 43/21043 | 39/16447 |
| Multivariable model* | 1 (Reference) | 0.93 (0.78-1.11) | 0.88 (0.72-1.08) | 0.88 (0.68-1.14) | 0.99 (0.71-1.37) | 1.13 (0.80-1.61) |
| **Any gastrointestinal diseases** |  |  |  |  |  |  |
| Cases/person-years | 7365/410565 | 1827/96705 | 1221/69947 | 658/37730 | 345/19107 | 290/14900 |
| Multivariable model* | 1 (Reference) | 1.00 (0.95-1.05) | **0.91 (0.86-0.97)** | **0.90 (0.83-0.98)** | 0.90 (0.81-1.01) | 0.95 (0.84-1.07) |
| **Artificially sweetened coffee** |  |  |  |  |  |  |
| **Number of participants** | 35528 | 2555 | 2402 | 1593 | 1061 | 968 |
| **Barrett’s oesophagus** |  |  |  |  |  |  |
| Cases/person-years | 309/453611 | 36/32487 | 14/30697 | 11/20339 | 11/13544 | 11/12233 |
| Multivariable model* | 1 (Reference) | 1.35 (0.95-1.92) | **0.57 (0.33-0.99)** | 0.71 (0.38-1.32) | 1.05 (0.56-1.96) | 1.19 (0.63-2.25) |
| **GERD** |  |  |  |  |  |  |
| Cases/person-years | 2273/442959 | 195/31546 | 190/29709 | 108/19810 | 71/13227 | 73/11938 |
| Multivariable model* | 1 (Reference) | 0.98 (0.84-1.14) | 1.00 (0.86-1.16) | 0.89 (0.73-1.09) | 0.84 (0.66-1.07) | 0.92 (0.72-1.17) |
| **Gastritis and duodenitis** |  |  |  |  |  |  |
| Cases/person-years | 2268/441280 | 185/31501 | 164/29780 | 107/19787 | 70/13215 | 75/11861 |
| Multivariable model* | 1 (Reference) | 0.96 (0.82-1.12) | 0.89 (0.76-1.05) | 0.91 (0.75-1.11) | 0.86 (0.67-1.10) | 1.01 (0.79-1.28) |
| **Celiac disease** |  |  |  |  |  |  |
| Cases/person-years | 122/454432 | 6/32628 | 4/30775 | 2/20375 | 3/13601 | 4/12277 |
| Multivariable model* | 1 (Reference) | 0.81 (0.35-1.86) | 0.66 (0.24-1.81) | 0.56 (0.14-2.30) | 1.41 (0.43-4.57) | 2.32 (0.82-6.61) |
| **Peptic ulcer** |  |  |  |  |  |  |
| Cases/person-years | 490/452238 | 41/32462 | 42/30583 | 26/20262 | 13/13535 | 17/12209 |
| Multivariable model* | 1 (Reference) | 0.95 (0.69-1.32) | 1.01 (0.73-1.40) | 0.97 (0.64-1.46) | 0.7 (0.39-1.23) | 0.99 (0.59-1.65) |
| **Crohn’s disease** |  |  |  |  |  |  |
| Cases/person-years | 58/454866 | 4/32640 | 3/30766 | 3/20371 | 2/13603 | 0/12298 |
| Multivariable model* | 1 (Reference) | 0.93 (0.33-2.61) | 0.69 (0.21-2.29) | 1.06 (0.32-3.54) | 0.98 (0.23-4.26) | 0 (0-Inf) |
| **Ulcerative colitis** |  |  |  |  |  |  |
| Cases/person-years | 143/454397 | 12/32580 | 13/30702 | 10/20341 | 4/13581 | 3/12270 |
| Multivariable model* | 1 (Reference) | 1.13 (0.62-2.07) | 1.29 (0.71-2.34) | 1.50 (0.76-2.94) | 0.85 (0.30-2.38) | 0.66 (0.20-2.17) |
| **IBS** |  |  |  |  |  |  |
| Cases/person-years | 411/453049 | 31/32519 | 30/30628 | 18/20307 | 18/13529 | 12/12230 |
| Multivariable model* | 1 (Reference) | 0.88 (0.61-1.27) | 0.88 (0.60-1.30) | 0.85 (0.52-1.38) | 1.2 (0.73-1.97) | 0.87 (0.48-1.59) |
| **Diverticulum** |  |  |  |  |  |  |
| Cases/person-years | 3055/437556 | 264/31188 | 234/29418 | 158/19471 | 111/12943 | 99/11798 |
| Multivariable model* | 1 (Reference) | 0.92 (0.81-1.04) | **0.83 (0.73-0.96)** | **0.85 (0.72-1.00)** | 0.84 (0.69-1.03) | **0.81 (0.66-1.00)** |
| **Pancreatitis** |  |  |  |  |  |  |
| Cases/person-years | 143/454454 | 11/32611 | 12/30722 | 10/20346 | 3/13583 | 8/12269 |
| Multivariable model* | 1 (Reference) | 0.82 (0.44-1.53) | 0.89 (0.48-1.64) | 1.12 (0.57-2.20) | 0.44 (0.14-1.42) | 1.23 (0.57-2.67) |
| **NAFLD** |  |  |  |  |  |  |
| Cases/person-years | 296/454047 | 26/32579 | 37/30589 | 26/20303 | 13/13553 | 7/12273 |
| Multivariable model* | 1 (Reference) | 0.81 (0.54-1.23) | 1.1 (0.77-1.58) | 1.24 (0.81-1.89) | 0.76 (0.43-1.37) | 0.40 (0.19-0.87) |
| **Cirrhosis** |  |  |  |  |  |  |
| Cases/person-years | 140/454818 | 19/32587 | 15/30733 | 5/20378 | 5/13598 | 4/12290 |
| Multivariable model* | 1 (Reference) | 1.15 (0.70-1.89) | 0.8 (0.46-1.40) | **0.36 (0.14-0.91)** | 0.47 (0.19-1.19) | **0.35 (0.12-0.98)** |
| **Biliary diseases** |  |  |  |  |  |  |
| Cases/person-years | 1069/449042 | 102/32125 | 80/30274 | 67/20013 | 46/13368 | 41/12105 |
| Multivariable model* | 1 (Reference) | 1.05 (0.85-1.29) | 0.85 (0.68-1.08) | 1.13 (0.88-1.47) | 1.09 (0.8-1.48) | 1.06 (0.76-1.48) |
| **Appendicitis** |  |  |  |  |  |  |
| Cases/person-years | 215/453724 | 13/32548 | 12/30683 | 8/20327 | 5/13566 | 4/12276 |
| Multivariable model* | 1 (Reference) | 0.81 (0.46-1.43) | 0.77 (0.42-1.4) | 0.78 (0.37-1.60) | 0.70 (0.28-1.74) | 0.59 (0.21-1.64) |
| **Gastrointestinal cancer** |  |  |  |  |  |  |
| Cases/person-years | 606/453054 | 81/32397 | 62/30527 | 37/20276 | 28/13501 | 35/12200 |
| Multivariable model* | 1 (Reference) | 1.31 (1.03-1.67) | 1 (0.76-1.32) | 0.86 (0.61-1.21) | 0.91 (0.61-1.35) | 1.24 (0.85-1.79) |
| **Any gastrointestinal diseases** |  |  |  |  |  |  |
| Cases/person-years | 7365/410565 | 624/28839 | 563/27278 | 374/18192 | 250/12115 | 251/10932 |
| Multivariable model* | 1 (Reference) | 0.97 (0.89-1.06) | **0.91 (0.83-0.99)** | 0.92 (0.82-1.02) | 0.88 (0.78-1.01) | 0.96 (0.84-1.09) |

Note: *Estimates are hazard ratios (95% confidence intervals), obtained from fully adjusted Cox regression models. GI, gastrointestinal; GERD, Gastroesophageal reflux disease; IBS, Irritable bowel syndrome; NAFLD, Non-alcoholic fatty liver disease.

**Supplementary Table 17. Associations of coffee consumption with incident GI diseases after excluding high-risk participants who simultaneously suffered from at least three chronic diseases.**

| **Outcome** | **Nonconsumers** | **Coffee intake, drinks/d** | | | | |
| --- | --- | --- | --- | --- | --- | --- |
|  |  | **≤ 1** | **1-2** | **2-3** | **3-4** | **> 4** |
| **Unsweetened coffee** | | | | | | |
| **Number of participants** | 31221 | 15820 | 20418 | 16820 | 11346 | 9873 |
| **Barrett’s oesophagus** |  |  |  |  |  |  |
| Cases/person-years | 236/399928 | 108/203834 | 131/263745 | 102/217330 | 77/146673 | 69/127348 |
| Multivariable model* | 1 (Reference) | 0.93 (0.74-1.18) | 0.89 (0.71-1.12) | 0.83 (0.65-1.07) | 0.94 (0.72-1.24) | 0.96 (0.72-1.29) |
| **GERD** |  |  |  |  |  |  |
| Cases/person-years | 1800/391506 | 828/199695 | 949/259378 | 762/213848 | 604/143876 | 520/124765 |
| Multivariable model* | 1 (Reference) | 0.93 (0.85-1.01) | **0.84 (0.77-0.91)** | **0.81 (0.74-0.88)** | 0.97 (0.87-1.07) | 0.95 (0.86-1.06) |
| **Gastritis and duodenitis** |  |  |  |  |  |  |
| Cases/person-years | 1794/390227 | 811/199372 | 941/258696 | 744/213418 | 522/144086 | 428/125252 |
| Multivariable model* | 1 (Reference) | **0.89 (0.82-0.97)** | **0.81 (0.74-0.88)** | **0.78 (0.71-0.85)** | **0.81 (0.73-0.90)** | **0.76 (0.68-0.85)** |
| **Celiac disease** |  |  |  |  |  |  |
| Cases/person-years | 101/400521 | 50/204071 | 52/264125 | 37/217631 | 29/146912 | 17/127613 |
| Multivariable model* | 1 (Reference) | 0.94 (0.67-1.33) | 0.78 (0.55-1.10) | 0.69 (0.46-1.03) | 0.83 (0.53-1.28) | **0.58 (0.34-0.99)** |
| **Peptic ulcer** |  |  |  |  |  |  |
| Cases/person-years | 384/398894 | 151/203461 | 182/263399 | 140/217050 | 116/146454 | 95/127185 |
| Multivariable model* | 1 (Reference) | **0.77 (0.63-0.93)** | **0.71 (0.59-0.85)** | **0.64 (0.52-0.79)** | **0.78 (0.62-0.97)** | **0.70 (0.55-0.89)** |
| **Crohn’s disease** |  |  |  |  |  |  |
| Cases/person-years | 48/400888 | 18/204316 | 23/264311 | 16/217786 | 9/147037 | 19/127599 |
| Multivariable model* | 1 (Reference) | 0.84 (0.48-1.46) | 0.84 (0.50-1.41) | 0.70 (0.38-1.27) | 0.57 (0.27-1.20) | 1.32 (0.73-2.39) |
| **Ulcerative colitis** |  |  |  |  |  |  |
| Cases/person-years | 116/400486 | 47/204111 | 59/264063 | 49/217548 | 41/146842 | 32/127533 |
| Multivariable model* | 1 (Reference) | 0.93 (0.66-1.32) | 0.92 (0.66-1.28) | 0.94 (0.65-1.34) | 1.14 (0.78-1.68) | 1.01 (0.66-1.55) |
| **IBS** |  |  |  |  |  |  |
| Cases/person-years | 296/399613 | 167/203540 | 173/263507 | 138/217144 | 92/146637 | 80/127336 |
| Multivariable model* | 1 (Reference) | 1.09 (0.90-1.33) | 0.90 (0.74-1.09) | 0.87 (0.70-1.08) | 0.86 (0.67-1.10) | 0.85 (0.65-1.12) |
| **Diverticulum** |  |  |  |  |  |  |
| Cases/person-years | 2479/386926 | 1264/197206 | 1512/255970 | 1266/210871 | 825/142387 | 723/123709 |
| Multivariable model* | 1 (Reference) | **0.93 (0.87-1.00)** | **0.85 (0.79-0.90)** | **0.84 (0.79-0.91)** | **0.81 (0.74-0.88)** | **0.80 (0.74-0.88)** |
| **Pancreatitis** |  |  |  |  |  |  |
| Cases/person-years | 108/400619 | 41/204180 | 71/264073 | 49/217602 | 35/146931 | 32/127517 |
| Multivariable model* | 1 (Reference) | 0.78 (0.54-1.13) | 1.06 (0.77-1.46) | 0.88 (0.62-1.27) | 0.93 (0.62-1.39) | 0.95 (0.62-1.46) |
| **NAFLD** |  |  |  |  |  |  |
| Cases/person-years | 201/400402 | 69/203981 | 82/264095 | 73/217572 | 49/146843 | 56/127474 |
| Multivariable model* | 1 (Reference) | 0.78 (0.59-1.03) | **0.68 (0.52-0.89)** | **0.67 (0.5-0.90)** | **0.62 (0.44-0.87)** | **0.72 (0.52-1.00)** |
| **Cirrhosis** |  |  |  |  |  |  |
| Cases/person-years | 98/400938 | 51/204226 | 45/264241 | 27/217782 | 20/147031 | 22/127654 |
| Multivariable model* | 1 (Reference) | 1.01 (0.71-1.44) | **0.64 (0.44-0.92)** | **0.42 (0.27-0.66)** | **0.43 (0.26-0.71)** | **0.48 (0.29-0.80)** |
| **Biliary diseases** |  |  |  |  |  |  |
| Cases/person-years | 858/396288 | 360/202223 | 425/261881 | 388/215431 | 288/145367 | 250/126223 |
| Multivariable model* | 1 (Reference) | **0.86 (0.76-0.97)** | **0.78 (0.69-0.88)** | **0.85 (0.74-0.96)** | 0.92 (0.79-1.06) | 0.89 (0.76-1.04) |
| **Appendicitis** |  |  |  |  |  |  |
| Cases/person-years | 183/399955 | 90/203697 | 105/263753 | 82/217282 | 61/146695 | 45/127419 |
| Multivariable model* | 1 (Reference) | 0.98 (0.76-1.27) | 0.89 (0.69-1.14) | 0.83 (0.63-1.10) | 0.91 (0.67-1.24) | 0.76 (0.53-1.08) |
| **Gastrointestinal cancer** |  |  |  |  |  |  |
| Cases/person-years | 500/399391 | 274/203372 | 384/263043 | 314/216677 | 216/146285 | 181/127098 |
| Multivariable model* | 1 (Reference) | 0.95 (0.82-1.10) | 1 (0.87-1.15) | 0.97 (0.84-1.13) | 0.98 (0.83-1.17) | 0.94 (0.78-1.14) |
| **Any gastrointestinal diseases** | | | | | | |
| Cases/person-years | 5990/365134 | 2878/187046 | 3460/244128 | 2833/201341 | 1989/135473 | 1716/117786 |
| Multivariable model* | 1 (Reference) | **0.92 (0.88-0.97)** | **0.85 (0.81-0.89)** | **0.84 (0.80-0.88)** | **0.88 (0.83-0.92)** | **0.86 (0.81-0.91)** |
| **Sugar-sweetened coffee** | | | | | | |
| **Number of participants** | 31221 | 5818 | 5379 | 3543 | 2057 | 1824 |
| **Barrett’s oesophagus** |  |  |  |  |  |  |
| Cases/person-years | 236/399928 | 70/74184 | 43/68886 | 30/45333 | 15/26159 | 27/23024 |
| Multivariable model* | 1 (Reference) | 1.45 (1.10-1.91) | 0.94 (0.67-1.33) | 1.02 (0.69-1.53) | 0.86 (0.50-1.48) | 1.84 (1.19-2.86) |
| **GERD** |  |  |  |  |  |  |
| Cases/person-years | 1800/391506 | 363/72457 | 309/67368 | 234/44259 | 123/25537 | 102/22671 |
| Multivariable model* | 1 (Reference) | 1.03 (0.92-1.15) | 0.94 (0.83-1.06) | 1.08 (0.94-1.25) | 0.96 (0.79-1.16) | 0.9 (0.72-1.11) |
| **Gastritis and duodenitis** |  |  |  |  |  |  |
| Cases/person-years | 1794/390227 | 299/72644 | 296/67227 | 183/44403 | 97/25607 | 96/22605 |
| Multivariable model* | 1 (Reference) | **0.81 (0.71-0.91)** | **0.87 (0.76-0.99)** | **0.81 (0.69-0.95)** | **0.72 (0.58-0.89)** | **0.79 (0.63-0.98)** |
| **Celiac disease** |  |  |  |  |  |  |
| Cases/person-years | 101/400521 | 16/74431 | 13/69063 | 5/45465 | 0/26242 | 6/23142 |
| Multivariable model* | 1 (Reference) | 0.85 (0.5-1.46) | 0.8 (0.44-1.46) | 0.5 (0.2-1.26) | 0 (0-Inf) | 1.36 (0.56-3.27) |
| **Peptic ulcer** |  |  |  |  |  |  |
| Cases/person-years | 384/398894 | 60/74189 | 56/68768 | 47/45244 | 24/26110 | 25/23081 |
| Multivariable model* | 1 (Reference) | **0.7 (0.53-0.93)** | **0.69 (0.52-0.93)** | 0.86 (0.62-1.19) | 0.73 (0.47-1.12) | 0.81 (0.53-1.26) |
| **Crohn’s disease** |  |  |  |  |  |  |
| Cases/person-years | 48/400888 | 13/74456 | 15/69044 | 5/45467 | 2/26225 | 2/23173 |
| Multivariable model* | 1 (Reference) | 1.28 (0.68-2.41) | 1.52 (0.82-2.84) | 0.74 (0.28-1.95) | 0.49 (0.11-2.09) | 0.5 (0.11-2.21) |
| **Ulcerative colitis** |  |  |  |  |  |  |
| Cases/person-years | 116/400486 | 19/74436 | 19/69039 | 17/45372 | 3/26222 | 5/23154 |
| Multivariable model* | 1 (Reference) | 0.84 (0.51-1.39) | 0.87 (0.52-1.45) | 1.15 (0.67-1.99) | 0.33 (0.1-1.08) | 0.53 (0.21-1.38) |
| **IBS** |  |  |  |  |  |  |
| Cases/person-years | 296/399613 | 55/74288 | 54/68836 | 33/45333 | 18/26157 | 13/23117 |
| Multivariable model* | 1 (Reference) | 1.06 (0.79-1.42) | 1.17 (0.87-1.59) | 1.13 (0.77-1.64) | 1.09 (0.66-1.79) | 0.92 (0.51-1.65) |
| **Diverticulum** |  |  |  |  |  |  |
| Cases/person-years | 2479/386926 | 521/71602 | 469/66523 | 287/43829 | 172/25336 | 163/22257 |
| Multivariable model* | 1 (Reference) | 1.00 (0.91-1.10) | 0.93 (0.84-1.04) | 0.86 (0.76-0.98) | 0.85 (0.72-1) | 0.93 (0.79-1.11) |
| **Pancreatitis** |  |  |  |  |  |  |
| Cases/person-years | 108/400619 | 29/74422 | 21/69035 | 11/45442 | 6/26215 | 4/23177 |
| Multivariable model* | 1 (Reference) | 1.36 (0.89-2.08) | 1.01 (0.62-1.66) | 0.78 (0.41-1.49) | 0.68 (0.29-1.59) | 0.46 (0.16-1.31) |
| **NAFLD** |  |  |  |  |  |  |
| Cases/person-years | 201/400402 | 41/74361 | 33/68986 | 12/45446 | 17/26189 | 19/23095 |
| Multivariable model* | 1 (Reference) | 1.06 (0.75-1.5) | 0.85 (0.58-1.25) | **0.44 (0.24-0.81)** | 0.98 (0.58-1.65) | 1.11 (0.66-1.87) |
| **Cirrhosis** |  |  |  |  |  |  |
| Cases/person-years | 98/400938 | 15/74496 | 15/69094 | 9/45471 | 6/26222 | 7/23152 |
| Multivariable model* | 1 (Reference) | 0.6 (0.35-1.05) | **0.53 (0.30-0.95)** | **0.43 (0.21-0.88)** | 0.44 (0.18-1.03) | 0.49 (0.21-1.13) |
| **Biliary diseases** |  |  |  |  |  |  |
| Cases/person-years | 858/396288 | 173/73572 | 134/68386 | 83/44983 | 61/25928 | 43/22945 |
| Multivariable model* | 1 (Reference) | 1.08 (0.91-1.28) | 0.9 (0.74-1.09) | 0.84 (0.66-1.06) | 1.02 (0.78-1.35) | 0.81 (0.58-1.12) |
| **Appendicitis** |  |  |  |  |  |  |
| Cases/person-years | 183/399955 | 36/74323 | 27/68975 | 19/45359 | 6/26204 | 18/23060 |
| Multivariable model* | 1 (Reference) | 1.08 (0.75-1.55) | 0.86 (0.57-1.31) | 0.91 (0.56-1.50) | 0.50 (0.22-1.14) | 1.62 (0.95-2.78) |
| **Gastrointestinal cancer** |  |  |  |  |  |  |
| Cases/person-years | 500/399391 | 104/74152 | 114/68664 | 81/45210 | 41/26095 | 53/23030 |
| Multivariable model* | 1 (Reference) | 0.87 (0.70-1.08) | 0.95 (0.76-1.17) | 0.99 (0.77-1.27) | 0.82 (0.59-1.15) | 1.2 (0.88-1.64) |
| **Any gastrointestinal diseases** | | | | | | |
| Cases/person-years | 5990/365134 | 1165/67464 | 1076/62659 | 691/41415 | 391/23968 | 350/21115 |
| Multivariable model* | 1 (Reference) | 0.97 (0.91-1.04) | 0.95 (0.89-1.02) | 0.92 (0.85-1) | 0.87 (0.78-0.97) | 0.88 (0.79-0.99) |
| **Artificially sweetened coffee** | | | | | | |
| **Number of participants** | 31221 | 1569 | 1795 | 1505 | 1053 | 1057 |
| **Barrett’s oesophagus** |  |  |  |  |  |  |
| Cases/person-years | 236/399928 | 21/20052 | 10/23119 | 9/19287 | 10/13486 | 10/13528 |
| Multivariable model* | 1 (Reference) | 1.52 (0.96-2.40) | 0.63 (0.33-1.20) | 0.71 (0.36-1.40) | 1.11 (0.57-2.15) | 1.13 (0.58-2.2) |
| **GERD** |  |  |  |  |  |  |
| Cases/person-years | 1800/391506 | 111/19515 | 108/22606 | 104/18806 | 53/13235 | 76/13166 |
| Multivariable model* | 1 (Reference) | 1.03 (0.85-1.25) | 0.84 (0.69-1.03) | 1 (0.81-1.22) | **0.72 (0.54-0.95)** | 1 (0.79-1.28) |
| **Gastritis and duodenitis** |  |  |  |  |  |  |
| Cases/person-years | 1794/390227 | 105/19461 | 115/22522 | 84/18862 | 72/13158 | 70/13129 |
| Multivariable model* | 1 (Reference) | 1.01 (0.82-1.23) | 0.94 (0.78-1.14) | 0.84 (0.67-1.05) | 1.00 (0.79-1.28) | 0.96 (0.75-1.24) |
| **Celiac disease** |  |  |  |  |  |  |
| Cases/person-years | 101/400521 | 5/20143 | 3/23165 | 4/19313 | 3/13537 | 2/13567 |
| Multivariable model* | 1 (Reference) | 1.19 (0.48-2.95) | 0.71 (0.22-2.28) | 1.23 (0.44-3.41) | 1.45 (0.44-4.7) | 1.08 (0.26-4.55) |
| **Peptic ulcer** |  |  |  |  |  |  |
| Cases/person-years | 384/398894 | 21/20079 | 26/23044 | 22/19238 | 13/13491 | 19/13473 |
| Multivariable model* | 1 (Reference) | 0.9 (0.58-1.40) | 0.95 (0.63-1.43) | 0.97 (0.62-1.52) | 0.79 (0.45-1.40) | 1.14 (0.7-1.86) |
| **Crohn’s disease** |  |  |  |  |  |  |
| Cases/person-years | 48/400888 | 2/20157 | 1/23171 | 2/19312 | 1/13545 | 1/13576 |
| Multivariable model* | 1 (Reference) | 0.89 (0.21-3.73) | 0.38 (0.05-2.81) | 0.87 (0.20-3.74) | 0.56 (0.07-4.29) | 0.50 (0.06-3.99) |
| **Ulcerative colitis** |  |  |  |  |  |  |
| Cases/person-years | 116/400486 | 7/20121 | 10/23119 | 8/19274 | 4/13528 | 5/13534 |
| Multivariable model* | 1 (Reference) | 1.22 (0.56-2.66) | 1.53 (0.78-3.00) | 1.53 (0.72-3.24) | 1.02 (0.36-2.86) | 1.21 (0.47-3.14) |
| **IBS** |  |  |  |  |  |  |
| Cases/person-years | 296/399613 | 20/20069 | 18/23105 | 16/19236 | 10/13508 | 12/13526 |
| Multivariable model* | 1 (Reference) | 1.19 (0.75-1.88) | 0.92 (0.56-1.49) | 0.99 (0.59-1.67) | 0.9 (0.47-1.73) | 1.06 (0.57-1.95) |
| **Diverticulum** |  |  |  |  |  |  |
| Cases/person-years | 2479/386926 | 142/19400 | 154/22236 | 126/18603 | 95/12941 | 100/13074 |
| Multivariable model* | 1 (Reference) | 0.88 (0.74-1.04) | **0.81 (0.69-0.96)** | **0.78 (0.65-0.94)** | 0.82 (0.66-1.01) | 0.84 (0.68-1.04) |
| **Pancreatitis** |  |  |  |  |  |  |
| Cases/person-years | 108/400619 | 4/20151 | 4/23159 | 7/19294 | 5/13514 | 7/13552 |
| Multivariable model* | 1 (Reference) | 0.59 (0.22-1.63) | 0.49 (0.18-1.35) | 1.02 (0.46-2.26) | 0.9 (0.35-2.31) | 1.20 (0.52-2.75) |
| **NAFLD** |  |  |  |  |  |  |
| Cases/person-years | 201/400402 | 14/20128 | 22/23053 | 17/19267 | 13/13486 | 5/13571 |
| Multivariable model* | 1 (Reference) | 0.94 (0.54-1.62) | 1.19 (0.75-1.88) | 1.15 (0.68-1.94) | 1.08 (0.59-1.96) | **0.38 (0.15-0.96)** |
| **Cirrhosis** |  |  |  |  |  |  |
| Cases/person-years | 98/400938 | 11/20116 | 7/23153 | 6/19319 | 5/13532 | 2/13578 |
| Multivariable model* | 1 (Reference) | 1.36 (0.72-2.59) | 0.62 (0.28-1.38) | 0.57 (0.24-1.35) | 0.58 (0.23-1.50) | 0.21 (0.05-0.88) |
| **Biliary diseases** |  |  |  |  |  |  |
| Cases/person-years | 858/396288 | 56/19861 | 46/22858 | 58/19006 | 45/13263 | 42/13371 |
| Multivariable model* | 1 (Reference) | 1.04 (0.79-1.37) | 0.71 (0.52-0.96) | 1.13 (0.86-1.49) | 1.22 (0.89-1.68) | 1.08 (0.78-1.50) |
| **Appendicitis** |  |  |  |  |  |  |
| Cases/person-years | 183/399955 | 9/20088 | 10/23092 | 7/19275 | 4/13509 | 3/13569 |
| Multivariable model* | 1 (Reference) | 0.94 (0.48-1.86) | 0.89 (0.46-1.71) | 0.75 (0.35-1.63) | 0.61 (0.22-1.67) | 0.44 (0.14-1.40) |
| **Gastrointestinal cancer** |  |  |  |  |  |  |
| Cases/person-years | 500/399391 | 46/19988 | 39/23013 | 46/19161 | 29/13455 | 29/13499 |
| Multivariable model* | 1 (Reference) | 1.31 (0.96-1.79) | 0.93 (0.67-1.31) | 1.24 (0.9-1.71) | 1.07 (0.72-1.58) | 1.04 (0.7-1.55) |
| **Any gastrointestinal diseases** | | | | | | |
| Cases/person-years | 5990/365134 | 358/17923 | 371/20911 | 333/17410 | 232/12086 | 246/12206 |
| Multivariable model* | 1 (Reference) | 1.00 (0.90-1.12) | **0.86 (0.78-0.96)** | 0.94 (0.84-1.06) | 0.93 (0.81-1.06) | 0.96 (0.84-1.10) |

Note: *Estimates are hazard ratios (95% confidence intervals), obtained from fully adjusted Cox regression models. GI, gastrointestinal; GERD, Gastroesophageal reflux disease; IBS, Irritable bowel syndrome; NAFLD, Non-alcoholic fatty liver disease.

**Supplementary Table 18. Associations of coffee consumption with incident GI diseases after additional adjustments to the intake of sugar-sweetened beverages, artificially sweetened beverages, natural juices, milk and water.**

| **Outcome** | **Nonconsumers** | **Coffee intake, drinks/d** | | | | |
| --- | --- | --- | --- | --- | --- | --- |
|  |  | **≤ 1** | **1-2** | **2-3** | **3-4** | **> 4** |
| **Unsweetened coffee** | | | | | | |
| **Number of participants** | 35528 | 17627 | 22684 | 18561 | 12583 | 11022 |
| **Barrett’s oesophagus** |  |  |  |  |  |  |
| Cases/person-years | 309/453611 | 133/226658 | 160/292328 | 131/239259 | 94/162308 | 87/141762 |
| Multivariable model* | 1 (Reference) | 0.93 (0.75-1.14) | 0.9 (0.73-1.1) | 0.91 (0.73-1.13) | 0.98 (0.77-1.26) | 1.03 (0.8-1.34) |
| **GERD** |  |  |  |  |  |  |
| Cases/person-years | 2273/442959 | 1030/221536 | 1200/286720 | 933/234971 | 708/158973 | 634/138710 |
| Multivariable model* | 1 (Reference) | 0.96 (0.89-1.03) | **0.89 (0.82-0.95)** | **0.85 (0.78-0.92)** | 0.96 (0.88-1.05) | 0.97 (0.88-1.07) |
| **Gastritis and duodenitis** |  |  |  |  |  |  |
| Cases/person-years | 2268/441280 | 998/221183 | 1147/286092 | 897/234546 | 624/159154 | 530/139203 |
| Multivariable model* | 1 (Reference) | **0.91 (0.85-0.98)** | **0.83 (0.77-0.89)** | **0.8 (0.74-0.87)** | **0.83 (0.75-0.91)** | **0.79 (0.71-0.88)** |
| **Celiac disease** |  |  |  |  |  |  |
| Cases/person-years | 122/454432 | 56/227012 | 64/292768 | 43/239683 | 33/162603 | 23/142079 |
| Multivariable model* | 1 (Reference) | 0.92 (0.67-1.28) | 0.85 (0.62-1.16) | 0.71 (0.49-1.03) | 0.82 (0.55-1.23) | 0.67 (0.42-1.08) |
| **Peptic ulcer** |  |  |  |  |  |  |
| Cases/person-years | 490/452238 | 177/226312 | 230/291830 | 171/238971 | 138/162032 | 116/141592 |
| Multivariable model* | 1 (Reference) | **0.74 (0.62-0.89)** | **0.74 (0.63-0.87)** | **0.66 (0.55-0.8)** | **0.78 (0.63-0.95)** | **0.71 (0.57-0.88)** |
| **Crohn’s disease** |  |  |  |  |  |  |
| Cases/person-years | 58/454866 | 20/227287 | 30/292987 | 20/239854 | 11/162736 | 24/142086 |
| Multivariable model* | 1 (Reference) | 0.76 (0.45-1.28) | 0.92 (0.58-1.47) | 0.76 (0.44-1.31) | 0.61 (0.31-1.21) | 1.49 (0.87-2.56) |
| **Ulcerative colitis** |  |  |  |  |  |  |
| Cases/person-years | 143/454397 | 59/227018 | 66/292762 | 57/239601 | 47/162533 | 34/142029 |
| Multivariable model* | 1 (Reference) | 0.95 (0.69-1.3) | 0.83 (0.61-1.13) | 0.87 (0.63-1.21) | 1.05 (0.73-1.49) | 0.84 (0.56-1.26) |
| **IBS** |  |  |  |  |  |  |
| Cases/person-years | 411/453049 | 201/226354 | 217/292024 | 173/239032 | 124/162186 | 101/141762 |
| Multivariable model* | 1 (Reference) | 1.01 (0.85-1.2) | 0.87 (0.73-1.04) | 0.87 (0.72-1.05) | 0.92 (0.74-1.13) | 0.83 (0.66-1.05) |
| **Diverticulum** |  |  |  |  |  |  |
| Cases/person-years | 3055/437556 | 1541/218549 | 1819/282841 | 1484/231657 | 997/157026 | 860/137434 |
| Multivariable model* | 1 (Reference) | 0.96 (0.91-1.03) | **0.87 (0.82-0.92)** | **0.86 (0.8-0.91)** | **0.84 (0.78-0.91)** | **0.81 (0.75-0.88)** |
| **Pancreatitis** |  |  |  |  |  |  |
| Cases/person-years | 143/454454 | 55/227091 | 84/292729 | 58/239651 | 41/162621 | 44/141968 |
| Multivariable model* | 1 (Reference) | 0.85 (0.62-1.17) | 1 (0.75-1.33) | 0.84 (0.61-1.17) | 0.86 (0.59-1.25) | 1.01 (0.7-1.47) |
| **NAFLD** |  |  |  |  |  |  |
| Cases/person-years | 296/454047 | 92/226864 | 110/292676 | 89/239588 | 67/162490 | 71/141919 |
| Multivariable model* | 1 (Reference) | **0.76 (0.6-0.97)** | **0.67 (0.53-0.84)** | **0.62 (0.48-0.8)** | **0.64 (0.48-0.84)** | **0.69 (0.51-0.91)** |
| **Cirrhosis** |  |  |  |  |  |  |
| Cases/person-years | 140/454818 | 57/227173 | 57/292933 | 45/239761 | 32/162723 | 29/142148 |
| Multivariable model* | 1 (Reference) | 0.87 (0.63-1.2) | **0.62 (0.45-0.86)** | **0.56 (0.39-0.8)** | **0.55 (0.36-0.83)** | **0.51 (0.33-0.8)** |
| **Biliary diseases** |  |  |  |  |  |  |
| Cases/person-years | 1069/449042 | 436/224804 | 530/290025 | 459/237133 | 344/160745 | 303/140412 |
| Multivariable model* | 1 (Reference) | **0.87 (0.78-0.98)** | **0.82 (0.74-0.92)** | **0.86 (0.76-0.97)** | 0.93 (0.81-1.06) | 0.9 (0.79-1.04) |
| **Appendicitis** |  |  |  |  |  |  |
| Cases/person-years | 215/453724 | 99/226625 | 115/292412 | 91/239311 | 65/162381 | 54/141864 |
| Multivariable model* | 1 (Reference) | 0.93 (0.73-1.18) | 0.84 (0.66-1.06) | 0.81 (0.62-1.05) | 0.84 (0.62-1.13) | 0.78 (0.57-1.09) |
| **Gastrointestinal cancer** |  |  |  |  |  |  |
| Cases/person-years | 606/453054 | 330/226173 | 439/291567 | 366/238581 | 248/161893 | 224/141475 |
| Multivariable model* | 1 (Reference) | 0.99 (0.86-1.13) | 0.99 (0.86-1.13) | 0.99 (0.86-1.13) | 0.99 (0.86-1.13) | 0.99 (0.86-1.13) |
| **Any gastrointestinal diseases** | | | | | | |
| Cases/person-years | 7365/410565 | 3465/206408 | 4155/268499 | 3335/220416 | 2341/148955 | 2054/130379 |
| Multivariable model* | 1 (Reference) | **0.94 (0.9-0.98)** | **0.87 (0.84-0.9)** | **0.85 (0.81-0.89)** | **0.88 (0.84-0.93)** | **0.87 (0.82-0.92)** |
| **Sugar-sweetened coffee** | | | | | | |
| **Number of participants** | 35528 | 6512 | 5963 | 3935 | 2256 | 2013 |
| **Barrett’s oesophagus** |  |  |  |  |  |  |
| Cases/person-years | 309/453611 | 86/82862 | 45/76215 | 35/50242 | 15/28650 | 30/25383 |
| Multivariable model* | 1 (Reference) | 1.39 (1.08-1.78) | 0.79 (0.57-1.1) | 0.96 (0.66-1.39) | 0.7 (0.41-1.2) | 1.62 (1.07-2.45) |
| **GERD** |  |  |  |  |  |  |
| Cases/person-years | 2273/442959 | 452/80730 | 373/74358 | 278/48934 | 147/27866 | 127/24917 |
| Multivariable model* | 1 (Reference) | 1.06 (0.96-1.18) | 0.96 (0.86-1.08) | 1.09 (0.96-1.25) | 1 (0.84-1.19) | 0.94 (0.77-1.14) |
| **Gastritis and duodenitis** |  |  |  |  |  |  |
| Cases/person-years | 2268/441280 | 382/80918 | 353/74223 | 221/49102 | 117/27974 | 120/24798 |
| Multivariable model* | 1 (Reference) | **0.86 (0.77-0.97)** | 0.89 (0.79-1) | **0.85 (0.73-0.98)** | **0.77 (0.64-0.94)** | 0.87 (0.71-1.05) |
| **Celiac disease** |  |  |  |  |  |  |
| Cases/person-years | 122/454432 | 20/83161 | 17/76379 | 5/50409 | 0/28732 | 7/25504 |
| Multivariable model* | 1 (Reference) | 0.9 (0.56-1.46) | 0.91 (0.53-1.54) | 0.43 (0.17-1.07) | 0 (0-Inf) | 1.38 (0.61-3.11) |
| **Peptic ulcer** |  |  |  |  |  |  |
| Cases/person-years | 490/452238 | 81/82846 | 70/76017 | 55/50138 | 28/28576 | 28/25444 |
| Multivariable model* | 1 (Reference) | 0.8 (0.63-1.02) | **0.75 (0.58-0.98)** | 0.88 (0.66-1.19) | 0.77 (0.52-1.15) | 0.81 (0.54-1.22) |
| **Crohn’s disease** |  |  |  |  |  |  |
| Cases/person-years | 58/454866 | 14/83198 | 15/76381 | 5/50411 | 2/28716 | 3/25541 |
| Multivariable model* | 1 (Reference) | 1.16 (0.64-2.13) | 1.3 (0.71-2.4) | 0.63 (0.24-1.65) | 0.41 (0.1-1.75) | 0.62 (0.18-2.14) |
| **Ulcerative colitis** |  |  |  |  |  |  |
| Cases/person-years | 143/454397 | 20/83175 | 20/76371 | 18/50311 | 4/28708 | 7/25504 |
| Multivariable model* | 1 (Reference) | 0.74 (0.46-1.2) | 0.78 (0.48-1.27) | 1.05 (0.62-1.77) | 0.38 (0.14-1.06) | 0.68 (0.3-1.53) |
| **IBS** |  |  |  |  |  |  |
| Cases/person-years | 411/453049 | 71/82936 | 62/76137 | 41/50248 | 20/28632 | 17/25464 |
| Multivariable model* | 1 (Reference) | 1.03 (0.79-1.33) | 1.02 (0.77-1.35) | 1.07 (0.76-1.5) | 0.94 (0.59-1.5) | 0.88 (0.53-1.47) |
| **Diverticulum** |  |  |  |  |  |  |
| Cases/person-years | 3055/437556 | 624/79804 | 540/73419 | 342/48441 | 205/27653 | 189/24488 |
| Multivariable model* | 1 (Reference) | 1 (0.92-1.09) | 0.92 (0.83-1.01) | **0.88 (0.78-0.99)** | 0.89 (0.76-1.03) | 0.94 (0.8-1.1) |
| **Pancreatitis** |  |  |  |  |  |  |
| Cases/person-years | 143/454454 | 36/83143 | 25/76344 | 13/50378 | 8/28693 | 7/25535 |
| Multivariable model* | 1 (Reference) | 1.34 (0.92-1.95) | 0.98 (0.62-1.53) | 0.75 (0.42-1.37) | 0.76 (0.36-1.6) | 0.69 (0.31-1.54) |
| **NAFLD** |  |  |  |  |  |  |
| Cases/person-years | 296/454047 | 53/83053 | 48/76247 | 21/50353 | 22/28650 | 21/25458 |
| Multivariable model* | 1 (Reference) | 1.03 (0.76-1.39) | 0.97 (0.7-1.34) | 0.63 (0.4-1) | 1.03 (0.65-1.63) | 0.99 (0.61-1.6) |
| **Cirrhosis** |  |  |  |  |  |  |
| Cases/person-years | 140/454818 | 24/83232 | 21/76420 | 14/50408 | 10/28702 | 9/25517 |
| Multivariable model* | 1 (Reference) | 0.74 (0.48-1.16) | **0.61 (0.38-0.99)** | 0.56 (0.31-1.01) | 0.6 (0.3-1.19) | 0.53 (0.25-1.1) |
| **Biliary diseases** |  |  |  |  |  |  |
| Cases/person-years | 1069/449042 | 207/82116 | 160/75587 | 108/49801 | 70/28355 | 51/25281 |
| Multivariable model* | 1 (Reference) | 1.09 (0.93-1.27) | 0.92 (0.78-1.1) | 0.95 (0.77-1.17) | 1.04 (0.8-1.34) | 0.84 (0.62-1.13) |
| **Appendicitis** |  |  |  |  |  |  |
| Cases/person-years | 215/453724 | 42/83040 | 33/76296 | 19/50303 | 6/28695 | 20/25412 |
| Multivariable model* | 1 (Reference) | 1.09 (0.78-1.54) | 0.93 (0.63-1.36) | 0.81 (0.49-1.32) | 0.45 (0.2-1.03) | 1.63 (0.98-2.71) |
| **Gastrointestinal cancer** |  |  |  |  |  |  |
| Cases/person-years | 606/453054 | 129/82856 | 126/75939 | 93/50109 | 45/28578 | 60/25364 |
| Multivariable model* | 1 (Reference) | 0.91 (0.75-1.11) | 0.9 (0.74-1.11) | 0.98 (0.78-1.24) | 0.78 (0.57-1.08) | 1.19 (0.89-1.6) |
| **Any gastrointestinal diseases** | | | | | | |
| Cases/person-years | 7365/410565 | 1417/74740 | 1240/68960 | 814/45582 | 457/26031 | 413/23076 |
| Multivariable model* | 1 (Reference) | 1 (0.94-1.06) | 0.94 (0.89-1) | 0.94 (0.87-1.01) | **0.9 (0.81-0.99)** | 0.91 (0.82-1.01) |
| **Artificially sweetened coffee** | | | | | | |
| **Number of participants** | 35528 | 1944 | 2205 | 1820 | 1296 | 1314 |
| **Barrett’s oesophagus** |  |  |  |  |  |  |
| Cases/person-years | 309/453611 | 28/24666 | 16/28200 | 11/23217 | 13/16508 | 15/16707 |
| Multivariable model* | 1 (Reference) | 1.4 (0.94-2.08) | 0.72 (0.43-1.2) | 0.65 (0.35-1.21) | 1.07 (0.6-1.9) | 1.24 (0.71-2.15) |
| **GERD** |  |  |  |  |  |  |
| Cases/person-years | 2273/442959 | 147/23964 | 165/27360 | 135/22546 | 83/16128 | 107/16234 |
| Multivariable model* | 1 (Reference) | 0.97 (0.82-1.15) | 0.93 (0.79-1.1) | 0.99 (0.83-1.19) | 0.84 (0.67-1.05) | 1.01 (0.82-1.24) |
| **Gastritis and duodenitis** |  |  |  |  |  |  |
| Cases/person-years | 2268/441280 | 144/23877 | 159/27374 | 107/22648 | 94/16053 | 97/16193 |
| Multivariable model* | 1 (Reference) | 0.98 (0.83-1.17) | 0.94 (0.79-1.1) | **0.81 (0.66-0.99)** | 0.98 (0.79-1.21) | 0.97 (0.78-1.2) |
| **Celiac disease** |  |  |  |  |  |  |
| Cases/person-years | 122/454432 | 6/24772 | 3/28274 | 4/23253 | 3/16577 | 3/16780 |
| Multivariable model* | 1 (Reference) | 0.99 (0.43-2.27) | 0.49 (0.15-1.56) | 0.84 (0.3-2.32) | 0.99 (0.31-3.2) | 1.03 (0.31-3.37) |
| **Peptic ulcer** |  |  |  |  |  |  |
| Cases/person-years | 490/452238 | 33/24656 | 42/28072 | 26/23151 | 16/16503 | 22/16670 |
| Multivariable model* | 1 (Reference) | 1.02 (0.71-1.45) | 1.1 (0.79-1.53) | 0.88 (0.58-1.32) | 0.72 (0.43-1.21) | 0.95 (0.6-1.49) |
| **Crohn’s disease** |  |  |  |  |  |  |
| Cases/person-years | 58/454866 | 3/24791 | 4/28258 | 3/23250 | 1/16585 | 1/16793 |
| Multivariable model* | 1 (Reference) | 0.89 (0.27-2.88) | 1 (0.35-2.85) | 0.93 (0.28-3.11) | 0.4 (0.05-3.01) | 0.35 (0.05-2.67) |
| **Ulcerative colitis** |  |  |  |  |  |  |
| Cases/person-years | 143/454397 | 8/24754 | 14/28194 | 9/23210 | 5/16567 | 6/16749 |
| Multivariable model* | 1 (Reference) | 0.96 (0.46-1.97) | 1.5 (0.84-2.66) | 1.17 (0.58-2.37) | 0.88 (0.35-2.23) | 1.01 (0.43-2.41) |
| **IBS** |  |  |  |  |  |  |
| Cases/person-years | 411/453049 | 26/24687 | 24/28172 | 24/23138 | 17/16510 | 18/16705 |
| Multivariable model* | 1 (Reference) | 0.98 (0.65-1.46) | 0.76 (0.5-1.15) | 0.99 (0.65-1.53) | 0.97 (0.58-1.6) | 0.95 (0.57-1.57) |
| **Diverticulum** |  |  |  |  |  |  |
| Cases/person-years | 3055/437556 | 201/23725 | 217/27011 | 176/22232 | 138/15747 | 134/16102 |
| Multivariable model* | 1 (Reference) | 0.91 (0.79-1.05) | **0.84 (0.73-0.97)** | **0.84 (0.72-0.98)** | 0.89 (0.74-1.06) | **0.82 (0.68-0.98)** |
| **Pancreatitis** |  |  |  |  |  |  |
| Cases/person-years | 143/454454 | 9/24768 | 11/28231 | 9/23222 | 6/16545 | 9/16766 |
| Multivariable model* | 1 (Reference) | 0.86 (0.43-1.7) | 0.89 (0.47-1.67) | 0.91 (0.45-1.84) | 0.77 (0.33-1.81) | 1.07 (0.51-2.22) |
| **NAFLD** |  |  |  |  |  |  |
| Cases/person-years | 296/454047 | 21/24742 | 36/28098 | 21/23195 | 21/16495 | 10/16768 |
| Multivariable model* | 1 (Reference) | 0.85 (0.54-1.33) | 1.16 (0.81-1.67) | 0.91 (0.57-1.44) | 1.1 (0.69-1.77) | 0.44 (0.23-0.85) |
| **Cirrhosis** |  |  |  |  |  |  |
| Cases/person-years | 140/454818 | 17/24740 | 15/28236 | 6/23259 | 7/16564 | 3/16788 |
| Multivariable model* | 1 (Reference) | 1.29 (0.77-2.17) | 0.86 (0.49-1.51) | **0.4 (0.17-0.93)** | 0.57 (0.25-1.26) | **0.2 (0.06-0.66)** |
| **Biliary diseases** |  |  |  |  |  |  |
| Cases/person-years | 1069/449042 | 77/24434 | 69/27858 | 78/22839 | 58/16248 | 54/16505 |
| Multivariable model* | 1 (Reference) | 1.01 (0.8-1.28) | **0.77 (0.6-0.99)** | 1.15 (0.9-1.46) | 1.15 (0.87-1.51) | 0.99 (0.74-1.33) |
| **Appendicitis** |  |  |  |  |  |  |
| Cases/person-years | 215/453724 | 12/24700 | 12/28182 | 8/23203 | 5/16543 | 5/16772 |
| Multivariable model* | 1 (Reference) | 0.99 (0.55-1.79) | 0.84 (0.46-1.53) | 0.69 (0.34-1.43) | 0.6 (0.24-1.48) | 0.57 (0.23-1.42) |
| **Gastrointestinal cancer** |  |  |  |  |  |  |
| Cases/person-years | 606/453054 | 66/24562 | 52/28102 | 51/23078 | 32/16483 | 42/16675 |
| Multivariable model* | 1 (Reference) | 1.42 (1.1-1.85) | 0.93 (0.69-1.25) | 1.07 (0.79-1.44) | 0.88 (0.6-1.28) | 1.1 (0.78-1.55) |
| **Any gastrointestinal diseases** | | | | | | |
| Cases/person-years | 7365/410565 | 478/21878 | 518/25129 | 424/20754 | 312/14676 | 330/14920 |
| Multivariable model* | 1 (Reference) | 0.97 (0.89-1.07) | **0.9 (0.82-0.99)** | 0.92 (0.83-1.02) | 0.93 (0.83-1.05) | 0.93 (0.83-1.05) |

Note: *Estimates are hazard ratios (95% confidence intervals), obtained from fully adjusted Cox regression models. GI, gastrointestinal; GERD, Gastroesophageal reflux disease; IBS, Irritable bowel syndrome; NAFLD, Non-alcoholic fatty liver disease.

**Supplementary Table 19. Associations of coffee consumption with incident GI diseases after removing sugar added to coffee from total sugar and total energy.**

| **Outcome** | **Nonconsumers** | **Coffee intake, drinks/d** | | | | |
| --- | --- | --- | --- | --- | --- | --- |
|  |  | **≤ 1** | **1-2** | **2-3** | **3-4** | **> 4** |
| **Unsweetened coffee** | | | | | | |
| **Number of participants** | 35528 | 17627 | 22684 | 18561 | 12583 | 11022 |
| **Barrett’s oesophagus** |  |  |  |  |  |  |
| Cases/person-years | 309/453611 | 133/226658 | 160/292328 | 131/239259 | 94/162308 | 87/141762 |
| Multivariable model* | 1 (Reference) | 0.92 (0.75-1.14) | 0.89 (0.73-1.09) | 0.9 (0.73-1.12) | 0.98 (0.76-1.25) | 1.03 (0.79-1.33) |
| **GERD** |  |  |  |  |  |  |
| Cases/person-years | 2273/442959 | 1030/221536 | 1200/286720 | 933/234971 | 708/158973 | 634/138710 |
| Multivariable model* | 1 (Reference) | 0.95 (0.88-1.03) | **0.88 (0.82-0.94)** | **0.84 (0.77-0.91)** | 0.95 (0.87-1.04) | 0.96 (0.87-1.06) |
| **Gastritis and duodenitis** | | | | | | |
| Cases/person-years | 2268/441280 | 998/221183 | 1147/286092 | 897/234546 | 624/159154 | 530/139203 |
| Multivariable model* | 1 (Reference) | **0.91 (0.84-0.98)** | **0.82 (0.76-0.89)** | **0.8 (0.73-0.86)** | **0.82 (0.75-0.9)** | **0.78 (0.71-0.87)** |
| **Celiac disease** |  |  |  |  |  |  |
| Cases/person-years | 122/454432 | 56/227012 | 64/292768 | 43/239683 | 33/162603 | 23/142079 |
| Multivariable model* | 1 (Reference) | 0.92 (0.66-1.27) | 0.84 (0.62-1.16) | 0.71 (0.49-1.03) | 0.83 (0.55-1.25) | 0.69 (0.43-1.1) |
| **Peptic ulcer** |  |  |  |  |  |  |
| Cases/person-years | 490/452238 | 177/226312 | 230/291830 | 171/238971 | 138/162032 | 116/141592 |
| Multivariable model* | 1 (Reference) | **0.74 (0.62-0.89)** | **0.74 (0.63-0.87)** | **0.66 (0.55-0.8)** | **0.77 (0.63-0.95)** | **0.7 (0.56-0.88)** |
| **Crohn’s disease** |  |  |  |  |  |  |
| Cases/person-years | 58/454866 | 20/227287 | 30/292987 | 20/239854 | 11/162736 | 24/142086 |
| Multivariable model* | 1 (Reference) | 0.79 (0.47-1.33) | 0.96 (0.6-1.52) | 0.79 (0.46-1.35) | 0.64 (0.32-1.25) | 1.55 (0.91-2.64) |
| **Ulcerative colitis** |  |  |  |  |  |  |
| Cases/person-years | 143/454397 | 59/227018 | 66/292762 | 57/239601 | 47/162533 | 34/142029 |
| Multivariable model* | 1 (Reference) | 0.95 (0.69-1.3) | 0.84 (0.62-1.14) | 0.88 (0.63-1.22) | 1.06 (0.74-1.51) | 0.85 (0.56-1.27) |
| **IBS** |  |  |  |  |  |  |
| Cases/person-years | 411/453049 | 201/226354 | 217/292024 | 173/239032 | 124/162186 | 101/141762 |
| Multivariable model* | 1 (Reference) | 1 (0.84-1.19) | 0.86 (0.72-1.02) | 0.86 (0.71-1.03) | 0.9 (0.73-1.12) | 0.82 (0.65-1.04) |
| **Diverticulum** |  |  |  |  |  |  |
| Cases/person-years | 3055/437556 | 1541/218549 | 1819/282841 | 1484/231657 | 997/157026 | 860/137434 |
| Multivariable model* | 1 (Reference) | 0.96 (0.9-1.02) | **0.86 (0.81-0.92)** | **0.85 (0.8-0.91)** | **0.84 (0.78-0.9)** | **0.81 (0.75-0.88)** |
| **Pancreatitis** |  |  |  |  |  |  |
| Cases/person-years | 143/454454 | 55/227091 | 84/292729 | 58/239651 | 41/162621 | 44/141968 |
| Multivariable model* | 1 (Reference) | 0.84 (0.61-1.15) | 1 (0.75-1.33) | 0.84 (0.61-1.17) | 0.86 (0.6-1.25) | 1.02 (0.7-1.48) |
| **NAFLD** |  |  |  |  |  |  |
| Cases/person-years | 296/454047 | 92/226864 | 110/292676 | 89/239588 | 67/162490 | 71/141919 |
| Multivariable model* | 1 (Reference) | **0.75 (0.59-0.96)** | **0.66 (0.52-0.83)** | **0.61 (0.48-0.79)** | **0.62 (0.47-0.83)** | **0.66 (0.5-0.88)** |
| **Cirrhosis** |  |  |  |  |  |  |
| Cases/person-years | 140/454818 | 57/227173 | 57/292933 | 45/239761 | 32/162723 | 29/142148 |
| Multivariable model* | 1 (Reference) | 0.86 (0.63-1.19) | **0.61 (0.44-0.85)** | **0.55 (0.38-0.79)** | **0.54 (0.35-0.81)** | **0.49 (0.32-0.77)** |
| **Biliary diseases** |  |  |  |  |  |  |
| Cases/person-years | 1069/449042 | 436/224804 | 530/290025 | 459/237133 | 344/160745 | 303/140412 |
| Multivariable model* | 1 (Reference) | **0.87 (0.78-0.97)** | **0.82 (0.74-0.92)** | **0.86 (0.77-0.97)** | 0.94 (0.82-1.07) | 0.91 (0.8-1.05) |
| **Appendicitis** |  |  |  |  |  |  |
| Cases/person-years | 215/453724 | 99/226625 | 115/292412 | 91/239311 | 65/162381 | 54/141864 |
| Multivariable model* | 1 (Reference) | 0.93 (0.73-1.19) | 0.84 (0.66-1.06) | 0.8 (0.62-1.04) | 0.83 (0.62-1.12) | 0.77 (0.56-1.06) |
| **Gastrointestinal cancer** |  |  |  |  |  |  |
| Cases/person-years | 606/453054 | 330/226173 | 439/291567 | 366/238581 | 248/161893 | 224/141475 |
| Multivariable model* | 1 (Reference) | 0.98 (0.85-1.12) | 0.97 (0.86-1.11) | 0.97 (0.85-1.12) | 0.97 (0.82-1.13) | 0.99 (0.83-1.17) |
| **Any gastrointestinal diseases** | | | | | | |
| Cases/person-years | 7365/410565 | 3465/206408 | 4155/268499 | 3335/220416 | 2341/148955 | 2054/130379 |
| Multivariable model* | 1 (Reference) | **0.94 (0.9-0.98)** | **0.87 (0.83-0.9)** | **0.84 (0.81-0.88)** | **0.88 (0.83-0.92)** | **0.86 (0.82-0.91)** |
| **Sugar-sweetened coffee** | | | | | | |
| **Number of participants** | 35528 | 6512 | 5963 | 3935 | 2256 | 2013 |
| **Barrett’s oesophagus** |  |  |  |  |  |  |
| Cases/person-years | 309/453611 | 86/82862 | 45/76215 | 35/50242 | 15/28650 | 30/25383 |
| Multivariable model* | 1 (Reference) | 1.37 (1.07-1.75) | 0.78 (0.56-1.08) | 0.95 (0.66-1.37) | 0.69 (0.4-1.18) | 1.6 (1.06-2.41) |
| **GERD** |  |  |  |  |  |  |
| Cases/person-years | 2273/442959 | 452/80730 | 373/74358 | 278/48934 | 147/27866 | 127/24917 |
| Multivariable model* | 1 (Reference) | 1.06 (0.96-1.18) | 0.96 (0.86-1.07) | 1.09 (0.96-1.24) | 1 (0.84-1.19) | 0.94 (0.78-1.13) |
| **Gastritis and duodenitis** | | | | | | |
| Cases/person-years | 2268/441280 | 382/80918 | 353/74223 | 221/49102 | 117/27974 | 120/24798 |
| Multivariable model* | 1 (Reference) | **0.86 (0.77-0.96)** | **0.88 (0.79-0.99)** | **0.84 (0.73-0.97)** | **0.77 (0.63-0.93)** | 0.85 (0.7-1.04) |
| **Celiac disease** |  |  |  |  |  |  |
| Cases/person-years | 122/454432 | 20/83161 | 17/76379 | 5/50409 | 0/28732 | 7/25504 |
| Multivariable model* | 1 (Reference) | 0.91 (0.56-1.48) | 0.92 (0.54-1.56) | 0.44 (0.18-1.09) | 0 (0-Inf) | 1.43 (0.64-3.2) |
| **Peptic ulcer** |  |  |  |  |  |  |
| Cases/person-years | 490/452238 | 81/82846 | 70/76017 | 55/50138 | 28/28576 | 28/25444 |
| Multivariable model* | 1 (Reference) | 0.8 (0.63-1.02) | **0.75 (0.58-0.97)** | 0.87 (0.65-1.17) | 0.76 (0.51-1.13) | 0.79 (0.53-1.18) |
| **Crohn’s disease** |  |  |  |  |  |  |
| Cases/person-years | 58/454866 | 14/83198 | 15/76381 | 5/50411 | 2/28716 | 3/25541 |
| Multivariable model* | 1 (Reference) | 1.19 (0.65-2.16) | 1.34 (0.73-2.44) | 0.65 (0.25-1.69) | 0.43 (0.1-1.82) | 0.65 (0.19-2.23) |
| **Ulcerative colitis** |  |  |  |  |  |  |
| Cases/person-years | 143/454397 | 20/83175 | 20/76371 | 18/50311 | 4/28708 | 7/25504 |
| Multivariable model* | 1 (Reference) | 0.73 (0.45-1.18) | 0.77 (0.47-1.25) | 1.02 (0.61-1.72) | 0.37 (0.14-1.03) | 0.65 (0.29-1.45) |
| **IBS** |  |  |  |  |  |  |
| Cases/person-years | 411/453049 | 71/82936 | 62/76137 | 41/50248 | 20/28632 | 17/25464 |
| Multivariable model* | 1 (Reference) | 1.03 (0.8-1.34) | 1.03 (0.78-1.36) | 1.08 (0.77-1.51) | 0.95 (0.6-1.51) | 0.89 (0.54-1.48) |
| **Diverticulum** |  |  |  |  |  |  |
| Cases/person-years | 3055/437556 | 624/79804 | 540/73419 | 342/48441 | 205/27653 | 189/24488 |
| Multivariable model* | 1 (Reference) | 1 (0.92-1.09) | 0.92 (0.83-1.01) | **0.88 (0.78-0.99)** | 0.89 (0.76-1.03) | 0.94 (0.8-1.1) |
| **Pancreatitis** |  |  |  |  |  |  |
| Cases/person-years | 143/454454 | 36/83143 | 25/76344 | 13/50378 | 8/28693 | 7/25535 |
| Multivariable model* | 1 (Reference) | 1.33 (0.91-1.94) | 0.97 (0.62-1.51) | 0.74 (0.41-1.34) | 0.74 (0.35-1.56) | 0.66 (0.3-1.48) |
| **NAFLD** |  |  |  |  |  |  |
| Cases/person-years | 296/454047 | 53/83053 | 48/76247 | 21/50353 | 22/28650 | 21/25458 |
| Multivariable model* | 1 (Reference) | 1 (0.74-1.35) | 0.94 (0.68-1.29) | 0.6 (0.38-0.95) | 0.98 (0.62-1.55) | 0.92 (0.57-1.48) |
| **Cirrhosis** |  |  |  |  |  |  |
| Cases/person-years | 140/454818 | 24/83232 | 21/76420 | 14/50408 | 10/28702 | 9/25517 |
| Multivariable model* | 1 (Reference) | 0.73 (0.47-1.14) | **0.6 (0.37-0.98)** | **0.56 (0.31-0.99)** | 0.6 (0.3-1.18) | 0.53 (0.26-1.1) |
| **Biliary diseases** |  |  |  |  |  |  |
| Cases/person-years | 1069/449042 | 207/82116 | 160/75587 | 108/49801 | 70/28355 | 51/25281 |
| Multivariable model* | 1 (Reference) | 1.1 (0.94-1.28) | 0.93 (0.78-1.11) | 0.96 (0.78-1.18) | 1.05 (0.82-1.35) | 0.85 (0.63-1.15) |
| **Appendicitis** |  |  |  |  |  |  |
| Cases/person-years | 215/453724 | 42/83040 | 33/76296 | 19/50303 | 6/28695 | 20/25412 |
| Multivariable model* | 1 (Reference) | 1.07 (0.76-1.5) | 0.9 (0.61-1.32) | 0.78 (0.48-1.27) | **0.43 (0.19-0.98)** | 1.51 (0.91-2.5) |
| **Gastrointestinal cancer** |  |  |  |  |  |  |
| Cases/person-years | 606/453054 | 129/82856 | 126/75939 | 93/50109 | 45/28578 | 60/25364 |
| Multivariable model* | 1 (Reference) | 0.91 (0.75-1.1) | 0.9 (0.73-1.1) | 0.97 (0.77-1.23) | 0.77 (0.56-1.06) | 1.17 (0.88-1.57) |
| **Any gastrointestinal diseases** | | | | | | |
| Cases/person-years | 7365/410565 | 1417/74740 | 1240/68960 | 814/45582 | 457/26031 | 413/23076 |
| Multivariable model* | 1 (Reference) | 1 (0.94-1.06) | 0.94 (0.88-1) | 0.93 (0.87-1.01) | **0.89 (0.81-0.98)** | 0.9 (0.81-1) |
| **Artificially sweetened coffee** | | | | | | |
| **Number of participants** | 35528 | 1944 | 2205 | 1820 | 1296 | 1314 |
| **Barrett’s oesophagus** |  |  |  |  |  |  |
| Cases/person-years | 309/453611 | 28/24666 | 16/28200 | 11/23217 | 13/16508 | 15/16707 |
| Multivariable model* | 1 (Reference) | 1.37 (0.92-2.03) | 0.7 (0.42-1.16) | 0.63 (0.34-1.17) | 1.03 (0.58-1.83) | 1.19 (0.68-2.06) |
| **GERD** |  |  |  |  |  |  |
| Cases/person-years | 2273/442959 | 147/23964 | 165/27360 | 135/22546 | 83/16128 | 107/16234 |
| Multivariable model* | 1 (Reference) | 0.97 (0.82-1.15) | 0.93 (0.79-1.1) | 0.99 (0.82-1.18) | 0.84 (0.67-1.05) | 1.01 (0.82-1.24) |
| **Gastritis and duodenitis** | | | | | | |
| Cases/person-years | 2268/441280 | 144/23877 | 159/27374 | 107/22648 | 94/16053 | 97/16193 |
| Multivariable model* | 1 (Reference) | 0.98 (0.83-1.16) | 0.93 (0.79-1.1) | **0.8 (0.66-0.98)** | 0.97 (0.78-1.2) | 0.96 (0.77-1.19) |
| **Celiac disease** |  |  |  |  |  |  |
| Cases/person-years | 122/454432 | 6/24772 | 3/28274 | 4/23253 | 3/16577 | 3/16780 |
| Multivariable model* | 1 (Reference) | 1.05 (0.46-2.41) | 0.52 (0.16-1.66) | 0.91 (0.33-2.52) | 1.08 (0.34-3.5) | 1.17 (0.36-3.81) |
| **Peptic ulcer** |  |  |  |  |  |  |
| Cases/person-years | 490/452238 | 33/24656 | 42/28072 | 26/23151 | 16/16503 | 22/16670 |
| Multivariable model* | 1 (Reference) | 1 (0.7-1.43) | 1.08 (0.78-1.5) | 0.86 (0.57-1.3) | 0.71 (0.43-1.19) | 0.93 (0.59-1.46) |
| **Crohn’s disease** |  |  |  |  |  |  |
| Cases/person-years | 58/454866 | 3/24791 | 4/28258 | 3/23250 | 1/16585 | 1/16793 |
| Multivariable model* | 1 (Reference) | 0.9 (0.28-2.93) | 1.01 (0.35-2.89) | 0.95 (0.29-3.17) | 0.41 (0.06-3.11) | 0.37 (0.05-2.78) |
| **Ulcerative colitis** |  |  |  |  |  |  |
| Cases/person-years | 143/454397 | 8/24754 | 14/28194 | 9/23210 | 5/16567 | 6/16749 |
| Multivariable model* | 1 (Reference) | 0.98 (0.48-2.03) | 1.53 (0.86-2.71) | 1.2 (0.6-2.43) | 0.89 (0.35-2.24) | 1.02 (0.43-2.41) |
| **IBS** |  |  |  |  |  |  |
| Cases/person-years | 411/453049 | 26/24687 | 24/28172 | 24/23138 | 17/16510 | 18/16705 |
| Multivariable model* | 1 (Reference) | 0.97 (0.65-1.45) | 0.75 (0.49-1.15) | 0.99 (0.65-1.52) | 0.96 (0.58-1.59) | 0.94 (0.57-1.55) |
| **Diverticulum** |  |  |  |  |  |  |
| Cases/person-years | 3055/437556 | 201/23725 | 217/27011 | 176/22232 | 138/15747 | 134/16102 |
| Multivariable model* | 1 (Reference) | 0.91 (0.79-1.06) | 0.84 (0.73-0.97) | **0.84 (0.72-0.99)** | 0.89 (0.74-1.06) | **0.81 (0.68-0.98)** |
| **Pancreatitis** |  |  |  |  |  |  |
| Cases/person-years | 143/454454 | 9/24768 | 11/28231 | 9/23222 | 6/16545 | 9/16766 |
| Multivariable model* | 1 (Reference) | 0.87 (0.44-1.72) | 0.89 (0.47-1.67) | 0.91 (0.45-1.83) | 0.75 (0.32-1.77) | 1.02 (0.49-2.13) |
| **NAFLD** |  |  |  |  |  |  |
| Cases/person-years | 296/454047 | 21/24742 | 36/28098 | 21/23195 | 21/16495 | 10/16768 |
| Multivariable model* | 1 (Reference) | 0.85 (0.54-1.34) | 1.16 (0.81-1.67) | 0.9 (0.57-1.43) | 1.09 (0.68-1.75) | 0.43 (0.22-0.84) |
| **Cirrhosis** |  |  |  |  |  |  |
| Cases/person-years | 140/454818 | 17/24740 | 15/28236 | 6/23259 | 7/16564 | 3/16788 |
| Multivariable model* | 1 (Reference) | 1.32 (0.78-2.21) | 0.88 (0.5-1.53) | 0.41 (0.17-0.94) | 0.57 (0.26-1.27) | **0.2 (0.06-0.66)** |
| **Biliary diseases** |  |  |  |  |  |  |
| Cases/person-years | 1069/449042 | 77/24434 | 69/27858 | 78/22839 | 58/16248 | 54/16505 |
| Multivariable model* | 1 (Reference) | 1.04 (0.82-1.31) | 0.79 (0.62-1.01) | 1.17 (0.92-1.49) | 1.17 (0.89-1.55) | 1.02 (0.76-1.37) |
| **Appendicitis** |  |  |  |  |  |  |
| Cases/person-years | 215/453724 | 12/24700 | 12/28182 | 8/23203 | 5/16543 | 5/16772 |
| Multivariable model* | 1 (Reference) | 0.98 (0.54-1.76) | 0.83 (0.46-1.51) | 0.68 (0.33-1.4) | 0.58 (0.23-1.44) | 0.54 (0.22-1.36) |
| **Gastrointestinal cancer** |  |  |  |  |  |  |
| Cases/person-years | 606/453054 | 66/24562 | 52/28102 | 51/23078 | 32/16483 | 42/16675 |
| Multivariable model* | 1 (Reference) | 1.41 (1.09-1.83) | 0.92 (0.69-1.24) | 1.06 (0.78-1.43) | 0.87 (0.6-1.26) | 1.09 (0.78-1.53) |
| **Any gastrointestinal diseases** | | | | | | |
| Cases/person-years | 7365/410565 | 478/21878 | 518/25129 | 424/20754 | 312/14676 | 330/14920 |
| Multivariable model* | 1 (Reference) | 0.98 (0.89-1.07) | **0.9 (0.82-0.99)** | 0.92 (0.83-1.02) | 0.93 (0.83-1.05) | 0.93 (0.83-1.05) |

Note: *Estimates are hazard ratios (95% confidence intervals), obtained from fully adjusted Cox regression models. GI, gastrointestinal; GERD, Gastroesophageal reflux disease; IBS, Irritable bowel syndrome; NAFLD, Non-alcoholic fatty liver disease.


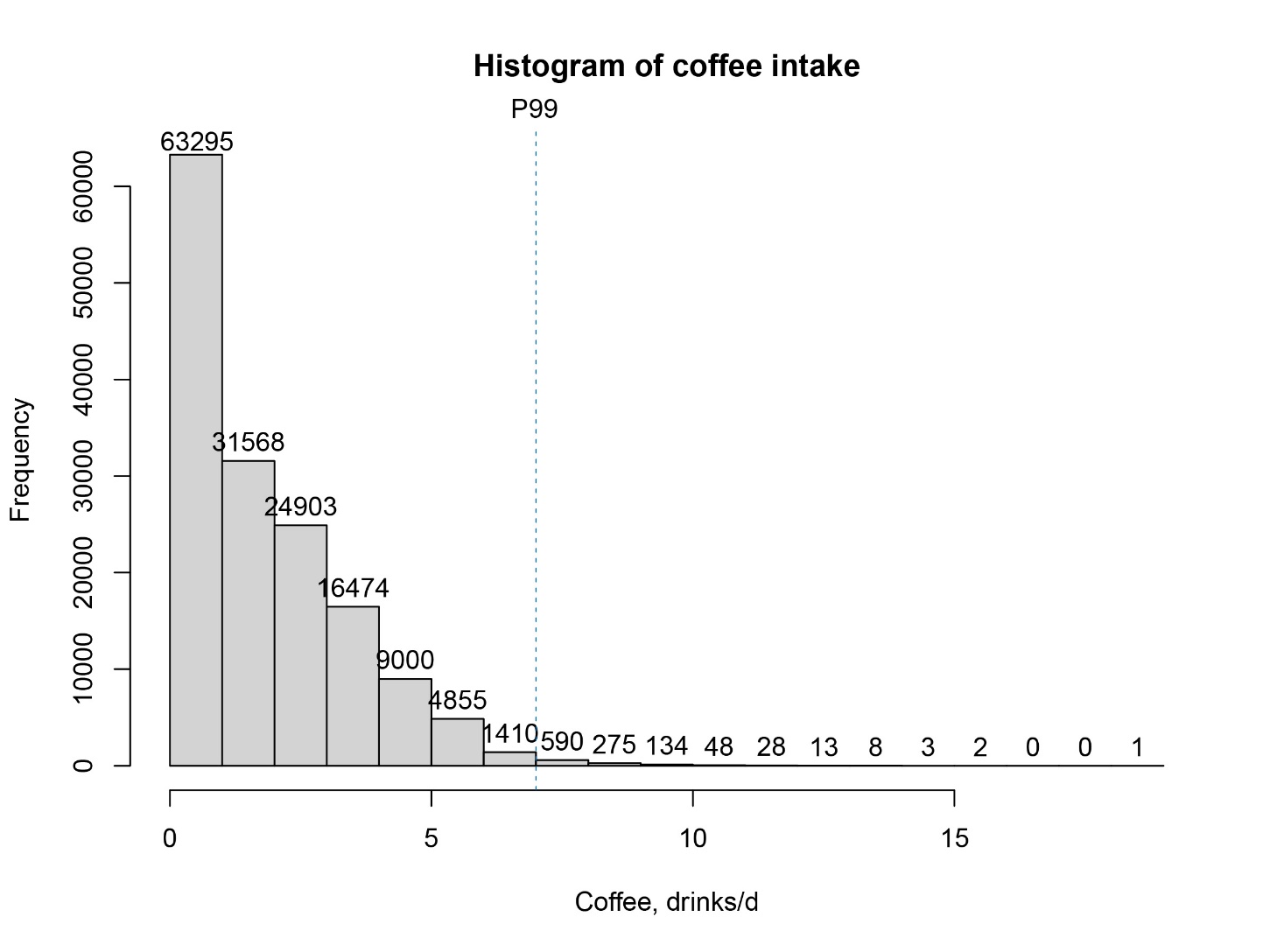


# Supplementary Figure 1. Histogram of total coffee intake. The dashed blue line is the 99th percentile of coffee intake.


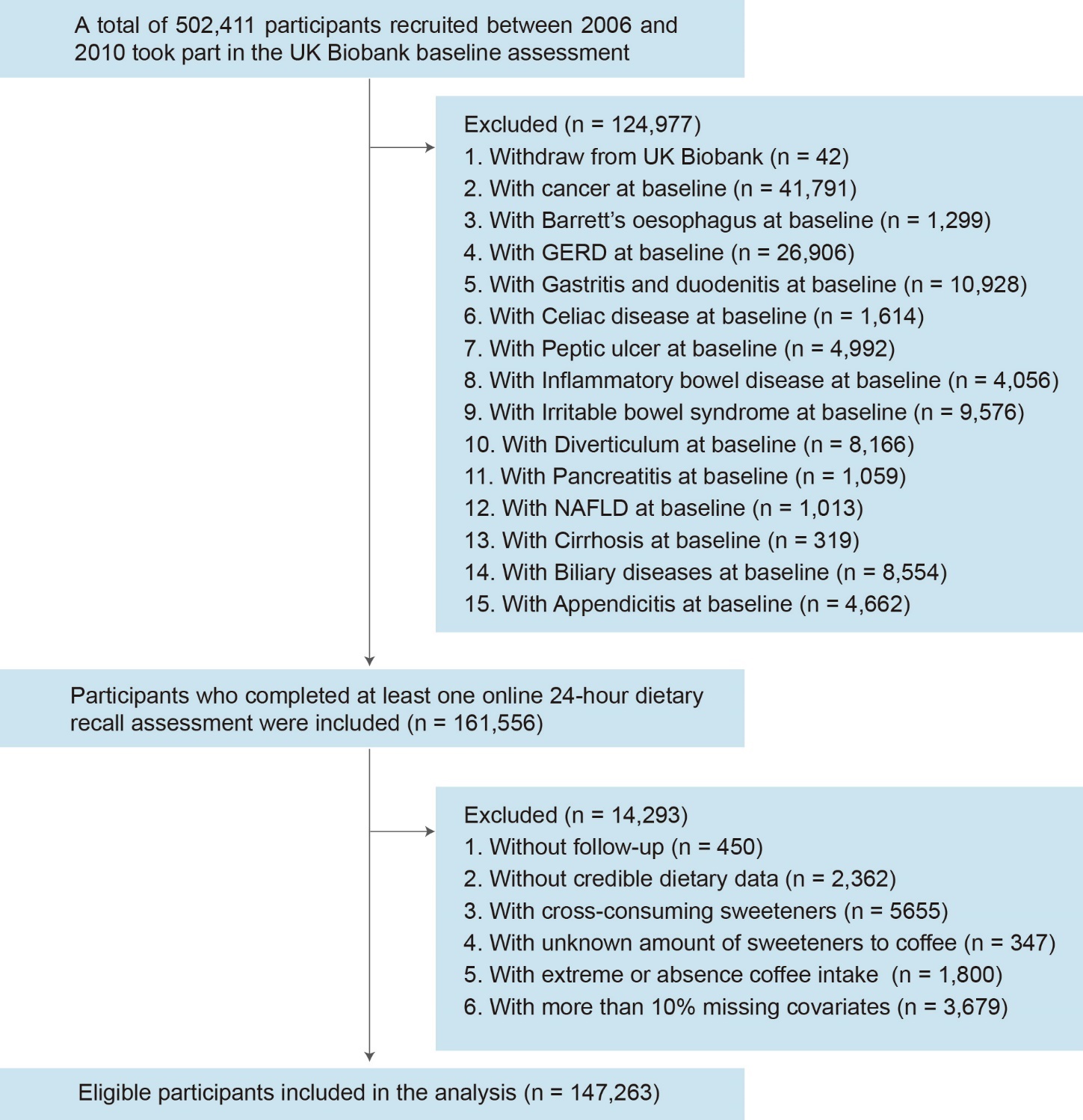


# Supplementary Figure 2. Flowchart of study design and participant enrollment.


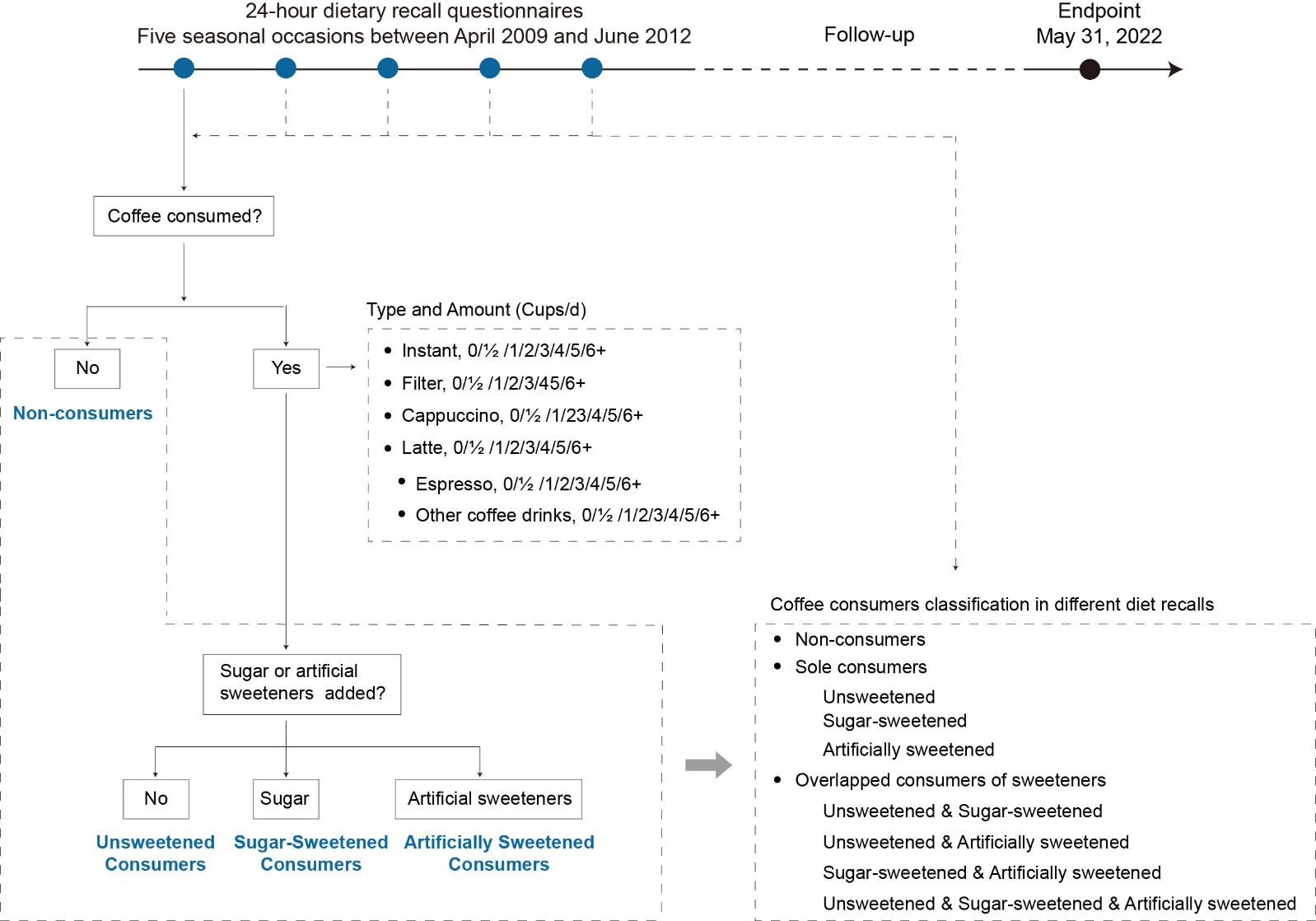


# Supplementary Figure 3. Flow chart of coffee consumer classification.


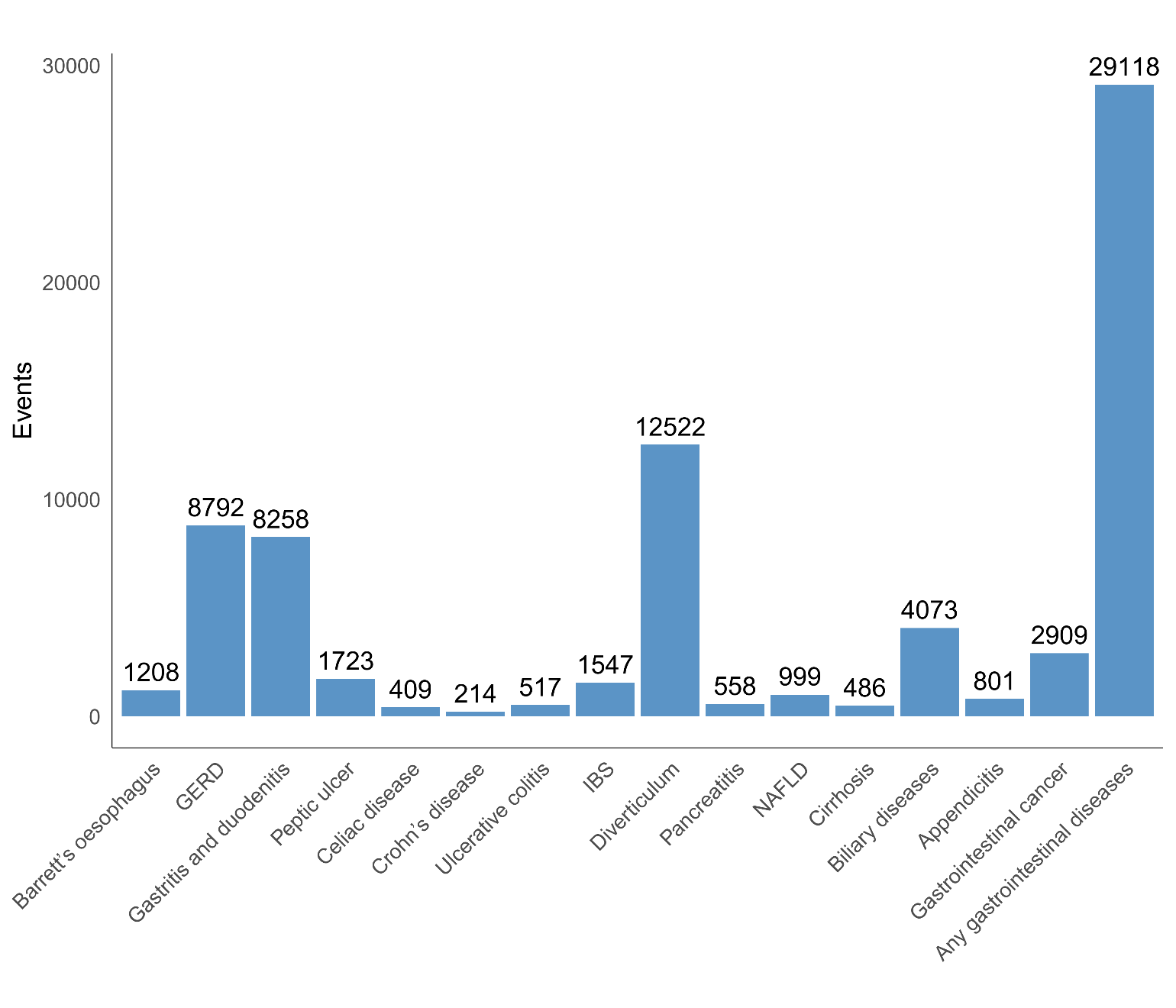


# **Supplementary Figure 4. An overview of case distribution among gastrointestinal disease subtypes.**


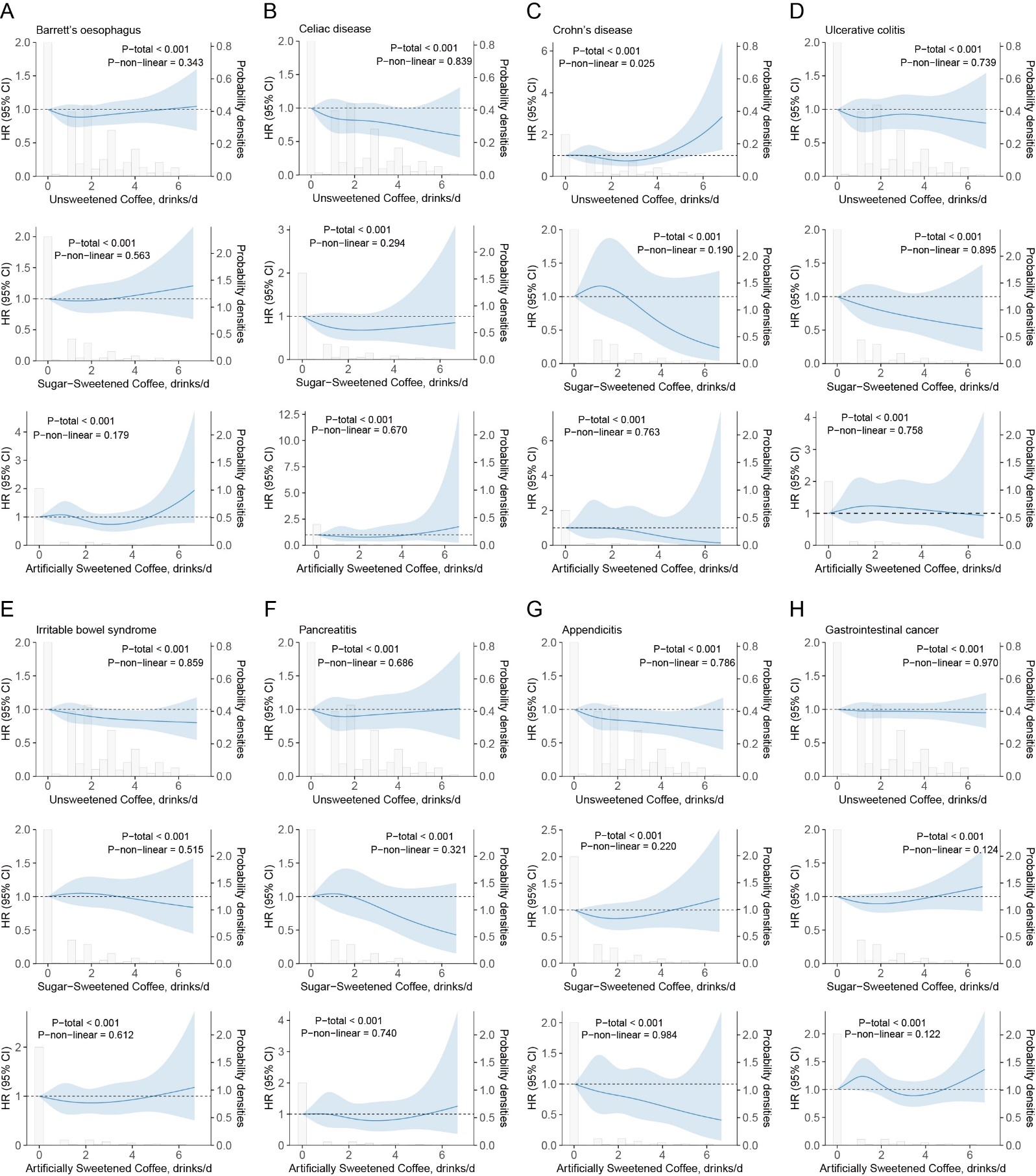


# Supplementary Figure 5. Dose–response associations of coffee consumption with incident barrett’s oesophagus (A), celiac disease (B), crohn’s disease (C), ulcerative colitis (D), irritable bowel syndrome (E), pancreatitis (F), appendicitis (G), and gastrointestinal cancer (H).

Note: Multivariable Cox regression model with restricted cubic splines adjusted for age (continuous), sex (male or female), body mass index (<25, ≥ 25 & < 30, and ≥ 30 km/m^2^), ethnicity (white or other), Townsend deprivation index (continuous), current employment status (work, retired or other), education level (degree or no degree), smoking status (current, former, or never), pack-years of smoking (continuous), physical activity level (low, moderate, or high), healthy sleep pattern (yes or no), hot drink temperature (very hot, hot, warm, or other), vitamin use (yes or no), mineral and other dietary supplements use (yes or no), NSAIDs use (yes or no), PPI use (yes or no), INFLA-score (continuous), family history of CVD disease (yes or no), family history of cancer (yes or no), number of long-term conditions (none, one, two, three and more), and intake of total energy, total sugar, tea, and AHEI score. HR, hazard ratio; CI, confidence interval; BMI, NSAIDs, Nonsteroidal anti-inflammatory drugs; AHEI, Alternative Healthy Eating Index, GI, gastrointestinal; GERD, Gastroesophageal reflux disease; IBS, Irritable bowel syndrome; NAFLD, Non-alcoholic fatty liver disease.


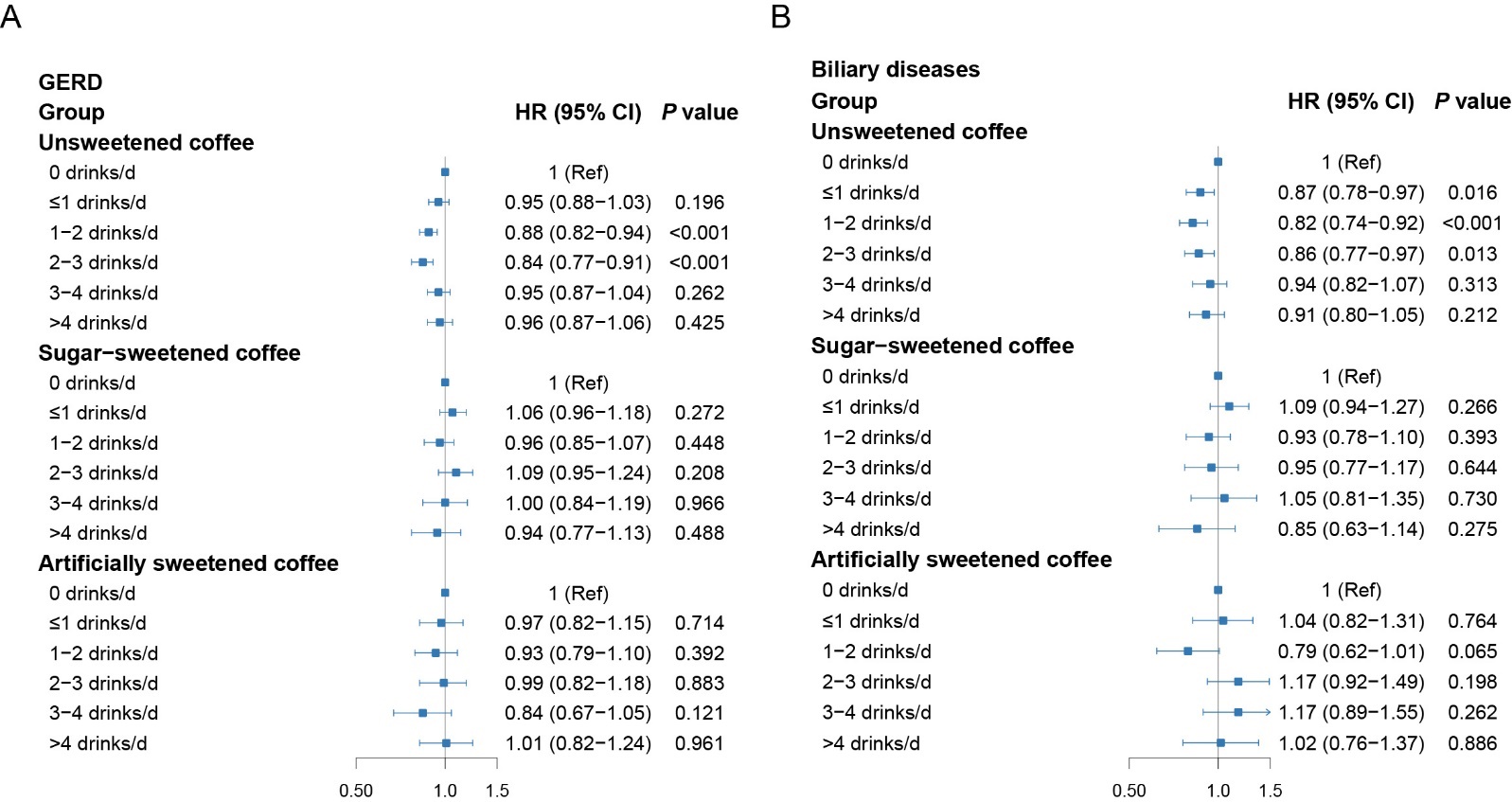


# Supplementary Figure 6. Association of coffee intake and risk of GERD and biliary diseases.

Note: Multivariable Cox regression model with restricted cubic splines adjusted for age (continuous), sex (male or female), body mass index (<25, ≥ 25 & < 30, and ≥ 30 km/m^2^), ethnicity (white or other), Townsend deprivation index (continuous), current employment status (work, retired or other), education level (degree or no degree), smoking status (current, former, or never), pack-years of smoking (continuous), physical activity level (low, moderate, or high), healthy sleep pattern (yes or no), hot drink temperature (very hot, hot, warm, or other), vitamin use (yes or no), mineral and other dietary supplements use (yes or no), NSAIDs use (yes or no), PPI use (yes or no), INFLA-score (continuous), family history of CVD disease (yes or no), family history of cancer (yes or no), number of long-term conditions (none, one, two, three and more), and intake of total energy, total sugar, tea, and AHEI score. HR, hazard ratio; CI, confidence interval; BMI, NSAIDs, Nonsteroidal anti-inflammatory drugs; AHEI, Alternative Healthy Eating Index, GERD, Gastroesophageal reflux disease.


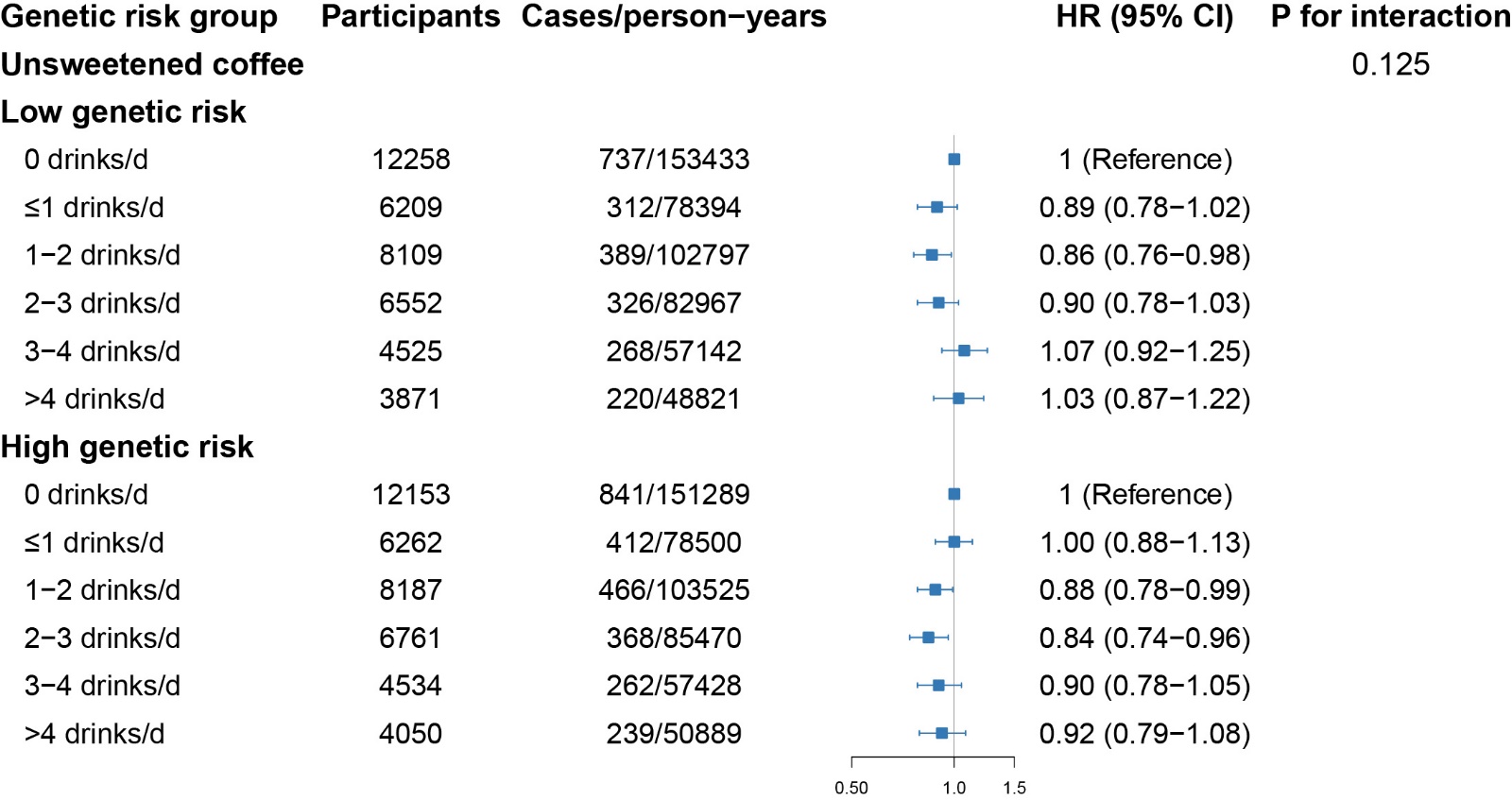


# Supplementary Figure 7. Associations of unsweetened coffee consumption with incident GERD according to the polygenic risk score.

Note: Multivariable model, estimates are hazard ratios (95% CIs) from multivariable Cox proportional hazard models adjusted for age (continuous), gender, body mass index ((<25, ≥ 25 & < 30, and ≥ 30 kg/m^2^), ethnicity (white or other), Townsend deprivation index (continuous), Current employment status (work, retired, or other), education level (degree or no degree), smoking status (current, former, or never), pack-years of smoking (continuous), physical activity level (low, moderate, or high), healthy sleep pattern (yes or no), hot drink temperature (very hot, hot, warm, or other), vitamin use (yes or no), mineral and other dietary supplements use (yes or no), NSAIDs use (yes or no), PPI use (yes or no), INFLA-score (continuous), family history of CVD disease (yes or no), family history of cancer (yes or no), number of long-term conditions (none, one, two, three and more), and intake of total energy, total sugar, total tea, AHEI score, genotyping batch, and the first 10 genetic principal components. HR, hazard ratio; CI, confidence interval; NSAIDs, Nonsteroidal anti-inflammatory drugs; AHEI, Alternative Healthy Eating Index, GERD, Gastroesophageal reflux disease.


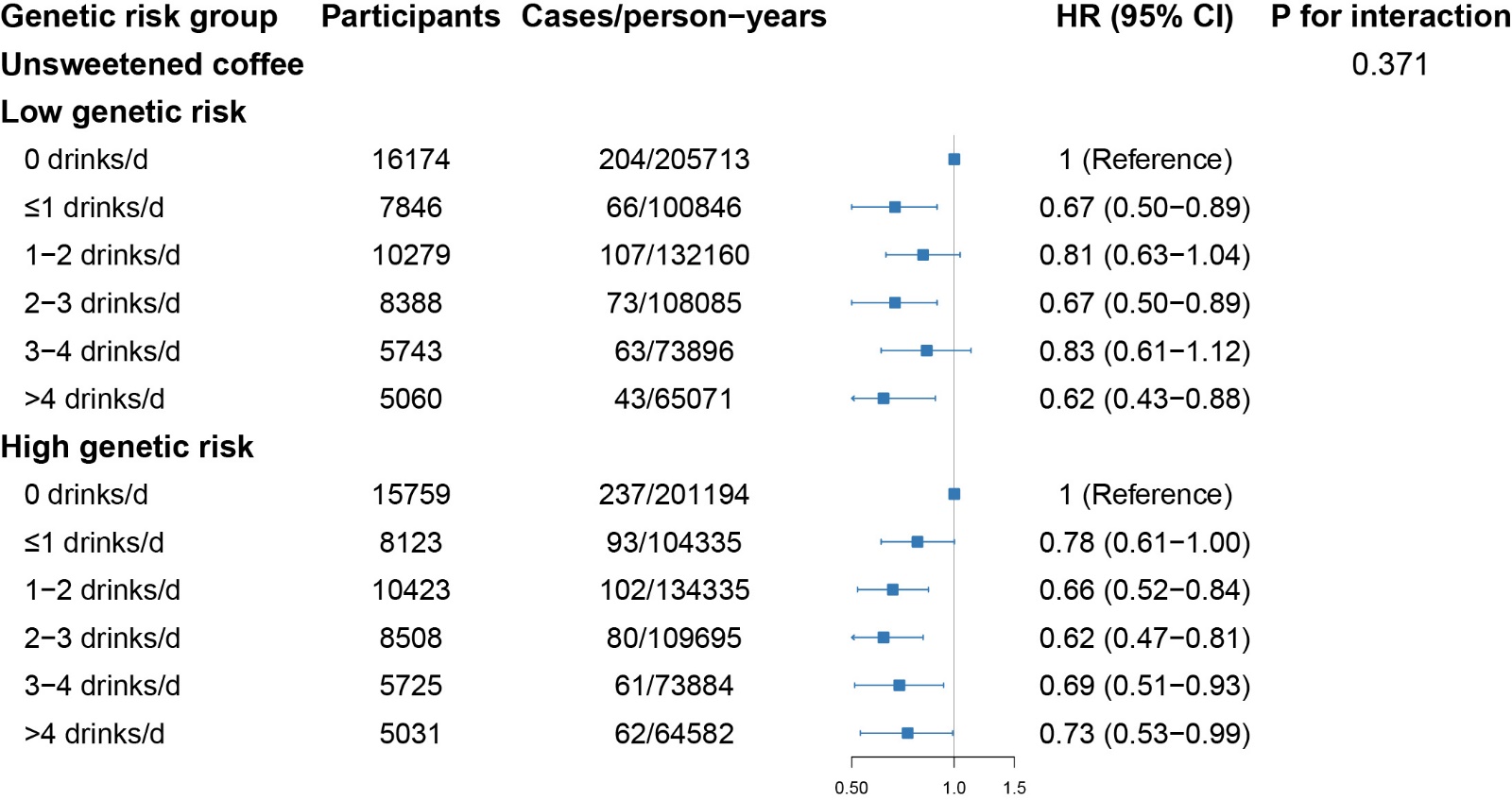


# Supplementary Figure 8. Associations of unsweetened coffee consumption with incident peptic ulcer according to the polygenic risk score.

Note: Multivariable model, estimates are hazard ratios (95% CIs) from multivariable Cox proportional hazard models adjusted for age (continuous), gender, body mass index ((<25, ≥ 25 & < 30, and ≥ 30 kg/m^2^), ethnicity (white or other), Townsend deprivation index (continuous), Current employment status (work, retired, or other), education level (degree or no degree), smoking status (current, former, or never), pack-years of smoking (continuous), physical activity level (low, moderate, or high), healthy sleep pattern (yes or no), hot drink temperature (very hot, hot, warm, or other), vitamin use (yes or no), mineral and other dietary supplements use (yes or no), NSAIDs use (yes or no), PPI use (yes or no), INFLA-score (continuous), family history of CVD disease (yes or no), family history of cancer (yes or no), number of long-term conditions (none, one, two, three and more), and intake of total energy, total sugar, total tea, AHEI score, genotyping batch, and the first 10 genetic principal components. HR, hazard ratio; CI, confidence interval; NSAIDs, Nonsteroidal anti-inflammatory drugs; AHEI, Alternative Healthy Eating Index.


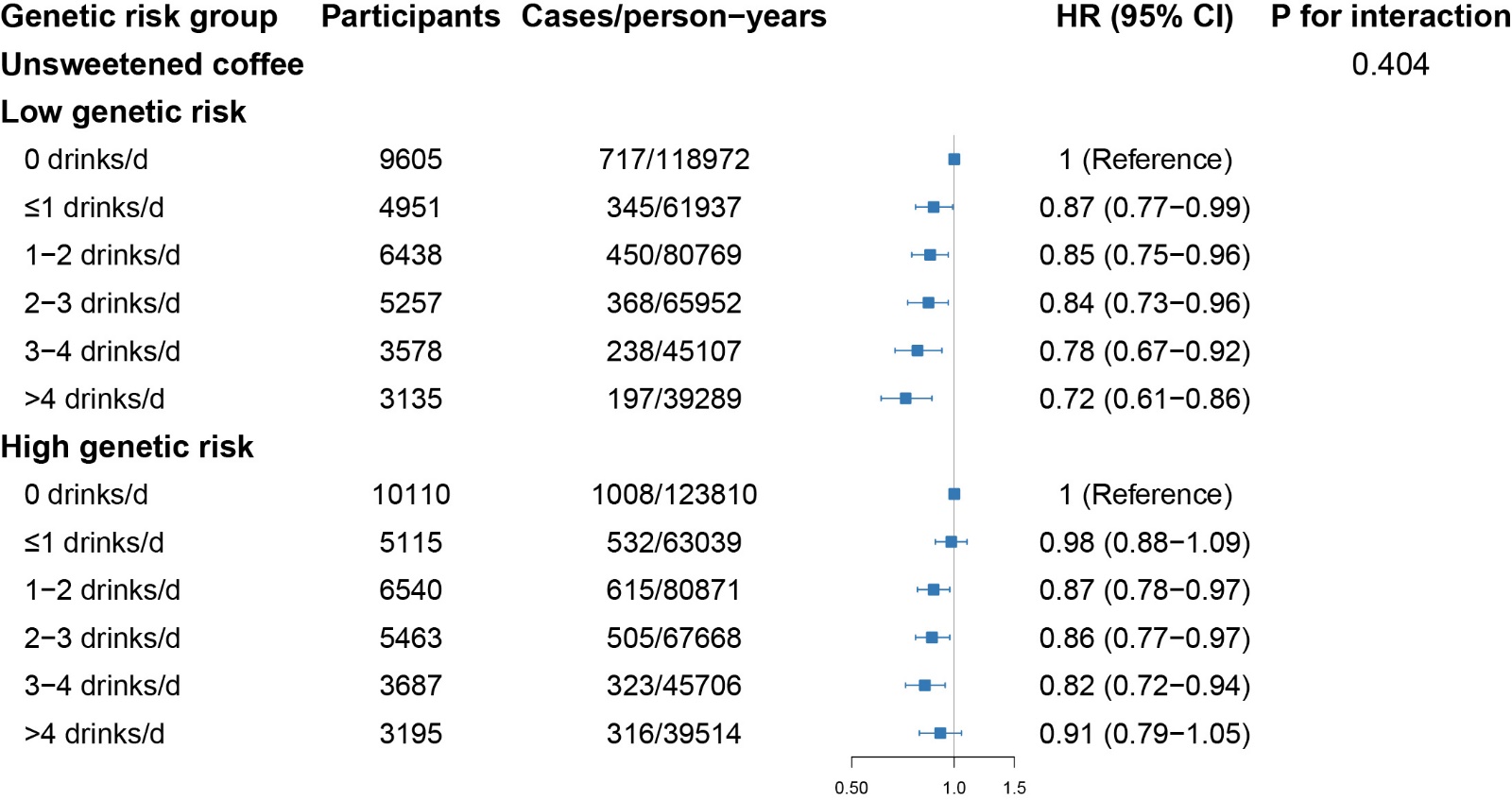


# Supplementary Figure 9. Associations of unsweetened coffee consumption with incident diverticulum according to the polygenic risk score.

Note: Multivariable model, estimates are hazard ratios (95% CIs) from multivariable Cox proportional hazard models adjusted for age (continuous), gender, body mass index ((<25, ≥ 25 & < 30, and ≥ 30 kg/m^2^), ethnicity (white or other), Townsend deprivation index (continuous), Current employment status (work, retired, or other), education level (degree or no degree), smoking status (current, former, or never), pack-years of smoking (continuous), physical activity level (low, moderate, or high), healthy sleep pattern (yes or no), hot drink temperature (very hot, hot, warm, or other), vitamin use (yes or no), mineral and other dietary supplements use (yes or no), NSAIDs use (yes or no), PPI use (yes or no), INFLA-score (continuous), family history of CVD disease (yes or no), family history of cancer (yes or no), number of long-term conditions (none, one, two, three and more), and intake of total energy, total sugar, total tea, AHEI score, genotyping batch, and the first 10 genetic principal components. HR, hazard ratio; CI, confidence interval; NSAIDs, Nonsteroidal anti-inflammatory drugs; AHEI, Alternative Healthy Eating Index.


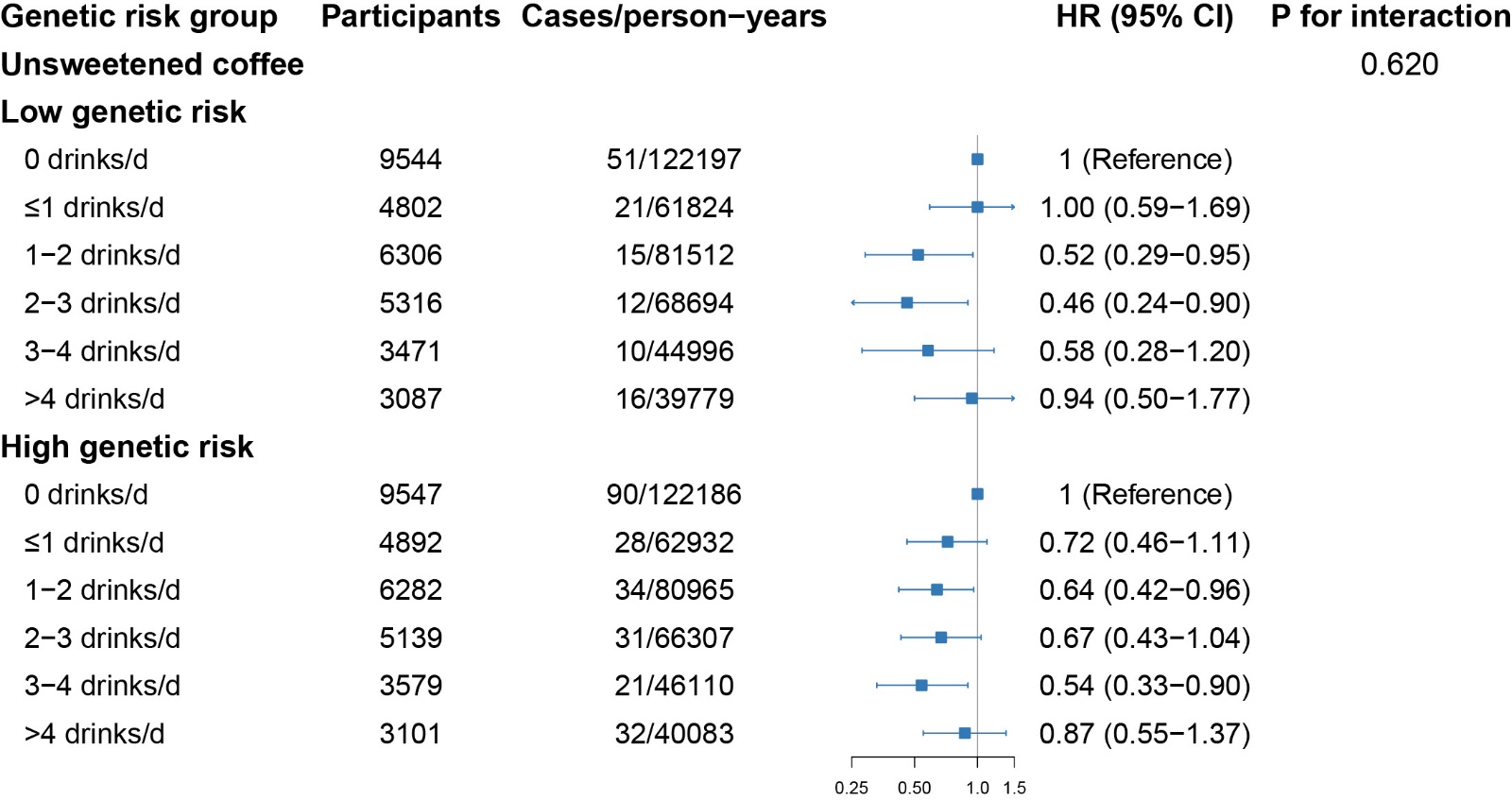


# Supplementary Figure 10. Associations of unsweetened coffee consumption with incident NAFLD according to the polygenic risk score.

Note: Multivariable model, estimates are hazard ratios (95% CIs) from multivariable Cox proportional hazard models adjusted for age (continuous), gender, body mass index ((<25, ≥ 25 & < 30, and ≥ 30 kg/m^2^), ethnicity (white or other), Townsend deprivation index (continuous), Current employment status (work, retired, or other), education level (degree or no degree), smoking status (current, former, or never), pack-years of smoking (continuous), physical activity level (low, moderate, or high), healthy sleep pattern (yes or no), hot drink temperature (very hot, hot, warm, or other), vitamin use (yes or no), mineral and other dietary supplements use (yes or no), NSAIDs use (yes or no), PPI use (yes or no), INFLA-score (continuous), family history of CVD disease (yes or no), family history of cancer (yes or no), number of long-term conditions (none, one, two, three and more), and intake of total energy, total sugar, total tea, AHEI score, genotyping batch, and the first 10 genetic principal components. HR, hazard ratio; CI, confidence interval; NSAIDs, Nonsteroidal anti-inflammatory drugs; AHEI, Alternative Healthy Eating Index; NAFLD, Non-alcoholic fatty liver disease.


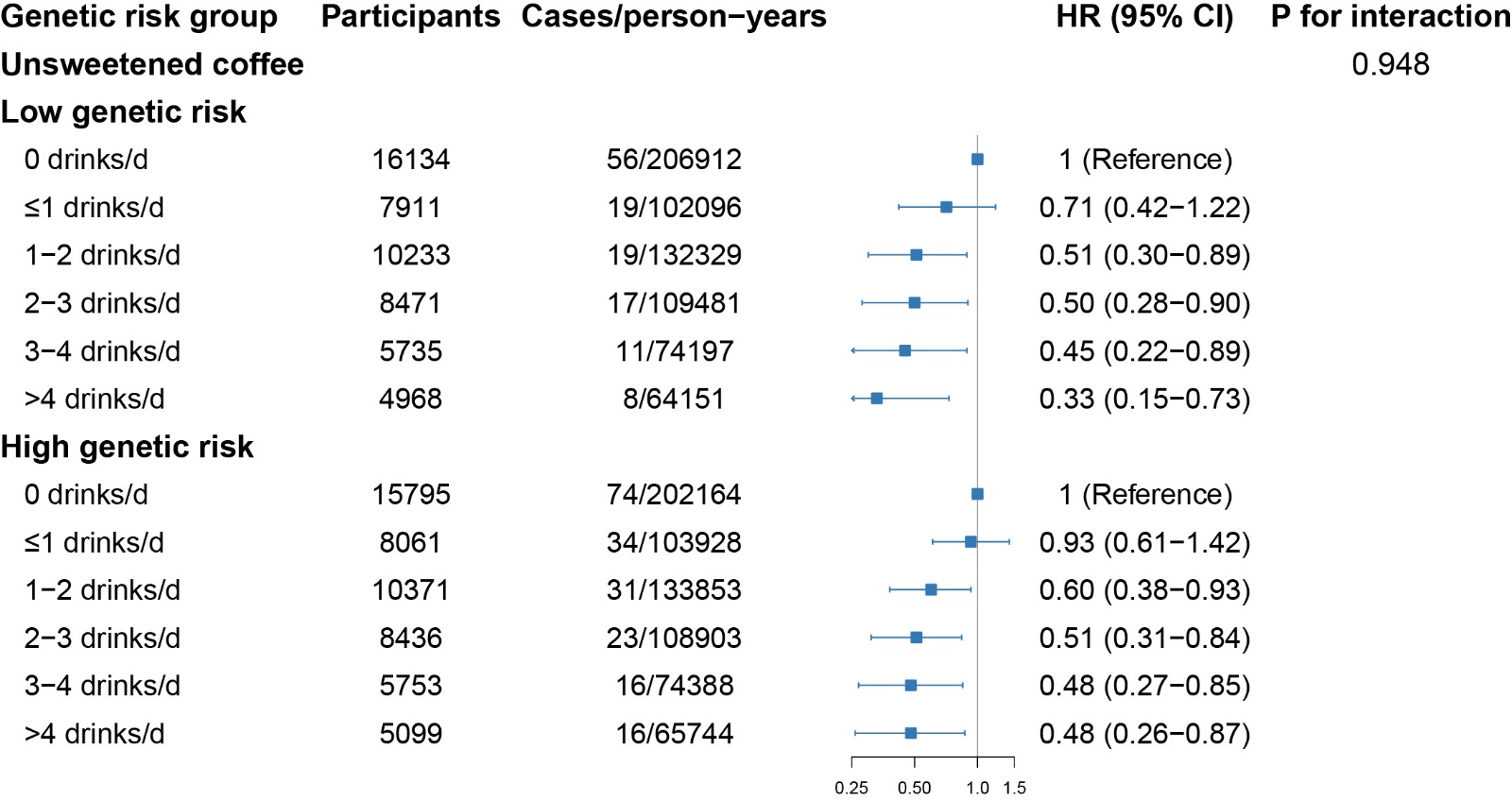


# Supplementary Figure 11. Associations of unsweetened coffee consumption with incident cirrhosis according to the polygenic risk score.

Note: Multivariable model, estimates are hazard ratios (95% CIs) from multivariable Cox proportional hazard models adjusted for age (continuous), gender, body mass index ((<25, ≥ 25 & < 30, and ≥ 30 kg/m^2^), ethnicity (white or other), Townsend deprivation index (continuous), Current employment status (work, retired, or other), education level (degree or no degree), smoking status (current, former, or never), pack-years of smoking (continuous), physical activity level (low, moderate, or high), healthy sleep pattern (yes or no), hot drink temperature (very hot, hot, warm, or other), vitamin use (yes or no), mineral and other dietary supplements use (yes or no), NSAIDs use (yes or no), PPI use (yes or no), INFLA-score (continuous), family history of CVD disease (yes or no), family history of cancer (yes or no), number of long-term conditions (none, one, two, three and more), and intake of total energy, total sugar, total tea, AHEI score, genotyping batch, and the first 10 genetic principal components. HR, hazard ratio; CI, confidence interval; NSAIDs, Nonsteroidal anti-inflammatory drugs; AHEI, Alternative Healthy Eating Index.


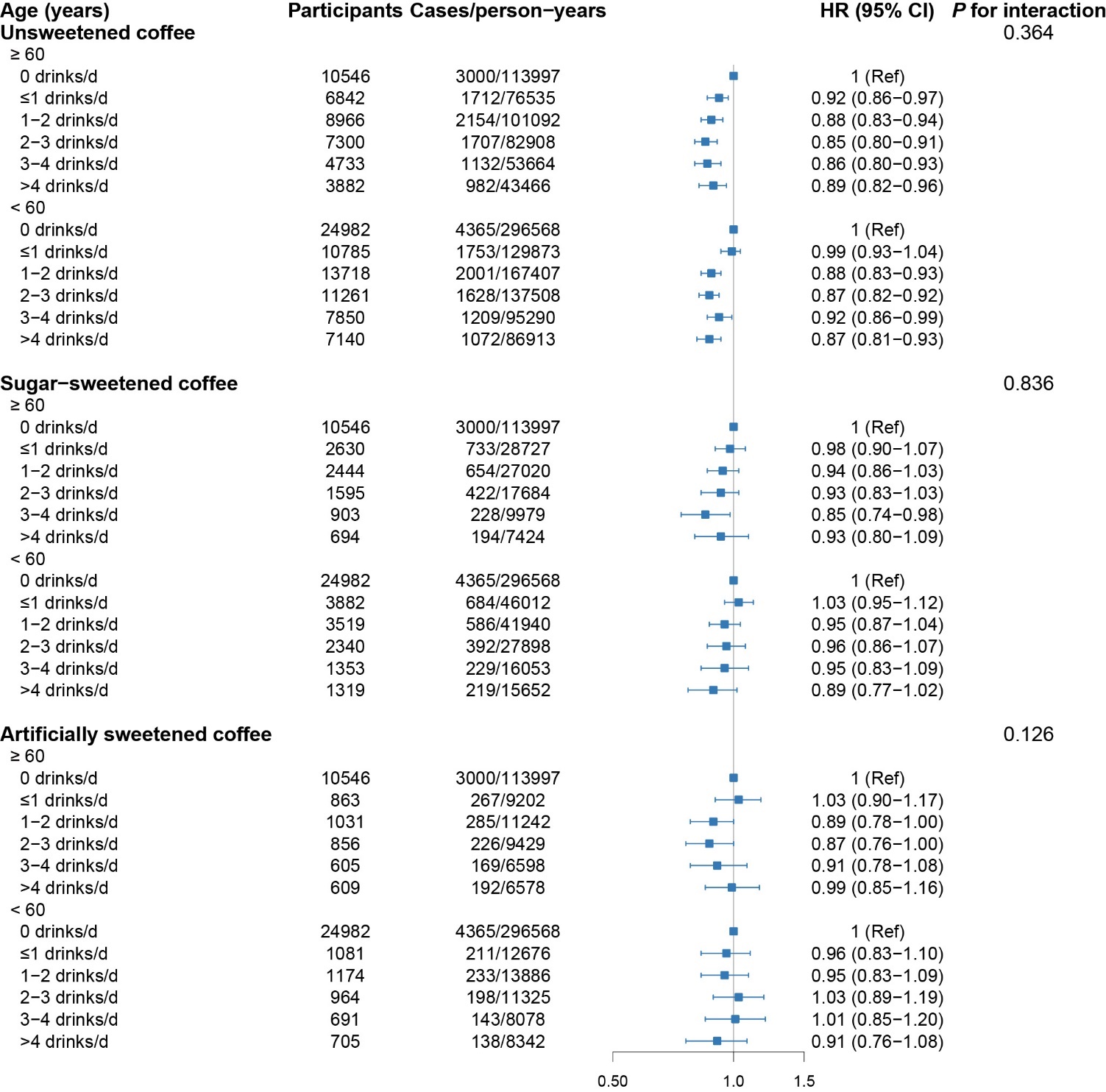


# Supplementary Figure 12. Age-stratified analysis of the association between coffee consumption and incident GI disease in the multivariable model.

Note: The estimated effects are based on fully adjusted models except for the corresponding subgroup covariates. HR, hazard ratio; CI, confidence interval; GI, gastrointestinal.


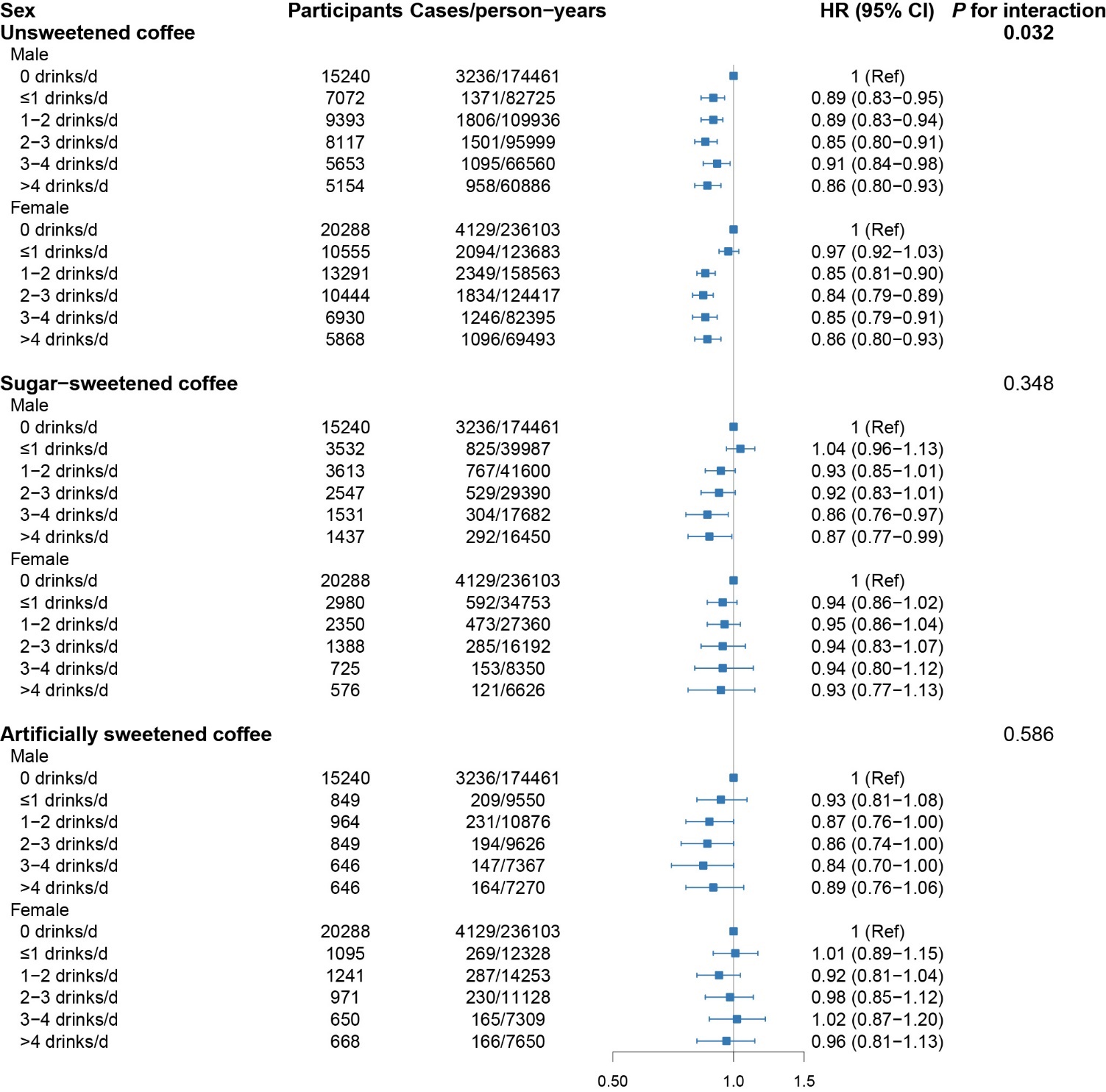


# Supplementary Figure 13. Sex-stratified analysis of the association between coffee consumption and incident GI disease in the multivariable model.

Note: The estimated effects are based on fully adjusted models except for the corresponding subgroup covariates. HR, hazard ratio; CI, confidence interval; GI, gastrointestinal.


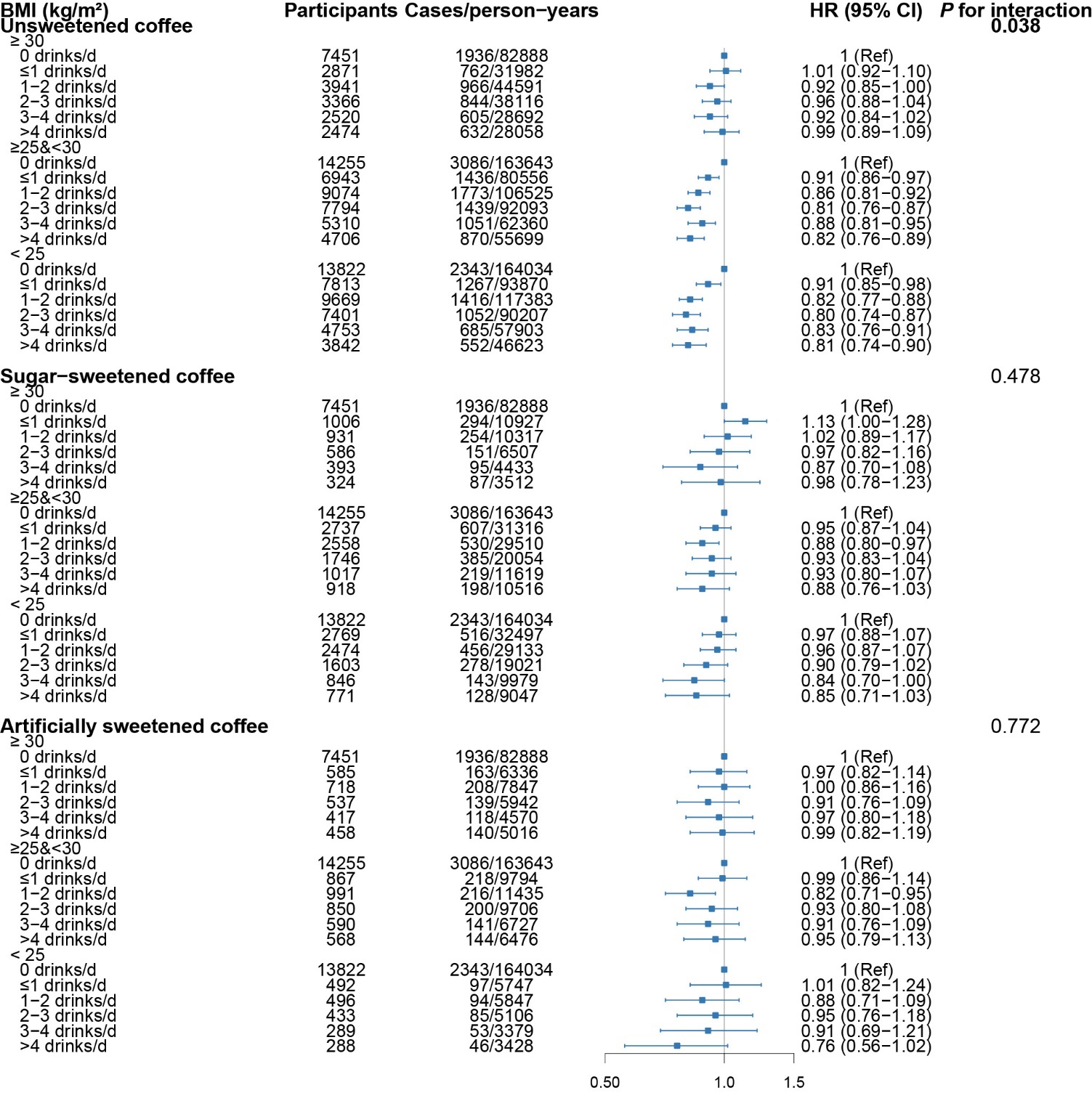


# Supplementary Figure 14. BMI-stratified analysis of the association between coffee consumption and incident GI disease in the multivariable model.

Note: The estimated effects are based on fully adjusted models except for the corresponding subgroup covariates. BMI, body mass index; HR, hazard ratio; CI, confidence interval; GI, gastrointestinal.


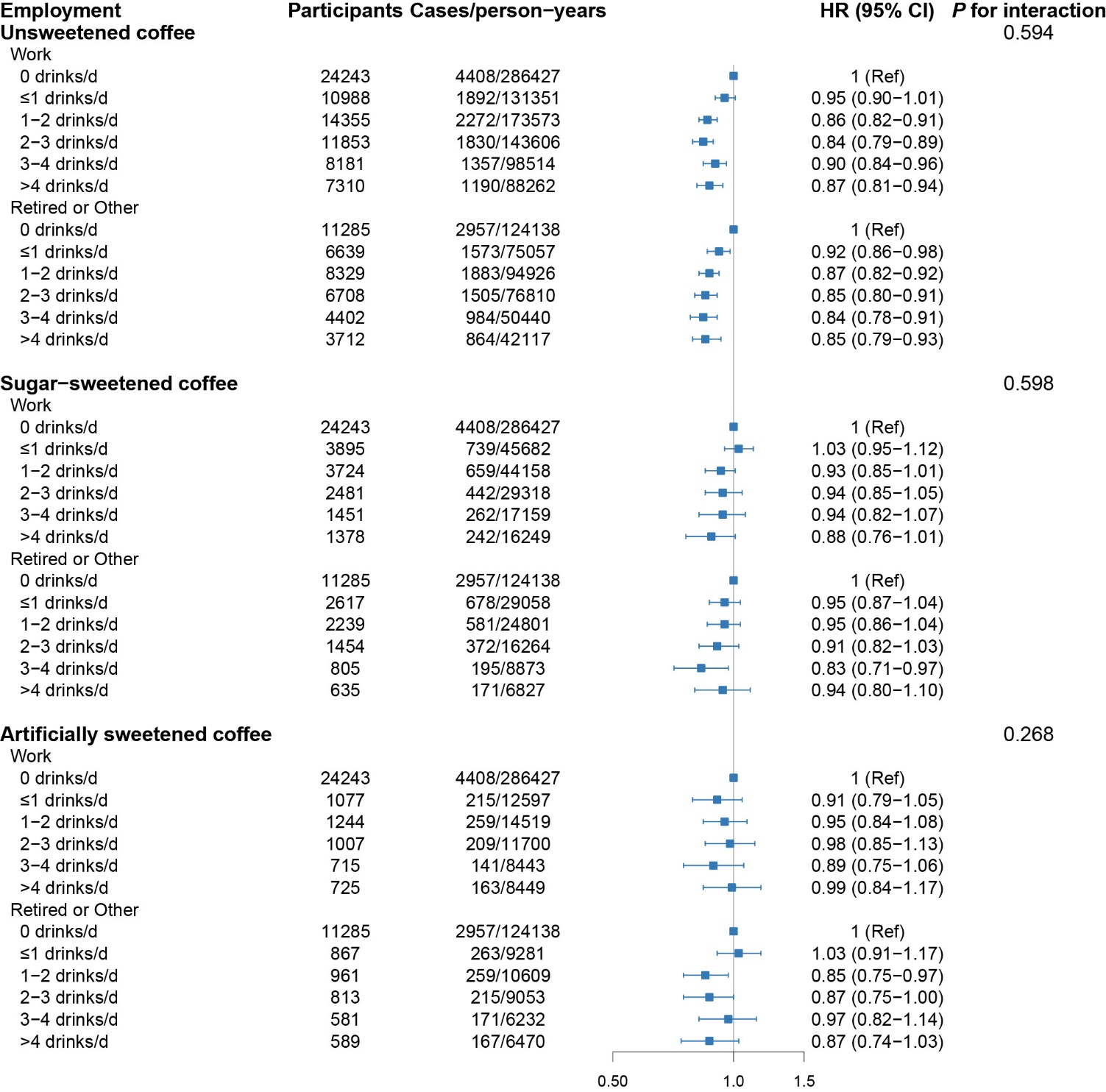


# Supplementary Figure 15. Employment-stratified analysis of the association between coffee consumption and incident GI disease in the multivariable model.

Note: The estimated effects are based on fully adjusted models except for the corresponding subgroup covariates. HR, hazard ratio; CI, confidence interval; GI, gastrointestinal.


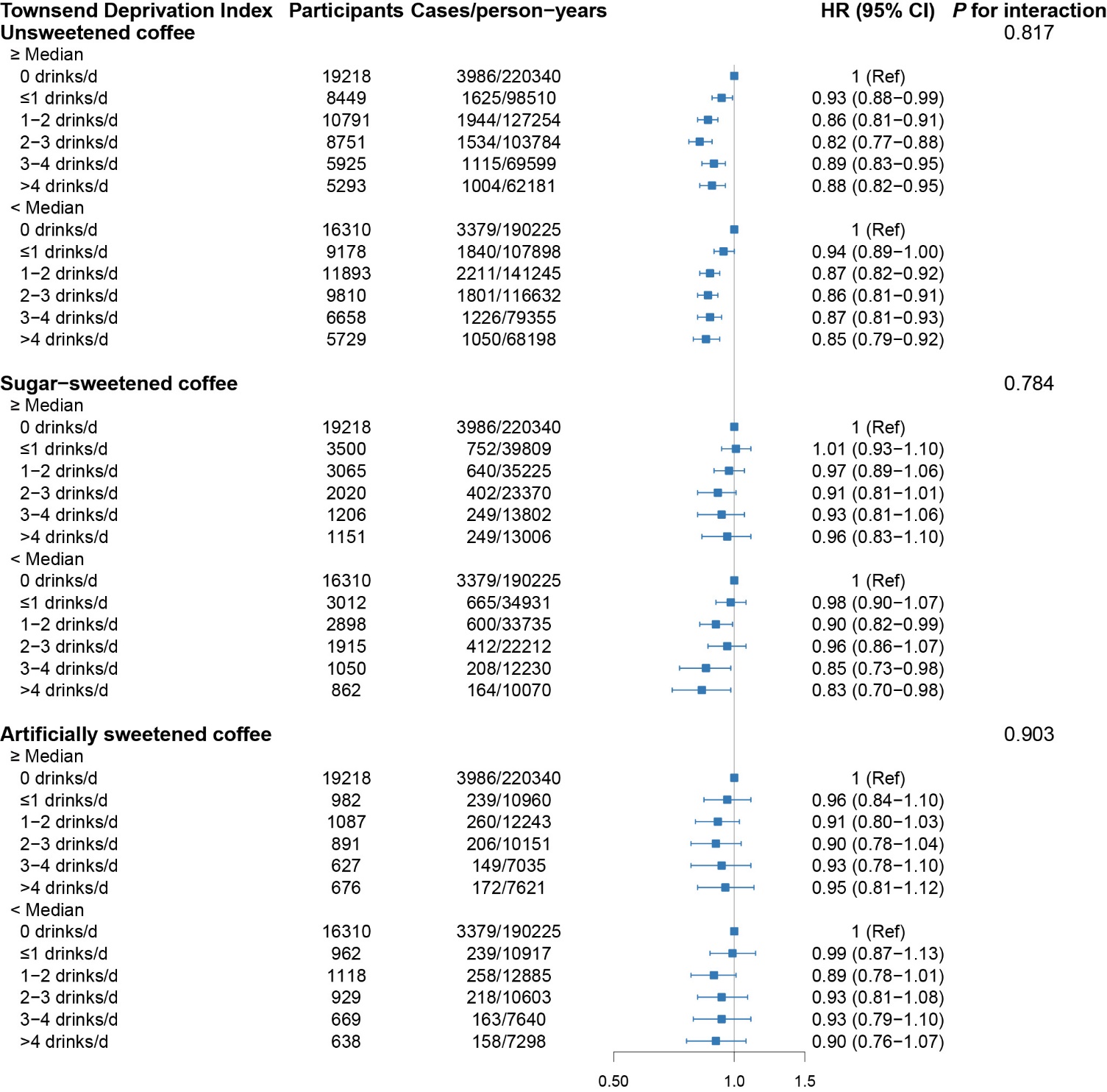


# Supplementary Figure 16. TDI-stratified analysis of the association between coffee consumption and incident GI disease in the multivariable model.

Note: The estimated effects are based on fully adjusted models except for the corresponding subgroup covariates. TDI, Townsend deprivation index; HR, hazard ratio; CI, confidence interval; GI, gastrointestinal.


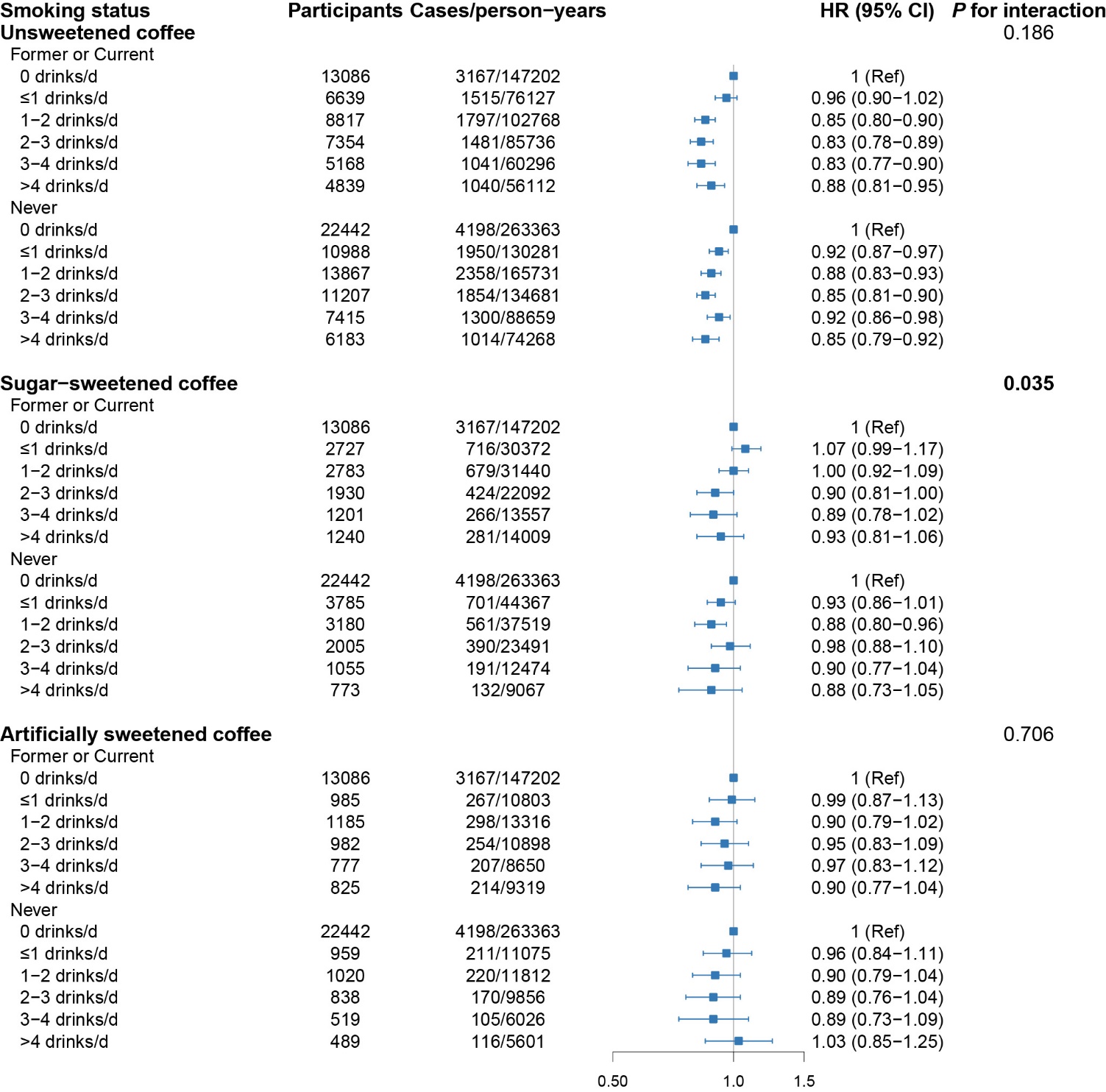


# Supplementary Figure 17. Smoking status-stratified analysis of the association between coffee consumption and incident GI disease in the multivariable model.

Note: The estimated effects are based on fully adjusted models except for the corresponding subgroup covariates. HR, hazard ratio; CI, confidence interval; GI, gastrointestinal.


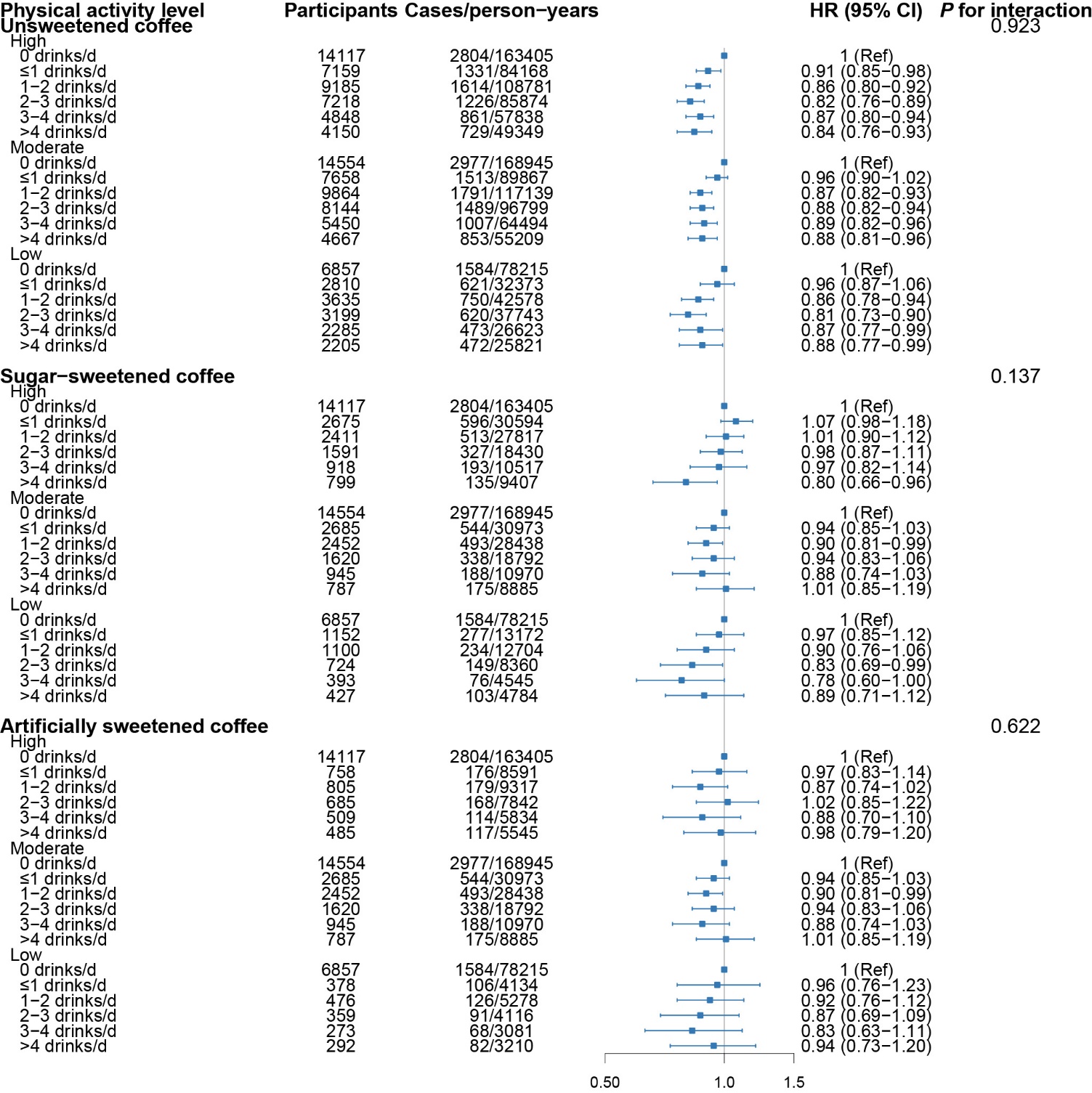


# Supplementary Figure 18. Physical activity level-stratified analysis of the association between coffee consumption and incident GI disease in the multivariable model.

Note: The estimated effects are based on fully adjusted models except for the corresponding subgroup covariates. HR, hazard ratio; CI, confidence interval; GI, gastrointestinal.


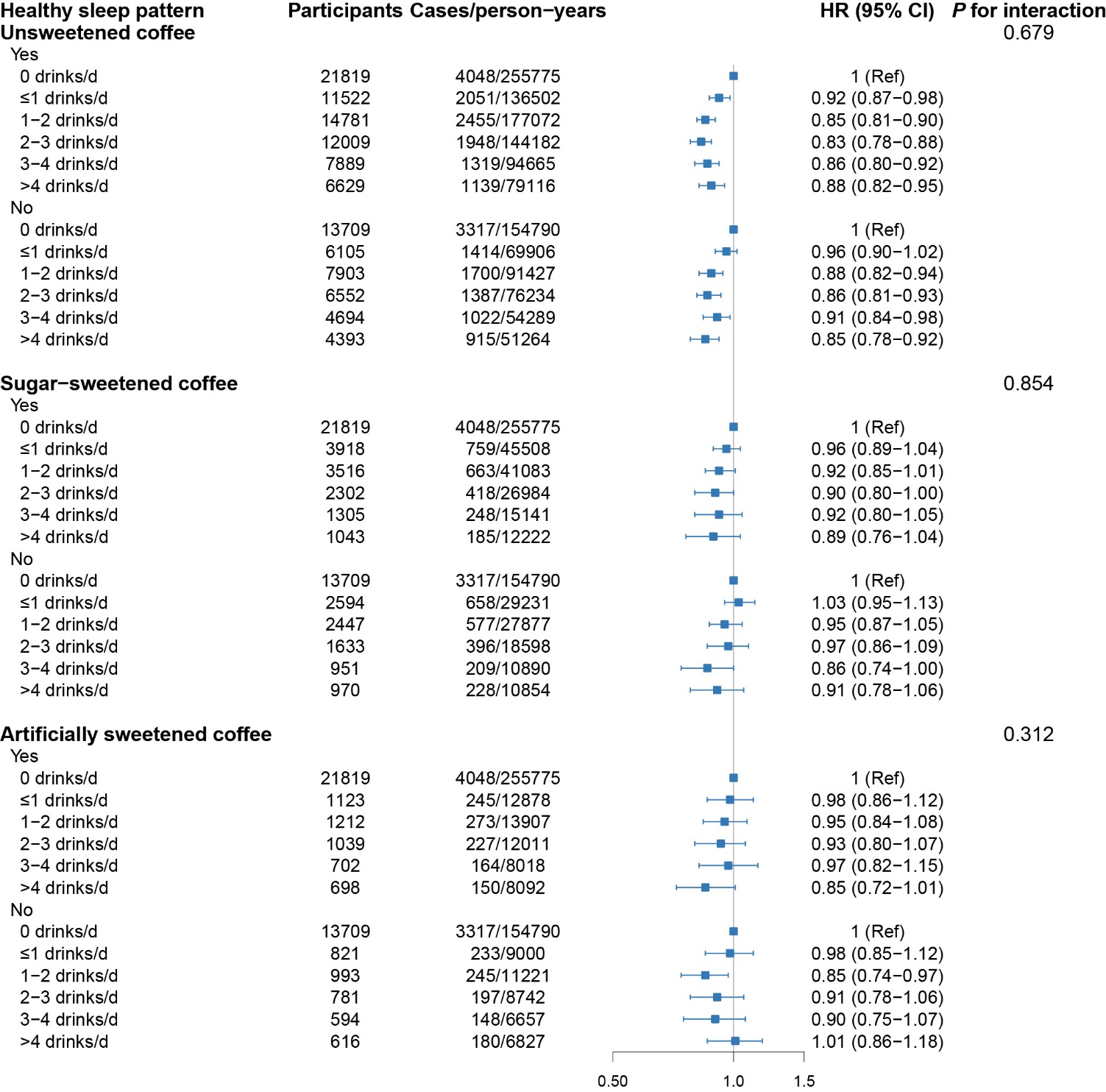


# Supplementary Figure 19. Healthy sleep pattern-stratified analysis of the association between coffee consumption and incident GI disease in the multivariable model.

Note: The estimated effects are based on fully adjusted models except for the corresponding subgroup covariates. HR, hazard ratio; CI, confidence interval; GI, gastrointestinal.


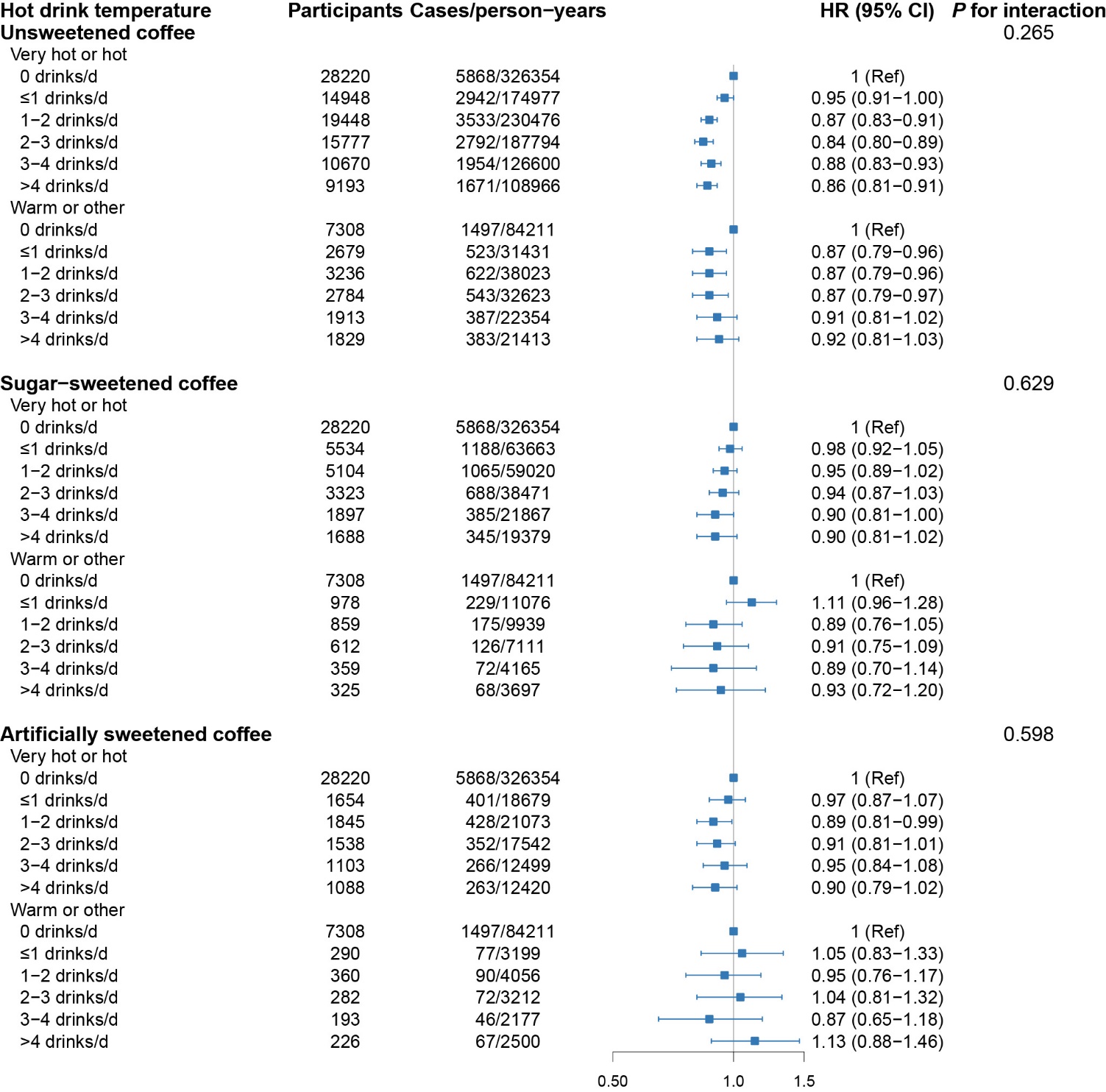


# Supplementary Figure 20. Hot drink temperature-stratified analysis of the association between coffee consumption and incident GI disease in the multivariable model.

Note: The estimated effects are based on fully adjusted models except for the corresponding subgroup covariates. HR, hazard ratio; CI, confidence interval; GI, gastrointestinal.


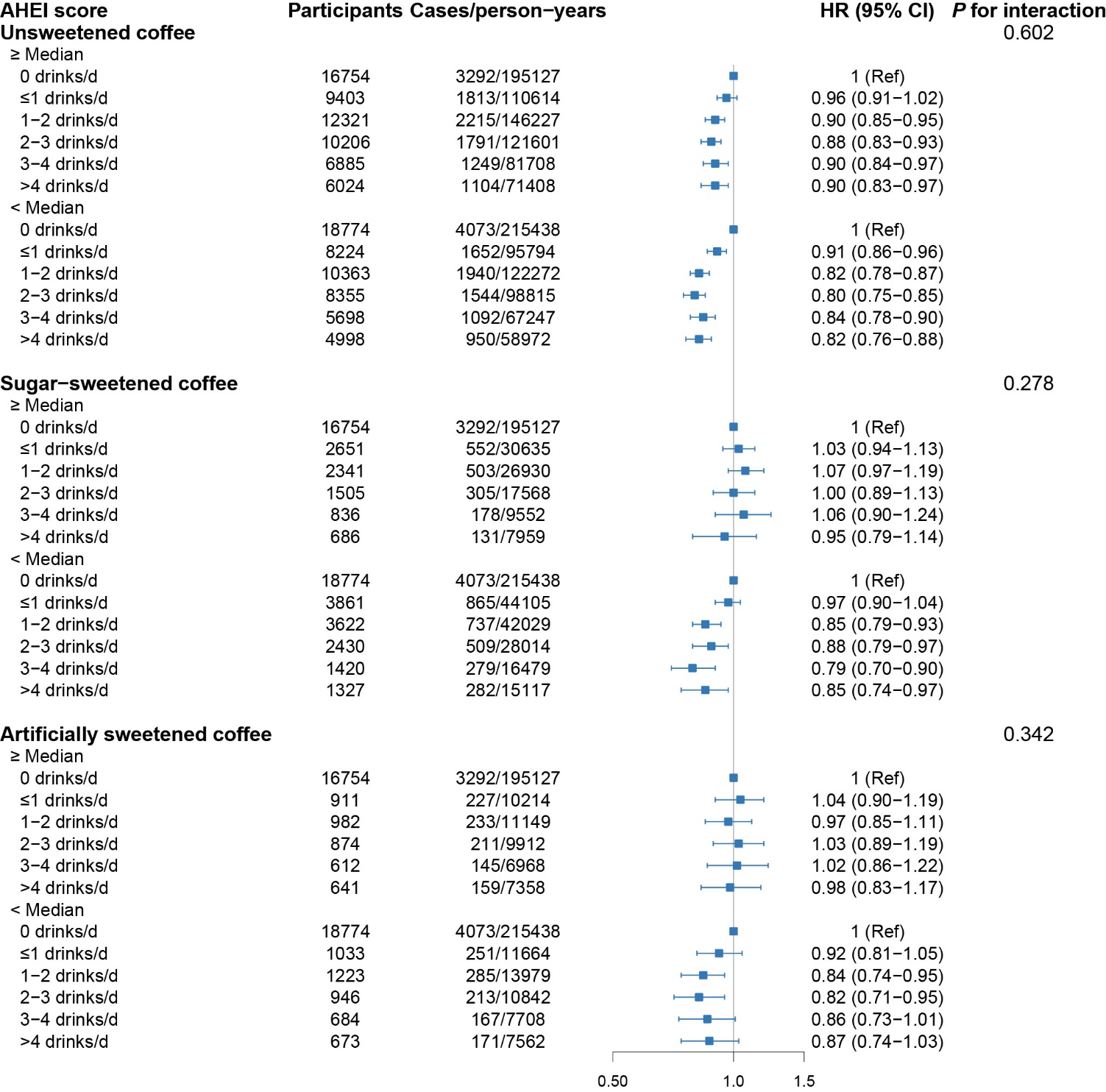


# Supplementary Figure 21. AHEI score-stratified analysis of the association between coffee consumption and incident GI disease in the multivariable model.

Note: The estimated effects are based on fully adjusted models except for the corresponding subgroup covariates. AHEI, Alternative Healthy Eating Index; HR, hazard ratio; CI, confidence interval; GI, gastrointestinal.


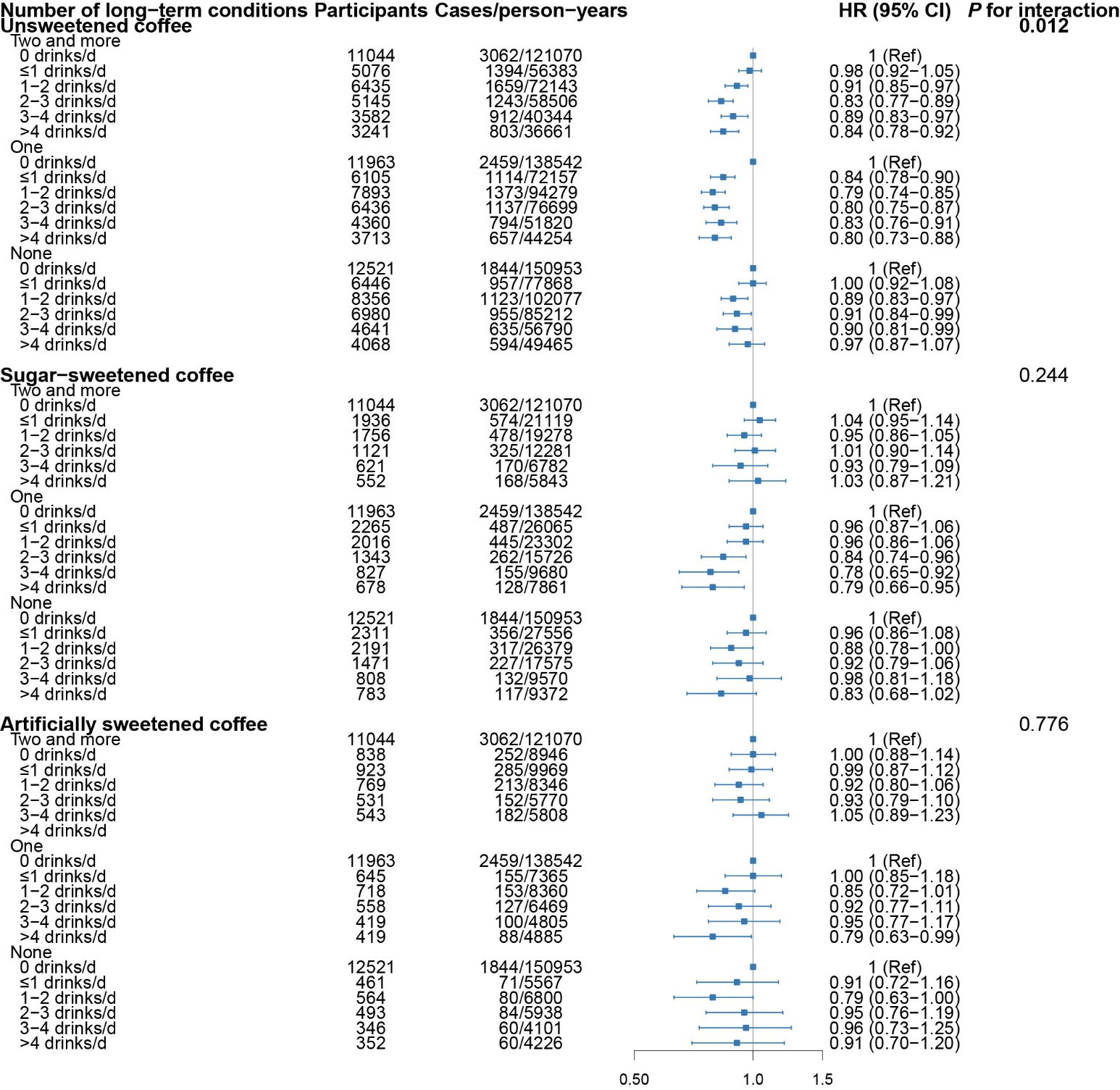


# Supplementary Figure 22. Number of long-term conditions-stratified analysis of the association between coffee consumption and incident GI disease in the multivariable model.

Note: The estimated effects are based on fully adjusted models except for the corresponding subgroup covariates. HR, hazard ratio; CI, confidence interval; GI, gastrointestinal.
